# Synthesis and Biophysical and Biological Studies of *N*-Phenylbenzamide Derivatives Targeting Kinetoplastid Parasites

**DOI:** 10.1021/acs.jmedchem.3c00697

**Published:** 2023-09-20

**Authors:** J. Jonathan Nué-Martinez, David Cisneros, María del Valle Moreno-Blázquez, Cristina Fonseca-Berzal, José Ignacio Manzano, Damien Kraeutler, Marzuq A. Ungogo, Maha A. Aloraini, Hamza A. A. Elati, Alexandra Ibáñez-Escribano, Laura Lagartera, Tomás Herraiz, Francisco Gamarro, Harry P. de Koning, Alicia Gómez-Barrio, Christophe Dardonville

**Affiliations:** † Instituto de Química Médica, IQM−CSIC, Juan de la Cierva 3, E-28006 Madrid, Spain; ‡ PhD Programme in Medicinal Chemistry, Doctoral School, Universidad Complutense de Madrid (UCM), 28040 Madrid, Spain; § Institute of Infection, Immunity and Inflammation, College of Medical, Veterinary and Life Sciences, University of Glasgow, G12 8TA Glasgow, U.K.; ∥ Instituto de Parasitología y Biomedicina “Löpez Neyra”, IPBLN-CSIC, Parque Tecnolögico de Ciencias de la Salud, 18016 Granada, Spain; ⊥ Departamento de Microbiología y Parasitología, Facultad de Farmacia, Universidad Complutense de Madrid (UCM), Plaza de Ramón y Cajal s/n, 28040 Madrid, Spain; # Instituto de Ciencia y Tecnología de Alimentos y Nutrición, ICTAN−CSIC, José Antonio Novais 10, Ciudad Universitaria, 28040 Madrid, Spain

## Abstract

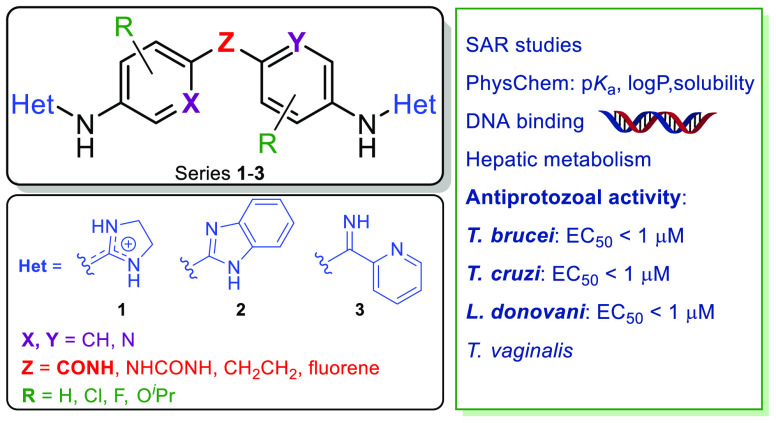

The AT-rich mitochondrial DNA (kDNA) of trypanosomatid parasites is a target of DNA minor groove binders. We report the synthesis, antiprotozoal screening, and SAR studies of three series of analogues of the known antiprotozoal kDNA binder 2-((4-(4-((4,5-dihydro-1*H*-imidazol-3-ium-2-yl)amino)benzamido)phenyl)amino)-4,5-dihydro-1*H*-imidazol-3-ium (**1a**). Bis(2-aminoimidazolines) (1) and bis(2-aminobenzimidazoles) (2) showed micromolar range activity against *Trypanosoma brucei,* whereas bisarylimidamides (3) were submicromolar inhibitors of *T. brucei*, *Trypanosoma cruzi,* and *Leishmania donovani*. None of the compounds showed relevant activity against the urogenital, nonkinetoplastid parasite *Trichomonas vaginalis*. We show that series **1** and **3** bind strongly and selectively to the minor groove of AT DNA, whereas series **2** also binds by intercalation. The measured p*K*_a_ indicated different ionization states at pH 7.4, which correlated with the DNA binding affinities (Δ*T*_m_) for series **2** and **3**. Compound **3a**, which was active and selective against the three parasites and displayed adequate metabolic stability, is a fine candidate for in vivo studies.

## 1 Introduction

Neglected tropical diseases caused by kinetoplastid parasites (i.e., *Trypanosoma cruzi*, *Trypanosoma brucei*, and *Leishmania*) are a great cause of suffering around the world. American and African trypanosomiases, as well as leishmaniasis, threaten millions of people mainly in the least developed countries.^1–4^ Available therapies to treat these illnesses are not satisfactory as they often present poor efficacy against drug-resistant parasite strains or a particular stage of the disease or patient condition (e.g., chronic Chagas disease, late-stage rhodesiense sleeping sickness, and HIV/leishmaniasis coinfection), and habitually require prolonged treatment regimens and high doses, with associated severe side effects.^4–6^ In addition, *T. brucei rhodesiense*, *T. cruzi,* and *Leishmania* spp. are all zoonotic and cause disease in various domestic animals as well, for which the treatment options are even more limited.^7–9^ Hence, new antiprotozoal drugs are needed to improve this far from ideal therapeutic arsenal.

Several classes of promising new chemical entities (NCE) are currently in clinical development under the aegis of the Drugs for Neglected Diseases initiative (DNDi).^10^ For instance, the polyadenylation specificity factor 3 (CPSF3) inhibitor acoziborole has reached phase IIb/III for the single-dose oral treatment of gambiense human African trypanosomiasis (HAT),^11^ and its derivative DNDI-6148 is a lead candidate in phase I for leishmaniasis^12^ and Chagas disease.^13^ Other NCEs being developed for visceral leishmaniasis include the nitroimidazole DNDI-0690, the proteasome inhibitors LXE408 and GSK3494245/DDD01305143, and the cdc2-related kinase 12 (CRK12) inhibitor GSK899/DDD853651.^13^

**Chart 1:**
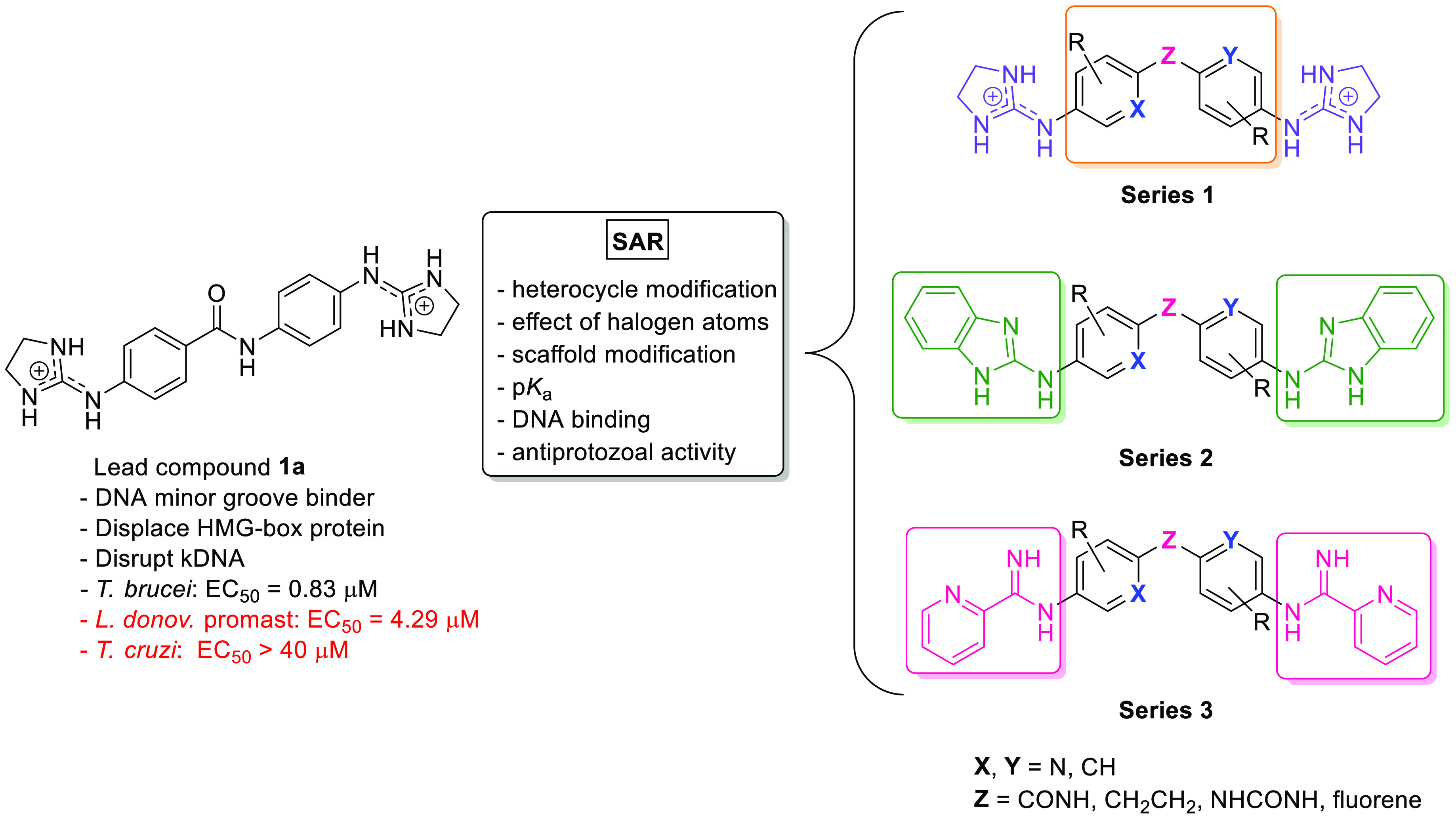
Prototype of Bis(2-aminoimidazoline) DNA Minor Groove Binder Active against *T. brucei* (**1a**) and New Series (**1**, **2**, and **3**) Prepared for SAR Studies

Bis(2-aminoimidazolines) are a class of AT-rich DNA minor groove binders (MGBs) with established in vitro and in vivo efficacy against *T. brucei*. The *N*-phenylbenzamide derivative **1a**, which was curative by oral administration in an acute mouse model of African trypanosomiasis, is the prototype of this series (Chart 1).^14–18^ Strong experimental evidence suggested that this compound can displace High Mobility Group (HMG)-box-containing proteins that are essential for kinetoplast DNA (kDNA) function from their DNA binding sites. This interaction led to the disruption of kDNA and eventually to the death of the parasite.^19^ Hence, these compounds were chosen as starting points to develop new molecules against all three main kinetoplastid parasite species, and potentially also against parasites containing AT-rich nuclear DNA (e.g., *Trichomonas vaginalis*).

In spite of their action on kDNA and their excellent activity against *T. brucei*, bis(2-aminoimidazolines) are significantly less active against *Leishmania* or *T. cruzi*.^16,17^ For instance, lead compound **1a** displays a 5-fold lower activity against promastigotes of *L. donovani* (EC_50_ = 4.29 μM) than against bloodstream forms of *T. b. brucei* (EC_50_ = 0.83 μM)^20^ and was inactive against *T. cruzi* (Table 1). Since a drug targeting the kDNA of intracellular amastigotes of *Leishmania* or *T. cruzi* will have to cross 4 membranes (host cell membrane, parasitophorous vacuole membrane, parasite cell membrane, and mitochondrial membranes) to reach its target, optimum membrane permeability (or efficient, mediated drug uptake) will be required to observe antiparasitic activity against these intracellular forms. We reported before that the chemical modification of bis(2-aminoimidazoline) compounds to lower their p*K*_a_ can lead to a notable improvement in membrane permeability and antitrypanosomal activity of these dicationic compounds.^17,20^

**Table 1:**
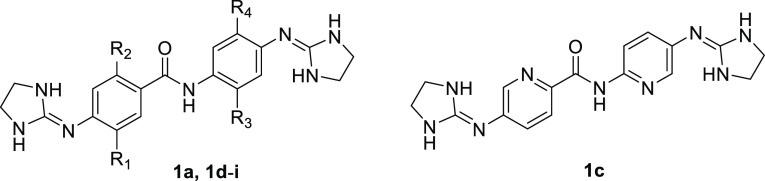
In Vitro Antiprotozoal Activity (EC_50_; μM)^a^ and Cytotoxicity (CC_50_; μM) of Bis(imidazolidin-2-imines) **1a** and **1c**–**i** (Series **1**)

cmpd	R_1_	R_2_	R_3_	R_4_	*T. brucei*			HEK^e^	*T. cruzi*^f^		L929^g^	*L. donovani*^h^		THP-1^i^	*T. vaginalis*^j^	Vero CCL-81
					WT^b^	B48^c^	RF^d^		epimast.	amast.		promast.	amast.		JH31A#4	
**1a**	H	H	H	H	0.83 ± 0.08	0.87 ± 0.2	1.1	>200	>40	nt^k^	>200	4.3 ± 0.54	nt	nt	nt	nt
					(>240)^20^											
**1c**					25.5 ± 3.2	37.5 ± 4.4	1.47	>200	>40	nt	>200	>50	nt	nt	>40	nt
					(>7.8)											
**1d**	Cl	H	Cl	H	nt	nt		>200	>40	nt	>200	>50	nt	nt	>40	nt
**1e**	Cl	H	H	Cl	5.7 ± 0.7	6.9 ± 1.5	1.21	>200	>40	nt	>200	>50	nt	nt	>40	nt
					(>35)											
**1f**	H	Cl	Cl	H	10.4 ± 0.8	14.8 ± 0.8	1.42	>200	>40	nt	>200	>50	nt	nt	>40	nt
					(>19)											
**1g**	H	Cl	H	Cl	47.7 ± 13.2	48.4 ± 3.2	1.01	>100	>40	nt	>200	>20	nt	38.7 ± 1.5	>40	nt
					(>2.1)											
**1h**	H	O^i^Pr	O^i^Pr	H	17.6 ± 2.1	16.0 ± 0.4	0.91	>100	>40	nt	>200	7.6 ± 0.4	>10	>50	>40	nt
					(>5.6)							(>6.5)				
**1i**	F	H	F	H	78.5 ± 6.1	57.4 ± 2.6	0.73	>100	>40	nt	>200	>20	nt	>50	>40	nt
					(>1.3)											
Penta.^l^					0.00034 ± 0.00002	0.111 ± 0.003	325									
					(3852)	(11.8)										
Dimi.^m^					0.010 ± 0.0007	0.012 ± 0.0008	1.23									
					(131)	(109)										
PAO^n^								1.31 ± 0.22								
Benzn.^o^									25.3 ± 2.1	0.5 ± 0.1	>200					
									(>7.9)	(>370)						
AmB^p^												0.07 ± 0.01	0.19 ± 0.05	23.1 ± 4.0		
												(330)	(121.6)			
Metro.^q^															2.56 ± 0.58	>300
															(>117)	

a Data are means ± SEM from three independent experiments (*n* = 3).

b Bloodstream trypomastigotes of *T. b. brucei* wild-type strain s427. The selectivity index is indicated between brackets: SI = CC_50_ (HEK)/EC_50_ (WT).

c *T. b. brucei* strain resistant to pentamidine.^30^

d Resistance factor = EC_50_ (B48)/EC_50_ (WT).

e Human endothelial kidney cells.

f Epimastigotes and intracellular amastigotes of *T. cruzi* strain CL-B5 *lacZ* (DTU TcVI). The selectivity index is indicated between brackets: = CC_50_ (L929)/EC_50_ (*T. cruzi*).

g Cytotoxicity on L929 fibroblasts.

h Promastigotes and intracellular amastigotes of *L. donovani* strain HU3. The selectivity index is indicated between brackets: = CC_50_ (THP-1)/EC_50_ (*L. donovani*).

i Cytotoxicity on THP-1 cells.

j *T. vaginalis* isolate JH31A#4. The selectivity index is indicated between brackets: = CC_50_ (Vero CCL-81)/EC_50_ (*T. vaginalis*).

k Not tested.

l Pentamidine.

m Diminazene.

n Phenylarsine oxide.

o Benznidazole.

p Amphotericin B.

q Metronidazole.

In this study, we decided to combine different chemical strategies to modify the physicochemical properties of lead compound **1a** in order to improve its activity against the intracellular parasites *L. donovani* and *T. cruzi* and conserve its binding affinity toward kDNA. We have shown earlier that the three NH groups of each 2-aminoimidazolinium group of **1a** are crucial for binding to AT-rich DNA because they form hydrogen bonds with thymine and adenine from both DNA strands.^18,19^ In addition, the nitrogen atoms that are not hydrogen bonded to DNA are associated with water molecules.^19^ Thus, new derivatives replacing the 2-aminoimidazoline rings of **1a** by the more acidic 1*H*-benzimidazol-2-ylamine (series **2**) or pyridine-2-carboxamidine (series **3**) heterocycles were synthesized. The central scaffold was also modified by incorporating lipophilic and/or electronegative substituents (F and Cl) and large electron-donating substituents (O^*i*^Pr) (Chart 1). Hence, three families of compounds with structural characteristics related to **1a** were prepared, their physicochemical properties (p*K*_a_, log *P*, and solubility) were determined, and the compounds were assayed in vitro against four protozoan parasites (*T. brucei*, *T. cruzi*, *L. donovani*, and *T. vaginalis*) for SAR studies. To evaluate the potential of the new compounds to target kDNA, we also assessed their DNA binding affinity and mode of binding with AT-rich and GC-rich DNA using different biophysical techniques. Finally, the metabolic stability of one selected candidate, active against all three trypanosomatid parasites, was measured in vitro to assess its potential for further in vivo studies.

## 2 Results

### 2.1 Chemistry

**Scheme 1:**
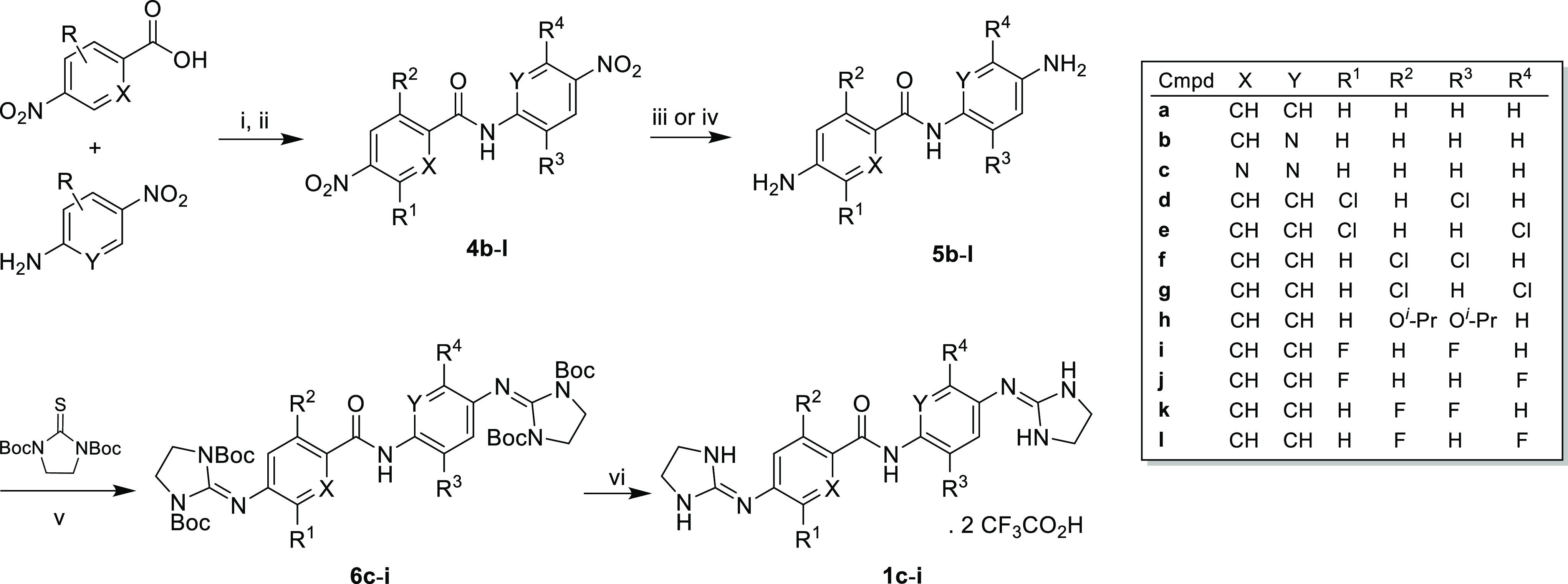
Synthesis of Starting Material Diamines **5b**–**l** and Target Bis(imidazolidin-2-imine) Derivatives **1c**–**i** Reagents and conditions: (i) carboxylic acid, SOCl_2_, 80 °C (for **4b**, **4f**–**l**), or (COCl)_2_, CH_2_Cl_2_, DMF_cat_, 0 °C (for **4c**); (ii) aniline, DIPEA, dry toluene, rt; (iii) SnCl_2_·2H_2_O, HCl_cat._, EtOH, 50 °C (for **5d**–**g**); (iv) H_2_, Pd–C 5%, EtOAc, rt (for **5b**–**c**, **5h**–**l**); (v) **5c**–**i**, di-*tert*-butyl 2-thioxoimidazolidine-1,3-dicarboxylate, HgCl_2_, Et_3_N, DMF, 0 to 60 °C, 30 h–7 days (20–88%); (vi) CH_2_Cl_2_, TFA, 0 °C, 2 h (23–85%).

The starting material dianilines **5b**–**l** were synthesized in two steps from commercially available 4-nitroanilines that were reacted with 4-nitrobenzoyl chlorides to give the 4-nitro-*N*-(4-nitrophenyl)benzamide intermediates **4b**–**l** in good yield (65–96%) (Scheme 1).^20^ The noncommercially available 2-isopropoxy-4-nitrobenzoic acid was synthesized as reported earlier.^21^ The reduction of the nitro groups was carried out by Parr hydrogenation with 5% Pd–C or with tin(II) chloride dihydrate/HCl_cat._ in EtOH at 50 °C^22^ for chlorine-containing compounds (**5d**–**g**). Diamine **5a** was commercially available. Bis(imidazolidin-2-imine) derivatives **1c**–**i** were synthesized from diamines **5c**–**i** using an excess of di-*tert*-butyl 2-thioxoimidazolidine-1,3-dicarboxylate^23^ (3 equiv) in the presence of an excess of triethylamine (7 equiv, Et_3_N) and HgCl_2_ (3 equiv) in dry DMF (Scheme 1).^20^ Since this reaction proceeds slowly at room temperature when working with poorly nucleophilic anilines (e.g., a low conversion rate was observed by HPLC–MS after 7 days), heating at 60 °C was required to reduce the reaction time. The progress and completion of the reaction were monitored by thin-layer chromatography (TLC) and by HPLC–MS.

In most cases, the reaction did not go to completion, and a mixture of mono- and disubstituted compounds was obtained. The crude Boc-protected products were purified by centrifugal PTLC on silica plates previously deactivated with a 9:1 hexane/Et_3_N mixture (**6c**–**h**) or by column chromatography using neutral aluminum oxide (**6i**) (Scheme 1). Removal of the Boc-protecting groups was accomplished with TFA at 0 °C to yield the corresponding bis(imidazolidin-2-imine) compounds **1c**–**i** as trifluoroacetate salts. In this reaction, working at low temperature in the absence of the protic solvent (e.g., using CH_2_Cl_2_) was important to avoid the rupture of the PhN=C bond, resulting in the loss of the imidazoline ring and the generation of the amine starting material. Compound **1i** was obtained in low yield because several recrystallizations were necessary to remove traces of the monosubstituted byproduct.

**Scheme 2:**
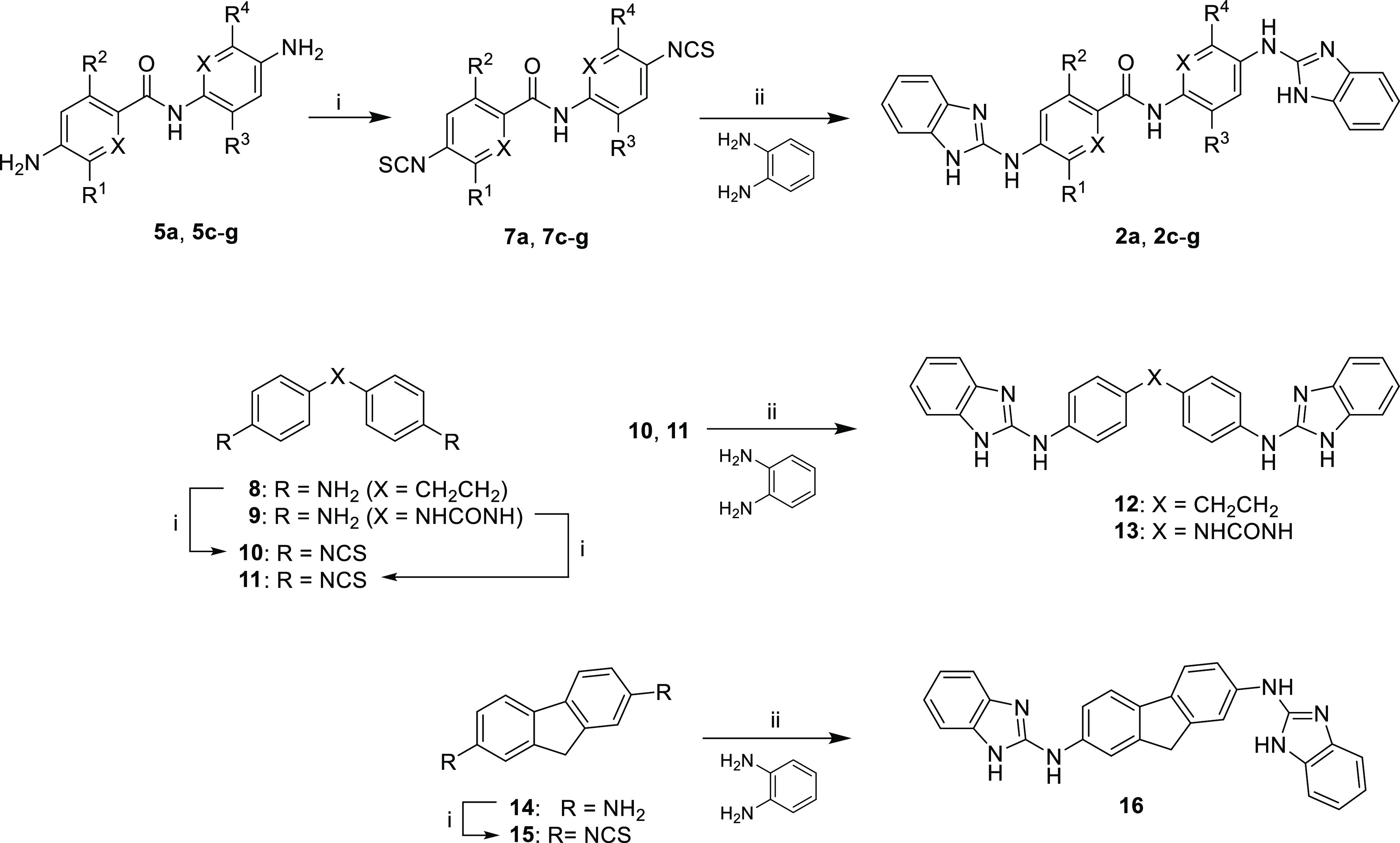
Synthesis of Bis(2-aminobenzimidazoles) **2a**, **2c**–**g**, **12**, **13**, and **16** Reagents and conditions. (i) CSCl_2_, Et_2_O:H_2_O (3:1), rt (24–92%); (ii) benzene-1,2-diamine, EDC·HCl, DMF, 60 °C (15–61%). See Scheme 1 and Table 2 for substituent patterns of **2a** and **2c**–**g**.

Bis(2-aminobenzimidazoles) **2a**, **2c**–**g**, **12**, **13**, and **16** were synthesized by reaction of the isothiocyanate precursors (**7a**, **7c**–**g**, **10**, **11**, and **15**, respectively) with benzene-1,2-diamine, followed by in situ cyclization of the thiourea intermediate using EDC hydrochloride as a promoting agent (Scheme 2). Isothiocyanates **7a, 7c**–**g**, **10**, **11**, and **15** were prepared from the corresponding diamines (**5a, 5c**–**g**, **8**, **9**, and **14**) using thiophosgene.^15^

**Scheme 3:**
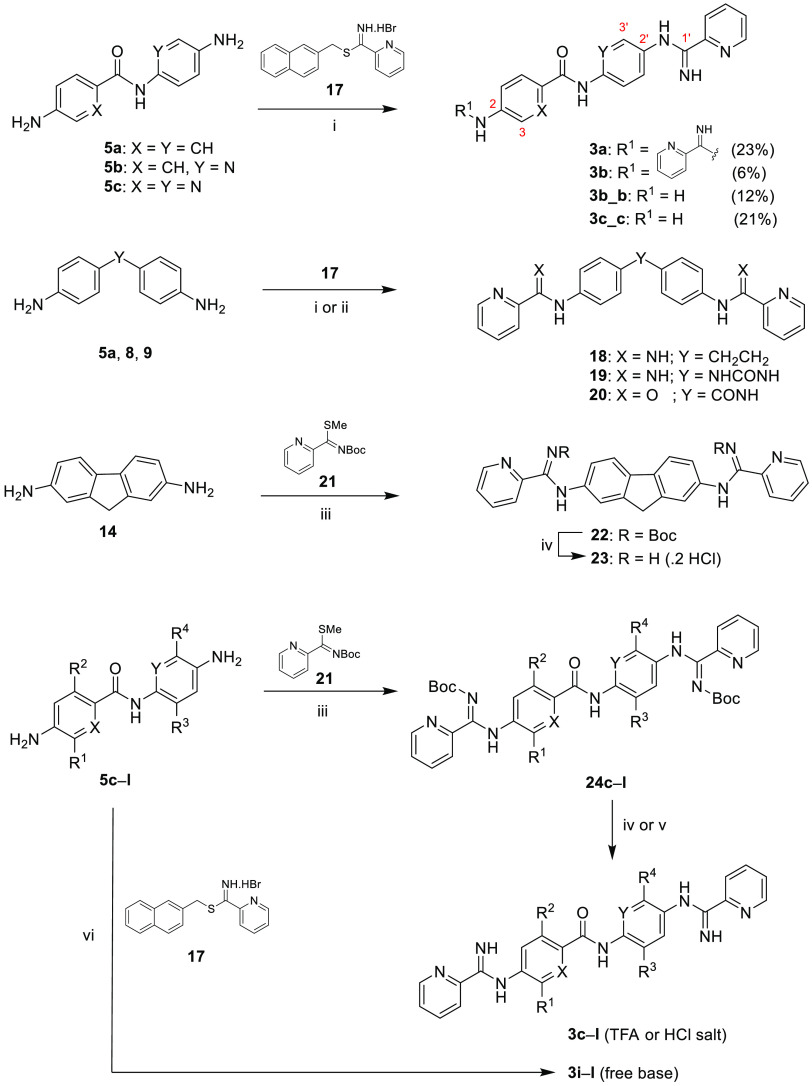
Synthesis of Bis(pyridine-2-carboxamidines) **3a**–**l**, **18**, **19**, and **23** Reagents and conditions: (i) diamine (**5a**–**c**, **8**, **9**), EtOH, CH_3_CN (3:1), rt, 24 h–5 days (6–39%); (ii) diamine **5a**, picolinyl chloride, Et_3_N, THF, rt (94%); (iii) Et_3_N, HgCl_2_, CH_2_Cl_2_, MW, 50 °C, 1 h (12–67%); (iv) CH_2_Cl_2_, TFA, 0 °C (76–97%); (v) CH_2_Cl_2_, 4 M HCl_g_-dioxane solution, 0 °C (65–67%); (vi) DMF, rt, 2–12 days (61–83%). See Scheme 1 and Table 3 for the substituent pattern of **3c**–**l**.

The synthesis of bis(pyridine-2-carboxamidines) **3a**, **3b**, **18,** and **19** was carried out in low yield (6–39%) by the reaction of *S*-(2-naphthylmethyl)-2-pyridylthioimidate hydrobromide^24^**17** with diamines **5a**–**c**, **8**, and **9** in EtOH/CH_3_CN (3:1) following a reported protocol (Scheme 3).^25^ With the aminopyridine scaffolds **5b** and **5c**, the monosubstituted derivatives **3b_b** and **3c_c** were isolated as major byproducts of the reaction by silica chromatography. With **5c**, no disubstituted product (**3c**) was isolated, although it was observed (≈20%) by HPLC–MS in the crude reaction mixture. The substitution pattern of **3b_b** and **3c_c** was determined by ^1^H and ^13^C 2D NMR spectroscopy (COSY, HSQC, and HMBC). For compound **3c_c**, the broad peak at 6.21 ppm integrating for 2H (i.e., free NH_2_) shows strong cross peaks with C-2 (134.5 ppm) and C-3 (119.4 ppm) of the picolinamide ring, implying that **3c**_**c** is substituted via the C-2′ amino group (Scheme 3). The substitution pattern of **3b_b** is similar to that of **3c**_**c**, as shown by the HMBC cross peak between the pyridine H-3′ (8.53 ppm) and the amidine quaternary C-1′ (151.7 ppm) carbon. Of note, the chemical shifts of the C2–NH_2_ (**3c**_**c**: 6.21 ppm, **3b**_**b**: 5.21 ppm) were consistent with the experimental values observed in the starting material diamines **5c** (6.12 ppm) and **5b** (5.06 ppm), respectively.

For the synthesis of halogen-containing bis(pyridine-2-carboxamidines) **3c**–**l**, dianiline precursors **5c**–**l** were reacted with *N*-(*tert*-butoxycarbonyl)pyridine-2-carbimidothioate (**21**) in the presence of HgCl_2_ and Et_3_N (Scheme 3).^26^ We have shown recently that this protocol is useful for the synthesis of bis(arylimidamides) from electron-poor dianilines, where reagent **17** failed to provide the expected products.^26^ Using an excess of the reagents and microwave irradiation as the heating source (50 °C, 1 h), **24c**–**l** were obtained smoothly by silica and/or reverse phase chromatography. Boc group removal was performed with TFA or with 4 M HCl_g_–dioxane solution. This protocol also works satisfactorily with different anilines such as **5a**, **5h**, **8**,^26^**9**, and **14**.

Eventually, we noticed that the yields of the synthesis of bis(pyridine-2-carboxamidines) using reagent **17** were greatly improved when DMF was used as solvent instead of the “classical” mixture of EtOH/CH_3_CN. In fact, even the fluorine-containing compounds **3i**–**l** could be obtained in 61–78% yield using this solvent and 4.5 equiv of **17** (Scheme 3, see method C2 in the Experimental Part).

### 2.2 Biology

#### 2.2.1 Antiprotozoal Activity

The activity of the three series of compounds was tested in vitro against three trypanosomatid parasites (*T. brucei*, *T. cruzi*, and *L. donovani*) and against the urogenital human parasite *T. vaginalis* (Tables 1, 2, and 3). Unspecific cytotoxicity against different mammalian cell lines (i.e., HEK, L929 fibroblasts, THP-1, and Vero CCL-80) was also tested to determine the selectivity indexes (SI) against the parasites.

**Table 2:**
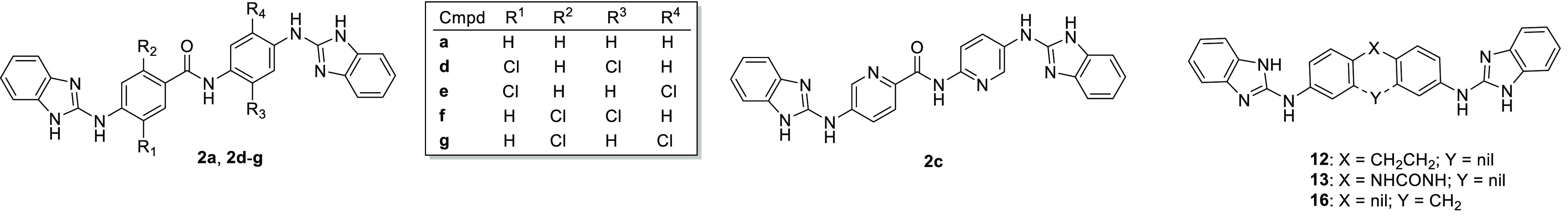
In Vitro Antiprotozoal Activity (EC_50_; μM)^a^ and Cytotoxicity (CC_50_; μM) of Bis(2-aminobenzimidazoles) **2a**–**g**, **12**, **13** and **16** (Series **2**)

cmpd	*T. brucei*			HEK^e^	*T. cruzi*^f^		L929^g^	*L. donovani*^h^		THP-1^i^	*T. vaginalis*^j^	Vero CCL-81
	WT^b^	B48^c^	RF^d^		epimast.	amast.		promast.	amast.		JH31A#4	
**2a**	85.6 ± 2.3^k^	81.9 ± 8.5^k^	0.96	>100	nt	nt	nt	nt	nt	nt	nt	nt
**2c**	11.3 ± 0.2	11.6 ± 0.3	1.03	80.9 ± 2.7	>40	nt	>200	>20	>20	>50	>40	nt
	(7.2)											
**2d**	1.96 ± 0.03	1.93 ± 0.04	0.99	16.8 ± 0.9	>40	nt	<50	>20	>20	>50	>40	nt
	(8.6)											
**2e**	1.68 ± 0.06	1.81 ± 0.06	1.08	20.3 ± 1.9	>40	nt	<50	>20	>20	>50	>40	nt
	(12.1)											
**2f**	5.56 ± 0.26	5.75 ± 0.11	1.03	16.2 ± 0.5	>40	nt	<50	>20	>20	>50	>40	nt
	(2.9)											
**2g**	2.64 ± 0.09	2.93 ± 0.17	1.11	10.2 ± 0.3	>40	nt	<50	>20	>20	>50	>40	nt
	(3.9)											
**12**	2.3 ± 0.2	2.2 ± 0.2	0.95	13.5 ± 0.1	>40	nt	<50	8.7 ± 3.8	>10	14.1 ± 4.4	>40	nt
	(5.9)							(1.6)				
**13**	57.1 ± 1.4	68.8 ± 1.4	1.20	>200	>40	nt	>200	>20	>20	>50	>40	nt
	(>3.5)											
**16**	2.44 ± 0.18	1.73 ± 0.06	0.71	9.15 ± 0.35	>40	nt	<50	>20	>20	12.1 ± 2.6	33.2 ± 2.7	41.4
	(3.8)										(1.2)	
Penta.^l^	0.00034 ± 0.00002	0.111 ± 0.003	325									
	(3852)	(11.8)										
Dimi.^m^	0.010 ± 0.0007	0.012 ± 0.0008	1.23									
	(131)	(109)										
PAO^n^				1.31 ± 0.22								
Benzn.^o^					25.3 ± 2.1	0.5 ± 0.1	>200					
					(>7.9)	(>370)						
AmB^p^								0.07 ± 0.01	0.19 ± 0.05	23.1 ± 4.0		
								(330)	(121.6)			
Metro.^q^											2.56 ± 0.58	>300
											(>117)	

a Data are means ± SEM from three independent experiments (*n* = 3).

b Bloodstream trypomastigotes of *T. b. brucei* wild-type strain s427. The selectivity index is indicated between brackets: SI = CC_50_ (HEK)/EC_50_ (WT).

c *T. b. brucei* strain resistant to pentamidine.^30^

d Resistance factor = EC_50_ (B48)/EC_50_ (WT).

e Human endothelial kidney cells.

f Epimastigotes and intracellular amastigotes of *T. cruzi* strain CL-B5 *lacZ* (DTU TcVI). The selectivity index is indicated between brackets: = CC_50_ (L929)/EC_50_ (*T. cruzi*).

g Cytotoxicity on L929 fibroblasts.

h Promastigotes and intracellular amastigotes of *L. donovani* strain HU3. The selectivity index is indicated between brackets: = CC_50_ (THP-1)/EC_50_ (*L. donovani*).

i Cytotoxicity on THP-1 cells.

j *Trichomonas vaginalis* isolate JH31A#4. The selectivity index is indicated between brackets: = CC_50_ (Vero CCL-81)/EC_50_ (*T. vaginalis*).

k Not tested.

l Pentamidine.

m Diminazene.

n Phenylarsine oxide.

o Benznidazole.

p Amphotericin B.

q Metronidazole.

The bis(2-aminoimidazolines) and the bis(2-aminobenzimidazoles) (series **1** and **2**, respectively) were mostly active against *T. b. brucei* with EC_50_ values in the micromolar range (Tables 1 and 2), whereas the bis(pyridine-2-carboxamidines) (series **3**) displayed low micromolar to submicromolar range activities against all trypanosomatid parasites (Table 3 and SAR studies below). In contrast, only two compounds from series **1** and **2** (**1h**: R_2_ = R_3_ = O^*i*^Pr; and **12**) showed antileishmanial effects in the low micromolar range against promastigotes of *L. donovani* with EC_50_ = 7.6 μM (SI_(THP-1/*L.d*.)_ > 6.5) and 8.7 μM (SI _THP-1/*L.d*._ = 1.6), respectively. Moreover, the compounds were inactive against amastigotes of *L. donovani* at the highest concentration tested (i.e., 20 μM). None of the compounds from series **1** and **2** showed appreciable activity against epimastigotes of *T. cruzi* CL-B5 (DTU TcVI), and as such, they were not assayed on intracellular amastigote forms.

**Table 3:**
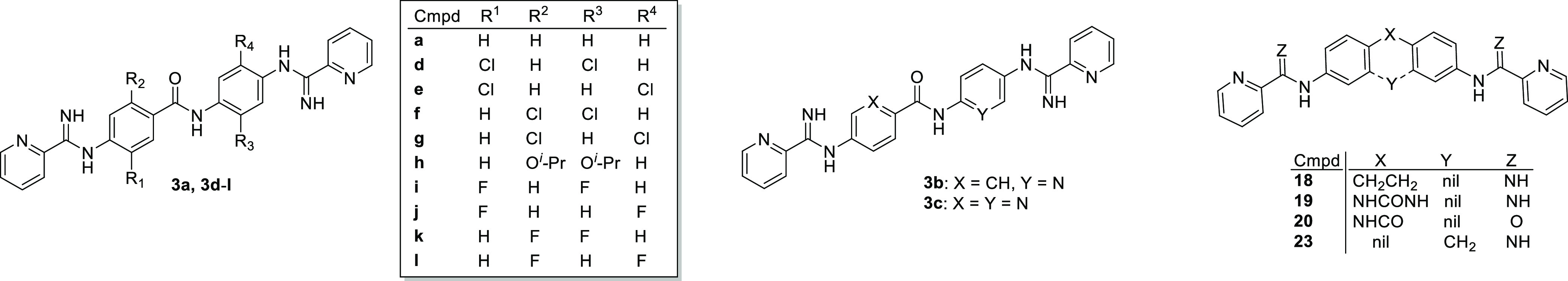
In Vitro Antiprotozoal Activity (EC_50_; μM)^a^ and Cytotoxicity (CC_50_; μM) of Bis(pyridine-2-carboxamidines) **3a**–**l**, **18**, **19**, **20**, and **23** (Series **3**)

cpd	*T. brucei*			HEK^e^	*T. cruzi*^f^		L929^g^	*L. donovani*^h^		THP-1^i^	*T. vaginalis*^j^	Vero CCL-81
	WT^b^	B48^c^	RF^d^		epimast.	amast.		promast.	amast.			
**3a**	0.40 ± 0.02	0.16 ± 0.03	0.4	>200	0.21 ± 0.02	1.28 ± 0.34	89.1 ± 8.3	0.26 ± 0.05	0.65 ± 0.20	>50	>40	nt^k^
	(>500)	(>1250)			(424)	(69.6)			(>76.9)			
**3b**	23.7 ± 0.7	24.0 ± 0.4	1.01	>100	>40	nt	>200	1.47 ± 0.46	>10	>50	>40	nt
	(>4.2)											
3b_b	nt	nt		>100	>40	nt	>200	>20	nt	>50	>40	nt
**3c**	0.12 ± 0.01	0.35 ± 0.02	2.92	14.2 ± 0.8	0.35 ± 0.07	nt	0.79 ± 0.08	0.97 ± 0.24	0.55 ± 0.06	11.2 ± 3.3	32.5	nt
	(118)	(40.5)			(2.3)				(20.3)			
3c_c	nt	nt		>100	8.23 ± 0.91	>10	84.4 ± 8.9	>20	nt	40.2 ± 3.3	nt	nt
					(10.3)	(<8.4)						
**3d**	10.5 ± 1.9	12.5 ± 2.0	1.2	46.0 ± 0.1	0.03 ± 0.00	0.58 ± 0.09	44.9 ± 7.6	1.63 ± 0.29	0.91 ± 0.30	18.6 ± 2.4	>40	nt
	(4.3)				(1495)	(77.3)			(20.4)			
**3e**	0.25 ± 0.04	0.69 ± 0.16	2.76	33.4 ± 3.1	0.06 ± 0.00	0.55 ± 0.17	9.55 ± 1.50	1.44 ± 0.13	0.78 ± 0.11	16.4 ± 1.3	25.7	28.0
	(134)	(48.4)			(159.2)	(17.4)			(21.1)		(1.1)	
**3f**	15.5 ± 2.7	28.1 ± 2.6	1.81	>100	32.7 ± 1.8	nt	>200	>20	nt	>50	>40	nt
	(>6.5)				(>6.1)							
**3g**	9.4 ± 0.96	7.6 ± 0.8	0.81	35.1 ± 3.5	0.03 ± 0.00	0.26 ± 0.04	103.6 ± 10.3	0.89 ± 0.22	2.56 ± 0.41	49.4 ± 6.8	35.1	>40
	(3.7)				(3454)	(398)			(19.3)		(>1.1)	
**3h**	5.07 ± 0.03	3.96 ± 0.22	0.78	69.0 ± 1.7	7.52 ± 0.26	3.51 ± 0.08	85.2 ± 4.8	3.00 ± 0.51	4.19 ± 0.57	>50	>40	nt
	(13.6)				(11.3)	(24.3)			(>11.9)			
**3i**	nt	nt		nt	0.22 ± 0.02	nt	7.51 ± 1.65	nt	nt	nt	11.5	19.5
					(34.1)						(1.7)	
**3j**	nt	nt		nt	0.53 ± 0.08	nt	<6.25	nt	nt	nt	17.0	10.7
					(<11.8)						(1.6)	
**3k**	nt	nt		nt	0.47 ± 0.04	nt	15.9 ± 0.7	nt	nt	nt	>40	nt
					(33.9)							
**3l**	nt	nt		nt	0.15 ± 0.01	nt	24.7 ± 4.1	nt	nt	nt	27.5	>40
					(165)						(>1.4)	
**18**	>100	50.1 ± 2.7	<0.5	>100	4.33 ± 0.65	7.66 ± 0.75	>200	0.33 ± 0.06	1.11 ± 0.23	>50	>40	nt
					(>46.2)	(>26.1)			(>45.0)			
**19**	11.09 ± 0.49	10.77 ± 0.88	0.98	nt	nt	nt	nt	nt	nt	nt	nt	nt
**20**	>100	>100		>100	>40	nt	>200	>20	nt	nt	>40	nt
**23**	5.4 ± 0.3	5.0 ± 0.3	0.93	58.5 ± 6.2	0.84 ± 0.13	1.26 ± 0.30	59.8 ± 8.4	0.77 ± 0.19	0.98 ± 0.12	46.5 ± 3.5	>40	nt
	(10.8)				(71.2)	(47.5)			(47.4)			
Penta.^l^	0.00034 ± 0.00002	0.111 ± 0.003	325									
	(3852)	(11.8)										
Dimi.^m^	0.010 ± 0.0007	0.012 ± 0.0008	1.23									
	(131)	(109)										
PAO^n^				1.31 ± 0.22								
Benzn.^o^					25.3 ± 2.1	0.54 ± 0.1	>200					
					(>7.9)	(>370)						
AmB^p^								0.07 ± 0.01	0.19 ± 0.05	23.1 ± 4.0		
								(330)	(121.6)			
Metro.^q^											2.56 ± 0.58	>300
											(>117)	

a Data are means ± SEM from three independent experiments (*n* = 3).

b Bloodstream trypomastigotes of *T. b. brucei* wild-type strain s427. The selectivity index is indicated between brackets: SI = CC_50_ (HEK)/EC_50_ (WT).

c *T. b. brucei* strain resistant to pentamidine.^30^

d Resistance factor = EC_50_ (B48)/EC_50_ (WT).

e Human endothelial kidney cells.

f Epimastigotes and intracellular amastigotes of *T. cruzi* strain CL-B5 *lacZ* (DTU TcVI). The selectivity index is indicated between brackets: = CC_50_ (L929)/EC_50_ (*T. cruzi*).

g Cytotoxicity on L929 fibroblasts.

h Promastigotes and intracellular amastigotes of *L. donovani* strain HU3. The selectivity index is indicated between brackets: = CC_50_ (THP-1)/EC_50_ (*L. donovani*).

i Cytotoxicity on THP-1 cells.

j *Trichomonas vaginalis* isolate JH31A#4. The selectivity index is indicated between brackets: = CC_50_ (Vero CCL-81)/EC_50_ (*T. vaginalis*).

k Not tested.

l Pentamidine.

m Diminazene.

n Phenylarsine oxide.

o Benznidazole.

p Amphotericin B.

q Metronidazole.

Few compounds displayed marginal trichomonacidal activity: the fluorene-derived bisbenzimidazole **16** and the bis(pyridine-2-carboxamidines) **3c**, **3e**, **3g**, and **3l** exhibited EC_50_ values in the range 25.7–33.2 μM, whereas derivatives **3i** and **3j** were slightly more potent with EC_50_ = 11.5 and 17 μM, respectively (Tables 2 and 3). According to the sequential procedure in this antiparasitic model,^27^ the unspecific cytotoxicity of the above-mentioned compounds was tested against Vero CCL-81 cells. After 24 h in contact with mammalian cells, **16** caused the reduction of about 50% of the cell culture at the highest concentration evaluated (i.e., 40 μM), while **3e**, **3i**, and **3j** presented higher cytotoxicity (CC_50_ = 10–30 μM). Only **3g** and **3l** exhibited minimal cytotoxic effects on mammalian cells at 40 μM. According to the present data, we conclude that these compounds have little or no selective antitrichomonal activity (SI_(VeroCCL-81/*T.v*.)_ < 7). As such, we focus below on their antikinetoplastid properties.

#### 2.2.2 SAR Studies. Bis(2-aminoimidazoline) Series **1** (Table 1)

Most of the chlorine-containing derivatives (**1e**–**g**) showed activity against both WT and drug-resistant (B48) strains of *T. b. brucei* in the low to medium micromolar range (EC_50_ = 5.7 to 48.4 μM). Compound **1e** (R_1_/R_4_ = Cl; EC_50_ = 5.7 μM), with both chlorine atoms in positions *ortho* to the imidazoline rings, was approximately 2- and 8-times more potent than the analogues with just one Cl atom *ortho* to the C=O group (R_2_ = Cl; **1f** and **1g**, respectively). In fact, **1e** was the most active and selective compound among the bis(2-aminoimidazoline) derivatives with SI_(HEK/*T.b*.)_ > 35. The introduction of two fluorine atoms in positions R_1_ and R_3_ led to a drastic drop (**1i**) of the anti-*T. brucei* activity with respect to the unsubstituted lead **1a**. In contrast, the introduction of hydrophobic isopropoxy moieties in the scaffold (**1h**: R_2_/R_3_ = O^*i*^Pr) provided a micromolar range trypanocide (EC_50_ = 17.6 μM, SI_(HEK/*T.b*.)_ > 5.6). Replacement of the *N*-phenylbenzamide scaffold of **1a** with a *N*-(pyridin-2-yl)picolinamide skeleton (**1c**) was detrimental (25.5 μM, SI > 5.7). Importantly, the cytotoxicity assays against both HEK cells and L929 fibroblasts showed that the bis(imidazolidin-2-amino) derivatives are nontoxic to mammalian cells, consistent with previous observations.^18,20,28^ Although **1i** was borderline more active against B48 parasites (RF = 0.73; *P* = 0.051), the compounds were equipotent against the multidrug-resistant strain B48 (i.e., RF ≈ 1, *P* > 0.05), indicating that the P2 and aquaporin-2 (AQP2) transporters are not involved in its uptake, unlike the melaminophenyl arsenical class of trypanocides and the diamidine minor groove binders like diminazene, DB75, and pentamidine.^29–31^ As resistance to those drugs is associated with changes in the P2 and AQP2 transporters, nondependence on these drug transporters ensures that no cross-resistance with the existing diamidine and arsenical trypanocidal drugs is likely to occur.^32^

#### 2.2.3 Bis(2-aminobenzimidazole) Series **2** (Table 2)

Six compounds (**2d**–**g**, **12**, and **16**) displayed EC_50_ values <6 μM against both the WT and B48 strains of *T. brucei*, with selectivity indexes vs HEK cells ranging from 2.9 (**2f**) to 12.1 (**2e**). This represented a >15-fold increase in activity compared to unsubstituted compound **2a** (EC_50_ = 85.6 μM). Like series **1**, chlorine atoms *ortho* to both 2-aminobenzimidazole moieties produced the best anti-*T. brucei* compound (**2e**, R_2_/R_4_ = Cl; EC_50_ = 1.68 μM, SI_(HEK/*T.b*.)_ = 12.1). In contrast, the presence of one (**2g**) or two chlorine atoms (**2f**) *ortho* to the amide linker was unfavorable, with a loss of activity [1.5-fold (*P* = 5.4 × 10^–5^) and 3.5-fold (*P* = 1.1 × 10^–6^), respectively, *n* = 5)] and selectivity (3–4 fold) relative to **2e**. Replacement of the *N*-phenylbenzamide scaffold with *N*-(pyridin-2-yl)picolinamide (**2c**) also resulted in a loss of activity against *T. brucei* (*P* = 3.8 × 10^–11^, *n* = 5), as well as a decrease of the cytotoxicity against mammalian (HEK) cells (CC_50_ = 80.9 μM, *P* = 1.2 × 10^–4^, *n* = 3). Replacement of the *N*-phenylbenzamide scaffold with 1,2-diphenylethane (**12**) or 9*H*-fluorene (**16**) maintained the potency against *T. brucei* compared to the best compound **2e** (*P* > 0.05). In contrast, a 1,3-diphenylurea scaffold (**13**) led to an almost complete loss of activity vs **2e** as well as the lowest cytotoxicity against HEK cells. The effect of replacing the 2-aminoimidazoline rings with 2-aminobenzimidazole heterocycles was dramatic, with a 100-fold lower activity against *T. brucei* for analogue **2a** (EC_50_ = 85.6 μM) compared to lead **1a** (EC_50_ = 0.83 μM, *P* = 2.0 × 10^–6^).

#### 2.2.4 Bis(pyridine-2-carboxamidine) Series **3** (Table 3)

Within this series, the anti-*T. brucei* activity was disparate. Three compounds (**3a**: R_1_–R_4_ = H, **3e**: R_1_/R_4_ = Cl, and **3c**: X/Y = N) displayed submicromolar EC_50_ values against *T. brucei* WT (i.e., 0.4, 0.25, and 0.12 μM, respectively) with SI_(HEK/WT)_ > 118. The rest of the compounds were either effective in the micromolar range but relatively poorly selective (**3b**, **3d**, **3f**–**h**, 1,3-diphenylurea **19**, and fluorene **23**) or inactive (1,2-diphenylethane **18** and **20**). Several compounds, **3a**, **3h**, and **18**, were marginally more active against B48 (RF < 0.81), whereas compounds **3c** and **3f** were slightly less potent against the strain B48 (RF = 1.81 and 2.92, respectively; *P* < 0.05), although the modest difference, compared to pentamidine controls (RF = 325), appears to indicate that uptake mechanisms other than TbAQP2 and TbAT1/P2 are principally involved. The rest of the active compounds (**3b**, **3d**, **3e**, **3g**, and **23**) were equipotent against the multidrug-resistant strain B48 (i.e., RF ≈ 1; *P* > 0.05), and it is clear that the P2 and TbAQP2 transporters do not play a major role in the uptake of this chemical class. Compound **3a** (R_1_–R_4_ = H), which displayed high efficacy and selectivity (EC_50_ = 0.16 μM, SI_(HEK/B48)_ > 1250) similar to the control drug pentamidine (EC_50_ = 0.11 μM) against the multidrug resistant strain B48 and much more active than most of the other current trypanocides against wild-type *T. brucei* (e.g., nitrofuran nifurtimox, EC_50_ = 2.4 μM, nitroimidazole fexinidazole, 1.0 μM,^33^ difluoromethylornithine, 22 μM^34^), emerged as a promising lead compound against *T. brucei*.

The anti-*T. cruzi* activity of series **3** was outstanding, with EC_50_ values on epimastigotes in the midnanomolar range and SI_(L929/epi)_ from 159 to 3454 for the chlorine-containing derivatives **3e** (R_1_/R_4_ = Cl) < **3d** (R_1_/R_3_ = Cl) < **3g** (R_2_/R_4_ = Cl). These were 9-, 7-, and 5-fold more active than the fluorine-containing counterparts **3j**, **3i**, and **3l**, respectively. In contrast, the fluorine-containing analogue **3k** (R_2_/R_3_ = F) was 69 times more potent than its chloro analogue **3f**. This difference could be due to the capacity of fluorine atoms in position *ortho* to the amide bridge to form intramolecular hydrogen bonds (IMHB) with the NH group, thus shielding polarity and improving membrane permeability.^35^ Other compounds displayed activities in the submicromolar [i.e., **3a** (R_1_–R_4_ = H) > fluorene **23** > **3c** (X/Y = N)] and low micromolar range (**18** > **3h**). The anti-*T. cruzi* activity against intracellular amastigotes was maintained for most series **3** compounds, although a loss of potency of ∼6 to 20-fold was observed for **3a**, **3d**, **3e**, and **3g**; even in those cases, however, the activity remained very promising (amastigote EC_50_ = 0.26–1.28 μM). Remarkably, the activity of compound **3h**, which has two large hydrophobic isopropoxy groups (R_2_/R_3_ = O^*i*^Pr), was twice as active against intracellular amastigotes as against epimastigotes, probably indicating that these substituents are favorable for crossing the host cell membrane (i.e., to reach the intracellular parasites), although the mechanism by which this occurs has not yet been studied and the compound was nontoxic to HEK cells. This trend was confirmed with intracellular amastigotes of *L. donovani*, which were equally susceptible to **3h** as promastigotes, and it could be noted that the addition of such groups would be deleterious to recognition by at least some protozoan drug transporters.^36,37^ Moreover, the rigid tricyclic fluorene derivative **23** was equally active against epimastigotes and intracellular amastigotes of *T. cruzi* (EC_50_ = 0.84 and 1.26 μM, respectively; *P* > 0.05). Importantly, all the compounds were selective toward *T. cruzi* with SI_(L929/amast)_ ranging from 17.4 (**3e**) to 398 (**3g**) against intracellular amastigotes. Compound **3g** (EC_50_ = 0.26 μM; SI_(L929/amast)_ = 398, SI_(L929/epi)_ = 3454), which is twice as potent and displays a similar SI as the reference drug benznidazole against amastigotes, emerged as a very interesting lead compound against *T. cruzi*.

All of the bis(arylimidamides), except **3f**, inhibited the growth of promastigotes of *L. donovani* with EC_50_ values ranging from 0.26 (**3a**) to 3.0 μM (**3h**). This represented a clear improvement in antileishmanial activity versus the bis(2-aminoimidazoline) lead compound **1a** (EC_50_ = 4.29 μM; *P* < 0.05, *n* = 3). In general, the antileishmanial activity was maintained against intracellular amastigotes, although with a small loss of potency (approximately 1.5 to 3-fold) for **3a**, **3g**, **3h**, **18**, and **23** (amastigote EC_50_ = 0.65–4.19 μM). The best activities were in the same range as those of the reference drug amphotericin B (EC_50_ = 0.19 μM). In contrast, the presence of chlorine atoms *ortho* to the 4-picolinimidamido groups (**3d**: R_1_/R_3_ = Cl; 0.91 μM and **3e**: R_1_/R_4_ = Cl; 0.78 μM) or two nitrogen atoms next to the amide linker (**3c**, X/Y = N) promoted the antileishmanial activity against intracellular amastigotes, as shown by the nearly 2-fold lower EC_50_ values versus promastigotes [(*P* = 0.0424, *P* = 0.0404, and *P* = 0.0026, respectively, *n* = 3)] (Table 3). Remarkably, the closely related analogue **3b** (X = CH, Y = N) was ineffective against amastigotes of *L. donovani* and epimastigotes of *T. cruzi*, probably accounting for its inability to form an IMHB with the NH of the amide group (vide supra).

Moreover, the compounds displayed a good selectivity toward intracellular forms of *L. donovani*, with SI_(THP-1/amast)_ from >11.9 for the least active compound **3h** to >76.9 for the most effective one (**3a**). Thus, compound **3a**, which displayed submicromolar efficacy against amastigotes and excellent selectivity, emerged as a new antileishmanial hit compound. The lack of antiprotozoal activity of **20** versus **3a** indicates that the amidine structure is crucial for the activity of this series. Moreover, two pyridine-2-carboxamidine groups are needed for the compounds to display potent antitrypanosomatid activity, as shown by the lack of activity of the monosubstituted compounds **3b_b** and **3c_c** versus **3b** and **3c**, respectively. This is consistent with previous reports showing that monoarylimidamide derivatives are generally less potent than bis(arylimidamides), even though submicromolar in vitro activities against *L. donovani* have been reported for several compounds.^38,39^

#### 2.2.5 DNA Binding Affinity. Thermal Melting Assays

Since kDNA is a potential target of these compounds, we assessed their binding properties with AT-containing DNA using thermal melting (*T*_m_) assays. Thermal denaturation can be used to measure the stabilization effect (i.e., the binding affinity) produced by a molecule on binding to duplex DNA. Hence, the *T*_m_ increase (Δ*T*_m_) for compound complexes relative to uncomplexed DNA provides an estimation of the DNA binding affinity of a compound. Δ*T*_m_ values were determined by circular dichroism spectroscopy with a DNA hairpin duplex containing CGATATATATCG [“(AT)_4_”] (Table 4). With the exception of **1e** (Δ*T*_m_ = 0.9 °C), the *T*_m_ increases measured for **1c**–**i** ranged from 3.1 °C (**1h**) to 8.2 °C (**1c**) and were larger than Δ*T*_m_ of lead **1a** (1.6 °C). This showed that the introduction of Cl, O^*i*^Pr or nitrogen atoms in the scaffold positively affected the binding affinity of bis(2-aminoimidazoline) derivatives to (AT)_4_ DNA. No binding to the (CG)_4_-containing hairpin was observed, as illustrated by **1g** (Δ*T*_m_ = 0 °C), which indicated selective binding to AT-DNA. Of note, the big difference between the value reported for lead **1a** with poly(dA·dT)_2_ (Δ*T*_m_ = 47.1 °C),^18^ and our value (Δ*T*_m_ = 1.6 °C) may be attributed to the different oligonucleotides used in the two experiments. DNA containing an A-tract has a narrow minor groove and can bind minor-groove agents without a significant average change in groove width or local helix axis bends in solution. In contrast, straight alternating AT sequences (i.e., (AT)_4_ used in our assays) require the minor groove to narrow with bending of the helix upon binding minor-groove agents.^40^

**Table 4:** Physicochemical Parameters and DNA Binding Affinity: Thermal Melting Increases (Δ*T*_m_) and Binding Constants Determined by SPR-Biosensor Experiments

	physicochemical parameters			DNA binding affinity		
cmpd	p*K*_a_ ± SD^a^	% ionization	log *P*^d^	Δ*T*_m_ (°C)^f^	*K*_D_ × 10^–^^6^ M^g^	
	(p*K*_a1_, p*K*_a2_)^b^	at pH 7.4^c^	(exp. log *P*)^e^	(AT)_4_	A_2_T_2_^h^	(CG)_4_^h^
**1a**	9.29 ± 0.07^20^	98.7	0.62	1.6	0.17^i^	>100^j^
	(9.20 ± 0.02; 10.26 ± 0.05)	(98.4; 99.9)	(0.21)^e^			
**1c**	8.17 ± 0.07	85.5	–0.91	8.2	0.38	>100
	(1.03 ± 0.01^o^; 8.08 ± 0.00; 9.24 ± 0.03)	(82.7; 98.6)				
**1d**	8.20 ± 0.10	86.3	1.65	7.3	45^k^	-l
	(8.05 ± 0.05; 9.13 ± 0.03)	(81.7; 98.2)	(1.36)^e^			
**1e**	8.24 ± 0.03	87.4	1.65	0.9	0.75	>100^j^
**1f**	8.60 ± 0.25	94.1	1.65	5.5	3.2	>100^j^
**1g**	8.82 ± 0.23	96.3	1.65	7.7	3.5	>100^j^
						
**1h**	9.09 ± 0.06	98	1.62	3.1	55^k^	-l
	(8.87 ± 0.04; 10.09 ± 0.02)	(96.6; 99.8)				
**1i**	nd		0.9	nd	0.55	-l
**2a**	4.49 ± 0.08	0.12	5.82	4.9	-l	-^*l*^
**2c**	3.85 ± 0.16	0.03	4.3	5.6	-l	-^*l*^
	(4.62 ± 0.01; 5.79 ± 0.01; 11.49 ± 0.00^p^)	(0.16; 2.4)				
**2d**	3.39 ± 0.07	0.01	6.86	5	-l	-*^l^*
**2e**	(5.06 ± 0.01; 6.20 ± 0.04; 11.83 ± 0.11^p^)	(0.45; 5.9)	6.86	5.1	-l	-*^l^*
			(3.21)^e^			
**2f**	3.41 ± 0.06	0.01	6.86	4	-l	-*^l^*
**2g**	3.38 ± 0.16	0.01	6.86	5.3	-l	-*^l^*
**12**	5.64 ± 0.08	1.71	7.24	6.1	-m	
	(5.43 ± 0.03; 6.68 ± 0.08)	(1.1; 16.0)	(4.32)^e^			
**13**	5.93 ± 0.05	3.38	6.23	7	-m	
**16**	5.69 ± 0.08	0.05	6.49	5.1	-m	
	(5.40 ± 0.05; 6.75 ± 0.06)	(1.0; 18.3)	(0.84)^e^			
**3a**	7.18 ± 0.13	37.6	3.73	5.9	0.58	>100
	(6.61 ± 0.02; 7.68 ± 0.05)	(14.0; 71.0)	(2.76)^e^			
**3b**	(3.07 ± 0.09; 6.59 ± 0.05)	(0.0; 13.4)	3.12	1.8	-l	>100
**3c**	(5.27 ± 0.04; 6.50 ± 0.04)	(0.7; 11.2)	2.21	2.3	-l	>100
			(2.57)^e^			
**3d**	5.90 ± 0.14	3.1	4.77	5.2	>100^j^	>100
	(5.45 ± 0.04; 6.82 ± 0.04)	(1.1; 20.8)	(3.94)^e^			
**3e**	n	n	4.77	1.6	>100^j^	>100
**3f**	(5.99 ± 0.03; 6.81 ± 0.03)	(3.7; 20.4)	4.77	5.1	>100^j^	>100
			(3.67)^e^			
**3g**	6.46 ± 0.02	10.3	4.77	8.9	>100^j^	>100
**3h**	4.76 ± 0.24	0.2	4.74	6.1	-l	>100
	(4.19 ± 0.01; 7.66 ± 0.01)	(0.0; 64.5)	(4.55)^e^			
**3i**	(5.71 ± 0.02; 7.09 ± 0.03)	(2.0; 32.8)	4.01	nt	nt	nt
			(3.39)^e^			
**3j**	(5.70 ± 0.01; 6.91 ± 0.03)	(2.0; 24.4)	4.01	nt	nt	nt
			(3.45)^e^			
**3k**	(6.00 ± 0.02; 7.15 ± 0.03)	(3.8; 36.2)	4.01	nt	nt	nt
			–3.74			
**3l**	(5.97 ± 0.01; 6.91 ± 0.04)	(3.6; 24.4)	4.01	nt	nt	nt
			(3.44)^e^			
**18**	6.70 ± 0.05	16.6	5.15	8	23.6^k^	>100
	(6.58; 7.65)	(13.1; 63.8)	(2.75)^e^			
**19**	(7.71 ± 0.08; 8.44 ± 0.01)	(67.1; 91.6)	(3.25)^e^	nt	nt	nt
**23**	4.23 ± 0.10; 6.91 ± 0.12	0.1; 24.5	4.39	11	9.82^k^	13

a Experimental p*K*_a_ values measured by UV-spectrophotometry using the 96-well plate method (H_2_O, 25 °C);^52^ only one (average) p*K*_a_ value could be measured for both heterocyclic rings using this technique.

b UV-metric titration using the SIRIUS T3 apparatus (p*K*_a_ values indicated between brackets) allowed to distinguish two p*K*_a_ values corresponding to the imino N of each side of the molecule.

c % ionization = 100 × [10^(pH–p*K*a)^]/[10^(pH–p*K*a)^ + 1] for p*K*_a_ > pH; % ionization = 100 × [10^(p*K*a–pH)^]/[10^(p*K*a–pH)^ + 1] for p*K*_a_ < pH.

d Calculated using MarvinSketch (ChemAxon) Version 23.4.

e Measured in octanol/water using the SIRIUS T3 apparatus.

f The increment in DNA thermal melting (Δ*T*_m_, °C) was measured with the oligonucleotide hairpin CGATATATATCGTCTCCGATATATATCG [(AT_4_)]. The melting temperature of (AT)_4_ DNA in sodium phosphate buffer (10 mM) was 43.6 ± 0.7 °C.

g Primary binding constant for fitting to a two-site binding model.

h DNA hairpins used in the SPR experiments (the loop is underlined): 5′-biotin-CGAATTCGTCTCCGAATTCG-3′ [A_2_T_2_]. 5′-biotin-CGCGCGCGTTTTCGCGCGCG-3′ [(CG)_4_].

i Taken from ref (42).

j Unspecific binding.

k Fitting to a one-site binding model.

l No binding at low concentration and unspecific binding to the chip dextran matrix at high concentration.

m Aggregate or precipitate.

n Not measured due to the lack of sample.

o p*K*_a_ value corresponding to the pyridine N.

p p*K*_a_ value corresponding to the heterocyclic NH.

Bis(2-aminobenzimidazoles) **2a**–**g**, **12**, **13**, and **16** showed Δ*T*_m_ values in the range 4.0–7.0 °C, indicating that this series also binds to the (AT)_4_-containing hairpin DNA (Table 4). With the exception of **2a** and **2d**, the binding affinities of the bis(2-aminobenzimidazoles) were lower than those of their bis(2-aminoimidazoline) counterparts, which may be related to the mostly uncharged nature of the benzimidazole series (p*K*_a_< 6.75) at physiological pH (Table 4). Indeed, positive charge(s) and a crescent-shaped molecule are known to promote kDNA minor groove binding.^14,41^ The replacement of the amide bond linker of **2a**–**g** by a more flexible ethylene chain (**12**) produced a *T*_m_ increase of 1.2 °C with respect to **2a** (Δ*T*_m_ = 4.9 °C). In contrast, replacing the *N*-phenylbenzamide skeleton with a rigid and planar fluorene scaffold (**16**) resulted in an insignificant (within the experimental error) increase in *T*_m_ versus **2a**.

The *T*_m_ increases for **3a**–**h** were in the range 1.6 °C (**3e**) to 11 °C (**23**), confirming that all the synthesized bis(arylimidamides) bind to AT-containing DNA, albeit with various affinities (Table 4). Most of the compounds except **3e** showed larger *T*_m_ increases than the bis(2-aminoimidazoline) lead **1a** upon binding to the (AT)_4_ DNA hairpin. According to these experiments, the strongest binder was the rigid fused-ring tricyclic fluorene derivative **23** (Δ*T*_m_ = 11.0 °C). This data agreed with the value reported by Boykin and co-workers for **23** complexed with poly(d(A-T)_2_) (Δ*T*_m_ = 15.2 °C).^25^ The chlorinated derivatives **3d**, **3f**, and **3g** also showed good affinity with Δ*T*_m_ values in the range 5.1–8.9 °C. In general, except for **3g** (R_2_/R_4_ = Cl), which displayed one of the strongest binding affinities (Δ*T*_m_ = 8.9 °C) of the series, the introduction of chlorine (**3d**–**f**) or pyridine rings into the scaffold (**3b** and **3c**) led to a drop in binding affinity with respect to the unsubstituted compound **3a**.

#### 2.2.6 Surface Plasmon Resonance-Biosensor Assays

**Figure 1:**
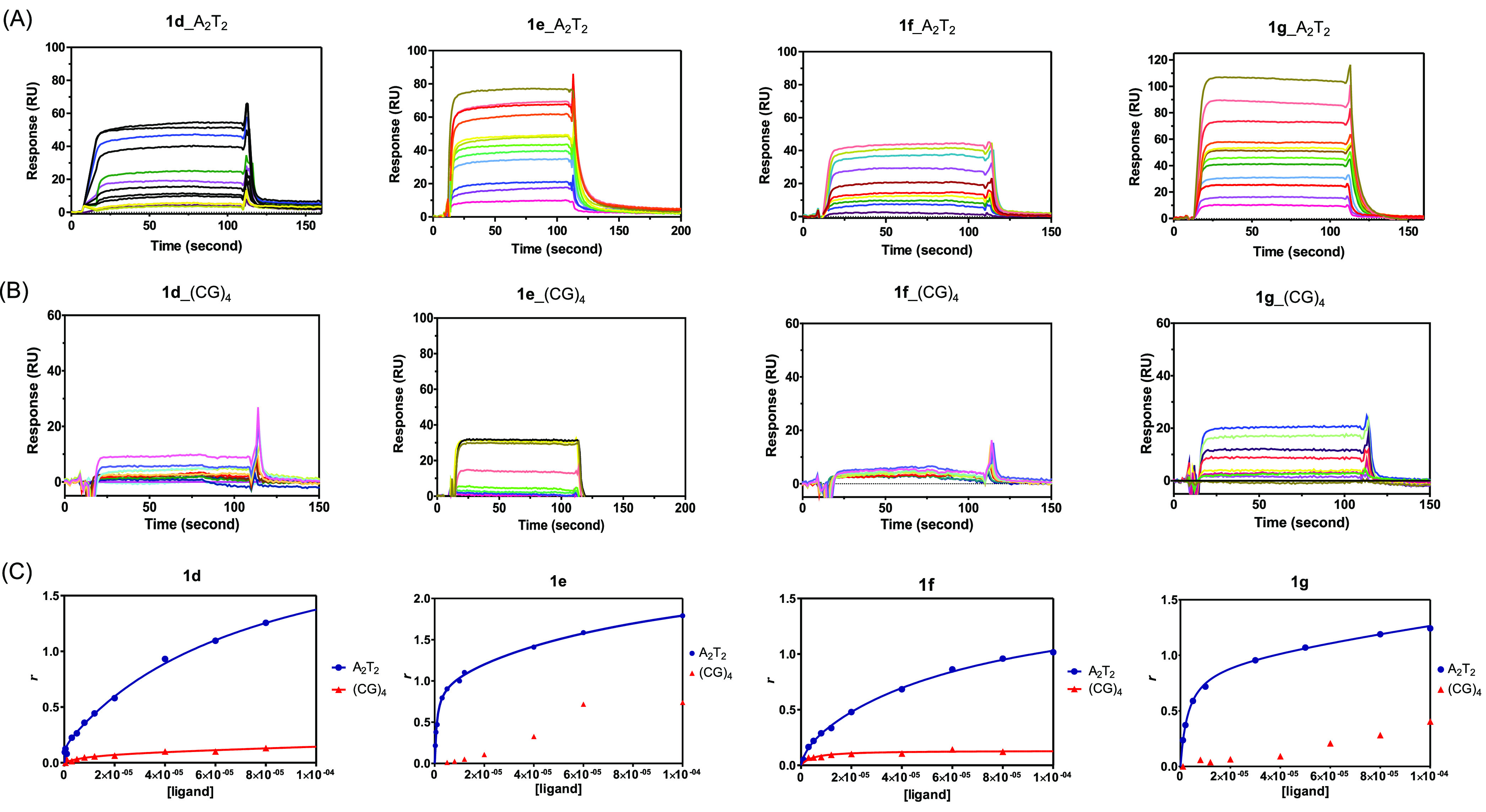
SPR binding affinity of bis(2-aminoimidazolines) **1d**–**g**. Sensorgrams for binding of chloro analogues **1d**–**g** to (A) A_2_T_2_ and (B) (CG)_4_ hairpin duplexes using increasing concentrations of the ligand in the range: 0.25–80 μM (**1d**, **1e**), 1–100 μM (**1f**), or 1–600 μM (**1g**) (from bottom to top). (C) SPR binding plots of **1d**–**g** for A_2_T_2_ and (CG)_4_ hairpins. The SPR response (RU) at equilibrium in the sensorgrams was converted to r (moles of bound compound per mole of DNA hairpin duplex; r = RU/RU_max_) and plotted against the free compound concentration, *C*_f_, flowing on the chip surface. The binding constants were determined by fitting the values to single-site or two-site binding models according to eq 1 (see Experimental Part).

Surface plasmon resonance (SPR) was used to measure the binding affinities of the compounds to two DNA hairpin duplexes containing CGAATTCG [“A_2_T_2_”] and CGCGCGCG [“(CG)_4_”] sequences.^42^ All of the *N*-phenylbenzamide-based bis(2-aminoimidazoline) derivatives (**1c**–**i**) bound to the A_2_T_2_ sequence with *K*_D_ values in the submicromolar to the micromolar range (Table 4, Figures 1 and S1), in agreement with the *T*_m_ experiments and with previous results obtained with similar compounds.^19,42^ The stoichiometry of binding was 1:1 for compounds **1c**, **1f**, **1h,** and **1i**, and >1:1 for **1d**, **1e**, and **1g,** as shown by *r* values (mole of bound compound per mole of DNA hairpin duplex) ≥ 1, respectively (Figures 1 and S1). Bis(2-aminoimidazoline) analogues with substituents on the *N*-phenylbenzamide rings were >2-fold weaker binders than **1a** (*K*_D_ = 0.17 × 10^–6^ M),^42^ as shown by primary binding constants (i.e., high affinity site) ranging from 0.38 × 10^–6^ M for **1c** (X/Y = N) to 45 × 10^–6^ M for the dichloro analogue **1d** (R_1_/R_3_ = Cl) (Table 4). Secondary binding constants were 80- to 30-times weaker, respectively. In these experiments, secondary binding generally accounts for nonspecific binding interactions with the DNA hairpin loop, as previously reported.^19^

The relative position and size of the substituents (i.e., Cl, O^*i*^Pr, and F) had a clear influence on the binding strength to the minor groove of A_2_T_2_-DNA. The compounds with chlorine (**1d**, **1f**, and **1g)** or O^*i*^Pr (**1h**) groups in positions *ortho* to the amide bond (R_2_/R_3_) had 4- to >40-times weaker binding affinity than the molecules with the Cl atoms next to the 2-aminoimidazoline group (**1e**, R_1_/R_4_ = Cl). This could be due to the conformational restriction and/or steric clash imposed by the presence of these groups near the amide bridge of the molecule. The fact that the fluorine atom *ortho* to the amide bond (**1i**, R_3_ = F) hardly affected the binding to DNA (*K*_D_ = 0.55 μM) compared to **1a** is consistent with the hypothesis that small size groups are preferred in this position. These compounds were AT-specific, as no significant binding to (CG)_4_ was observed up to 100 μM (Figure 1 and S1; Table 4).

For the bis(2-aminobenzimidazole) derivatives (**2a**–**g**, **12**, **13**, and **16**) no binding to A_2_T_2_ or (CG)_4_ sequences was observed at the highest concentration tested (100 μM). Unspecific binding to the chip dextran matrix (compounds **2a**–**g**) or aggregation/precipitation (**12**, **13**, and **16**) was observed instead, indicating that SPR-biosensor assays are not adequate to determine the binding affinity of this class of poorly water-soluble compounds.

Bis(arylimidamides) **3a** and **18** (amide and ethylene linkers, respectively) bound selectively to A_2_T_2_-containing DNA versus CG-containing sites with primary binding constants in the submicromolar range comparable to those of lead compound **1a** (Table 4). For compound **18**, this represents a 10-fold increase in affinity compared with its bis(2-aminoimidazoline) analogue reported earlier.^42^ In contrast, the fluorene derivative **23** showed little sequence selectivity with similar binding constants for A_2_T_2_ and (CG)_4_ oligonucleotides, which is consistent with a mixed intercalative/groove binding mode of interaction.^43^ The binding stoichiometry for compounds **3a**, **18**, and **23** was 2 mol of bound compound per mole of AT-containing DNA hairpin duplex (Figure S2), which is similar to lead **1a** and other related bis(imidazolin-2-imine) derivatives.^19,42,44^ From these SPR experiments, we did not detect significant binding to A_2_T_2_ and (CG)_4_-containing sequences at the highest concentration tested (100 μM) for the chlorine-containing compounds **3d**–**g**, whereas **3b**, **3c**, and **3h** showed nonspecific binding to the chip dextran matrix. This apparent discrepancy with the thermal melting assays, which showed binding of **3d**–**g** to (AT)_4_ DNA (vide supra) might indicate strong sequence selectivity for these derivatives. The A_2_T_2_ sequence used in the SPR assays has a very narrow minor groove of 3.5–4.0 Å in the center of the sequence (vs 5.16–6.79 Å for (AT)_4_),^45^ which tends to accommodate a more planar conformation of the bound ligand and favors van der Waals interactions with the walls of the groove.^42^ Hence, the presence of large chlorine substituents may be unfavorable for such interactions with this minor groove.

#### 2.2.7 DNA Binding Mode

**Figure 2:**
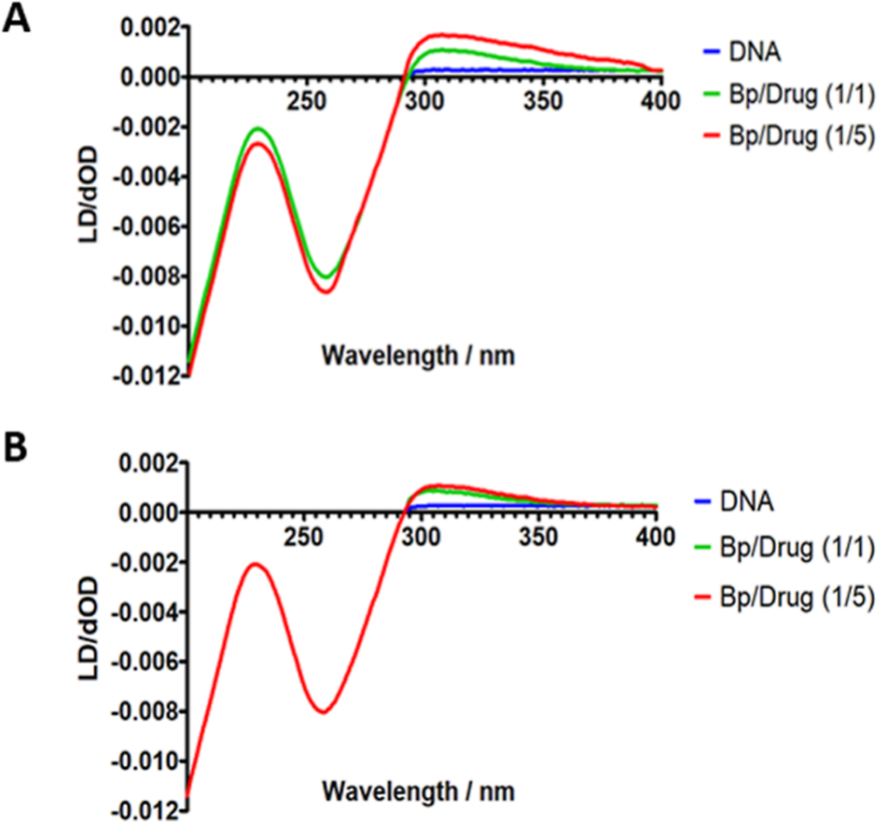
LD spectra for natural DNA (378.8 μM) titrated with **3a** (A) and **1g** (B) using a Bp/D ratio of 0/1, 1/1, and 1/5 in phosphate buffer at 25 °C. A positive induced LD signal indicative of groove binding is observed at 310 nm.

Once the DNA binding affinity and AT-selectivity of the compounds were established, we explored the binding modes of series **1** and **3** using flow linear dichroism (LD).^46^ Positive-induced LD signals are indicative of minor groove binding, whereas intercalating molecules induce a negative LD signal. LD spectra were recorded for natural DNA (salmon testes) titrated with representative compounds **1g** and **3a** in phosphate buffer at 25 °C working with a base pair/drug (Bp/D) ratio of 0/1, 1/1, and 1/5.^47^ A positive-induced LD signal was observed at ≈310 nm (where the DNA does not absorb) upon addition of increasing concentrations of **1g** and **3a** (Figure 2). Altogether, the results of LD, SPR, and CD experiments indicate that **1g** and **3a** bind specifically to the minor groove of AT-containing DNA, like lead **1a**.

#### 2.2.8 Fluorescent Intercalator Displacement (FID) Assay

We sought to determine whether the bis(2-aminobenzimidazole) series **2** compounds could bind DNA by intercalation. A FID assay utilizing the displacement of the DNA-bound fluorescent intercalator ethidium bromide was accomplished with compounds **2a**, **2c**, **2d**, **12**, **13**, **16**, and **3a** as controls (see Supporting Information).^48^ The addition of compounds **2a**, **2c**, and **12** to (AT)_4_ and (CG)_4_ hairpins resulted in >50 and >87% decreases in fluorescence, respectively, indicating strong intercalation (Table S2B). Binding of **2d**, **13**, and **16** to (AT)_4_ and (CG)_4_ reduced the fluorescence (*F*) by 25 and ≥43%, respectively, indicating that the compounds intercalate, although to a lower extent. In contrast, the addition of **3a** to (AT)_4_ and (CG)_4_ did not decrease the % *F*, consistent with its specific minor groove mode of binding.

#### 2.2.9 Determination of Physicochemical Parameters (p*K*_a_, log *P*, and Solubility)

The protonation state of drugs in the body has a profound influence on their ADMET properties and their binding to biological targets.^49^ For DNA MGBs, the (di)cationic nature of the ligand, in addition to a crescent shape matching the curve of the groove, is crucial to allowing the best fitting between the MGB and the groove through van der Waals and hydrogen bonding interactions.^50^ Therefore, the p*K*_a_ values of the compounds were measured by UV spectrophotometry using the 96-well plate methodology developed in our group.^51,52^ This allowed the determination of the average p*K*_a_ value of these dibasic compounds (Table 4). For several compounds, the p*K*_a_ was also determined with the Sirius T3 apparatus, which allowed distinguishing the p*K*_a_ values of both heterocyclic moieties within the same molecule (i.e., pyridine-2-carboxamidine, 2-aminoimidazoline, and 2-aminobenzimidazole) (Table 4).

In contrast to the predicted values (6.54–7.70, calculated with the Chemicalize ChemAxon LLC software), the experimental p*K*_a_ (H_2_O, 25 °C) of the bisbenzimidazole derivatives **2a**–**g** was in the range 3.38–6.75 (i.e., % ionization at pH 7.4 ranging from 0.01 to 18.3), indicating that the compounds are mostly neutral at physiological pH (Table 4). These measured p*K*_a_ values were much lower (i.e., approximately 1.5–2 p*K*_a_ units) than the predicted ones, highlighting the limits of p*K*_a_ prediction tools for compounds containing a large number of possible tautomeric states.^53,54^ In contrast, the bis(2-aminoimidazolines) **1a**–**i** are mostly dicationic, with experimental p*K*_a_ values in the range 8.05–10.26 (i.e., > 85.5% ionized) that is approximately >1 p*K*_a_ unit higher than the predicted ones. Replacement of the 2-aminoimidazoline heterocycles by pyridine-2-carboxamidine substituents led to a reduction of the p*K*_a_ of the molecules by >2 p*K*_a_ units, giving mean values in the range 4.19 (**3h**)–8.44 (**19**). Accordingly, the derivatives showed disparate percentages of ionization at pH 7.4 ranging from 0.2% (**3h**) up to 71% (**3a**) and 91.6% (**19**). The p*K*_a_ SAR for these series followed the trends of previous series of bis(2-aminoimidazolines) containing similar scaffolds.^20,51,52,54^ Interestingly, a positive correlation between the high p*K*_a_ values of each compound and Δ*T*_m_ was observed for series 2 (**2c**, **2e**, **12**, **13**, and **16**) and 3 (**3a**–**d**, **3f**, **3h**, and **18**) (Figure S3; *r*^2^ = 0.76), indicating that an increased percentage of ionization favored binding to DNA. This is consistent with the well-established central role played by the positively charged ends of DNA MGBs in enhancing electrostatic interactions with DNA.^55^ The absence of a similar correlation with series **1** is probably due to the high basicity of the bis(2-aminoimidazoline) compounds, which are almost totally dicationic at pH 7.4.

The log *P* of 11 representative compounds from the different series was measured, and the calculated values were also determined for all the compounds. Other physicochemical parameters are listed in Table S2. All of the new compounds display higher log *P* values than lead **1a**, with most of them being <5. It is noteworthy that calculations tend to overestimate log *P* values from 0.3–0.4 (series **1**) or 0.2–2.4 (series **3**) up to 3.6 for series **2** (Table 4). Based on these estimated values, compounds **2e** (6.86), **12** (7.24), **16** (6.49), or **18** (5.15) would break the “rule of 5” (i.e., log *P* < 5) for this parameter, whereas the experimental log *P* is much lower than predicted (3.21, 4.32, 0.84, and 2.75, respectively). This is especially relevant for series **2**, whose most predicted values were >5. Of note, there was no apparent correlation between the activity against *T. brucei* and the experimental p*K*_a_ or log *P* of the molecules.

The aqueous kinetic solubility of compounds **3a**, **3d**, and **18** was determined by UV spectroscopy in 96 multiwell plates (Table 5). As expected, the solubility of these basic compounds increased substantially (2-, 16-, and 7-fold) at low pH (1.2). Altogether, these compounds fulfill the R05 for oral bioavailability and are potential candidates for in vivo studies.

**Table 5:** Solubility and Physicochemical Parameters of Selected Compounds

cmpd	MW	HBD	HBA	log *P*^a^	“Ro5”^b^	kinetic solubility^c^ (μM)		
						pH 1.2	pH 5.5	pH 7.4
**3a**	435	5	7	2.76	+	107.3 ± 10.7	107.8 ± 10.8	6.35 ± 0.63
**3d**	504	5	7	3.94	+	104.9 ± 10.5	nd	46.3 ± 4.6
**18**	420	4	6	2.75	+	101.6 ± 0.2	nd	9.95 ± 0.51

a Measured in octanol/water using the SIRIUS T3 apparatus.

b Compounds that fulfill Lipinski’s “rule of 5” for oral bioavailability are marked +.

c At 25 °C in buffer with 1% DMSO after 2h stirring.

#### 2.2.10 Microsomal and Plasma Stability

The microsomal stability toward metabolism by cytochrome P450 (Phase-I metabolism) and uridine glucuronosyl-transferase (UGT) (Phase-II metabolism) of the selected hit compound **3a** was studied in the presence of NADPH and UDPGA. Compound **3a** was apparently not metabolized by human and mouse (CD-1) liver microsomes, as we did not observe the formation of any significant metabolite during the course of the reaction, i.e., up to 120 min, indicating a half-life >2 h. In comparison, human liver microsomes rapidly metabolized (high intrinsic clearance) diclofenac under the same conditions with a half-life <30 min (Table S1). Compound **3a** was not apparently modified in human serum, and it was stable for 1 h of incubation. Thus, under the experimental conditions used in this study, compound **3a** is metabolically stable in human and mouse microsomal fractions and in human serum.

## 3 Discussion

AT-specific DNA MGBs have a record of success as chemotherapeutic agents against human and animal infections caused by trypanosomatid parasites.^41,56,57^ In particular, the bis(2-aminoimidazoline) class of DNA MGBs, such as lead **1a**, are very effective in vitro and in vivo against *T. brucei*.^14,18,28^ However, like diamidines,^58,59^ these compounds are much less active against the intracellular parasite *Leishmania*. The high polarity (i.e., dicationic nature) of the compounds is probably not the sole reason for reduced cidal activity against intracellular parasites. In fact, *Leishmania* does not express particularly good transporters for diamidines, including pentamidine,^60,61^ but expression of *T. brucei* AQP2 in *Leishmania* renders them just as susceptible to pentamidine as trypanosomes.^62^ The same hypersensitization occurs with expression of TbAQP2 or TbAT1 in *Trypanosoma congolense*, which are otherwise relatively insensitive to diamidines.^63^ Hence, the different parasites express transporters with different substrate preferences and affinities, which will influence the SAR from cell to cell.

In this work, we studied the effect of scaffold modifications that lower the basicity and/or increase the lipophilicity of **1a** on the antiparasitic activity and DNA binding properties. For the bis(2-aminoimidazolines) **1c**–**i**, which displayed EC_50_ values in the micromolar range against *T. brucei* but were mostly inactive against *T. cruzi* and *Leishmania* (except **1h**), the introduction of Cl, O^*i*^Pr, pyridine rings, 1,2-diphenylethane,^18^ or 1,3-diphenylurea^18^ did not improve the antikinetoplastid activity versus **1a**. From this series, the dichloro derivative **1e**, which was the most active and selective compound against both WT and pentamidine-resistant (B48) *T. brucei* strains, was approximately 7-times less potent than **1a** against this parasite. In previous studies, we observed that the introduction of one chlorine atom in position *ortho* to the imidazoline rings (R_4_ = Cl) increased the anti-*T. brucei* activity by nearly 4-fold.^20^ Hence, the presence of two halogen atoms in the bis(2-aminoimidazoline) scaffold appears to be counterproductive for antiprotozoal activity.

In contrast, the same scaffold modifications for the benzimidazole series **2** (**2c**–**g**, **12**, **13**, and **16)**, led to an up to 50-fold increase in anti-*T. brucei* activity with respect to the unmodified *N*-phenylbenzamide analogue **2a**. The replacement of the 2-aminoimidazoline groups by 2-aminobenzimidazole heterocycles afforded compounds with the same hydrogen bond-forming capacity as series **1**, but with much lower p*K*_a_ values (3.38–6.75) and higher lipophilicity (calcd. log *P* = 4.30–7.24). However, these structural modifications did not improve the in vitro antiparasitic activity against extracellular (*T. brucei*) or intracellular (*T. cruzi, Leishmania*) kinetoplastid parasites with respect to lead **1a** or the monochlorinated analogues reported earlier.^20^ The moderate selectivity indexes and the nondifferentiation against cells lacking the high affinity transporters TbAT1 and TbAQP2 for diamidines,^61,64^ melaminophenyl arsenicals, and other trypanocidal drugs suggest a lack of high affinity carriers for this class of molecules in *T. cruzi* and *Leishmania* spp., consistent with earlier reports.^61^ The absence of specific protozoal transporters is exacerbated by the lack of a strong accumulative driving force for the neutral bis(2-aminobenzimidazole) compounds, potentially impacting the translocation across both plasma membrane and mitochondrial membranes. A large body of evidence suggests that pentamidine and other dicationic MGBs derive their antiprotozoal selectivity from selective uptake by the parasites.^29,32,62,63,65^ This concerns probably mostly uptake over the plasma membrane, but accumulation of dicationic drugs in kinetoplastid parasites is ultimately driven by the mitochondrial membrane potential, driving a buildup of the dications in the mitochondria,^61,66–68^ where MGBs interfere with the functioning and replication of the kinetoplast.^69–71^ On the other hand, the neutral compounds might be expected to remain cytosolic rather than accumulate disproportionally in the mitochondria and therefore act on AT-rich nuclear rather than kinetoplast DNA, albeit at a much lower level of accumulation. This dynamic of mitochondrial segregation of cationic MGBs and low accumulation of neutral MGBs is certainly part of the explanation for the lower toxicity of such compounds against mammalian cells, in addition to the relatively inefficient uptake by organic cation transporters with OCT1–3 displaying *K*_m_ values between 10 and 20 μM for pentamidine^64^ compared to 35 nM for TbAQP2.^30^ Nothing is currently known about transporters for diamidines or other MGBs in *Trichomonas* spp.

Interestingly, the main SAR results (e.g., effect of Cl atoms) seemed to apply to both series **1** and **2**. However, none of the series **2** compounds showed activity against *T. cruzi* or *L. donovani*, except **12**, which had marginal activity against promastigotes of *L. donovani* (EC_50_ = 8.7 μM), albeit with low selectivity (SI_(THP-1/promast.)_ = 1.6). As a whole, the chemical modification 2-aminoimidazoline ↔ 2-aminobenzimidazole provided no improvement in antiprotozoal activity or DNA binding affinity, which is probably related to the low p*K*_a_ of the bis(2-aminobenzimidazoles).

Consistent with earlier results from Boykin and co-workers showing that the bis(arylimidamide) analogues of furamidine-based diamidines are especially active against the intracellular parasites *Leishmania* and *T. cruzi*,^72–77^ series **3** analogues were very potent inhibitors of *T. cruzi* and *Leishmania*, but also of *T. brucei* growth, with adequate selectivity. In particular, **3a**, **3c**–**e**, **18**, and **23**, which display submicromolar EC_50_ values and SI_(THP-1/amast.)_ > 20 against intracellular amastigotes of *L. donovani* in the same range as amphotericin B and present druglike properties, can be considered as new antileishmanial hit compounds. These values are in the same range as furamidine’s bis(arylimidamide) derivative DB766^73^ (i.e., *N*,*N*″-(furan-2,5-diylbis(3-isopropoxy-4,1-phenylene))dipicolinimidamide), and superior to analogues modified on the terminal group.^72^

Regarding the SAR of this series, the presence of nitrogen atoms in the central scaffold had diverse effects on the activity. The introduction of two pyridine rings (**3c**) increased the antileishmanial activity very efficiently, especially against amastigote forms (EC_50_ = 0.55 μM). In contrast, the closely related *N*-(pyridin-2-yl)benzamide analogue (**3b**), which was almost as active as **3c** against promastigotes (EC_50_ = 1.46 μM), was ineffective against intracellular amastigotes of *L. donovani.* Since **3b** and **3c** have similar DNA binding affinities (Δ*T*_m_ = 1.8 and 2.3 °C, respectively), this difference is probably related to dissimilar uptake of the compounds resulting from different membrane permeabilities or drug transport. The introduction of two chlorine atoms adjacent to the arylimidamide group (**3d**, **3e**) or O^*i*^Pr groups (**3g**) on the *N*-phenylbenzamide scaffold also appeared as a good strategy to improve the activity against intracellular amastigotes of *T. cruzi* and, to a lesser extent, *L. donovani.* Of note, the positive effect of large *O*-alkyl groups was consistent with the SAR reported for furamidine-based bis(arylimidamides) series (e.g., DB766) despite being based on quite different central scaffolds.^73,78,79^ The 1,2-diphenylethane (**18**) is another interesting scaffold that displayed excellent activities and selectivity against intracellular amastigotes of *L. donovani*. The latter also worked as an *N*-phenylbenzamide surrogate for the bis(2-aminoimidazoline) series active against *T. brucei*.^15,18^

All of the compounds were also tested against the protozoan parasite *T. vaginalis* because its genome has a high content of AT base pairs, including an unusually high number of genomic repeats,^80^ which may potentially be targeted by DNA MGBs. In previous studies, Crowell et al. showed that dicationic DNA MGBs at AT sites, such as aromatic diamidines, have potential as antitrichomonal agents with activities in the micromolar range.^81^ Among the compounds studied here, only seven showed a weak trichomonacidal effect (EC_50_ = 11.5–32.5 μM) displaying a cytotoxic effect on mammalian cells, with the exception of **3l**. We do not have an evidence-based explanation for the lack of trichomonacidal activity of these compounds, which are AT-specific DNA MGBs, but this is likely to be related to a poor uptake of these dicationic compounds in *Trichomonas*, as is the case for *T. congolense*, which is much less susceptible to diamidines than *T. brucei*.^63^

In the present study, we did not observe a correlation between the antiparasite activity of the new compounds and the DNA binding affinity (Δ*T*_m_). On the one hand, such a correlation between the cellular activity and the binding affinity for the target depends on several factors (e.g., cell uptake differences, intracellular distribution, and distinct cellular targets) and is not always easy to demonstrate.^69,82^ On the other hand, we were able to confirm that kDNA is the cellular target of lead compound **1a** in *T. brucei* using a combination of flow cytometry, imaging techniques, and biophysical experiments.^19^ Notwithstanding, in the present work, we showed that the three series of compounds stabilize AT-rich DNA duplexes upon binding, with affinity constants to A_2_T_2_ in the submicromolar to micromolar range similar to previously reported bis(2-aminoimidazolines)^19,42^ and diamidines (e.g., pentamidine, DB829).^41^ Most of series **1–3** compounds hold the basic structural features of MGBs, including a crescent-shaped *N*-phenylbenzamide scaffold and positive charge(s) that can promote binding to kDNA for series **1** (p*K*_a_ = 8.05–10.26) and **3** (p*K*_a_ = 6.46–8.44), contrary to series **2** compounds (p*K*_a_ = 3.38–6.75), which are mostly neutral at physiological pH. Linear dichroism experiments with selected molecules from series **1** and **3** (**1g**, **3a**) confirmed unequivocally that both compounds are minor groove binders like lead **1a**. FID assays ruled out an intercalative mode of binding for **3a**. These results were consistent with the SPR data showing AT base-pair specificity versus CG and, hence, a minor groove preference for these ligands.^14,19,42,47,83–87^ In contrast, the fluorene derivative **23**, which bound to AT and GC-containing oligonucleotides with similar *K*_D_ values (9.8 and 13 μM, respectively), displayed no base-pair sequence preference, which is consistent with previous findings reporting both intercalation and minor groove binding for the bis(2-aminoimidazoline) analogue of **23**.^43^ According to the FID assay, bis(2-aminobenzimidazole) compounds **2a**, **2c**, **2d**, **12**, **13**, and **16** appeared to have an intercalative mode of binding, although a mixed “groove binding/intercalation” mode cannot be ruled out.

Despite their strong binding affinity and selectivity for AT-rich DNA, the compounds reported here may have additional cellular target(s) besides kDNA. This is the case, for instance, for the dicationic DNA MGB pentamidine, whose effects against the nonkinetoplastid pathogens *Plasmodium falciparum* and *Pneumocystis carinii* point toward other cellular targets in those organisms,^88^ including RNA splicing in *P. carinii*(89) and binding to ferriprotoporphyrin IX in *P. falciparum*.^90^ For some compounds, the level of human cell line toxicity supports this hypothesis. Daliry et al. showed that the trypanocidal activity of bis(arylimidamides) related to DB766 did not correlate with their binding affinity to *T. cruzi* kinetoplast DNA. They observed that a strong affinity with kDNA per se was not sufficient to trigger the trypanocidal activity of the studied diamidines and speculated that “cell uptake differences and possibly distinct cellular targets need to be considered”.^91^ As far as bis(arylimidamides) are concerned, the recently discovered cytochrome P450, CYP5122A1 (sterol 14-demethylase, an antifungal azole target), which is essential for *L. donovani* survival,^92^ could also be involved in their mode of action, as proposed by Werbovetz and co-workers.^93^

## 4 Conclusions

Efforts to improve the activity of the DNA MGB lead compound **1a** against intracellular kinetoplastid parasites were successful with the bis(arylimidamide) series **3**. The bis(pyridine-2-carboxamidine) **3a** was definitively the best in class, showing excellent activity and selectivity against *T. brucei*, *T. cruzi*, and *L. donovani*, as well as druglike properties. Hence, compound **3a** is a candidate for further in vivo studies. Nonetheless, other compounds from this series were also highly efficacious in terms of activity and selectivity against intracellular amastigotes of *T. cruzi* (**3a**, **3d**, **3g**, and **23**) and *L. donovani* (**3c**–**e**, **3g**, **18**, and **23**).

We showed that compounds from series **1** and **3** are DNA MGBs, whereas compounds from series **2** appear to intercalate, even though a mixed mode of binding cannot be ruled out. Therefore, kDNA is a probable target of the new compounds reported here, although their exact MoA remains to be determined. The correlation observed between the p*K*_a_ and the DNA binding affinities (Δ*T*_m_) for series **2** and **3** showed that the ionization state of these molecules is a driving force that influences both the uptake into the parasites and the extent and mode of binding to DNA.

## 5 Experimental Part

### 5.1 Chemistry

All the commercial chemicals were obtained from Sigma-Aldrich, Fluorochem, Acros Organics, or Alfa Aesar and were used without further purification. Deuterated solvents for NMR use were purchased from Merck (Sigma-Aldrich). Dry solvents were either obtained from Acros Organics and Sigma-Aldrich in SureSeal bottles or were distilled using standard procedures, according to Vogel’s Textbook of Practical Organic Chemistry. Solvents for synthesis purposes were used at GPR grade. Reactions heated by microwaves were realized in a Biotage Initiator microwave oven reactor (frequency: 2045 GHz). Chromatographic columns were run using Silica gel 60 (230–400 mesh ASTM) or Aluminum Oxide (activated, Neutral Brockman I STD grade 150 mesh). Analytical TLC was performed using Merck Kieselgel 60 F_254_ silica gel plates or Alugram© Alox N/UV_254_ aluminum oxide plates. TLC plates were visualized under UV light (254 and 365 nm) and/or revealed with staining reagents (i.e., iodine, phosphomolybdic acid, and ninhydrin). Flash chromatography was performed in an Isolera One (Biotage) with Isolera 3.3.0 version, using Biotage Sfär columns (silica D, duo 60 μm) or SiliaSep Flash cartridges (SiliCycle) 40–63 μm, 60 Å. Reverse-phase chromatography was performed using LiChroprep RP-18 (25–40 μm), Merck, and Claricep Screw-on Flash C18 columns (spherical, 20–35 μm, 100 Å, 122 g). Centrifugal TLC was carried out with the Chromatotron using circular glass plates prepared with silica gel 60 PF_254_ containing gypsum. ^1^H- and ^13^C NMR spectra were recorded on a Bruker Avance-300, a Bruker DPX-400 Advance spectrometer, and a Varian-500. Chemical shifts of the ^1^H NMR spectra were referenced to the residual peak of the deuterated solvent: CDCl_3_ (δ 7.26 ppm), methanol-*d*_4_ (δ 3.31 ppm), and DMSO-*d*_6_ (δ 2.5 ppm). Chemical shifts of the ^13^C NMR spectra were referenced to CDCl_3_ (δ 77.16 ppm), methanol-*d*_4_ (δ 49.0 ppm), and DMSO-*d*_6_ (δ 39.52 ppm). Signal multiplicity for ^1^H NMR is defined as singlet (s), doublet (d), triplet (t), quartet (q), multiplet (m), and br (broad signal). Coupling constants *J* are expressed in hertz (Hz). LC–MS spectra were recorded on a WATERS apparatus integrated with a HPLC separation module (2695), PDA detector (2996), and a Micromass ZQ spectrometer using electrospray ionization (ESI^+^). Analytical HPLC was performed with a SunFire C18–3.5 μm column (4.6 mm × 50 mm). Mobile phase A: CH_3_CN + 0.08% formic acid and B: H_2_O + 0.05% formic acid. UV detection was carried out over 190 to 440 nm. Melting points were determined using a Mettler Toledo MP70 digital melting point apparatus and are uncorrected. Some of them show a broad melting range due to the low degree of crystallinity. Elemental analysis was carried out at the Microanalysis Laboratory, “Manuel Lora Tamayo” Organic Chemistry Centre—CSIC. High-resolution mass spectra were recorded at the Elemental Microanalysis Unity at the Pharmacy College, Complutense University of Madrid. All of the biologically tested compounds were ≥95% pure by HPLC except **2d** (93%), **3b** (94%), **3b**_**b** (91%), **3g** (91%), and **20** (93%).

#### 5.1.1 Synthesis of Bis(imidazolidin-2-iminium) Salt Derivatives (**1c–i**)

##### 5.1.1.1 Method A

Trifluoroacetic acid (2 mL) was added to a cooled solution (ice–water bath) of the Boc-protected compound **6c**–**i** (1 mmol, 60–220 mg scale) dissolved in CH_2_Cl_2_ (2–6 mL). The resulting solution was stirred for 4 h at the same temperature. Trifluoroacetic acid and dichloromethane excesses were evaporated under vacuum. This process was repeated by dissolving the crude residue in CH_2_Cl_2_ and evaporating the solvents under a high vacuum. The sticky solid was crushed with diethyl ether to precipitate the product as a powder.

##### 5.1.1.2 5-(Imidazolidin-2-ylideneamino)-*N*-(5-(imidazolidin-2-ylideneamino)pyridin-2-yl)picolinamide Ditrifluoroacetate Salt (**1c**)

Compound **6c** (64 mg, 0.08 mmol) was reacted with TFA according to method A. **1c** was obtained as a whitish solid (16.7 mg, 35%). mp 214.0–226.8 °C. ^1^H NMR (400 MHz, DMSO-*d*_6_): δ 11.34 (br s, 1H), 10.72 (br s, 1H), 10.44 (s, 1H), 8.95 (br s, 2H), 8.66 (d, *J* = 2.6 Hz, 1H), 8.60 (br s, 2H), 8.34 (d, *J* = 2.5 Hz, 1H), 8.33 (d, *J* = 8.9 Hz, 1H), 8.26 (d, *J* = 8.5 Hz, 1H), 7.97 (dd, *J* = 8.5, 2.5 Hz, 1H), 7.85 (dd, *J* = 8.9, 2.6 Hz, 1H), 3.74 (s, 4H), 3.68 (s, 4H). ^13^C NMR (101 MHz, DMSO-*d*_6_): δ 161.5, 158.5, 157.8, 148.8, 145.1, 144.6, 142.9, 136.7, 135.1, 131.6, 129.1, 123.3, 113.6, 42.8, 42.7. HPLC (UV) > 95%. LRMS (ESI ^+^) *m*/*z*: 366.3 [M + H]. HRMS (ESI^+^) *m*/*z*: 366.1781 [M + H] (calcd for C_17_H_20_N_9_O, 366.1785).

##### 5.1.1.3 3-Chloro-*N*-(2-chloro-4-(imidazolidin-2-ylideneamino)phenyl)-4-(imidazolidin-2-ylideneamino)benzamide Ditrifluoroacetate Salt (**1d**)

Compound **6d** (64 mg, 0.08 mmol) was reacted with TFA according to method A. Compound **1d** was obtained as a whitish solid (14.5 mg, 28%). mp 222.4–223.8 °C. ^1^H NMR (400 MHz, DMSO-*d*_6_): δ 10.84 (br s, 2H), 10.37 (s, 1H), 8.67 (br s, 2H), 8.58 (br s, 2H), 8.22 (d, *J* = 2.0 Hz, 1H), 8.03 (dd, *J* = 8.3, 2.0 Hz, 1H), 7.66 (d, *J* = 8.3 Hz, 1H), 7.59 (d, *J* = 8.6 Hz, 1H), 7.49 (d, *J* = 2.4 Hz, 1H), 7.28 (dd, *J* = 8.6, 2.4 Hz, 1H), 3.70 (br s, 8H). ^13^C NMR (101 MHz, DMSO-*d*_6_): δ 163.5, 158.1, 157.8, 136.2, 135.1, 133.8, 132.5, 130.5, 129.9, 129.6, 129.5, 128.3, 128.0, 124.1, 122.3, 42.8, 42.7. HPLC (UV) > 95%. LRMS (ESI^+^) *m*/*z*: 432.4 [M + H]. HRMS (ESI^+^) *m*/*z*: 432.1102 [M + H] (calcd for C_19_H_20_Cl_2_N_7_O, 432.1101).

##### 5.1.1.4 3-Chloro-*N*-(3-chloro-4-(imidazolidin-2-ylideneamino)phenyl)-4-(imidazolidin-2-ylideneamino)benzamide Ditrifluoroacetate Salt (**1e**)

Compound **6e** (212 mg, 0.25 mmol) was reacted with TFA according to method A. Compound **1e** was obtained as a whitish powder (140 mg, 85%). mp > 114.0 °C. ^1^H NMR (500 MHz, DMSO-*d*_6_): δ 10.95 (br s, 1H), 10.75 (s, 1H), 10.59 (br s, 1H), 8.70 (br s, 2H), 8.50 (br s, 2H), 8.23 (d, *J* = 2.1 Hz, 1H), 8.14 (d, *J* = 2.4 Hz, 1H), 8.03 (dd, *J* = 8.3, 2.1 Hz, 1H), 7.82 (dd, *J* = 8.7, 2.4 Hz, 1H), 7.67 (d, *J* = 8.3 Hz, 1H), 7.48 (d, *J* = 8.7 Hz, 1H), 3.70 (s, 4H), 3.67 (s, 4H). ^13^C NMR (126 MHz, DMSO-*d*_6_): δ 163.6, 158.6, 158.1, 139.6, 136.1, 134.2, 130.6, 129.7, 129.4, 129.3, 128.1, 128.0, 127.9, 121.2, 119.9, 42.8, 42.7. HPLC (UV) > 95%. LRMS (ESI^+^) *m*/*z*: 432.3 [M + H]. HRMS (ESI^+^) *m*/*z*: 432.1093 [M + H] (calcd for C_19_H_20_Cl_2_N_7_O, 432.1101).

##### 5.1.1.5 2-Chloro-4-(imidazolidin-2-ylideneamino)-*N*-(2-chloro-4-(imidazolidin-2-ylideneamino)phenyl)-benzamide Ditrifluoroacetate Salt (**1f**)

Compound **6f** (98 mg, 0.12 mmol) was reacted with TFA according to method A. Compound **1f** was obtained as a whitish solid (76 mg, 60%). mp 196.5–207.3 °C. ^1^H NMR (500 MHz, DMSO-*d*_6_): δ 11.03 (br s, 1H), 10.76 (br s, 1H), 10.27 (s, 1H), 8.74 (br s, 2H), 8.60 (br s, 2H), 7.73 (d, *J* = 8.5 Hz, 1H), 7.68 (d, *J* = 8.3 Hz, 1H), 7.48 (d, *J* = 2.5 Hz, 1H), 7.47 (d, *J* = 2.2 Hz, 1H), 7.32 (dd, *J* = 8.3, 2.2 Hz, 1H), 7.27 (dd, *J* = 8.5, 2.5 Hz, 1H), 3.71 (s, 4H), 3.69 (s, 4H). ^13^C NMR (126 MHz, DMSO-*d*_6_): δ 165.2, 158.3, 158.0, 138.9, 135.0, 133.6, 132.9, 131.6, 130.7, 129.5, 128.8, 124.9, 124.0, 123.1, 121.7, 43.2, 43.1. HPLC (UV) > 95%. LRMS (ESI^+^) *m*/*z*: 432.3 [M + H]. HRMS (ESI^+^) *m*/*z*: 432.1106 [M + H] (calcd for C_19_H_20_Cl_2_N_7_O, 432.1101).

##### 5.1.1.6 2-Chloro-4-(imidazolidin-2-ylideneamino)-*N*-(3-chloro-4-(imidazolidin-2-ylideneamino)phenyl)-benzamide Ditrifluoroacetate Salt (**1g**)

Compound **6g** (159 mg, 0.2 mmol) was reacted with TFA according to method A, yielding compound **1g** as a whitish solid (102 mg, 81%). mp 190.2–201.7 °C. ^1^H NMR (500 MHz, DMSO-*d*_6_): δ 11.08 (s, 1H), 10.87 (s, 1H), 10.44 (s, 1H), 8.75 (s, 2H), 8.39 (s, 2H), 8.08 (d, *J* = 2.4 Hz, 1H), 7.66 (dd, *J* = 8.5, 2.4 Hz, 2H), 7.49 (d, *J* = 2.2 Hz, 1H), 7.47 (d, *J* = 8.5 Hz, 1H), 7.32 (dd, *J* = 8.5, 2.2 Hz, 1H), 3.71 (s, 4H), 3.66 (s, 4H). ^13^C NMR (126 MHz, DMSO-*d*_6_): δ 164.7, 158.6, 157.5, 139.5, 138.6, 133.3, 131.0, 130.1, 129.6, 127.9, 123.5, 121.3, 120.3, 119.2, 42.8, 42.7. HPLC (UV): 95%. LRMS (ESI^+^) *m*/*z*: 432.3 [M + H]. HRMS (ESI^+^) *m*/*z*: 432.1078 [M + H] (calcd for C_19_H_20_Cl_2_N_7_O, 432.1101).

##### 5.1.1.7 4-(Imidazolidin-2-ylideneamino)-*N*-(4-(imidazolidin-2-ylideneamino)-3-isopropoxyphenyl)-2-isopropoxybenzamide Ditrifluoroacetate Salt (**1h**)

Compound **6h** (114 mg, 0.13 mmol) was reacted with TFA according to method A. **1h** was obtained as a whitish solid (47 mg, 51%). mp.: > 201 °C. ^1^H NMR (500 MHz, DMSO-*d*_6_): δ 11.08 (s, 1H), 10.59 (s, 1H), 9.91 (s, 1H), 8.74 (s, 2H), 8.41 (s, 2H), 8.06 (d, *J* = 8.5 Hz, 1H), 7.12 (d, *J* = 2.0 Hz, 1H), 7.03 (d, *J* = 2.4 Hz, 1H), 6.99 (dd, *J* = 8.5, 2.0 Hz, 1H), 6.85 (dd, *J* = 8.6, 2.4 Hz, 1H), 4.88 (hept, *J* = 6.1 Hz, 1H), 4.74 (hept, *J* = 6.1 Hz, 1H), 3.72 (s, 4H), 3.66 (s, 4H), 1.44 (d, *J* = 6.1 Hz, 6H), 1.36 (d, *J* = 6.1 Hz, 6H). ^13^C NMR (101 MHz, DMSO-*d*_6_): δ 162.0, 158.2, 157.6, 156.3, 147.4, 140.8, 133.0, 131.6, 126.7, 121.4, 119.5, 116.0, 114.9, 109.7, 108.9, 73.2, 71.7, 42.7, 42.7, 22.0, 21.9. HPLC (UV) > 95%. LRMS (ESI^+^) *m*/*z*: 480.4 [M + H]. HRMS (ESI^+^) *m*/*z*: 480.2707 [M + H] (calcd for C_25_H_34_N_7_O_3_, 480.2718).

##### 5.1.1.8 3-Fluoro-*N*-(2-fluoro-4-(imidazolidin-2-ylideneamino)phenyl)-4-(imidazolidin-2-ylideneamino)benzamide Ditrifluoroacetate Salt (**1i**)

Compound **6i** (53 mg, 0.07 mmol) was reacted with TFA according to method A. Compound **1i** was obtained as a beige solid after several recrystallizations from ^*i*^PrOH/Et_2_O (9.7 mg, 23%). ^1^H NMR (400 MHz, methanol-*d*_4_): δ 7.95–7.87 (m, 2H), 7.82 (t, *J* = 8.4 Hz, 1H), 7.58 (t, *J* = 8.2 Hz, 1H), 7.24 (dd, *J* = 11.1, 2.4 Hz, 1H), 7.17 (dt, *J* = 8.5, 1.6 Hz, 1H), 3.83 (s, 4H), 3.80 (s, 4H). ^13^C NMR (101 MHz, methanol-*d*_4_): δ 166.5, 160.1 (d, *J* = 4.1 Hz), 158.6 (d, *J* = 17.0 Hz), 156.1 (d, *J* = 16.6 Hz), 136.0 (d, *J* = 6.4 Hz), 135.7 (d, *J* = 9.9 Hz), 128.6 (d, *J* = 2.2 Hz), 128.2 (d, *J* = 13.1 Hz), 128.0, 125.9 (d, *J* = 3.7 Hz), 125.5 (d, *J* = 12.2 Hz), 121.0 (d, *J* = 3.4 Hz), 117.5 (d, *J* = 21.8 Hz), 113.1 (d, *J* = 23.2 Hz), 44.4, 44.2. HPLC (UV) > 95%. LRMS (ESI^+^) *m*/*z*: 400.4 [M + H]. HRMS (ESI^+^) *m*/*z*: 200.5887 [M + 2H] (calcd for C_19_H_21_F_2_N_7_O, 200.5883).

#### 5.1.2 Synthesis of Bis(benzimidazoles) (**2a**, **2c–g**, **12**, **13**, and **16**)

##### 5.1.2.1 Method B

The reaction was performed in a KIMAX tube. A solution of isothiocyanate (**7c**–**g, 10**, **11**, **13**; 1 equiv) and *o*-phenylendiamine (2.2 equiv) in anhydrous DMF (4 mL) was stirred at 0 °C (ice–water bath) until complete formation of the thiourea intermediate (checked by TLC and HPLC–MS). Then, EDC hydrochloride (2.5 equiv) solid was added in one step, and the reaction mixture was allowed to stir at 60 °C for 24 h. Ice was added to the crude reaction mixture, and the tube was shaken vigorously. The solid precipitate was collected and purified by centrifugal PTLC using silica plates.

##### 5.1.2.2 4-((1*H*-Benzo[*d*]imidazol*-*2-yl)amino)-*N*-(4-((1*H*-benzo[*d*]imidazol-2-yl)amino)phenyl)benzamide (**2a**)

A solution of **7a** (200 mg, 0.64 mmol) and *o*-phenylendiamine (151 mg, 1.4 mmol) in dry DMF (10 mL) was stirred at room temperature for 2 h until complete starting material consumption. Metallic iodine (405 mg, 1.6 mmol) was added, followed by the addition of potassium carbonate (221 mg, 1.6 mmol). The resulting reaction mixture was allowed to stir overnight at room temperature. The reaction was quenched with 5% aq. Na_2_S_2_O_3_ solution (3.5 mL), diluted brine (50 mL), and extracted with CH_2_Cl_2_ (3 × 30 mL) to yield a yellowish oil. Column chromatography using silica (Hexane: EtOAc, 100:0 → 40:60) yielded **2a** as a brownish solid (178 mg, 61%). mp.: > 300 °C. ^1^H NMR (500 MHz, DMSO-*d*_6_): δ 12.97 (br s, 1H), 11.72 (s, 1H), 11.37 (s, 1H), 10.57 (s, 1H), 8.21 (d, *J* = 8.2 Hz, 2H), 8.02 (d, *J* = 8.5 Hz, 2H), 7.65 (d, *J* = 8.2 Hz, 2H), 7.51 (dd, *J* = 5.9, 3.2 Hz, 2H), 7.49–7.42 (m, 4H), 7.27 (m, 4H). ^13^C NMR (126 MHz, DMSO-*d*_6_): δ 164.6, 147.9, 146.9, 139.9, 137.6, 131.3, 129.7, 129.6, 123.7, 123.5, 121.7, 120.6, 112.3, 111.9. HPLC (UV) > 95%. LRMS (ESI^+^) *m*/*z*: 460.2 [M + H]. HRMS (ESI^+^) *m*/*z*: 460.1872 [M + H] (calcd for C_27_H_22_N_7_O, 460.1881).

##### 5.1.2.3 5-((1*H*-Benzo[*d*]imidazol-2-yl)amino)-*N*-(5-((1*H*-benzo[*d*]imidazol-2-yl)amino)pyridin-2-yl)picolinamide (**2c**)

Method B. A solution of **7c** (20 mg, 64 μmol) and *o*-phenylendiamine (15 mg, 140 μmol) in dry CH_3_CN (1 mL) was stirred at 0 °C for 48 h until complete starting material consumption. EDC hydrochloride (30.6 mg, 160 μmol) was added to the solution, and the mixture was stirred at 60 °C overnight. Ice was added to the crude to facilitate product precipitation. The precipitate was filtered on a fritted plate and washed with cold water to yield the product as a brownish solid (13 mg, 44%). mp 210.6 °C. ^1^H NMR (300 MHz, DMSO-*d*_6_): δ 11.32 (s, 1H), 11.07 (s, 1H), 10.26 (s, 1H), 10.19 (s, 1H), 9.60 (s, 1H), 8.96 (d, *J* = 2.5 Hz, 1H), 8.77 (d, *J* = 2.6 Hz, 1H), 8.66 (dd, *J* = 8.5, 2.2 Hz, 1H), 8.33 (dd, *J* = 9.0, 2.6 Hz, 1H), 8.26 (d, *J* = 8.9 Hz, 1H), 8.19 (d, *J* = 8.7 Hz, 1H), 7.52–7.23 (m, 4H), 7.11–6.93 (m, 4H). ^13^C NMR (75 MHz, DMSO-*d*_6_): δ 161.5, 150.3, 149.2, 144.5, 142.9, 142.5, 140.8, 140.4, 137.5, 137.4, 134.4, 132.9, 132.7, 126.7, 123.7, 123.0, 120.9, 120.6, 119.9, 116.5, 116.0, 113.0, 110.0, 109.5. HPLC (UV) > 95%. LRMS (ES^+^) *m*/*z*: 462.2 [M + H]. HRMS (ESI^+^) *m*/*z*: 462.1773 [M + H] (calcd for C_25_H_20_N_9_O, 462.1785).

##### 5.1.2.4 4-((1*H*-Benzo[*d*]imidazol-2-yl)amino)-*N*-(4-((1*H*-benzo[*d*]imidazol-2-yl)amino)-2-chlorophenyl)-3-chlorobenzamide (**2d**)

Compound **7d** (125 mg, 0.33 mmol) and *o*-phenylendiamine (78 mg, 0.72 mmol) were reacted according to method B for 14 h. EDC.HCl (157 mg, 0.82 mmol) was added, and the reaction mixture was stirred at 60 °C for 12 h. The crude product was precipitated by adding ice and was further purified by centrifugal PTLC (CH_2_Cl_2_:MeOH, 100:0 → 70:30) to yield **2d** as a yellowish solid (75 mg, 43%). mp.: 209.8–219.8 °C. ^1^H NMR (500 MHz, DMSO-*d*_6_): δ 11.17 (br s, 1H), 11.08 (br s, 1H), 9.94 (s, 1H), 9.70 (s, 1H), 9.08 (br s, 1H), 8.94 (br s, 1H), 8.21 (d, *J* = 2.5 Hz, 1H), 8.14 (d, *J* = 2.1 Hz, 1H), 8.03 (dd, *J* = 8.7, 2.1 Hz, 1H), 7.62 (dd, *J* = 8.7, 2.5 Hz, 1H), 7.45 (d, *J* = 8.7 Hz, 1H), 7.47–7.38 (m, 1H), 7.31 (d, *J* = 7.2 Hz, 1H), 7.11 (d, *J* = 8.9 Hz, 1H), 7.13–7.05 (m, 1H), 7.02 (q, *J* = 5.7 Hz, 2H), 6.85 (dd, *J* = 5.7, 3.2 Hz, 1H), 6.55–6.50 (m, 1H). ^13^C NMR (126 MHz, DMSO-*d*_6_): δ 163.9, 150.0, 149.5, 149.3, 139.6, 139.1, 130.4, 129.9, 129.4, 128.8, 128.5, 127.7, 127.4, 123.0, 121.0, 120.7, 118.6, 118.4, 117.2, 111.2. HPLC (UV) = 93%. LRMS (ESI^+^) *m*/*z*: 528.2 [M + H]. HRMS (ESI^+^) *m*/*z*: 528.1170 [M + H] (calcd for C_27_H_20_Cl_2_N_7_O, 528.1101).

##### 5.1.2.5 4-((1*H*-Benzo[*d*]imidazol-2-yl)amino)-*N*-(4-((1*H*-benzo[*d*]imidazol-2-yl)amino)-3-chlorophenyl)-3-chlorobenzamide (**2e**)

Compound **7e** (102 mg, 0.27 mmol) and *o*-phenylendiamine (64 mg, 0.59 mmol) were reacted according to method B for 2 h. EDC.HCl (131 mg, 0.68 mmol) was added, and the reaction mixture was stirred at 60 °C for 48 h. Compound **2e** was isolated following the general procedure and purified by centrifugal PTLC (CH_2_Cl_2_:MeOH, 100:0 → 70:30) to yield a yellowish solid (21 mg; 15%). mp 211.4–224.9 °C. ^1^H NMR (400 MHz, DMSO-*d*_6_): δ 10.93 (br s, 3H), 9.50 (br s, 2H), 9.23 (br s, 2H), 8.85 (d, *J* = 9.8 Hz, 3H), 8.72 (d, *J* = 9.8 Hz, 3H), 8.59–8.30 (m, 6H). ^13^C NMR (101 MHz, DMSO-*d*_6_): δ 161.1, 154.2, 152.1, 146.1, 144.6, 143.8, 140.7, 134.4, 133.6, 123.4, 112.9. HPLC (UV) > 95%. LRMS (ESI^+^) *m*/*z*: 528.1 [M + H]. HRMS (ESI^+^) *m*/*z*: 528.1102 [M + H] (calcd for C_27_H_20_Cl_2_N_7_O, 528.1101).

##### 5.1.2.6 4-((1*H*-Benzo[*d*]imidazol-2-yl)amino)-*N*-(4-((1*H*-benzo[*d*]imidazole-2-yl)amino)-2-chlorophenyl)-2-chlorobenzamide (**2f**)

Compound **7f** (50 mg, 0.13 mmol) and *o*-phenylendiamine (31 mg, 0.29 mmol) were reacted according to method B for 20 h. EDC.HCl (63 mg, 0.33 mmol) was added, and the reaction mixture was stirred at 60 °C for 48 h. The product was isolated following the general procedure and purified by centrifugal PTLC (CH_2_Cl_2_: MeOH–NH_3(saturated)_, 100:0 → 80:20) to yield **2f** as a yellowish solid (20 mg, 17%). mp 195.5 °C. ^1^H NMR (400 MHz, DMSO-*d*_6_): δ 11.17 (s, 1H), 11.06 (s, 1H), 9.92 (s, 1H), 9.84 (s, 1H), 9.70 (s, 1H), 8.23 (d, *J* = 2.4 Hz, 1H), 8.21 (d, *J* = 2.4 Hz, 1H), 7.69 (dd, *J* = 2.2, 8.1 Hz, 1H), 7.59–7.53 (m, 3H), 7.43 (d, *J* = 7.1 Hz, 1H), 7.40 (d, *J* = 7.1 Hz, 1H), 7.32 (t, *J* = 8.7 Hz, 2H), 7.04 (m, 4H). ^13^C NMR (101 MHz, DMSO-*d*_6_): δ 165.2, 150.0, 149.5, 143.2, 142.8, 142.6, 139.9, 132.6, 132.6, 131.0, 130.1, 129.2, 128.2, 127.5, 127.2, 120.7, 120.6, 120.3, 120.0, 117.0, 117.0, 116.3, 116.1, 115.9, 115.0, 109.8, 109.6. HPLC (UV): > 95%. LRMS (ESI^+^) *m*/*z*: 528.3 [M + H]. HRMS (ESI^+^) *m*/*z*: 528.1088 [M + H] (calcd for C_27_H_20_Cl_2_N_7_O, 528.1101).

##### 5.1.2.7 4-((1*H*-Benzo[*d*]imidazol-2-yl)amino)-*N*-(4-((1*H*-benzo[*d*]imidazol-2-yl)amino)-3-chlorophenyl)-2-chlorobenzamide (**2g**)

Compound **7g** (83 mg, 0.22 mmol) and *o*-phenylendiamine (52 mg, 0.48 mmol) were reacted according to method B for 2 h. EDC·HCl (106 mg, 0.55 mmol) was added, and the reaction mixture was stirred at 60 °C for 72 h. Compound **2g** was isolated following the general procedure and purified by centrifugal PTLC (CH_2_Cl_2_: MeOH–NH_3(saturated)_, 100:0 → 90:10) to yield a yellowish solid (68 mg; 59%). mp > 300 °C. ^1^H NMR (500 MHz, methanol–*d*_4_): δ 8.07 (d, *J* = 8.8 Hz, 1H), 8.02 (d, *J* = 2.4 Hz, 1H), 7.85 (d, *J* = 2.1 Hz, 1H), 7.57 (dd, *J* = 8.8, 2.4 Hz, 1H), 7.56 (d, *J* = 8.5 Hz, 1H), 7.53 (dd, *J* = 8.5, 2.1 Hz, 1H), 7.43–7.29 (m, 4H), 7.12 (m, 2H), 7.07 (m, 2H). ^13^C NMR (126 MHz, methanol–*d*_4_): δ 168.0, 152.4, 151.1, 144.9, 135.9, 134.5, 133.2, 131.0, 129.9, 125.5, 123.0, 122.5, 122.4, 122.2, 120.8, 119.1, 116.7. HPLC (UV) > 94%. LRMS (ESI^+^) *m*/*z*: 528.2 (M + H). HRMS (ESI^+^) *m*/*z*: 528.1073 [M + H] (calcd for C_27_H_20_Cl_2_N_7_O, 528.1101).

##### 5.1.2.8 *N*,*N*′-(Ethane-1,2-diylbis(4,1-phenylene))bis(1*H*-benzo[*d*]imidazol-2-amine) (**12**)

A solution of **10** (225 mg, 0.76 mmol) and *o*-phenylendiamine (180 mg, 1.67 mmol) in dry DMF (3 mL) was stirred at room temperature. After 15 min, the brownish, transparent solution turned into a turbid, yellowish one. After 3 h, starting material consumption was revealed by TLC. EDC hydrochloride (364 mg, 1.93 mmol) was added, and the resulting mixture was heated at 60 °C. The crude reaction mixture was extracted with H_2_O, and the combined organic phase was washed with brine, dried, and concentrated under vacuum to yield a brownish oil. The product was purified by column chromatography on silica using CH_2_Cl_2_:MeOH (100:0 → 92:8) to yield **12** as a whitish solid (182 mg, 54%). mp > 177.3 °C. ^1^H NMR (400 MHz, DMSO-*d*_6_): δ 9.29 (s, 2H), 7.63 (d, *J* = 8.5 Hz, 4H), 7.29 (dd, *J* = 5.8, 3.2 Hz, 4H), 7.16 (d, *J* = 8.5 Hz, 4H), 6.97 (dd, *J* = 5.8, 3.2 Hz, 4H), 2.83 (s, 4H). ^13^C NMR (101 MHz, DMSO-*d*_6_): δ 162.4, 150.8, 138.7, 133.8, 128.7, 120.0, 117.2, 36.7. HPLC (UV) > 95%. LRMS (ESI^+^) *m*/*z*: 445.3 [M + H]. HRMS (ESI^+^) *m*/*z*: 445.2125 [M + H] (calcd for C_28_H_25_N_6_, 445.2135).

##### 5.1.2.9 1,3-Bis(4-((1*H*-benzo[*d*]imidazol-2-yl)amino)phenyl)urea (**13**)

Compound **11** (103 mg, 0.32 mmol) and *o*-phenylendiamine (76 mg, 0.70 mmol) were reacted according to method B for 5 h. EDC.HCl (154 mg, 0.80 mmol) was added, and the reaction mixture was stirred at 60 °C for 48 h. The precipitate was filtered on a fritted plate and washed with cold water. Centrifugal PTLC on a silica plate (CH_2_Cl_2_: MeOH–NH_3(saturated)_ (98.75:1.25 → 80:20) gave **13** as a yellowish solid (38 mg; 25%). mp > 207.6 °C. ^1^H NMR (400 MHz, DMSO-*d*_6_): δ 10.86 (br s, 1H), 9.25 (br s, 1H), 8.47 (s, 1H), 7.65 (d, *J* = 8.9 Hz, 2H), 7.39 (d, *J* = 8.9 Hz, 2H), 7.28 (dd, *J* = 5.8, 3.3 Hz, 2H), 6.97 (dd, *J* = 5.8, 3.3 Hz, 2H). ^13^C NMR (101 MHz, DMSO-*d*_6_): δ 171.4, 152.9, 151.0, 135.3, 133.3, 119.8, 119.1, 117.9. HPLC (UV) > 95%. LRMS (ESI^+^) *m*/*z*: 475.4 [M + H]. HRMS (ESI^+^) *m*/*z*: 475.1980 [M + H] (calcd for C_27_H_23_N_8_O, 475.1989).

##### 5.1.2.10 *N*^2^,*N*^7^-Bis(1*H*-Benzo[*d*]imidazol-2-yl)-9*H*-fluorene-2,7-diamine (**16**)

Method B. A solution of **15** (100 mg, 0.36 mmol) and *o*-phenylendiamine (86 mg, 0.8 mmol) in dry DMF (4 mL) was stirred at room temperature. After 3 h, starting materials consumption was revealed by TLC. EDC hydrochloride (173 mg, 0.9 mmol) was added, and the resulting mixture was heated at 60 °C. Ice was added to the crude to facilitate product precipitation. The precipitate was collected by filtration and purified by centrifugal PTLC on silica using CH_2_Cl_2_:MeOH (100:0 → 70:30) to yield **16** as a whitish solid (94 mg; 61%). mp 196.3–210.2 °C. ^1^H NMR (500 MHz, DMSO-*d*_6_): δ 10.95 (br s, 2H), 9.52 (s, 2H), 8.12 (d, *J* = 2.0 Hz, 2H), 7.69 (d, *J* = 8.2 Hz, 2H), 7.61 (dd, *J* = 8.2, 2.0 Hz, 2H), 7.33 (br s, 4H), 7.07–6.92 (m, 4H), 3.95 (s, 2H). ^13^C NMR (126 MHz, DMSO-*d*_6_): δ ^13^C NMR (126 MHz, DMSO): δ 150.7, 143.6, 143.2, 139.1, 134.5, 132.8, 129.7, 120.4, 119.6, 119.2, 116.0, 115.8, 113.8, 109.4, 36. HPLC (UV) > 95%. LRMS (ESI^+^) *m*/*z*: 429.2 (M + H). HRMS (ESI^+^) *m*/*z*: 429.1811 [M + H] (calcd for C_27_H_21_N_6_, 429.1822).

#### 5.1.3 Synthesis of Bis(pyridine-2-carboxamidines) (**3a–b**, **3i–l**, **18**, **19**, **23**)

##### 5.1.3.1 Method C1

To a solution of diamine (**5a**–**c**, **8**, **9**) (0.5 g, 2.2 mmol) in a 3:1 mixture of anhydrous EtOH/CH_3_CN (30 mL) stirred at 0 °C (ice–water bath) was added slowly a solution of naphthalen-2-ylmethylpyridine-2-carbimidothioate hydrobromide (**17**) (1.97 g, 5.5 mmol, 2.5 equiv) in 6 mL of EtOH/CH_3_CN (3:1). The resulting reaction mixture was allowed to stir at room temperature for 48 h. The solvents were removed under vacuum, and the crude product was purified as specified in each case.

##### 5.1.3.2 Naphthalen-2-ylmethylpyridine-2-carbimidothioate Hydrobromide (**17**)

A solution of pyridine-2-carbothioic acid amide (6 g, 1 equiv) and 2-(bromomethyl)-naphthalene (9.6 g, 1 equiv) in dry CHCl_3_ (75 mL) was heated to reflux (65 °C) for 1.5 h. Then, the reaction mixture was cooled immediately in an ice–water bath and poured into cool Et_2_O (350 mL). The resulting suspension was filtered, and the organic phase was concentrated until dryness to yield **17** as white solid. The spectroscopic data were consistent with the literature.^94^^1^H NMR (300 MHz, DMSO-*d*_6_): δ 8.85–8.77 (m, 1H), 8.32 (dd, *J* = 8.0, 0.9 Hz, 1H), 8.19–8.10 (m, 1H), 8.08 (d, *J* = 1.7 Hz, 1H), 8.03–7.89 (m, 3H), 7.80 (ddt, *J* = 7.7, 4.7, 0.9, 0.9 Hz, 1H), 7.62 (dd, *J* = 8.4, 1.8 Hz, 1H), 7.59–7.51 (m, 2H), 4.86 (s, 2H). mp decomposes at 192 °C. Anal. Calcd (C_17_H_15_BrN_2_S) C, 56.83; H, 4.21; N, 7.80; S, 8.92. Found: C, 56.51; H, 4.26; N, 7.82; S, 9.03.

##### 5.1.3.3 4-(Picolinimidamido)-*N*-(4-(picolinimidamido)phenyl)benzamide (**3a**)

The reaction was performed following method C1 with **5a** (0.5 g, 2.2 mmol) and **17** (1.97 g, 5.5 mmol), in 6 mL of EtOH/CH_3_CN (3:1). The resulting reaction mixture was allowed to stir at room temperature for 48 h. The solvents were removed under vacuum and the crude product was purified by silica gel column chromatography using *n*-hexane:EtOAc (20:80 → 0:100). The free base of **3a** was obtained as a yellowish solid (220 mg, 23%). ^1^H NMR (400 MHz, DMSO-*d*_6_): δ 10.05 (s, 1H), 8.65 (ddt, *J* = 6.7, 4.9, 1.4 Hz, 2H), 8.33 (d, *J* = 8.0 Hz, 2H), 8.07–7.88 (m, 4H), 7.79 (d, *J* = 8.7 Hz, 2H), 7.56 (dddd, *J* = 9.0, 7.5, 4.8, 1.3 Hz, 2H), 7.05 (d, *J* = 8.0 Hz, 2H), 6.95 (d, *J* = 8.2 Hz, 2H), 6.90–6.00 (m, 4H). ^13^C NMR (101 MHz, DMSO-*d*_6_): δ 164.8, 153.7, 151.8, 151.6, 151.2, 148.1, 148.0, 145.6, 137.2, 137.1, 134.2, 129.0, 128.6, 125.6, 125.4, 121.6, 121.5, 121.4, 121.2, 113.7, 112.5. mp 246.9–259.4 °C. HPLC (UV) > 95%. LRMS (ESI^+^) *m*/*z*: 436.4 [M + H]^+^. HRMS (ESI^+^) *m*/*z*: 436.1881 [M + H]^+^ (calcd for C_25_H_22_N_7_O, 436.1880).

##### 5.1.3.4 4-(Picolinimidamido)-*N*-(5-(picolinimidamido)pyridin-2-yl)benzamide Hydrochloride Salt (**3b**)

Method C1. Diamine **5b** (86 mg, 0.38 mmol) was suspended in anhydrous acetonitrile (2 mL) and anhydrous EtOH (6 mL). The flask was cooled with an ice–water bath, and solid **17** (284 mg, 0.79 mmol) was added at once to the suspension. The thick, yellowish slurry was stirred at 0 °C and allowed to warm up to room temperature. After 5 days of stirring at room temperature, Et_2_O was added to the reaction mixture. The precipitate was collected on a fritted plate and rinsed with Et_2_O. The precipitate was dissolved in EtOH (20 mL), and the cooled solution (ice–water bath) was basified with 1 N NaOH until pH ≈ 10. The solution was concentrated under vacuum, and the product was partitioned between water (25 mL) and EtOAc (40 mL). The aqueous phase was extracted with EtOAc (2 × 50 mL), and the combined organic extracts were washed with brine, dried (Na_2_SO_4_), and evaporated to give a crude yellow residue. Silica chromatography (5 g SI cartridge) with CHCl_3_/NH_3(sat.)_-MeOH: 0 → 5% yielded a mixture (≈75/25, 38 mg) of the expected product **3b** (*M* = 436) and the monosubstituted product **3b_b** (*M* = 332). The mixture was dissolved in CH_2_Cl_2_/MeOH (2 mL), cooled with an ice bath, and treated with HCl(sat.)-dioxane solution for 1 h with gentle stirring. The monosubstituted product **3b_b** that precipitated from the reaction mixture was collected by filtration and rinsed with Et_2_O to give a colorless solid (20 mg, 12%). The filtrate was evaporated under a vacuum, and product **3b** was recrystallized from CH_2_Cl_2_/MeOH at −20 °C. The product was rinsed with Et_2_O to yield **3b** as a yellowish solid (10 mg, 6%). ^1^H NMR (400 MHz, DMSO-*d*_6_): δ 11.95 (br s, 1H), 11.81 (br s, 1H), 11.27 (s, 1H), 10.17 (br s, 2H), 9.48 (br s, 2H), 8.95–8.84 (m, 2H), 8.54 (d, *J* = 2.7 Hz, 1H), 8.49–8.38 (m, 3H), 8.29–8.21 (m, 4H), 8.10 (s, 1H), 8.01 (dd, *J* = 8.8, 2.7 Hz, 1H), 7.87 (dt, *J* = 8.0, 4.2 Hz, 2H), 7.74–7.61 (m, 2H), 7.29 (s, 1H), 7.19 (s, 1H), 7.08 (s, 1H). ^13^C NMR (126 MHz, DMSO-*d*_6_): δ 165.3, 160.2, 151.8, 149.9, 148.5, 145.9, 144.3, 138.5, 138.4, 138.3, 136.5, 130.0, 129.8, 129.0, 128.7, 125.7, 124.1, 123.9, 122.6, 119.8, 119.5, 115.3. mp > 130 °C. HPLC (UV): 93%. LRMS (ESI^+^) *m*/*z*: 437.3 [M + H]^+^. HRMS (ESI^+^) *m*/*z*: 437.1824 [M + H]^+^ (calcd for C_24_N H_21_N_8_O, 437.1833).

##### 5.1.3.5 4-Amino-*N*-(5-(picolinimidamido)pyridin-2-yl)benzamide Hydrochloride Salt (**3b_b**)

The isolated product contains ≈9% of **3b**. ^1^H NMR (400 MHz, DMSO-*d*_6_): δ 11.18 (s, 0.1H), 10.31 (s, 0.2H), 9.58 (s, 0.2H), 8.90 (dd, *J* = 4.7, 2.7 Hz, 1H), 8.56 (dd, *J* = 5.7, 2.7 Hz, 2H), 8.53 (d, *J* = 2.6 Hz, 1H), 8.39 (d, *J* = 9.1 Hz, 1H), 8.31–8.24 (m, 1H), 8.25–8.19 (m, 2H), 8.07–8.00 (m, 2H), 7.89–7.83 (m, 1H), 7.17 (d, *J* = 8.2 Hz, 1H), 5.10 (br s, 2H). ^13^C NMR (101 MHz, DMSO-*d*_6_): δ 165.6, 160.1, 151.7, 149.9, 144.8, 144.0, 138.5, 138.4, 137.4, 129.9, 129.8, 128.8, 127.0, 125.7, 124.1, 118.8, 115.3. LRMS (ESI^+^) *m*/*z*: 333 [M + H]^+^.

##### 5.1.3.6 5-Amino-*N*-(5-(picolinimidamido)pyridin-2-yl)picolinamide (**3c_c**)

Method C1. Diamine **5c** (130 mg, 0.57 mmol) was suspended in anhydrous acetonitrile (3 mL) and anhydrous EtOH (9 mL). The flask was cooled with an ice bath and solid **17** (428 mg, 1.19 mmol) was added at once to the suspension. The thick, brownish slurry was stirred at 0 °C and allowed to warm up to room temperature. After 10 days of stirring at room temperature, the reaction was concentrated under vacuum, and Et_2_O (70 mL) was added to the reaction mixture. The precipitate was collected on a fritted plate and rinsed with Et_2_O to give a beige solid (257 mg). The solid was suspended in EtOH (10 mL), and the cooled solution (ice bath) was basified with 1 N NaOH until pH ≈ 10. The mixture was partitioned between water (20 mL) and EtOAc (50 mL). The aqueous phase was extracted with EtOAc (2 × 50 mL), and the combined organic extracts were washed with brine, dried (Na_2_SO_4_), and evaporated to give an orangish residue. Silica chromatography (5 g SI cartridge) with CHCl_3_/NH_3(sat.)_-MeOH: 0 → 5% yielded the pure monosubstituted product **3c_c** as a brownish solid (40 mg, 21%). HPLC (UV) > 95%. ^1^H NMR (500 MHz, DMSO): δ 10.07 (s, 1H), 8.64 (d, *J* = 4.9 Hz, 1H), 8.33 (d, *J* = 7.9 Hz, 1H), 8.24 (d, *J* = 8.7 Hz, 1H), 8.01 (d, *J* = 2.7 Hz, 1H), 7.98 (d, *J* = 3.0 Hz, 2H), 7.95 (dd, *J* = 8.9, 2.7 Hz, 1H), 7.88 (d, *J* = 8.5 Hz, 1H), 7.56 (dd, *J* = 7.5, 4.9 Hz, 1H), 7.44 (dd, *J* = 8.6, 2.7 Hz, 1H), 7.05 (dd, *J* = 8.5, 2.7 Hz, 2H), 7.01–6.67 (m, 2H), 6.21 (s, 2H). ^13^C NMR (126 MHz, DMSO-*d*_6_): δ 162.0, 153.2, 151.2, 148.3, 148.1, 146.1, 141.5, 137.2, 136.0, 134.5, 131.3, 125.6, 123.4, 121.5, 119.4, 113.0. LRMS (ESI^+^) *m*/*z*: 334.2 [M + H]^+^.

##### 5.1.3.7 Method C2

Diamine **5i**–**l** (1 equiv) and **17** (4.5 equiv) were dissolved in anhydrous DMF (2.5 mL) in a KIMAX tube. The reaction mixture was stirred under an argon atmosphere for several days. Water (3 mL) was added, and the mixture was extracted with EtOAc and CH_2_Cl_2_ to remove organic byproducts. The aqueous layer was concentrated in vacuo to yield a brownish crude oil. Then, hexane was added, and the precipitate was collected by filtration over a Buchner funnel. The precipitate was redissolved in water, and a saturated aqueous NaHCO_3_ solution was added to precipitate the product. The precipitate was filtered over a filter plate, giving **3i**–**l** as white powder.

##### 5.1.3.8 3-Fluoro-*N*-(2-fluoro-4-(picolinimidamido)phenyl)-4-(picolinimidamido)benzamide (**3i**)

Method C2. Starting from a mixture of diamines **5i** (23.1 mg, 0.09 mmol) and **17** (157.6 mg, 0.44 mmol). After 12 days of stirring at room temperature, **3i** was isolated as a white powder (25.9 mg, 63%). HPLC (UV): >95%. ^1^H NMR (300 MHz, DMSO-*d*_6_): δ 9.94 (s, 1H), 8.65 (d, *J* = 4.9 Hz, 2H), 8.42–8.21 (m, 2H), 7.97 (t, *J* = 7.8 Hz, 2H), 7.90–7.76 (m, 2H), 7.62–7.54 (m, 2H), 7.49 (t, *J* = 8.6 Hz, 1H), 7.36–6.33 (m, 7H). ^13^C NMR (101 MHz, DMSO-*d*_6_): δ 164.1, 156.5 (d, *J* = 246.7 Hz), 153.6 (d, *J* = 243.2 Hz), 153.1, 152.7, 150.92, 150.86, 148.2, 141.6 (d, *J* = 13.5 Hz), 137.4 (d, *J* = 4.3 Hz), 128.8 (d, *J* = 6.2 Hz), 128.0 (d, *J* = 3.1 Hz), 125.8, 124.6 (d, *J* = 3.0 Hz), 124.1 (d, *J* = 3.4 Hz), 121.8, 121.6, 120.1 (d, *J* = 12.9 Hz), 117.8, 115.6 (d, *J* = 21.7 Hz), 109.3 (d, *J* = 20.5 Hz). mp 228.8–230.6 °C. LRMS (ESI^+^) *m*/*z*: 472 [M + H]^+^. HRMS (ESI^+^) *m*/*z*: 472.1694 [M + H]^+^ (calcd for C_25_H_20_F_2_N_7_O, 472.1692).

##### 5.1.3.9 3-Fluoro-*N*-(3-fluoro-4-(picolinimidamido)phenyl)-4-(picolinimidamido)benzamide (**3j**)

Method C2. Starting from a mixture of diamines **5j** (13.5 mg, 0.05 mmol) and **17** (92 mg, 0.26 mmol). After 12 days of stirring at room temperature, **3j** was isolated as a white powder (14.8 mg, 61%). HPLC (UV): > 95%. ^1^H NMR (400 MHz, DMSO-*d*_6_): δ 10.22 (s, 1H), 8.69–8.61 (m, 2H), 8.35–8.29 (m, 2H), 8.02–7.92 (m, 2H), 7.89–7.78 (m, 3H), 7.64–7.51 (m, 3H), 7.42–6.38 (m, 6H). ^13^C NMR (126 MHz, DMSO-*d*_6_): δ 163.9, 153.6 (d, *J* = 243.1 Hz), 153.2 (d, *J* = 240.8 Hz), 153.1, 153.0, 151.2, 150.9, 148.1 (d, *J* = 13.8 Hz), 141.6 (d, *J* = 13.6 Hz), 137.3 (d, *J* = 15.3 Hz), 134.9 (d, *J* = 10.2 Hz), 133.1 (d, *J* = 13.5 Hz), 129.4 (d, *J* = 6.1 Hz), 125.7 (d, *J* = 26.8 Hz), 124.4 (d, *J* = 3.1 Hz), 124.0 (d, *J* = 3.3 Hz), 123.8 (d, *J* = 4.2 Hz), 121.7 (d, *J* = 29.4 Hz), 116.5 (d, *J* = 3.0 Hz), 115.4 (d, *J* = 22.0 Hz), 108.3 (d, *J* = 25.3 Hz). mp 242.1–243.8 °C. LRMS (ESI^+^) *m*/*z*: 472 [M + H]^+^. HRMS (ESI^+^) *m*/*z*: 472.1693 [M + H]^+^ (calcd for C_25_H_20_F_2_N_7_O, 472.1692).

##### 5.1.3.10 2-Fluoro-*N*-(2-fluoro-4-(picolinimidamido)phenyl)-4-(picolinimidamido)benzamide (**3k**)

Method C2. Starting from a mixture of diamines **5k** (15.5 mg, 0.06 mmol) and **17** (105.8 mg, 0.29 mmol). After 5 days of stirring at room temperature, **3k** was isolated as a white powder (21.8 mg, 79%). HPLC (UV): > 95%. ^1^H NMR (400 MHz, DMSO-d_6_): δ 10.49 (s, 1H), 10.39–9.13 (m, 3H), 9.00–8.82 (m, 2H), 8.37 (d, *J* = 7.9 Hz, 2H), 8.23 (qd, *J* = 8.2, 1.7 Hz, 2H), 8.05 (t, *J* = 8.5 Hz, 1H), 7.97–7.79 (m, 3H), 7.75–7.24 (m, 4H), 4.25–3.25 (br, 2H), ^13^C NMR (101 MHz, DMSO-*d*_6_): δ 162.4, 159.7 (d, *J* = 250.9), 159.7, 158.1, 157.8, 157.5, 154.5 (d, *J* = 248.5 Hz), 149.9, 149.7, 145.3, 144.6, 138.4 (d, *J* = 11.1 Hz), 132.11 (d, *J* = 9.6 Hz), 131.6 (d, *J* = 3.9 Hz), 128.6 (d, *J* = 25.1 Hz), 126.0, 125.78 (d, *J* = 11.8 Hz), 123.9, 123.8, 122.4, 121.7, 118.7, 115.7, 114.3 (d, *J* = 22.3 Hz), 113.8 (d, *J* = 24.8 Hz). mp 218.2–219.9 °C. LRMS (ESI^+^) *m*/*z*: 472 [M + H]^+^. HRMS (ESI^+^) *m*/*z*: = 472.1696 [M + H]^+^ (calcd for C_25_H_20_F_2_N_7_O, 472.1692).

##### 5.1.3.11 2-Fluoro-*N*-(3-fluoro-4-(picolinimidamido)phenyl)-4-(picolinimidamido)benzamide (**3l**)

Method C2. Starting from a mixture of diamine **5l** (10.0 mg, 0.04 mmol) and **17** (62.0 mg, 0.17 mmol). After 2 days of stirring at room temperature, **3l** was isolated as a white powder (14 mg, 78%). HPLC (UV): > 95%. ^1^H NMR (400 MHz, DMSO-*d*_6_): δ 10.28 (s, 1H), 8.69–8.60 (m, 2H), 8.36–8.26 (m, 2H), 8.02–7.92 (m, 2H), 7.76 (dd, *J* = 13.0, 2.3 Hz, 1H), 7.66 (t, *J* = 8.4 Hz, 1H), 7.58 (dddd, *J* = 7.9, 6.9, 4.8, 1.2 Hz, 2H), 7.46 (dd, *J* = 8.5, 2.2 Hz, 1H), 7.02–6.93 (m, 2H), 6.92–6.62 (m, 5H). ^13^C NMR (101 MHz, DMSO-*d*_6_): δ 163.1, 160.7 (d, *J* = 249.6 Hz), 155.8 (d, *J* = 10.1 Hz), 153.8 (d, *J* = 240.3 Hz), 151.7, 151.5, 148.7, 148.6, 137.8 (d, *J* = 8.4 Hz), 135.4 (d, *J* = 10.2 Hz), 133.7 (d, *J* = 13.6 Hz)), 131.4 (d, *J* = 4.3 Hz), 126.2 (d, *J* = 18.8 Hz), 124.4 (d, *J* = 4.3 Hz), 122.1, 122.1, 118.6, 118.4, 116.5, 109.7 (d, *J* = 23.1 Hz), 108.3 (d, *J* = 25.0 Hz). mp 212.7–214.2 °C. LRMS (ESI^+^) *m*/*z*: 472 [M + H]^+^. HRMS (ESI^+^) *m*/*z*: = 472.1695 [M + H]^+^ (calcd for C_25_H_20_F_2_N_7_O, 471.1692).

##### 5.1.3.12 *N*,*N*″-(Ethane-1,2-diylbis(4,1-phenylene))dipicolinimidamide (**18**)

The reaction was performed following the general Method C1 with commercial 4,4′-diaminobibenzyl (106 mg, 0.5 mmol) and **17** (449 mg, 1.25 mmol) suspended in a 3:1 mixture of anhydrous EtOH/CH_3_CN (16 mL). The mixture was allowed to stir at room temperature overnight. The solvent was eliminated under a vacuum, and the solid crude was purified by silica column chromatography using hexane/EtOAc (100:0 → 10:90) as the elution system. Product **18** was obtained as a yellowish solid (51 mg, 24%). ^1^H NMR (500 MHz, DMSO-*d*_6_): δ 8.63 (ddd, *J* = 4.8, 1.8, 1.1 Hz, 2H), 8.31 (d, *J* = 7.8 Hz, 2H), 7.95 (td, *J* = 7.8, 1.8 Hz, 2H), 7.55 (ddd, *J* = 7.8, 4.8, 1.1 Hz, 2H), 7.33–7.17 (m, 4H), 6.88 (d, *J* = 7.8 Hz, 4H), 6.81–6.10 (br, 4H) 2.87 (s, 4H). ^13^C NMR (126 MHz, DMSO-*d*_6_): δ 151.8, 151.4, 148.0, 147.5, 137.1, 135.6, 129.2, 125.4, 121.4, 121.3, 36.9. mp 174.7–180.0 °C. HPLC (UV) > 95%. LRMS (ESI^+^) *m*/*z*: 421.2 [M + H]^+.^ HRMS (ESI^+^) *m*/*z*: = 421.2132[M + H]^+^ (calcd for C_26_H_25_N_6_, 421.2135).

##### 5.1.3.13 *N*,*N*′-((Carbonylbis(azanediyl))bis(4,1-phenylene))dipicolinimidamide (**19**)

Method C2 starting from a mixture of diamines **9** (16.1 mg, 0.07 mmol) and **17** (95.5 mg, 0.27 mmol). After 4 h stirring at room temperature, **19** was isolated as an off-white powder (24.7 mg, 83%). mp: desc >300 °C. ^1^H NMR (500 MHz, DMSO-*d*_6_): δ 8.65–8.60 (m, 2H), 8.51 (s, 2H), 8.31 (d, *J* = 7.9 Hz, 2H), 7.94 (td, *J* = 7.7, 1.8 Hz, 2H), 7.58–7.51 (m, 2H), 7.47–7.44 (d, *J* = 8.5 Hz, 4H), 6.89 (d, *J* = 8.5 Hz, 4H), 6.75–6.2 (br, 4H). ^13^C NMR (101 MHz, DMSO-*d*_6_): δ 152.8, 151.8, 151.6, 148.0, 144.0, 137.1, 134.7, 125.3, 121.9, 121.2, 119.3. HPLC (UV) > 95%. LRMS (ESI^+^) *m*/*z*: 451.3 [M + H]. HRMS (ESI^+^) *m*/*z*: = 451.1981 [M + H] (calcd for C_25_H_23_N_8_O, 451.1989).

#### 5.1.4 Synthesis of Bis(pyridine-2-carboxamidine) Salts (**3c–l**, **23**) Starting from the Boc-Protected Precursors (**22**, **24c–l**)

##### 5.1.4.1 Method D

To a cooled (ice–water bath) solution of the Boc-protected bis(pyridine-2-carboxamidines) (**22**, **24c**–**l**) (scale: 10–50 mg) in CH_2_Cl_2_ (2–3 mL) was added slowly TFA (2 mL) or 4 M HCl–dioxane solution (2 mL). The resulting solution was stirred for 2 h at 0 °C. The solvents were removed under vacuum to give a crude oil which was dried under a high vacuum. The crude product was triturated with Et_2_O to precipitate the bis(pyridine-2-carboxamidine) (**3c**–**l**, **23**) as a powder.

##### 5.1.4.2 5-(Picolinimidamido)-*N*-(5-(picolinimidamido)pyridin-2-yl)picolinamide Ditrifluoroacetate Salt (**3c**)

The reaction was performed with **24c** (80 mg, 0.13 mmol) and TFA following method D. Compound **3c** was obtained as a brownish solid (78 mg, 90%). ^1^H NMR (500 MHz, DMSO-*d*_6_): δ 11.68 (br s, 1H), 10.55 (s, 1H), 9.44 (br s, 1H), 8.95–8.87 (m, 2H), 8.85–8.79 (m, 2H), 8.48–8.40 (m, 5H), 8.27 (d, *J* = 8.1 Hz, 2H), 8.22 (d, *J* = 9.9 Hz, 1H), 7.98–7.80 (m, 4H). ^13^C NMR (101 MHz, DMSO-*d*_6_): δ 163.0, 162.2, 150.2, 150.0, 149.2, 148.9, 144.7, 135.0, 129.3, 129.2, 124.3, 124.2, 123.9, 119.8, 114.2, 113.6. mp 135.0–138.0 °C. HPLC (UV): > 95%. LRMS (ESI^+^) *m*/*z*: 438.3 [M + H]. HRMS (ESI^+^) *m*/*z*: 219.0932 [M + 2H]^2+^ (calcd for C_24_H_22_N_8_O, 219.0953).

##### 5.1.4.3 3-Chloro-*N*-(2-chloro-4-(picolinimidamido)phenyl)-4-(picolinimidamido)benzamide Ditrifluoroacetate Salt (**3d**)

The reaction was performed with **24d** (50 mg, 70 μmol) and TFA following method D. Compound **3d** was obtained as a yellowish solid (45 mg, 88%). ^1^H NMR (500 MHz, DMSO-*d*_6_): δ 12.0–11.4 (br, 1H), 10.41 (s, 1H), 10.09 (br s, 1H), 9.56 (br s, 1H), 8.91 (d, *J* = 4.4 Hz, 1H), 8.84 (d, *J* = 5.3 Hz, 1H), 8.42–8.32 (m, 2H), 8.32–8.20 (m, 3H), 8.17 (t, *J* = 7.8, 7.8 Hz, 1H), 8.09 (dd, *J* = 8.3, 2.0 Hz, 1H), 7.87 (dd, *J* = 8.3, 4.7 Hz, 1H), 7.82–7.73 (m, 3H), 7.61–7.46 (m, 2H), 3.63 (br s, 2H). ^13^C NMR (126 MHz, DMSO-*d*_6_): δ 163.8, 159.6, 149.9, 149.4, 144.6, 138.5, 138.2, 134.9, 130.1, 129.6, 129.3, 128.7, 128.1, 127.2, 125.2, 123.9, 123.1. mp 118.1–120.0 °C. HPLC (UV) > 95%. LRMS (ESI^+^) *m*/*z*: 504.32 [M + H]^+^. HRMS (ESI^+^) *m*/*z*: = 504.1087 [M + H]^+^ (calcd for C_25_H_20_Cl_2_N_7_O, 504.1101).

##### 5.1.4.4 3-Chloro-*N*-(3-chloro-4-(picolinimidamido)phenyl)-4-(picolinimidamido)benzamide Ditrifluoroacetate Salt (**3e**)

The reaction was performed with **24e** (25 mg, 36 μmol) and TFA following method D. Compound **3e** was obtained as a yellowish solid (20 mg, 76%). ^1^H NMR (400 MHz, DMSO-*d*_6_): δ 12.0–11.5 (br, 1H), 10.70 (s, 1H), 10.23–9.92 (br, 1H), 9.46–9.01 (br, 1H), 8.91 (d, *J* = 4.8 Hz, 1H), 8.84–8.78 (m, 1H), 8.38 (d, *J* = 8.1 Hz, 2H), 8.29–8.21 (m, 3H), 8.16 (s, 1H), 8.05 (d, *J* = 8.3 Hz, 1H), 7.96–7.85 (m, 2H), 7.78 (s, 1H), 7.58 (d, *J* = 8.7 Hz, 1H), 7.48 (br s, 1H), 7.38–7.27 (m, 1H). ^13^C NMR (101 MHz, DMSO-*d*_6_): δ 164.1, 158.0, 150.0, 149.2, 144.2, 138.7, 138.4, 129.4, 128.8, 128.1, 123.7, 122.9, 121.3, 120.1. mp 130.7 °C. HPLC (UV) > 95%. LRMS (ESI^+^) *m*/*z*: 504.3 [M + H]^+^. HRMS (ESI^+^) *m*/*z*: = 504.1087 [M + H]^+^ (calcd for C_25_H_20_Cl_2_N_7_O, 504.1101).

##### 5.1.4.5 2-Chloro-*N*-(2-chloro-4-(picolinimidamido)phenyl)-4-picolinimidamido)benzamide Dihydrochloride (**3f**)

The reaction was performed with **24f** (57 mg, 80 μmol) in dry CH_2_Cl_2_ (1.5 mL) and 4 M HCl–dioxane solution following method D. The crude product was crushed with Et_2_O to yield **3f** as a brownish solid (31 mg, 67%). ^1^H NMR (400 MHz, DMSO-*d*_6_): δ 10.46 (s, 1H), 10.02 (br s, 1H), 9.41 (br s, 1H), 8.90 (br s, 2H), 8.37 (d, *J* = 7.8 Hz, 2H), 8.28–8.18 (m, 2H), 7.99–7.78 (m, 4H), 7.72 (m, 2H), 7.50 (d, *J* = 9.1 Hz, 2H), 7.38–7.30 (m, 2H). ^13^C NMR (101 MHz, DMSO-*d*_6_): δ 164.9, 154.3, 149.9, 149.8, 138.6, 131.2, 130.5, 128.8, 128.7, 128.5, 128.1, 127.9, 127.6, 127.4, 125.3, 123.9. mp > 130.2 °C. HPLC (UV) > 95%. LRMS (ESI^+^) *m*/*z*: 504.1 [M + H]^+^. HRMS (ESI^+^) *m*/*z*: = 504.1098 [M + H]^+^ (calcd for C_25_H_20_Cl_2_N_7_O, 504.1101).

##### 5.1.4.6 2-Chloro-*N*-(3-chloro-4-(picolinimidamido)phenyl)-4-(picolinimidamido)benzamide Ditrifluoroacetate Salt (**3g**)

The reaction was performed with **24g** (30 mg, 40 μmol) and TFA according to method D. After 2 h, excess TFA was removed under a vacuum. The crude product was dissolved in MeOH, and the solvent was evaporated to give a solid that was dried under a high vacuum. The purplish solid was crushed with Et_2_O to yield pure **3g** as a whitish solid (24 mg, 82%). ^1^H NMR (400 MHz, DMSO-*d*_6_): δ 12.05–11.61 (br, 1H), 11.01 (s, 1H), 10.23–9.83 (br, 1H), 9.47–9.15 (br, 1H), 8.92–8.90 (m, 1H), 8.89–8.87 (m, 1H), 8.46–8.31 (m, 2H), 8.29–8.15 (m, 3H), 7.90–7.81 (m, 2H), 7.81–7.75 (m, 2H), 7.69 (br s, 1H), 7.58 (d, *J* = 8.6 Hz, 1H), 7.50 (br s, 1H), 3.52 (br s, 3H). ^13^C NMR (101 MHz, DMSO-*d*_6_): δ 164.9, 159.8, 150.0, 149.9, 149.7, 149.5, 144.3, 140.3, 138.5, 138.3, 131.0, 130.9, 130.2, 129.6, 128.7, 128.3, 124.5, 123.6, 120.6, 119.5. mp 214.0–222.9 °C. LRMS (ESI^+^) *m*/*z*: 504.1 [M + H]^+^. HRMS (ESI^+^) *m*/*z*: = 504.1089 [M + H]^+^ (calcd for C_25_H_20_Cl_2_N_7_O, 504.1101).

##### 5.1.4.7 2-Isopropoxy-*N*-(2-isopropoxy-4-(picolinimidamido)phenyl)-4-(picolinimidamido)benzamide Dihydrochloride (**3h**)

The reaction was performed with **24h** (45 mg; 60 μmol) in dry CH_2_Cl_2_ (3 mL) and 4 M HCl–dioxane solution following method D. The crude product was crushed with Et_2_O to yield **3h** as a brownish solid (24 mg, 65%). ^1^H NMR (400 MHz, DMSO-*d*_6_): δ 9.83 (s, 1H), 9.21 (s, 1H), 8.89 (d, *J* = 4.8 Hz, 1H), 8.74–8.61 (m, 1H), 8.40 (dd, *J* = 8.5, 4.6 Hz, 1H), 8.22 (m, 1H), 8.17–7.97 (m, 1H), 7.85 (dd, *J* = 7.7, 4.8 Hz, 1H), 7.77 (dd, *J* = 9.9, 8.5 Hz, 2H), 7.14 (d, *J* = 2.3 Hz, 1H), 6.93 (dd, *J* = 8.5, 2.3 Hz, 1H), 6.45–6.41 (m, 1H), 6.36–6.32 (m, 1H), 4.75–4.58 (m, 4H), 3.73–3.64 (m, 1H), 3.52–3.43 (m, 1H), 1.44–1.36 (m, 12H). ^13^C NMR (101 MHz, DMSO-*d*_6_): δ 163.2, 159.5, 157.3, 149.8, 149.0, 148.6, 148.3, 147.4, 147.1, 144.5, 138.5, 138.4, 137.9, 133.1, 129.1, 128.6, 128.0, 126.5, 123.8, 122.6, 122.0, 121.4, 114.9, 108.6, 107.8, 72.2, 71.9, 22.0, 21.9. mp 142.9–150.0 °C. HPLC (UV) > 95%. LRMS (ESI^+^) *m*/*z*: 552.2 [M + H]^+^. HRMS (ESI^+^) *m*/*z*: = 552.2712 [M + H]^+^ (calcd for C_31_H_34_N_7_O_3_, 552.2718).

##### 5.1.4.8 3-Fluoro-*N*-(2-fluoro-4-(picolinimidamido)phenyl)-4-(picolinimidamido)benzamide Ditrifluoroacetate (**3i**)

The reaction was performed with **24i** (10.7 mg; 0.016 mmol) and TFA according to method D. Compound **3i** was obtained as a yellowish solid (7.5 mg, 80%). ^1^H NMR (300 MHz, DMSO-*d*_6_): δ 10.24 (s, 1H), 9.44 (br s, 1H), 8.89 (d, *J* = 4.0 Hz, 1H), 8.76 (d, *J* = 3.5 Hz, 1H), 8.37 (d, *J* = 7.9 Hz, 3H), 8.23 (t, *J* = 7.8 Hz, 1H), 8.1 (t, *J* = 7.7 Hz, 1H), 8.02–7.67 (m, 7H), 7.46 (d, *J* = 10.8 Hz, 1H), 7.40–7.24 (m, 2H). > 95%; LRMS (ESI^+^) *m*/*z*: 472 [M + H]^+^.

##### 5.1.4.9 3-Fluoro-*N*-(3-fluoro-4-(picolinimidamido)phenyl)-4-(picolinimidamido)benzamide Ditrifluoroacetate (**3j**)

The reaction was performed with **24j** (6.50 mg, 0.010 mmol) and TFA according to method D. Compound **3j** was obtained as a yellowish solid (5.5 mg, 97%). ^1^H NMR (300 MHz, DMSO): δ 11.63 (br s, 1H), 10.74 (s, 1H), 10.12 (br s, 1H), 9.38 (br s, 1H), 8.96–8.79 (m, 2H), 8.47–7.67 (m, 9H), 7.66–7.41 (m, 2H). HPLC (UV) > 95%; LRMS (ESI^+^) *m*/*z*: 472 [M + H]^+^.

##### 5.1.4.10 2-Fluoro-*N*-(2-fluoro-4-(picolinimidamido)phenyl)-4-(picolinimidamido)benzamide Ditrifluoroacetate (**3k**)

The reaction was performed with **24k** (15.3 mg; 0.023 mmol) and TFA according to method D. Compound **3k** was obtained as a yellowish solid (12.6 mg, 94%). ^1^H NMR (300 MHz, DMSO-*d*_6_): δ 10.25 (br s, 1H), 8.83 (s, 2H), 8.47 (s, 1H), 8.30 (s, 4H), 7.89 (s, 5H), 7.13 (s, 6H). HPLC (UV) > 95%; LRMS (ESI^+^) *m*/*z*: 472 [M + H].

##### 5.1.4.11 2-Fluoro-*N*-(3-fluoro-4-(picolinimidamido)phenyl)-4-(picolinimidamido)benzamide Ditrifluoroacetate (**3l**)

The reaction was performed with **24l** (12.7 mg; 0.019 mmol) and TFA according to method D. Compound **3l** was obtained as a yellowish solid (10.3 mg, 93%). ^1^H NMR (300 MHz, DMSO-*d*_6_): δ 11.72 (br s, 1H), 10.90 (s, 1H), 10.06 (br s, 1H), 9.30 (br s, 2H), 8.89 (t, *J* = 5.8 Hz, 2H), 8.38 (d, *J* = 8.1 Hz, 2H), 8.30–8.17 (m, 2H), 7.97 (d, *J* = 12.8 Hz, 1H), 7.86 (dt, *J* = 13.9, 7.7 Hz, 3H), 7.65 (d, *J* = 8.9 Hz, 1H), 7.53 (t, *J* = 8.7 Hz, 1H), 7.45 (d, *J* = 9.8 Hz, 1H), 7.34 (d, *J* = 8.5 Hz, 1H). HPLC (UV) = 95%; LRMS (ESI^+^) *m*/*z*: 472 [M + H].

##### 5.1.4.12 *N*-(4-(4-(Picolinamido)benzamido)phenyl)picolinamide (**20**)

A suspension of **5a** (150 mg, 0.7 mmol) and picolinyl chloride (294 mg, 1.7 mmol) in dry THF (8 mL) was stirred at room temperature. Et_3_N (140 mg, 1.4 mmol) was added dropwise, turning the brown into a whitish solution. After 2 h, the reaction mixture was diluted with CH_2_Cl_2_ whereupon a whitish precipitate appeared. The product was collected by filtration as a whitish solid (271 mg, 94%). ^1^H NMR (500 MHz, DMSO-*d*_6_): δ 10.91 (s, 1H), 10.62 (s, 1H), 10.18 (s, 1H), 8.79–8.73 (m, 2H), 8.22–8.15 (m, 2H), 8.12–8.05 (m, 4H), 8.00 (d, *J* = 8.9 Hz, 2H), 7.89 (d, *J* = 8.9 Hz, 2H), 7.77 (d, *J* = 8.9 Hz, 2H), 7.73–7.65 (m, 2H). ^13^C NMR (126 MHz, DMSO-*d*_6_): δ 164.7, 162.9, 162.2, 150.0, 149.7, 148.5, 148.4, 141.3, 138.2, 138.1, 135.3, 134.0, 129.9, 128.4, 127.2, 126.9, 122.6, 122.3, 120.6, 120.5, 119.5. HPLC (UV) = 93%. LRMS (ESI^+^) *m*/*z*: 438.2 [M + H]. HRMS (ESI^+^) *m*/*z*: 438.1555 [M + H]^+^ (calcd for C_25_H_20_N_5_O_3_, 438.1561).

##### 5.1.4.13 *N*-(7-Benzimidamido-9*H*-fluoren-2-yl)picolinimidamide Dihydrochloride Salt (**23**)

The reaction was performed with **22** (59 mg, 0.1 mmol) in dry CH_2_Cl_2_ (2 mL) and 4 M HCl–dioxane solution following method D. Compound **23** was obtained as a yellowish solid (33.4 mg, 83%). Spectroscopic data were consistent with the literature.^25^^1^H NMR (400 MHz DMSO-*d*_6_): δ 10.18 (br s, 2H), 9.39 (br s, 2H), 8.89 (d, *J* = 7.2 Hz, 2H), 8.58 (d, *J* = 8.0 Hz, 2H), 8.14–8.24 (m, 4H), 7.83–7.87 (m, 2H), 7.74 (s, 2H), 7.53 (d, *J* = 8.0 Hz, 2H), 4.08 (s, 2H). ^13^C NMR (101 MHz DMSO-*d*_6_): δ 159.8, 149.7, 144.0, 143.9, 140.4, 138.5, 133.5, 128.6, 124.9, 124.2, 122.9, 121.8, 36.8. HPLC (UV) > 95%. LRMS (ESI^+^) *m*/*z*: 405.38 [M + H]^+^. HRMS (ESI^+^) *m*/*z*: 203.0943 [M + 2H]^2+^ (calcd for C_26_H_21_N_5_, 203.0948).

#### 5.1.5 Synthesis of Boc-Protected Bis(imidazolidin-2-imines) Derivatives (**6c–i**)

##### 5.1.5.1 Method E

Mercury(II) chloride (3 equiv) was added to a cooled solution (ice–water bath) of di-*tert*-butyl 2-thioxoimidazolidine-1,3-dicarboxylate^23^ (3 equiv), diamine **5c**–**i** (1 equiv., 100–300 mg scale), and anhydrous triethylamine (7 equiv) in dry DMF (3 mL/0.7 mmol) under an argon atmosphere. The reaction mixture was stirred for 1 h at 0 °C. Then, the ice–water bath was removed, and the reaction mixture was stirred at 60 °C for the time specified in each case. The reaction mixture was diluted with CH_2_Cl_2_ and filtered over celite using a mixture of CH_2_Cl_2_:MeOH (1:1, 200 mL). The solvent was removed under vacuum, and the crude product was diluted with EtOAc (100 mL) and extracted with water (3 × 150 mL). The organic phase was washed with brine, dried over MgSO_4_, and evaporated under vacuum. The pure compounds were obtained by crystallization or by silica centrifugal thin-layer chromatography, as specified in each case.

##### 5.1.5.2 Di-*tert*-butyl-2-((6-(5-((1,3-bis(*tert*-butoxycarbonyl)imidazolidin-2-ylidene)amino)picolinamido)pyridin-3-yl)imino)imidazolidine-1,3-dicarboxylate (**6c**)

The reaction was performed with **5c** (229 mg, 1.0 mmol), di-*tert*-butyl 2-thioxoimidazolidine-1,3-dicarboxylate (907 mg, 3.0 mmol), HgCl_2_ (813 mg, 3.0 mmol), and Et_3_N (708 mg, 7 mmol) in dry DMF (5 mL) according to method E. The reaction mixture was stirred at 60 °C for 6 days. The crude product was purified by centrifugal PTLC using a silica plate previously neutralized with *n*-hexane (235 mL) and Et_3_N (15 mL). The elution system was prepared with a petroleum ether/EtOAc mixture (8:2 → 6:4 → 0:1). **6c** was obtained as a brownish solid (358 mg; 47%). mp 93.0–103.0 °C. ^1^H NMR (400 MHz, DMSO-*d*_6_): δ 10.13 (s, 1H), 8.19 (d, *J* = 2.4 Hz, 1H), 8.14 (d, *J* = 8.8 Hz, 1H), 8.03 (d, *J* = 8.4 Hz, 1H), 7.89 (d, *J* = 2.6 Hz, 1H), 7.41 (dd, *J* = 8.4, 2.4 Hz, 1H), 7.34 (dd, *J* = 8.8, 2.6 Hz, 1H), 3.81 (s, 4H), 3.78 (s, 4H), 1.30 (br s, 36H). ^13^C NMR (101 MHz, DMSO-*d*_6_): δ 161.5, 149.4, 149.2, 149.0, 145.2, 142.1, 142.0, 141.5, 141.4, 140.9, 140.6, 129.5, 127.8, 122.5, 112.7, 82.0, 81.7, 43.2, 43.1, 27.5, 27.4. HPLC (UV) > 95%. LRMS (ESI^+^) *m*/*z*: 766.8 [M + H].

##### 5.1.5.3 Di-*tert*-butyl-2-((4-((4-((1,3-bis(*tert*-butoxycarbonyl)imidazolidin-2-ylidene)amino)-2-chlorophenyl)carbamoyl)-2-chlorophenyl)imino)imidazolidine-1,3-dicarboxylate (**6d**)

The reaction was performed with **5d** (200 mg, 0.68 mmol), di-*tert*-butyl 2-thioxoimidazolidine-1,3-dicarboxylate (617 mg, 2.04 mmol), HgCl_2_ (553 mg, 2.04 mmol), and Et_3_N (482 mg, 4.76 mmol) according to method E. The reaction mixture was stirred at 60 °C for 6 days. The crude product was purified by centrifugal PTLC using silica plates previously neutralized with *n*-hexane (235 mL) and Et_3_N (15 mL); petroleum ether/EtOAc was used as an elution system (8:2 → 6:4 → 0:1). **6d** was obtained as an orangish solid (302 mg; 54%). mp > 115.3 °C. ^1^H NMR (300 MHz, Chloroform-*d*): δ 8.32 (d, *J* = 8.8 Hz, 1H), 8.19 (s, 1H), 7.87 (d, *J* = 2.1 Hz, 1H), 7.61 (dd, *J* = 8.4, 2.1 Hz, 1H), 7.03 (d, *J* = 2.4 Hz, 1H), 6.99 (d, *J* = 8.4 Hz, 1H), 6.90 (dd, *J* = 8.8, 2.4 Hz, 1H), 3.80 (s, 4H), 3.79 (s, 4H), 1.33 (s, 18H), 1.32 (s, 18H). ^13^C NMR (101 MHz, chloroform-*d*): δ 163.7, 150.2, 150.0, 149.5, 145.2, 140.1, 140.0, 129.6, 128.8, 128.7, 126.2, 125.6, 123.1, 122.1, 121.9, 121.7, 120.8, 83.3, 83.2, 43.3, 42.9, 28.03, 28.02. HPLC (UV) > 95%. LRMS (ESI^+^) *m*/*z*: 832.7 [M + H].

##### 5.1.5.4 Di-*tert*-butyl-2-((4-(4-((1,3-bis(*tert*-butoxycarbonyl)imidazolidin-2-ylidene)amino)-3-chlorobenzamido)-2-chlorophenyl)imino)imidazolidine-1,3-dicarboxylate (**6e**)

The reaction was performed with **5e** (200 mg, 0.68 mmol), di-*tert*-butyl 2-thioxoimidazolidine-1,3-dicarboxylate (617 mg, 2.04 mmol), HgCl_2_ (553 mg, 2.04 mmol), and Et_3_N (482 mg, 4.76 mmol) according to method E. The reaction mixture was stirred at 60 °C for 6 days. The crude product was purified by centrifugal PTLC using silica plates previously neutralized with *n*-hexane (235 mL) and Et_3_N (15 mL). Petroleum ether/EtOAc was used as the elution system (8:2 → 6:4 → 0:1) **6e** was obtained as a yellowish solid (268 mg; 47.3%). mp 188.7–211.2 °C. ^1^H NMR (400 MHz, DMSO-*d*_6_): δ 10.07 (s, 1H), 8.02 (d, *J* = 2.1 Hz, 1H), 7.91 (d, *J* = 2.4 Hz, 1H), 7.78 (dd, *J* = 8.4, 2.1 Hz, 1H), 7.52 (dd, *J* = 8.7, 2.4 Hz, 1H), 7.01 (d, *J* = 8.4 Hz, 1H), 6.90 (d, *J* = 8.7 Hz, 1H), 3.80 (s, 4H), 3.77 (s, 4H), 1.31 (br s, 36H). ^13^C NMR (101 MHz, DMSO-*d*_6_): δ 163.5, 149.6, 149.4, 149.3, 141.7, 140.5, 139.6, 134.1, 128.5, 128.0, 126.8, 125.3, 125.2, 120.9, 120.4, 120.3, 119.2, 81.9, 81.6, 42.8, 42.7, 27.51, 27.50. HPLC (UV) > 95%. LRMS (ESI^+^) *m*/*z*: 832.7 [M + H].

##### 5.1.5.5 Di-*tert*-butyl 2-((4-(4-((1,3-bis(*tert*-butoxycarbonyl)imidazolidin-2-ylidene)amino)-2-chlorobenzamido)-3-chlorophenyl)imino)imidazolidine-1,3-dicarboxylate (**6f**)

The reaction was performed with **5f** (200 mg, 0.68 mmol), di-*tert*-butyl 2-thioxoimidazolidine-1,3-dicarboxylate (617 mg, 2.04 mmol), HgCl_2_ (553 mg, 2.04 mmol), and Et_3_N (482 mg, 4.76 mmol) according to method E. The reaction mixture was stirred 30 h at 60 °C. The crude product was purified by centrifugal PTLC using a 2 mm silica plate previously neutralized with *n*-hexane (235 mL) and Et_3_N (15 mL); Petroleum ether/EtOAc was used as the elution system (8:2 → 6:4 → 0:1). **6f** was obtained as a whitish solid (440 mg; 78%). mp > 180 °C. ^1^H NMR (400 MHz, DMSO-*d*_6_): δ 9.67 (s, 1H), 7.54–7.43 (m, 2H), 6.94 (d, *J* = 2.4 Hz, 1H), 6.91 (d, *J* = 1.9 Hz, 1H), 6.87–6.79 (m, 2H), 3.78 (s, 4H), 3.77 (s, 4H), 1.32 (s, 18H), 1.31 (s, 18H). ^13^C NMR (101 MHz, DMSO-*d*_6_): δ 165.1, 158.0, 157.3, 157.0, 155.6, 155.5, 152.50, 152.46, 149.3, 148.4, 130.8, 129.8, 128.0, 118.3, 117.1, 116.4, 106.5, 82.5, 82.4, 45.10, 45.07, 27.8, 27.5. HPLC (UV) > 95%. LRMS (ESI^+^) *m*/*z*: 832.7 [M + H].

##### 5.1.5.6 Di-*tert*-butyl-2-((4-(4-((1,3-bis(*tert*-butoxycarbonyl)imidazolidin-2-ylidene)amino)-2-chlorobenzamido)-2-chlorophenyl)imino)imidazolidine-1,3-dicarboxylate (**6g**)

The reaction was performed with **5g** (300 mg, 1.0 mmol), di-*tert*-butyl 2-thioxoimidazolidine-1,3-dicarboxylate (916 mg, 3.0 mmol), HgCl_2_ (823 mg, 3.0 mmol), and Et_3_N (716 mg, 7.1 mmol) in dry DMF (10 mL) according to method E. The reaction mixture was stirred at 60 °C for 3 days. The crude product was filtered over florisil. The organic phase was concentrated and centrifugal PTLC was performed in a 2 mm silica plate using hexane/EtOAc/MeOH as the elution system (80:20:0 → 0:20:10) to yield the product as a whitish solid (739 mg; 88%). mp 192.4–196.0 °C. ^1^H NMR (400 MHz, DMSO-*d*_6_): δ 10.27 (s, 1H), 7.88 (d, *J* = 1.9 Hz, 1H), 7.42 (d, *J* = 8.2 Hz, 2H), 6.92–6.87 (m, 2H), 6.84 (d, *J* = 8.2 Hz, 1H), 3.78 (br s, 4H), 3.76 (br s, 4H), 1.32 (s, 18H), 1.31 (s, 18H). ^13^C NMR (101 MHz, DMSO-*d*_6_): δ 166.2, 164.7, 151.4, 149.6, 149.4, 141.8, 140.8, 139.7, 134.2, 130.4, 129.5, 129.3, 125.3, 121.3, 120.5, 120.1, 119.3, 118.6, 81.8, 81.7, 43.1, 42.8, 27.5. HPLC (UV) 95%. LRMS (ESI^+^) *m*/*z*: 832.7 [M + H].

##### 5.1.5.7 Di-*tert*-butyl 2-((4-(4-((1,3-bis(*tert*-Butoxycarbonyl)imidazolidin-2-ylidene)amino)-2-isopropoxybenzamido)-2-isopropoxyphenyl)imino)imidazolidine-1,3-dicarboxylate (**6h**)

The reaction was performed with **5h** (150 mg, 0.44 mmol), di-*tert*-butyl 2-thioxoimidazolidine-1,3-dicarboxylate (399 mg, 1.32 mmol), HgCl_2_ (358 mg, 1.32 mmol), and Et_3_N (312 mg, 3.08 mmol) in dry DMF (5 mL) according to method E. The reaction was stirred at 60 °C for 3 days. The crude product was purified by centrifugal PTLC using a silica plate previously neutralized with *n*-hexane (235 mL) and Et_3_N (15 mL). The elution system was prepared with a hexane/EtOAc mixture (7:3 → 4:6). **6h** was obtained as a brownish solid (174 mg; 45%). mp > 93.5 °C. ^1^H NMR (400 MHz, DMSO-*d*_6_): δ 9.82 (s, 1H), 8.20 (d, *J* = 8.6 Hz, 1H), 7.85 (d, *J* = 8.4 Hz, 1H), 6.64 (d, *J* = 1.8 Hz, 1H), 6.59 (d, *J* = 2.2 Hz, 1H), 6.55 (dd, *J* = 8.5, 1.8 Hz, 1H), 6.43 (dd, *J* = 8.6, 2.2 Hz, 1H), 4.71 (hept, *J* = 6.2 Hz, 1H), 4.54 (hept, *J* = 6.2 Hz, 1H), 3.78 (s, 4H), 3.75 (s, 4H), 1.36 (d, *J* = 6.0 Hz, 12H), 1.29 (s, 18H), 1.28 (s, 18H). ^13^C NMR (101 MHz, DMSO-*d*_6_): δ 162.3, 156.2, 153.7, 149.7, 149.4, 147.1, 144.8, 140.6, 139.3, 131.8, 123.5, 120.7, 116.5, 114.5, 113.3, 106.9, 106.5, 81.7, 81.4, 72.5, 71.3, 43.0, 42.9, 27.5, 22.1, 21.9. HPLC (UV) > 95%. LRMS (ESI^+^) *m*/*z*: 880.8 [M + H].

##### 5.1.5.8 Di-*tert*-butyl 2-((4-((4-((1,3-bis(*tert*-Butoxycarbonyl)imidazolidin-2-ylidene)amino)-2-fluorophenyl)carbamoyl)-2-fluorophenyl)imino)imidazolidine-1,3-dicarboxylate (**6i**)

The reaction was performed with **5i** (108 mg, 0.41 mmol), di-*tert*-butyl 2-thioxoimidazolidine-1,3-dicarboxylate (371 mg, 1.23 mmol), HgCl_2_ (334 mg, 1.23 mmol), Et_3_N (0.285 mL, 2 mmol), and anhydrous DMF (3 mL) according to method E. The reaction mixture was stirred for 7 days at room temperature. The mixture was diluted with DMF (10 mL) and filtered on celite. The filter cake was rinsed successively with DMF (20 mL) and CH_2_Cl_2_ (20 mL). The filtrate was evaporated under a vacuum, and the crude yellow oil was partitioned between CH_2_Cl_2_ (60 mL) and water (40 mL). The organic phase was washed with brine, dried (MgSO_4_), and evaporated to give a crude yellow oil. Flash chromatography on neutral alumina eluting with hexane/EtOAc (5:1 → 1:1 → 0:1) yielded **6i** as a grayish solid (67 mg, 20%). ^1^H NMR (300 MHz, chloroform-*d*): δ 8.20 (dd, *J* = 11.0, 6.8 Hz, 1H), 7.81 (d, *J* = 3.4 Hz, 1H), 7.62–7.35 (m, 2H), 7.04 (d, *J* = 8.2 Hz, 1H), 6.80–6.60 (m, 2H), 3.78 (s, 4H), 3.77 (s, 4H), 1.31 (s, 18H), 1.30 (s, 18H). HPLC (UV) 85%. LRMS (ESI^+^) *m*/*z*: 800.6 [M + H].

#### 5.1.6 Synthesis of Boc-Protected Bis(pyridine-2-carboxamidines) (**24c–l**)

##### 5.1.6.1 Method F

A microwave vial was charged with diamine **5c**–**l** (0.4 mmol, 1 equiv, 90–150 mg scale), methyl *N*-(*tert*-butoxycarbonyl)pyridine-2-carbimidothioate^26^ (**21**; 4 equiv), and HgCl_2_ (4 equiv). The vial was sealed with a septum cap and purged with argon. Dry CH_2_Cl_2_ (2–4 mL) was added, followed by dry Et_3_N (4 equiv). The reaction mixture was irradiated for 1 h at 50 °C. The crude reaction mixture was diluted with CH_2_Cl_2_ and filtered through celite and florisil. The filter pad was rinsed successively with CH_2_Cl_2_ and a CH_2_Cl_2_/MeOH (1:1) mixture (50 mL). The solvents were evaporated under a vacuum, and the product was purified by chromatography, as described for each compound.

##### 5.1.6.2 *tert-*Butyl-(*N*,*N*″-(9*H*-fluorene-2,7-diyl)dipicolinimidamide)carbamate (**22**)

The reaction was carried out in a Kimax tube loaded with 9*H*-fluorene-2,7-diamine (270 mg, 1.38 mmol), **21** (870 mg, 3.45 mmol), HgCl_2_ (899 mg, 3.31 mmol), Et_3_N (0.8 mL, 5.52 mmol), and a mixture of dry CH_2_Cl_2_ (5 mL) and dry MeOH (1.5 mL) at room temperature for 7 h. Purification using centrifugal chromatography (silica plates previously deactivated with *n*-hexane/Et_3_N) using *n*-hexane:EtOAc (90:10 → 20:80) gave **22** as a yellowish solid (156 mg, 19%). ^1^H NMR (300 MHz, DMSO-*d*_6_): δ 9.88 (s, 2H), 8.69 (d, *J* = 4.8 Hz, 2H), 8.03 (d, *J* = 1.9 Hz, 2H), 7.99 (dd, *J* = 7.7, 1.7 Hz, 2H), 7.80 (d, *J* = 8.3 Hz, 2H), 7.74–7.64 (m, 4H), 7.57 (dd, *J* = 7.7, 4.8 Hz, 2H), 3.97 (s, 2H), 1.22 (s, 18H). HPLC (UV) = 90%. LRMS (ESI^+^) *m*/*z*: 605 (M + H)^+^.

##### 5.1.6.3 *tert*-Butyl-(-((6-((5-(-*N*′-(*tert*-butoxycarbonyl)picolinimidamido)pyridin-2-yl)carbamoyl)pyridin-3-yl)amino)(pyridin-2-yl)methylene)carbamate (**24c**)

The reaction was performed following the general Method F with diamine **5c** (99 mg, 0.43 mmol), **21** (436 mg, 1.73 mmol), HgCl_2_ (469 mg, 1.73 mmol), and Et_3_N (1.75 mL, 1.73 mmol). Purification by medium pressure chromatography (4g silica cartridge) using hexane/EtOAc (100:0 → 80:20) gave **24c** as a yellowish solid (97 mg; 36%). ^1^H NMR (300 MHz, DMSO-*d*_6_): δ 10.49 (br m, 8H), 9.78 (s, 1H), 8.03–7.88 (m, 2H), 7.81 (d, *J* = 8.5 Hz, 1H), 7.70 (d, *J* = 2.7 Hz, 1H), 7.03 (dd, *J* = 8.7, 2.7 Hz, 2H), 6.16 (s, 1H), 5.14 (s, 1H), 1.20 (m, 18H). ^13^C NMR (126 MHz, DMSO-*d*_6_): δ 161.5, 148.1, 141.7, 141.3, 136.3, 134.4, 133.7, 123.1, 122.6, 119.3, 113.3, 57.7, 8.4. mp 187.6 °C. HPLC (UV) > 95%. LRMS (ESI^+^) *m*/*z*: 638.4 [M + H].

##### 5.1.6.4 *tert*-Butyl-(((4-((4-(-*N*′-(*tert*-butoxycarbonyl)picolinimidamido)-2-chlorophenyl)carbamoyl)-2-chlorophenyl)amino)(pyridin-2-yl)methylene)carbamate (**24d**)

The reaction was performed following the general Method F with diamine **5d** (150 mg, 0.5 mmol), **21** (515 mg, 2 mmol), HgCl_2_ (554 mg, 2 mmol), and Et_3_N (0.3 mL, 2 mmol). The crude solid (800 mg) was purified by reverse phase chromatography with a C18 (12 g) cartridge using H_2_O/CH_3_CN (100:0 → 0:100) as the elution system. The product was obtained as a yellowish solid (159 mg, 44%). ^1^H NMR (500 MHz, DMSO-*d*_6_): δ 10.04 (s, 1H), 9.99 (br s, 2H), 8.70 (ddd, *J* = 4.8, 1.7, 1.0 Hz, 2H), 8.62 (ddd, *J* = 3.6, 1.7, 1.0 Hz, 1H), 8.08–7.98 (m, 3H), 7.96–7.83 (m, 1H), 7.74–7.67 (m, 3H), 7.59 (ddd, *J* = 7.7, 4.8, 1.0 Hz, 2H), 7.50 (d, *J* = 8.7 Hz, 2H), 1.24 (s, 9H), 1.22 (s, 9H). ^13^C NMR (126 MHz, DMSO-*d*_6_): δ 163.9, 160.1, 156.3, 151.4, 151.1, 149.3, 149.1, 148.5, 138.5, 137.2, 130.2, 129.6, 128.9, 128.8, 127.0, 125.6, 125.0, 123.2, 123.1, 120.8, 119.5, 80.4, 79.0, 27.6, 27.5. mp 188.7–211.2 °C. HPLC (UV) > 92%. LRMS (ESI^+^) *m*/*z*: 704.3 [M + H]^+^.

##### 5.1.6.5 *tert*-Butyl-(((4-((4-(*N*′-(*tert*-butoxycarbonyl)picolinimidamido)-3-chlorophenyl)carbamoyl)-2-chlorophenyl)amino)(pyridin-2-yl)methylene)carbamate (**24e**)

The reaction was performed following the general Method F with diamine **5e** (79 mg, 0.3 mmol), **21** (273 mg, 1.1 mmol), HgCl_2_ (293 mg, 1.1 mmol), and Et_3_N (0.2 mL, 1.1 mmol). The crude solid obtained (512 mg) was purified by reverse phase chromatography with a C18 (12 g) cartridge using H_2_O/CH_3_CN (100:0 → 0:100) as the elution system. The product was obtained as a yellowish solid (61 mg, 32%). ^1^H NMR (500 MHz, DMSO-*d*_6_): δ 10.28 (s, 1H), 9.38 (s, 1H), 8.86–8.57 (m, 3H), 8.55–8.39 (m, 1H), 8.19–7.79 (m, 4H), 7.76–7.46 (m, 4H), 7.37–7.21 (m, 1H), 7.18–6.92 (m, 2H), 1.22 (br s, 18H). ^13^C NMR (126 MHz, DMSO-*d*_6_): δ 163.6, 151.1, 148.5, 148.3, 141.6, 137.1, 128.6, 127.0, 125.5, 124.9, 123.1, 121.2, 120.7, 119.2, 80.4, 80.1, 27.6, 27.5. mp 244.1–269.7 °C. HPLC (UV) > 95%. LRMS (ESI^+^) *m*/*z*: 704.3 [M + H]^+^.

##### 5.1.6.6 *tert*-Butyl-(((4-((4-(-*N*′-(*tert*-butoxycarbonyl)picolinimidamido)-2-chlorophenyl)carbamoyl)-3-chlorophenyl)amino)(pyridin-2-yl)methylene)carbamate (**24f**)

The reaction was performed following the general Method F with diamine **5f** (112 mg, 0.38 mmol), **21** (240 mg, 0.95 mmol), HgCl_2_ (248 mg, 0.91 mmol), and Et_3_N (154 mg, 1.5 mmol). The crude solid (372 mg) was purified by circular chromatography using a 2 mm silica plate previously neutralized with *n*-hexane (235 mL) and Et_3_N (15 mL); hexane/EtOAC (6:4 → 4:6) was used as the elution system. The product was obtained as a yellowish solid (181 mg, 67%). ^1^H NMR (300 MHz, DMSO-*d*_6_): δ 10.09 (s, 1H), 9.99 (s, 1H), 9.81 (s, 1H), 8.70 (s, 1H), 8.56 (s, 1H), 8.14–7.92 (m, 4H), 7.79–7.50 (m, 6H), 7.26–7.13 (m, 1H), 7.13–6.99 (m, 1H), 1.24 (d, *J* = 4.1 Hz, 18H). ^13^C NMR (126 MHz, DMSO-*d*_6_): δ 162.3, 160.1, 160.0, 156.3, 156.3, 151.4, 151.1, 149.1, 149.1, 147.1, 139.8, 138.8, 138.2, 137.3, 137.2, 129.8, 129.7, 127.8, 125.7, 125.6, 124.2, 123.4, 123.1, 123.1, 120.9, 119.6, 118.6, 79.2, 79.0, 27.6, 27.6. mp 157.5–170.0 °C. HPLC (UV) > 95%. LRMS (ESI^+^) *m*/*z*: 704.2 [M + H]^+^.

##### 5.1.6.7 *tert*-Butyl-(((4-((4-(-*N*′-(*tert*-butoxycarbonyl)picolinimidamido)-3-chlorophenyl)carbamoyl)-3-chlorophenyl)amino)(pyridin-2-yl)methylene)carbamate (**24g**)

The reaction was performed following the general Method F with diamine **5g** (109 mg, 0.37 mmol), **21** (235 mg, 0.93 mmol), HgCl_2_ (242 mg, 0.89 mmol), and Et_3_N (150 mg, 1.5 mmol). The crude solid (539 mg) was purified by circular chromatography using a 2 mm silica plate previously neutralized with *n*-hexane (235 mL) and Et_3_N (15 mL); CH_2_Cl_2_/EtOAC (95:5 → 90:10) was used as the elution system. The product was obtained as a yellowish solid (32 mg, 12%). ^1^H NMR (300 MHz, DMSO-*d*_6_): δ 10.53 (s, 1H), 10.10 (br s, 2H), 9.58 (m, 1H), 9.37 (m, 1H), 8.75–8.66 (m, 2H), 8.67–8.57 (m, 1H), 8.15–7.93 (m, 3H), 7.85–7.76 (m, 1H), 7.73–7.66 (m, 1H), 7.65–7.52 (m, 3H), 7.06–6.92 (m, 1H), 1.24 (s, 9H), 1.22 (s, 9H). ^13^C NMR (101 MHz, DMSO-*d*_6_): δ 165.7, 160.8, 160.0, 156.3, 153.2, 151.1, 144.8, 141.7, 138.5, 137.3, 133.6, 129.5, 125.7, 123.1, 120.6, 119.9, 118.8, 113.6, 113.1, 80.1, 79.2, 27.51, 27.47. mp > 300 °C. HPLC (UV) > 95%. LRMS (ESI^+^) *m*/*z*: 704.2 [M + H]^+^.

##### 5.1.6.8 *tert-*Butyl-(((4-(4-(-*N*′-(*tert*-butoxycarbonyl)picolinimidamido)-2-isopropoxyphenyl)carbamoyl)-3-isopropoxyphenyl)amino)(pyridin-2-yl)methylene)carbamate (**24h**)

A solution of **5h** (50 mg, 0.15 mmol), **21** (96 mg, 0.38 mmol), and HgCl_2_ (98 mg, 0.36 mmol), in dry CH_2_Cl_2_ (3 mL) was stirred at room temperature, followed by the dropwise addition of Et_3_N (61 mg, 0.6 mmol). The reaction mixture was stirred 6 days at room temperature. The crude was filtered through a pad of florisil rinsing with CH_2_Cl_2_ and MeOH. The filtrate was extracted with H_2_O, washed with brine, and dried over Na_2_SO_4_. The organic phase was concentrated and purified by circular chromatography using a 2 mm silica plate previously neutralized with *n*-hexane (235 mL) and Et_3_N (15 mL); hexane/EtOAc (2:3) was used as the elution system. The product was obtained as a yellowish solid (56 mg, 50%). ^1^H NMR (500 MHz, DMSO-*d*_6_): δ 10.06 (s, 1H), 9.91 (s, 1H), 9.80 (s, 1H), 8.70 (dd, *J* = 9.9, 4.8 Hz, 2H), 8.33 (d, *J* = 8.8 Hz, 1H), 8.10–7.95 (m, 3H), 7.77–7.65 (m, 3H), 7.59 (ddd, *J* = 12.4, 7.9, 5.1 Hz, 3H), 7.48 (s, 1H), 7.37 (d, *J* = 7.5 Hz, 1H), 4.72 (hept, *J* = 6.0 Hz, 1H), 4.59 (hept, *J* = 6.0 Hz, 1H), 1.47 (d, *J* = 6.0 Hz, 6H), 1.39 (d, *J* = 6.0 Hz, 6H), 1.25 (s, 9H), 1.22 (s, 9H). ^13^C NMR (126 MHz, DMSO-*d*_6_): δ 162.3, 160.1, 160.0, 156.3, 156.3, 151.4, 151.1, 149.1, 149.1, 147.1, 139.8, 138.8, 138.2, 137.3, 137.2, 129.8, 129.7, 127.8, 125.7, 125.6, 124.2, 123.4, 123.1, 123.1, 120.9, 119.6, 118.6, 79.2, 79.0, 27.6, 27.6. mp 96.3–102.0 °C. HPLC (UV) > 95%. LRMS (ESI^+^) *m*/*z*: 752.45 [M + H]^+^.

##### 5.1.6.9 *tert*-Butyl ((*E*)-((4-((4-((*E*)-*N*′-(*tert*-Butoxycarbonyl)picolinimidamido)-2-fluorophenyl)carbamoil)-2-fluorophenyl)amino)(pyridin-2-yl)methyleno)carbamate (**24i**)

The reaction was performed following the general Method F with diamine **5i** (51 mg, 0.19 mmol), **21** (195 mg, 0.77 mmol), HgCl_2_ (211 mg, 0.78 mmol), and Et_3_N (107 μL, 0.77 mmol). The crude solid was purified by reverse phase chromatography (12g cartridge, C-18). The unreacted reagents and byproducts eluted first with H_2_O/CH_3_CN: 90/10 → 40/60, whereas compound **24i** eluted with 100% DMSO. Lyophilization yielded **24i** as a yellowish powder (23 mg, 18%). ^1^H NMR (500 MHz, DMSO-*d*_6_): δ 10.09–9.92 (m, 2H), 8.70 (d, *J* = 4. Hz, 1H), 8.59–8.47 (m, 1H), 8.31 (s, 1H), 8.14–8.06 (m, 1H), 8.00 (dd, *J* = 7.8, 1.8 Hz, 1H), 7.92–7.66 (m, 5H), 7.63–7.47 (m, 4H), 7.24–7.05 (m, 1H), 1.24 (s, 18H). LRMS (ESI^+^) *m*/*z*: 672 [M + H]^+^.

##### 5.1.6.10 *tert*-Butyl ((*E*)-((4-((4-((*E*)-*N*′-(*tert*-Butoxycarbonyl)picolinimidamido)-3-fluorophenyl)carbamoyl)-2-fluorophenyl)amino)(pyridin-2-yl)methylene)carbamate (**24j**)

The reaction was performed following the general Method F with diamine **5j** (51 mg, 0.19 mmol), **21** (197 mg, 0.78 mmol), HgCl_2_ (217 mg, 0.8 mmol), and Et_3_N (109 μL, 0.77 mmol). The crude solid was purified by reverse phase chromatography (12g cartridge, C-18). The unreacted reagents and byproducts eluted first with H_2_O/CH_3_CN: 90/10 → 40/60, whereas compound **24j** eluted with 100% DMSO. Lyophilization yielded **24j** as a yellowish powder (28.2 mg, 22%). ^1^H NMR (300 MHz, DMSO-*d*_6_): δ 10.09–9.92 (m, 3H), 8.82–8.44 (m, 2H), 8.14–6.95 (m, 12H), 1.22 (s, 18H). HPLC (UV): > 95%. LRMS (ESI^+^) *m*/*z*: 672 [M + H]^+^.

##### 5.1.6.11 *tert*-Butyl ((*E*)-((4-((4-((E)-*N*′-(*tert*-Butoxycarbonyl)picolinimidamido)-2-fluorophenyl)carbamoyl)-3-fluorophenyl)amino)(pyridin-2-yl)methylene)carbamate (**24k**)

The reaction was performed following the general Method F with diamine **5k** (50 mg, 0.19 mmol), **21** (194 mg, 0.77 mmol), HgCl_2_ (208 mg, 0.8 mmol), and Et_3_N (110 μL, 0.79 mmol). The crude solid was purified by reverse phase chromatography (12g cartridge, C-18). The unreacted reagents and byproducts eluted first with H_2_O/CH_3_CN: 90/10 → 40/60, whereas compound **24k** eluted with 100% DMSO. Lyophilization yielded **24k** as a yellowish powder (48.4 mg, 38%). ^1^H NMR (500 MHz, DMSO-*d*_6_): δ 10.19 (s, 1H), 9.99 (s, 1H), 9.88 (d, *J* = 3,3 Hz, 1H), 8.70 (tt, *J* = 5.5, 1.1 Hz, 2H), 8.21–7.96 (m, 3H), 7.83 (t, *J* = 13.6 Hz, 2H), 7.76 (t, *J* = 8.7 Hz, 1H), 7.73–7,66 (m, 2H), 7.63 (d, *J* = 8.3 Hz, 1H), 7.61–7.53 (m, 2H), 7.52 (d, *J* = 8.3 Hz, 1H), 1.25–1.22 (m, 18H). HPLC (UV): > 95%. LRMS (ESI^+^) *m*/*z*: 672 [M + H].

##### 5.1.6.12 *tert*-Butyl ((*E*)-((4-((4-((*E*)-*N*′-(*tert*-Butoxycarbonyl)picolinimidamido)-3-fluorophenyl)carbamoyl)-3-fluorophenyl)amino)(pyridin-2-yl)methylene)carbamate (**24l**)

The reaction was performed following the general Method F with diamine **5l** (50 mg, 0.19 mmol), **21** (194 mg, 0.77 mmol), HgCl_2_ (214 mg, 0.8 mmol), and Et_3_N (106 μL, 0.79 mmol). The crude solid was purified by reverse phase chromatography (12g cartridge, C-18). The unreacted reagents and byproducts eluted first with H_2_O/CH_3_CN: 90/10 → 40/60, whereas compound **24k** eluted with 100% DMSO. Lyophilization yielded **24k** as a yellowish powder (27.8 mg, 22%). ^1^H NMR (500 MHz, DMSO-*d*_6_): δ 10.56–10.33 (m, 1H), 10.31–10.12 (m, 1H), 9.61–9.39 (m, 1H), 8.72–8.63 (m, 2H), 8.13–7.36 (m, 10H), 7.35–6.45 (m, 1H), 1.26 (s, 9H), 1.22 (d, *J* = 5.7 Hz, 9H). HPLC (UV) > 95%. LRMS (ESI^+^) *m*/*z*: 672 [M + H].

#### 5.1.7 Spectrophotometric p*K*_a_ Measurements

The p*K*_a_ was measured using the 96-well microtiter plate method reported earlier.^51,52^ Briefly, the compounds were dissolved in DMSO to a concentration of 5 mM (stock solution) or less, ensuring that the maximum absorbance of the compound was below 1.5 AU during the assay. Each line of the UV-transparent 96-well microplate (Thermo Scientific Nunc) was loaded with 196 μL of buffer solutions of increasing pH. Then, 4 μL of the compound stock solutions were added to each well with a micropipette (the resulting analyte solution was premixed with the micropipette). One blank solution was prepared for each buffer by adding 4 μL of DMSO to 196 μL of the corresponding buffer solution (i.e., free of analyte compounds) in the well. The 96-well plate was loaded into the UV spectrophotometer (CLARIOStar Plus), incubated at 25 °C, and shaken at 700 rpm for 5 min in a double orbital mode before the reading was performed. UV-spectra scans were recorded between 200 and 600 nm at 2 nm resolution. The raw UV-spectra scans were imported to the Excel template provided in Dardonville et al.,^51^ and the data were processed, as reported.^52,95^ Buffer solutions (pH from 1 to 12) of constant ionic strength (0.1 M KCl) were prepared according to the online buffer calculator: https://www.biomol.net/en/tools/buffercalculator.htm. The number and range of buffer solutions (e.g., every 0.2, 0.5, or 1 pH unit) needed to determine p*K*_a_ values were adjusted depending on the compound tested. In general, a first screening with 12 buffers ranging from 3 to 12 should give an approximate p*K*_a_ value, which can be refined when repeating the experiment using buffers within ±2 pH units of the p*K*_a_ value.

p_s_*K*_a_ values were measured with the Sirius T3 apparatus at 25 °C in a 0.15 M aqueous KCl solution under a nitrogen atmosphere using the UV-metric method, DMSO stock solutions of the samples, and methanol as a cosolvent. The aqueous p*K*_a_ (at 0% co-solvent) was worked out using the Yasuda-Shedlovski extrapolation.

Log *P* values were determined with the Sirius T3 apparatus at 25 °C using the pH-metric method.

#### 5.1.8 Kinetic Solubility Measurements

Ten μL of 10 mM stock solution (DMSO) of the compound (**3a**, **3d**, and **18**) was added to 990 μL of buffer solution (pH 1.2, 5.5, or 7.4) in an Eppendorf tube (DMSO final concentration was 1% (v/v). Blank samples (1% DMSO in buffer) were also prepared. The samples (prepared in triplicate) were shaken at room temperature for 2 h and centrifuged at 135 rpm for 15 min. 170 μL of the supernatant were transferred to a 96-well plate and diluted by adding 30 μL of CH_3_CN. The absorption of the compounds and blank samples was read at 269 nm with a Multiscan Spectrum (Thermo Electron) UV spectrophotometer. The blank contribution was subtracted, and the solubility was worked out from a calibration curve obtained between 0 and 500 μM for each sample. Results are expressed as mean ± SD.

### 5.2 Biophysical Experiments

#### 5.2.1 DNA and Oligonucleotides

Deoxyribonucleic acid sodium salt from salmon testes (i.e., unspecific DNA containing 41.2% GC) employed in LD experiments was purchased from Sigma-Aldrich (ref. D1626). Hairpin oligonucleotides used in the SPR–biosensor assays were acquired from Sigma-Aldrich, with reverse-phase HPLC purification (the loop is underlined): [Biotin]CGAATTCGTCTCCGAATTCG [i.e., (A_2_T_2_)] and [Biotin]CGCGCGCGTTTTCGCGCGCG [i.e., (CG)_4_]. Oligonucleotides used in the thermal melting experiments were purchased from Integrated DNA Technologies (IDT), with HPLC purity: 5′-CATATATATCCCCATATATATG-3′ [i.e., (AT)_4_] and 5′-CGCGCGCGTTTTCGCGCGCG-3′ [i.e., (CG)_4_].

#### 5.2.2 Thermal Melting Experiments

Frozen samples of lyophilized DNA oligonucleotides were removed from the fridge and warmed to room temperature over 5 min. These oligonucleotides were dissolved in 1 mL of 10 mM sodium phosphate buffer (pH 7.0) containing NaCl (100 mM) (oligonucleotide stock solution). The DNA concentration was measured using a Nanodrop 2000 spectrophotometer. CD spectra were recorded on a Jasco 8 J-810 Spectropolarimeter using a 1 mm path-length quartz cuvette. The oligonucleotide (AT)_4_ or (CG)_4_ was diluted at 60 μM concentration in a volume of 200 μL of oligonucleotide stock solution. Test compounds were prepared as 120 μM stock solutions in DMSO. Scans from 320 to 220 nm were performed with a 50 nm/min scanning speed. For each spectrum, an average of three spectra was taken, and the spectrum of the corresponding buffer was subtracted for baseline correction. The melting curves were obtained by recording the change of the molar ellipticity at 270 nm in a range of temperatures from 5 to 85 °C. The temperature was controlled using a Jasco Peltier, with the rate of temperature rising at 40 °C/h. The resulting melting temperatures were calculated by fitting the denaturing curves with the program Origin Pro 6.0.

#### 5.2.3 SPR-Biosensor Assays

The compounds were dissolved in DMSO and diluted to the required concentrations with the buffer containing 10 mM MES, 100 mM NaCl, 1mM EDTA, and surfactant P-20 at 0.005% (v/v) at pH 6.25. SPR binding experiments were performed at 25 °C with a Biacore X100 apparatus (Biacore GE) using the filtered buffer described above. The DNA hairpins were immobilized on a streptavidin-derivatized gold chip (SA chip from Biacore) by injection of a 25 nM hairpin DNA solution with a flow rate of 1 μL/min until ∼400 RU were reached. Flow cell 1 was used as a reference, while flow cell 2 was immobilized with the hairpins in different chips. Direct binding was measured by the injection of increasing concentrations of each compound over the immobilized DNA surfaces at a flow rate of 50 μL/min for a period of 60 s, followed by a dissociation period of 120 s. Regeneration of the surface was made with NaCl 200 mM/NaOH 10 mM using a flow rate of 10 μL/min for 30 s. The binding affinity was determined by fitting the results to a two-site or one-site (*K*_2_ = 0) binding model according to the equation
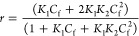
1where *r* is the mole of bound compound per mole of DNA hairpin duplex, *C*_f_ is the free concentration at equilibrium, and *K*_1_ and *K*_2_ the microscopic binding constants.

#### 5.2.4 LD Experiments

The spectra were recorded for natural DNA (salmon testes) titrated with compounds **1a** in phosphate buffer at 25 °C, following the methodology previously reported by Rozas’ group.^47^ Titrations were carried out with a DNA concentration of 378.8 μM working with a Bp/D ratio of 0, 1, and 5, varying the Bp/D ratio from 5 to 1 over 2 additions (Figure 2). Since the experiment was done in a very small volume (70 μL), the concentrations of DNA and the compounds were kept constant to avoid the dilution effect.^47^ Each solution was then prepared individually, and the corresponding spectra were recorded.

### 5.3 Biology

#### 5.3.1 Chemical Compounds

For the biological assays, stock solutions of synthesized compounds were prepared in DMSO as follows: 20 mM for both anti-*T. cruzi* and trichomonacidal experiments; 10 mM for assays against *T. brucei* and *L. donovani*. Amphotericin B (AmB), metronidazole, 3-(4,5-dimethylthiazol-2-yl)-2,5-diphenyltetrazolium bromide (MTT), resazurin, chlorophenol red β-d-galactopyranoside (CPRG), and phorbol 12-myristate 13-acetate (PMA) were purchased from Sigma-Aldrich (St. Louis, MO). l-Glutamine and penicillin/streptomycin were obtained from Gibco. All chemicals were of the highest quality available. Benznidazole was kindly provided by LAFEPE (Laboratório Farmacêutico de Pernambuco, Pernambuco, Brazil). Pentamidine, diminazene, and phenylarsine oxide were purchased from Sigma-Aldrich (St-Louis-MO).

#### 5.3.2 In Vitro Activity against *L. donovani*

*Leishmania donovani* (MHOM/ET/67/HU3) was employed in the screening of synthesized compounds. This line was grown at 28 °C in RPMI 1640-modified medium (Invitrogen) supplemented with 10% heat-inactivated fetal bovine serum (hiFBS) (Invitrogen), as described.^96^

The sensitivity of *Leishmania* promastigotes to the different compounds was determined after incubation for 72 h at 28 °C in the presence of increasing concentrations of the compounds. The concentration of compound required to inhibit 50% of parasite growth (EC_50_) was calculated using the MTT colorimetric assay, as described previously.^97^ For the compound susceptibility analysis of the intracellular amastigote forms of *L. donovani* clinical isolates, stationary-phase promastigotes were used to infect macrophage-differentiated THP-1 cells at a macrophage/parasite ratio of 1:10. After overnight infection at 35 °C with 5% CO_2_ in RPMI 1640 medium plus 5% hiFBS, extracellular parasites were removed by washing twice with PBS buffer. Infected macrophages were incubated with different concentrations of compounds in RPMI 1640 medium plus 10% hiFBS at 37 °C in a 5% CO_2_ atmosphere for 72 h. Following incubation, the medium of the samples was removed and SDS 0.05% was added for around 10 min until lysis of macrophages occurred, liberating intact and viable intracellular amastigotes. We diluted the samples 1/10 with RPMI modified medium supplemented with 10% hiFBS and incubated the samples for 4–7 days at 28 °C in order to let the intracellular amastigotes transform into promastigote forms. Finally, the resazurin colorimetric assay was used to determine the EC_50_ in a similar way, as previously described.^98^ Data are means ± standard deviations from three independent experiments (*n* = 3).

#### 5.3.3 Human Cell Lines Culture and Determination of Cellular Toxicity

The human myelomonocytic cell line THP-1 was grown in RPMI-1640 supplemented with 10% hiFBS, 2 mM glutamate, 100 U/mL penicillin, and 100 μg/mL streptomycin at 37 °C and 5% CO_2_. Five ×10^5^ THP-1 cells per well in 24-well plates were differentiated to macrophages with 20 ng/mL of PMA treatment for 48 h followed by 24 h of culture in complete fresh medium. The cellular toxicity of all compounds was determined using the colorimetric MTT-based assay,^99^ as described for *Leishmania* promastigotes, with the exception of the incubation temperature, which was 37 °C in this case.

#### 5.3.4 In Vitro Activity against *T. brucei*

EC_50_ values were determined for bloodstream forms of both strain Lister 427^100^ and the multidrug-resistant strain B48, which is derived from Lister 427 by knockout of the TbAT1 aminopurine transporter^101^ and in vitro adaptation to high concentrations of pentamidine.^30^ Both strains were cultured in full HMI-9 media (Gibco) supplemented with 10% hiFBS at 37 °C/5% CO_2_, as described.^102^ The drug sensitivity was determined using a resazurin-based assay exactly as described previously in 96 wells, with 23 doubling dilutions and no drug control for each compound. Each well was seeded with 2000 cells and incubated for 70 h with the drug before the addition of the resazurin indicator dye and a further incubation of 24 h (*n* = 3).^20^ Results are expressed as the mean value of EC_50_ ± SEM (standard error of the mean).

#### 5.3.5 Unspecific Cytotoxicity Assays

Cytotoxicity against human embryonic kidney (HEK) cells was determined exactly as described previously.^103^ Briefly, cells were grown in Dulbecco’s modified Eagle’s medium (DMEM; Sigma) supplemented with 10% New-born Calf Serum (Gibco), 1% of a penicillin/streptomycin solution (Gibco), and 1% (vv) of 200 mM glutamax (Gibco) at 37 °C/5% CO_2_. 96-well plates were seeded with 30,000 cells/well in 100 μL medium and incubated for 24 h to allow adhesion, after which an equal value of a drug serial dilution was added and the plates incubated for 30 h prior to the addition of resazurin solution, followed by a final 24 h incubation. Plates were read on a FLUOstar Optima fluorimeter (BMG Labteach, Durham, NC), and the data was analyzed using Prism 8.0 (GraphPad).

#### 5.3.6 *T. cruzi* Epimastigote Susceptibility Assays

The activity profile of the compounds was explored on the *T. cruzi* CL-B5 *lacZ* strain (DTU TcVI) by applying the screening procedure previously described.^104,105^ Briefly, axenic cultures of log-phase epimastigotes in the Liver Infusion Tryptose (LIT) medium were seeded in 96-well microplates at a density of 2.5 × 10^5^ parasites/mL and treated with compounds for 72 h at 28 °C. Stock solutions of either the studied compounds or the reference drug benznidazole were prepared in DMSO and added to the parasite cultures at a final concentration of the solvent lower than 0.2% v/v. Afterward, 50 μL/well of the chromogenic substrate CPRG prepared in 0.9% Triton X-100 (pH 7.4, final concentration 200 μM) was added. After 3 h of incubation at 37 °C, absorbance was read at 595 nm (ELx808 ELISA reader, Biotek Instruments Inc.). Each assay was run in similar conditions three times separately (*n* = 3).

#### 5.3.7 Unspecific Cytotoxicity Assays

The potential toxic effect induced by the studied compounds was investigated on cultures of L929 cells maintained in MEM (Sigma-Aldrich) medium at 37 °C and 5% CO_2._ Accordingly, 10,000 cells/well were distributed in 96-well plates and incubated for 72 h (37 °C, 5% CO_2_) within the studied compounds previously dissolved in DMSO; the final concentration of the solvent in cell cultures was up to 1% v/v, which is not toxic to the cells.^106^ Then, 20 μL of a resazurin solution prepared in PBS (pH 7.0, 2 mM) was added per well. After 3 h of incubation at 37 °C with 5% CO_2_, fluorescence intensity was read at 535 nm (excitation) and 590 nm (emission) (Infinite 200 multifunctional microplate reader, Tecan). Each assay was performed separately three times (*n* = 3).^104,105^

#### 5.3.8 *T. cruzi* Amastigotes Susceptibility Assays

Only those compounds with an activity profile on epimastigotes similar to that of benznidazole were moved to a more specific assay on the intracellular form of the parasite.^59^ First, 10,000 L929 cells/well were seeded in 48-well plates and, after attachment, infected with CL-B5 *lacZ* tissue culture-derived trypomastigotes at a ratio of 1:6 (cell/parasite). After 24 h of incubation at 33 °C with 5% CO_2_, nonpenetrated parasites were rinsed with PBS, and then infected cultures were treated with compounds diluted in fresh MEM for 7 days at the same conditions of temperature and humidity. Finally, 50 μL of CPRG prepared in 3% Triton X-100 (pH 7.4, final concentration 400 μM) was added, and after 3 h of incubation at 37 °C, absorbance was read at 595 nm (Infinite 200 multifunctional microplate reader, Tecan). Each assay was run similarly for three (*n* = 3). Results are expressed as the mean value of EC_50_ ± SD (standard deviation) (SPSS, v20, IBM).

#### 5.3.9 Trichomonacidal Assays

The antiparasitic effect of the compounds was evaluated against the *T. vaginalis* isolate JH31A#4 from the American Type Culture Collection (ATCC). The parasite was cultured in TYM (trypticase-yeast extract-maltose) medium and supplemented with 10% hiFBS and antibiotics. The in vitro screening process was executed following the sequential procedure described previously.^27,107^ The compounds, diluted in DMSO at different concentrations, were added to 1 × 10^5^*T. vaginalis* cells/mL cultures in the exponential growth phase. After 24 h in contact with the parasites at 37 °C and 5% CO_2_, 200 μL of each tube were seeded in sterile 96-well flat-bottomed microplates. Then, culture media was discarded from the plates by centrifugation, and the parasites were subsequently resuspended in 200 μL of sterile phosphate buffered saline (PBS). The antiparasitic activity of each compound was measured by a fluorometric method using the redox dye resazurin. For that, each well was incubated for 1 h in contact with 20 μL of the redox dye (3 mM stock solution in PBS) at 37 °C and 5% CO_2_. Finally, the fluorescence was read in an Infinite 200 Tecan fluorometer at λ_excitation_ 535 nm and λ_emission_ 590 nm. Each plate included a growth control and a positive control in which the reference drug metronidazole was evaluated at its MIC_100_ concentration (24 μM). Each experiment was performed in triplicate and repeated at least two times (*n* ≥ 2).

#### 5.3.10 Unspecific Cytotoxic Assays in the *T. vaginalis* Model

Compounds with trichomonacidal effects (i.e., **3c**, **3e**, **3g**, **3i**, **3j**, **3l**, and **16**) were evaluated at the same concentrations against African green monkey kidney epithelial cells (Vero CCL-81, ATCC). Cells were previously cultured in RPMI-1640 medium supplemented with 10% of hiFBS and antibiotics in a humidified atmosphere at 37 °C and 5% CO_2_. The cytotoxicity experiments were executed as reported previously.^27,104,108^ Briefly, 5 × 10^4^ cells/well were incubated in 96-well flat-bottom microplates for 6 h at 37 °C and 5% CO_2_. Then, the compound was added at the same concentrations used for the susceptibility assays with *T. vaginalis* and incubated with mammalian cells for 24 h at 37 °C and 5% CO_2_. Then, 20 μL of resazurin (1 mM stock solution in PBS) was added per well. After 3 h of incubation, the plates were read in the fluorometer at λ_excitation_ 535 nm and λ_emission_ 590 nm (Infinite 200, Tecan). Each assay was performed at least two times (*n* > 2).^63^

#### 5.3.11 Statistical Analysis

Statistical significance was calculated using the Student’s unpaired-*t* test. Differences were considered significant at a *P* value of <0.05.

## Supporting Information Available

Microsomal stability assays, FID assay, synthesis of starting materials (**4b–l**, **5b–l**, **7a**, **7c–g**, **10**, **11**, and **15**) and **23**, NMR spectra (^1^H and^13^C), and HPLC-MS traces of target compounds (PDF)
Molecular formula strings and activity data (CSV)

## Abbreviations

AmB: amphotericin B
CD: circular dichroism
CPRG: chlorophenol red β-d-galactopyranoside
hiFBS: heat-inactivated fetal bovine serum
HEK cells: human embryonic kidney cells
HMG protein: human mobility group protein
IMHB: intramolecular hydrogen bond
kDNA: kinetoplast DNA
LD: linear dichroism
MGB: minor groove binder
MTT: 3-(4,5-dimethylthiazol-2-yl)-2,5-diphenyltetrazolium bromide
PBS: phosphate buffer saline
PMA: phorbol 12-myristate 13-acetate
SI: selectivity index
SPR: surface plasmon resonance
UGT: uridine glucuronosyl-transferase
