# Supplementary material for: Synthesis and Biophysical and Biological Studies of N-Phenylbenzamide Derivatives Targeting Kinetoplastid Parasites
Source: J Med Chem. 2023 Sep 20;66(19):13452–80. doi: 10.1021/acs.jmedchem.3c00697 (PMC10578353; doi:10.1021/acs.jmedchem.3c00697)
Supplement: Supplementary file 1 — jm3c00697_si_001.pdf [file jm3c00697_si_001.pdf]

Supplementary Material

Synthesis, biophysical and biological studies of *N*-phenylbenzamide derivatives targeting kinetoplastid parasites

J. Jonathan Nué-Martinez[a,b], David Cisneros[a,b], Maria del Valle Moreno Blázquez[a], Cristina Fonseca-Berzal[c], José Ignacio Manzano[d], Damien Kraeutler[a], Marzuq A. Ungogo[e], Maha A. Aloraini[e], Hamza A.A. Elati[e], Alexandra Ibáñez-Escribano[c], Laura Lagartera[a], Tomás Herraiz[f], Francisco Gamarro[d], Harry P. de Koning[e], Alicia Gómez Barrio[c*], Christophe Dardonville[a*]

[a] Instituto de Química Médica, IQM–CSIC, Juan de la Cierva 3, E–28006 Madrid, Spain.

[b] PhD Programme in Medicinal Chemistry, Doctoral School, Universidad Complutense de Madrid (UCM).

[c] Departamento de Microbiología y Parasitología, Facultad de Farmacia, Universidad Complutense de Madrid (UCM), Plaza de Ramón y Cajal s/n, 28040 Madrid, Spain.

[d] Instituto de Parasitología y Biomedicina "López Neyra", IPBLN−CSIC, Parque Tecnológico de Ciencias de la Salud, 18016, Granada, Spain.

[e] Institute of Infection, Immunity and Inflammation, College of Medical, Veterinary and Life Sciences, University of Glasgow, Glasgow, United Kingdom.

[f] Instituto de Ciencia y Tecnología de Alimentos y Nutrición, ICTAN–CSIC, José Antonio Novais 10, Ciudad Universitaria 28040, Madrid, Spain.

Corresponding authors: dardonville@iqm.csic.es; agbarrio@ucm.es

Table of contents **Page**

- Figures S1-S2: SPR binding affinity S2
- Figure S3: Correlation between pK_a and ΔT_m S3
- Table S1 and protocol for microsomal stability assays S3
- Table S2: Fluorescent Intercalator Displacement assay S5
- Table S3: Physicochemical parameters of series 1–3 compounds S7
- Synthesis of starting material **4b**–**l**, **5b**–**l**, **7a**, **7c**–**g**, **10**, **11**, and **15** S9
- Synthesis of **23** using method C2 S21
- NMR spectra and HPLC-MS traces of target compounds S22
- References S66

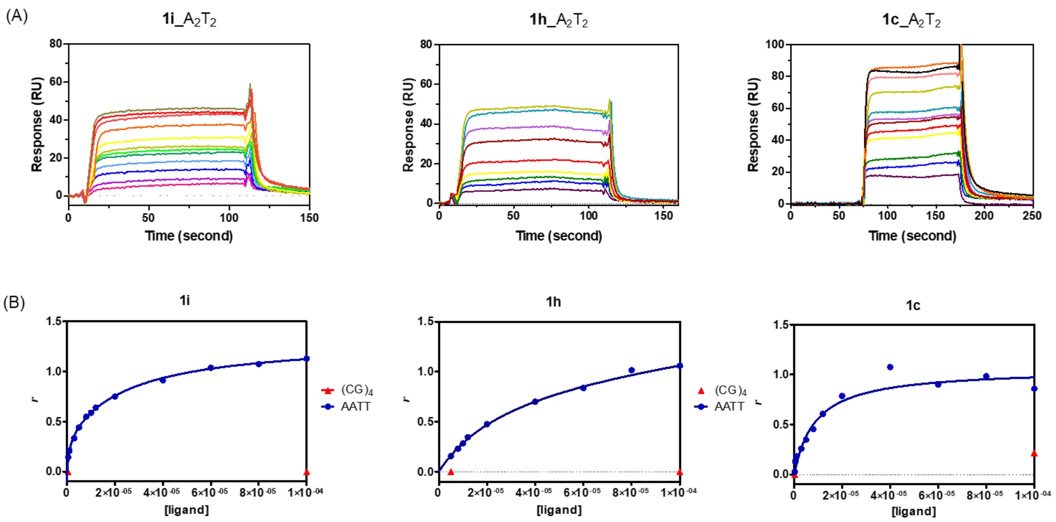

Figure S1. SPR binding affinity: (A) Sensorgrams for binding of **1c**, **1h**, and **1i** to A_2T_2 hairpin duplex using increasing concentrations of the ligand in the range 0.25–100 µM (**1c**), 5–100 µM (**1h**), and 0.5–100 µM (**1i**) (from bottom to top). (B) SPR binding plots of **1c**, **1h**, and **1i** for A_2T_2 and $(CG)_4$ hairpins: the SPR response (RU) at equilibrium (plateau) in the sensorgrams was transformed to *r* and represented against the unbound concentration of the compounds.

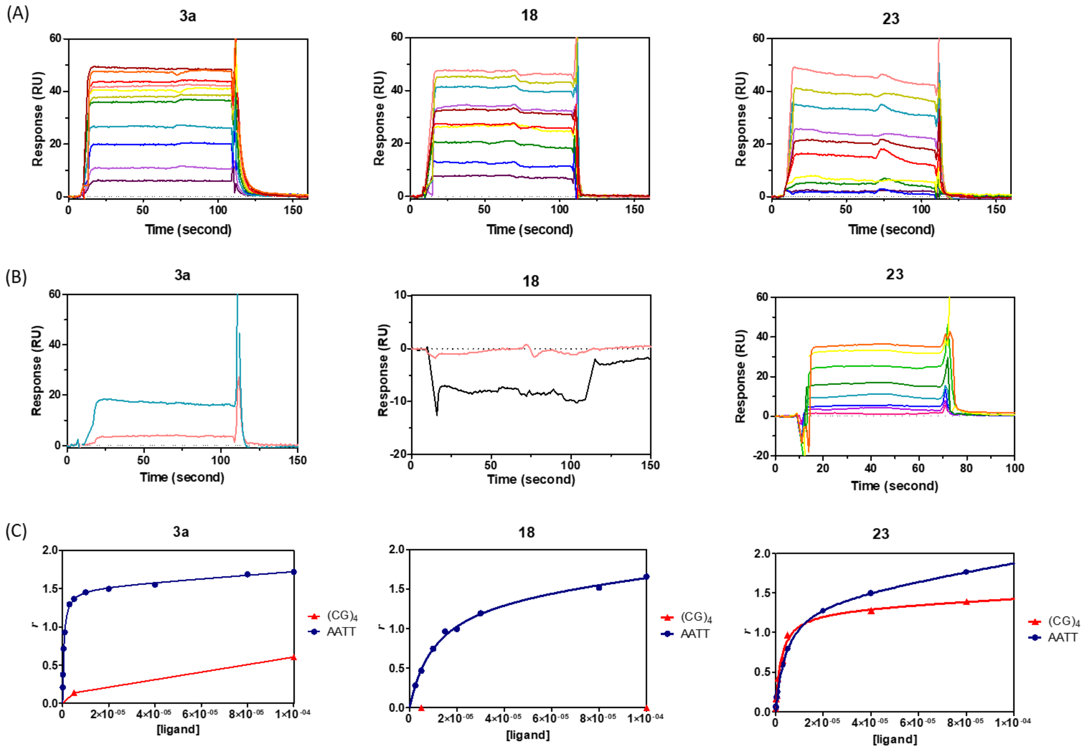

Figure S2. SPR binding affinity: sensorgrams for binding of **3a**, **18** and **23** to A_2T_2 (A) and $(CG)_4$ (B) hairpin duplexes using increasing concentrations of the ligand in the range 0.1–100 µM (**3a**), 2–100 µM (**18**), and 0.1–80 µM (**19**) (from bottom to top). (C) SPR binding plots of **3a**, **18** and **23** for A_2T_2 and $(CG)_4$ hairpins. The SPR response (RU) at equilibrium (plateau) in the sensorgrams was transformed to *r* and represented against the unbound concentration of the compounds.

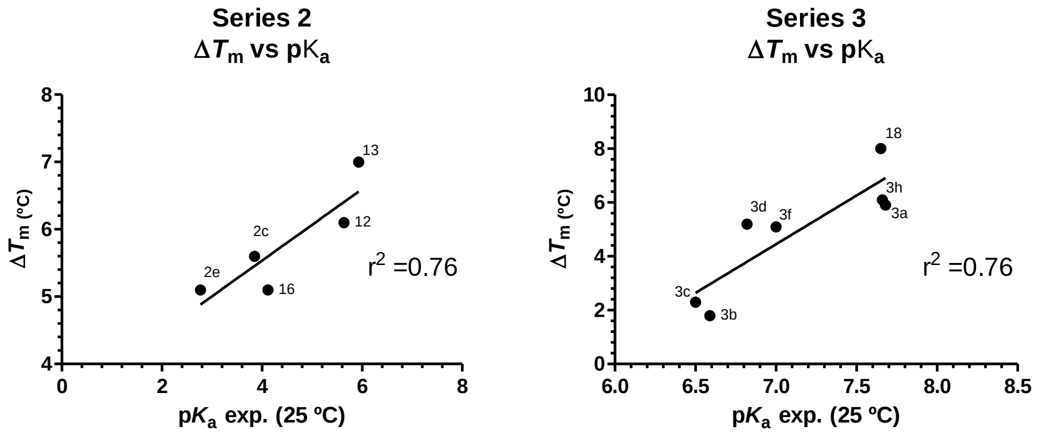

Figure S3. Correlation between pK_a and ΔT_m for series 2 and 3. For consistency, only the high pK_a values measured with the T3 apparatus were considered for each compound.

Microsomal stability. Metabolic stability of **3a** toward metabolism by cytochrome P450 (Phase-I metabolism) and Uridine Glucuronosyl-Transfererase (UGT) (Phase-II metabolism) was studied in presence of the cofactors NADPH and UDPGA, respectively. Incubation media (100–400 µL, final reaction volume) containing 1 mg/mL protein of human liver microsomes (HLM) (Gentest; Corning) or mouse (CD-1) liver microsomes (Sigma), in 80 mM potassium phosphate buffer (pH 7.4) and NADPH (1 mM) were added with **3a** (5 µM, final concentration) or diclofenac (25 µM) and incubated in a water bath at 37 °C during 2 h. On the other hand, incubation media (100 µL of final reaction volume) containing human or mouse liver microsomes (1 mg/mL protein) in 80 mM potassium phosphate buffer (pH 7.4) were added with $MgCl_2$ (4 mM), UDPGA (2 mM), and sonicated for 3 min with the tubes placed in ice bath, and subsequently added with **3a** (5 µM) and incubated at 37 °C for 1 h. The stability in serum was assessed with fractions of 100 µL (final reaction volume) containing 80 mM potassium phosphate buffer with 5 µM of **3a** and 20 µL of human serum (Sigma) and incubated at 37 °C for 1 h. Sampling: aliquots (60-100 µL) were withdrawn at 0, 15, 30, 60 or 120 min and added to 60-100 µL of acetonitrile, vortexed and centrifuged at 10000 rpm. Controls without microsomal fraction and/or cofactors were also carried out. The solution of compound **3a** was prepared in water.

Analysis: An aliquot of supernatant (20 µL) was analyzed by RP-HPLC (Agilent 1100 apparatus with a 1100 diode array detector (DAD) using a reversed phase 3.9 × 150 mm,

4 µm, Nova-pak C18 column (Waters, Milford, MA, USA) under the following chromatographic conditions: eluent A: 50 mM ammonium phosphate buffer (pH 3) and eluent B: 20 % A in acetonitrile. A linear gradient was used from 0 to 32 % B in 8 min, and then 100% B for 12 min. Under these conditions **3a** eluted at 6.5 min. Peak areas of **3a** and diclofenac were determined by absorbance at 280 nm. Assays were carried out at least in duplicate.

Table S1. Microsomal stability of **3a** toward metabolism by cytochrome P450 (Phase-I metabolism) and Uridine Glucuronosyl-Transferase (UGT) (Phase-II metabolism) studied in presence of NADPH and UDPGA.

**Fraction**	**Fase I/II**	**Assay**	Compound **3a** (% remaining)	**Diclofenac** (% remaining)
Microsomal CD-1 Metabolism	P450-NADPH	Control (without fraction)	100	
		Assay (60 min)	92	
Microsomal1a HLM Metabolism	P450-NADPH	Control (without fraction)	100	
		Assay (60 min)	89	
Human liver microsomes (HLM)<br><br>Microsomal stability	P450-NADPH	0 min	100	100.0
		15 min	93.2	48.8
		30 min	94.6	24.5
		60 min	100	7.16
		120 min	93.8	-
Human liver microsomes (HLM)<br><br>Metabolism	UGT-enzymes (UDPGA)	Control (without UDPGA)	100	-
		Assay (60 min)	95.0	-
Mouse liver Microsomes (MLM) CD-1<br><br>Microsomal stability	P450-NADPH	0 min	100	100
		15 min	99.2	87.4
		30 min	94.6	75.1
		60 min	94.8	67.3
		120 min	93.3	-
Mouse liver microsomes (MLM) CD-1<br><br>Metabolism	UGT-enzymes (UDPGA)	Control (without UDPGA)	100	
		Assay (60 min)	91.7	
Human serum (20 uL)		Control (without serum)	100	
		Assay (60 min)	89.1	

Fluorescent Intercalator Displacement (FID) assay. The FID assay was performed following the 96-well single point assay protocol[1] using two hairpin oligonucleotides: 5'-CATATATATCCCCATATATATG-3' [$(AT)_4$] and 5'-CGCGCGCGTTTTCGCGCGCG-3' [$(CG)_4$]. Fluorescence was registered at λ = 595 nm. The fluorescence of the 0% control (ethidium bromide alone) was averaged and subtracted from the fluorescence of each substrate well (i.e., compounds **2a**, **2c**, **2d**, **12**, **13**, **16**, **3a**) and each 100% control well (with DNA but no compound) to normalize the data. For each of sequence, duplicate 100% control wells and triplicates of two concentrations (7.5 and 10 μM) of the compounds of interest were assayed. For each sequence, the % fluorescence (F) was calculated by dividing the median normalized compound fluorescence measurement (at each concentration) by its corresponding averaged normalized 100% measurement.

Table S2A. Remaining fluorescence measured for each compound relative to the reference 100% control (DNA + ethidium bromide).

	**Fluorescence**			
**Compound**	**AT_4**[a]		**$(CG)_4$**[b]	
	7.5 μM	10 μM	7.5 μM	100 μM
**2a**	2.0	2.0	0.29	0[c]
**2c**	4.0	2.0	0.29	0.29
**2d**	3.0	3.0	0.29	0.29
**12**	2.0	2.0	1.29[c]	0.79[c]
**13**	3.0	3.0	2.29[c]	1.29[c]
**16**	3.0[c]	3.0	1.29[c]	1.29[c]
**3a**	4.0	3.5	2.29[c]	2.29[c]

[a] Reference 100% control = 4.0; [b] Reference 100% control = 2.29; [c] n = 2.

Table S2B. Percentage of fluorescence decrease measured for each compound.

	**% F decrease relative to control**			
**Compound**	**AT_4**[a]		**$(CG)_4$**[b]	
	7.5 μM	10 μM	**7.5 μM**	**10 μM**
**2a**	50	50	87.3	100
**2c**	0	50	87.3	87.3
**2d**	25	25	87.3	87.3
**12**	50	50	43.7[c]	65.5[c]
**13**	25	25	0[c]	43.7
**16**	25[c]	25	43.7[c]	43.7[c]
**3a**	0	12.5	0[c]	0[c]

Table S2. Physicochemical parameters of series 1–3 compounds.

Cmpd	$logD_{7.4}$[a]	MW	"Ro5"	Atom count	Non-H atom	Rotable bond	Ring count	Aromatic ring count	Hetero ring count	HBA	HBD	TPSA	Intrinsic Solubility ($logS_0$)[a]
**1a**	N/A	363.4	+	48	27	4	4	2	2	7	5	101.9	-5.00[b]
**1c**	N/A	365.4	+	46	27	4	4	2	2	9	5	126.7	-3.72[b]
**1d**	0.99	432.3	+	48	29	4	4	2	2	7	5	101.9	-4.54
**1e**	1.14	432.3	+	48	29	4	4	2	2	7	5	101.9	-4.54
**1f**	0.91	432.3	+	48	29	4	4	2	2	7	5	101.9	-4.54
**1g**	1.04	432.3	+	48	29	4	4	2	2	7	5	101.9	-4.54
**1h**	0.68	479.6	+	68	35	8	4	2	2	9	5	120.4	-3.93
**1i**	0.20	399.4	+	48	29	4	4	2	2	7	5	101.9	-3.71
**2a**	N/A	459.5	-	56	45	4	6	6	2	7	5	101.9	-8.39[b]
**2c**	N/A	461.5	+	54	35	4	6	6	4	9	5	126.7	-7.11[b]
**2d**	6.01	528.4	-	56	37	4	6	6	2	7	5	101.9	-10.53
**2e**	6.01	528.4	-	56	37	4	6	6	2	7	5	101.9	-10.53
**2f**	6.01	528.4	-	56	37	4	6	6	2	7	5	101.9	-10.53
**2g**	6.01	528.4	-	56	37	4	6	6	2	7	5	101.9	-10.53
**12**	6.24	444.5	-	58	34	5	6	6	2	6	4	72.8	-9.71
**13**	4.85	474.5	-	58	36	4	6	6	2	7	6	114.0	-8.37[b]
**16**	5.47	428.5	-	53	33	2	7	6	2	6	4	72.8	-8.61[b]
**3a**	3.60	435.5	+	54	33	6	4	4	2	7	5	126.6	-5.77 (-5.37)[c]
**3b**	2.98	436.5	+	53	33	6	4	4	3	8	5	139.5	-5.15
**3c**	2.15	437.5	+	52	33	6	4	4	4	9	5	152.4	-4.20

**3d**	4.81	504.4	-	54	35	6	4	4	2	7	5	126.6	-7.02
**3e**	4.82	504.4	-	54	35	6	4	4	2	7	5	126.6	-7.02
**3f**	4.81	504.4	-	54	35	6	4	4	2	7	5	126.6	-7.02
**3g**	4.82	504.4	-	54	35	6	4	4	2	7	5	126.6	-7.02
**3h**	4.83	551.7	-	74	41	10	4	4	2	9	5	145.1	-6.36
**3i**	N/A	471.5	+	54	35	6	4	4	2	7	5	125.6	-7.04[b]
**3j**	N/A	471.5	+	54	35	6	4	4	2	7	5	125.6	-7.04[b]
**3k**	N/A	471.5	+	54	35	6	4	4	2	7	5	125.6	-7.06[b]
**3l**	N/A	471.5	+	54	35	6	4	4	2	7	5	125.6	-7.05[b]
**18**	5.02	420.5	-	56	32	7	4	4	2	6	4	97.5	-6.18
**19**	3.64	450.5	-	56	34	6	4	4	2	7	6	138.7	-5.65
**23**	4.26	404.8	+	51	31	4	5	4	2	6	4	97.5	-6.54

[a] Calculated using ChemAxon package “Chemicalize”. [b] Calculated with ChemDraw 20.0.0.41. [c] Measured at 25 ºC using the SIRIUS T3 apparatus.

1) Synthesis of 4-nitro-*N*-(4-nitrophenyl)benzamides (4b–l)

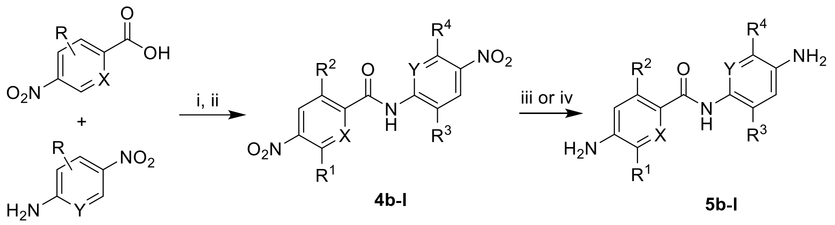

Cmpd	X	Y	R^1	R^2	R^3	R^4
**a**	CH	CH	H	H	H	H
**b**	CH	N	H	H	H	H
**c**	N	N	H	H	H	H
**d**	CH	CH	Cl	H	Cl	H
**e**	CH	CH	Cl	H	H	Cl
**f**	CH	CH	H	Cl	Cl	H
**g**	CH	CH	H	Cl	H	Cl
**h**	CH	CH	H	O*i*-Pr	O*i*-Pr	H
**i**	CH	CH	F	H	F	H
**j**	CH	CH	F	H	H	F
**k**	CH	CH	H	F	F	H
**l**	CH	CH	H	F	H	F

[a]Reagents and conditions: (i) carboxylic acid, $SOCl_2$, 80 ºC (for **4b**, **4f–l**), or $(COCl)_2$, CH_2Cl_2, DMF_{cat}, 0 °C (for **4c**); (ii) aniline, DIPEA, dry toluene, rt; (iii) $SnCl_2.2H_2O$, $HCl_{cat.}$, EtOH, 50 ºC (for **5d–g**); (iv) H_2, Pd-C 5%, EtOAc, rt (for **5b–c**, **5h–l**).

Method G. The reactions were carried out in a round-bottom flask sealed with a screw cap. A solution of carboxylic acid (1.5 equiv., 0.5 – 3 g scale) in $SOCl_2$ (5 – 50 mL) was stirred at 80 °C for the time specified in each case. After cooling back to room temperature, the flask was open carefully because of internal pressure of SO_2 and HCl. The solution was concentrated under vacuum to eliminate excess $SOCl_2$. To facilitate the complete elimination of $SOCl_2$, CH_2Cl_2 was added to the oily residue and the solvent was evaporated again. The acid chloride, which was obtained as oil, was added slowly with a syringe over a stirred solution of nitroaniline (1.0 equiv.) and DIPEA (2.0 equiv.) in anhydrous toluene (20 mL) under Argon atmosphere. The reaction mixture was stirred overnight at room temperature.

Workup 1: The solvent was removed under vacuum and the reaction crude was diluted with MeOH allowing the precipitation of a crude solid. The precipitate was filtrated over a filter funnel and washed successively with 0.1 M aq. HCl (10 mL) and MeOH (20 mL) to give the product as solid powder.

Workup 2: few drops of methanol were added to the reaction mixture to precipitate the product. The solid was isolated by filtration and washed successively with 0.1 M HCl and MeOH to yield the pure benzamides.

2-Isopropoxy-4-nitroaniline. To a pressure round-bottom flask containing a magnetic stir bar was added sequentially 2-amino-5-nitrophenol (1540 mg, 10 mmol), anhydrous *N*,*N*-dimethylformamide (15 mL), K_2CO_3 (1380 mg, 10 mmol), and 2-iodopropane (1.2 mL, 12 mmol). The reaction was refluxed for 48 h. After cooling, the mixture was diluted with distilled water (40 mL) and neutralized with 10 mL of 1 N Na_2CO_3 (until pH = 8). The resulting mixture was extracted with dichloromethane and washed with aqueous saturated sodium chloride solution (brine). The organic layer was dried over magnesium sulphate, filtered, and the solvent was evaporated under vacuum. Column chromatography was performed using hexane/ethyl acetate: 90/10 → 20/80 as elution system, yielding 2-isopropoxy-4-nitroaniline as yellowish oil (1.393 g, 71%). ^{1}H NMR (500 MHz, Methanol-d_4) δ 7.74 (dd, *J* = 8.8, 2.4 Hz, 1H), 7.66 (d, *J* = 2.4 Hz, 1H), 6.68 (d, *J* = 8.8 Hz, 1H), 4.67 (hept, *J* = 6.1 Hz, 1H), 1.38 (d, *J* = 6.1 Hz, 6H). ^{13}C NMR (126 MHz, Methanol-d_4) δ 147.9, 144.4, 138.3, 120.3, 112.5, 109.3, 72.5, 22.1. mp: 58.8–60.1 °C. HPLC (UV) > 95 %. LRMS (ESI$^+$) 197.4 *m/z* [M+H]$^+$.

4-Nitro-*N*-(5-nitropyridin-2-yl)benzamide (4b). A microwave oven glass tube was charged with a solution of 2-amino-5-nitropyridine (566.4 mg; 4.07 mmol; 1.0 equiv.), 4-nitrobenzoyl chloride (1.13 g, 6.11 mmol) and DIPEA (1.05 g; 1.42 mL; 8.14 mmol) in anhydrous toluene (8 mL). The reaction mixture was heated at 100 °C for 30 minutes under Argon atmosphere. Compound **4b** was obtained as whitish solid (930 mg; 80%) after recrystallization from MeOH. ^{1}H NMR (300 MHz, DMSO-d_6) δ 11.90 (s, 1H), 9.25 (d, *J* = 2.2 Hz, 1H), 8.69 (dd, *J* = 9.2, 2.2 Hz, 1H), 8.44 (d, *J* = 9.2 Hz, 1H), 8.35 (d, *J* = 8.7 Hz, 2H), 8.24 (d, *J* = 8.7 Hz, 2H). ^{13}C NMR (75 MHz, DMSO-d_6) δ 165.40, 156.11, 149.58, 144.60, 140.39, 139.13, 134.31, 129.92, 123.45, 113.83. mp: 209–211 ºC. HPLC (UV) > 95%. LRMS (ESI$^-$) *m/z* 287.4 [M-H]$^-$. HRMS (ESI$^-$) *m/z* 287.0432 [M-H]$^-$ (Calc. for $C_{12}H_7N_4O_5$: 287.0422).

5-Nitro-*N*-(5-nitropyridin-2-yl)picolinamide (4c). A suspension of 5-nitropicolinic acid (1.9 g; 11.2 mmol) in dry CH_2Cl_2 (10 mL) was stirred at 0 °C (ice-water bath), followed by the dropwise addition of a catalytic amount of DMF (10 drops). Oxalyl chloride (1.4 g; 1 mL; 11.2 mmol) was added dropwise under argon atmosphere to the stirred mixture, allowing to stir overnight and warming to room temperature. The resulting mixture was cooled to 0 °C (ice-water bath), following by the dropwise addition of Et_3N. A solution of 5-nitropyridin-2-amine (1.1 g; 8 mmol) in dry toluene (15 mL) was

slowly added over the acid chloride. The resulting mixture was stirred for 12 hours at room temperature. The mixture was cooled with an ice-water bath and cold MeOH was added slowly to precipitate product **4c** as greyish solid (1.9 g; 81%). ^{1}H NMR (300 MHz, DMSO-d_6) δ 11.02 (s, 1H), 9.51 (d, *J* = 2.4 Hz, 1H), 9.25 (d, *J* = 2.7 Hz, 1H), 8.86 (dd, *J* = 8.6, 2.4 Hz, 1H), 8.74 (dd, *J* = 9.2, 2.7 Hz, 1H), 8.46 (d, *J* = 9.2 Hz, 1H), 8.43 (d, *J* = 8.6 Hz, 1H). ^{13}C NMR (75 MHz, DMSO-d_6) δ 161.4, 154.5, 152.3, 146.3, 145.0, 144.2, 140.8, 134.8, 134.0, 123.7, 113.1. mp: 238.8 – 241.3 °C. HPLC (UV) > 95 %. LRMS (ESI$^-$) *m/z* 288.3 [M-H]$^-$.

3-Chloro-*N*-(2-chloro-4-nitrophenyl)-4-nitrobenzamide (4d). A mixture of 3-chloro-4-nitrobenzoic acid (850 mg; 1.5 mmol) and $SOCl_2$ (5 mL) reacted according to Method G. The acid chloride was obtained as yellowish oil after 12 hours of reaction. The acid chloride was added over a suspension of 2-chloro-4-nitroaniline (485 mg; 2.8 mmol) and DIPEA (728 mg; 0.98 mL; 5.6 mmol) in dry toluene (20 mL). The resulting mixture was stirred under argon atmosphere for 16 hours at room temperature. After workup 1, **4d** was obtained as brownish solid (760 mg; 76%). ^{1}H NMR (300 MHz, DMSO-d_6) δ 10.73 (s, 1H), 8.44 (d, *J* = 2.6 Hz, 1H), 8.32 (d, *J* = 1.8 Hz, 1H), 8.29 (dd, *J* = 8.9, 2.6 Hz, 1H), 8.27 (d, *J* = 8.5 Hz, 1H), 8.13 (dd, *J* = 8.5, 1.8 Hz, 1H), 8.02 (d, *J* = 8.9 Hz, 1H). ^{13}C NMR (101 MHz, DMSO-d_6) δ 163.2, 149.5, 145.1, 140.7, 138.1, 131.0, 128.7, 128.3, 127.5, 125.9, 125.2, 125.1, 123.0. mp: 188.5 – 193.5 °C. HPLC (UV) > 95 %. LRMS (ESI$^-$) *m/z* 354.3 [M-H]$^-$.

3-Chloro-*N*-(3-chloro-4-nitrophenyl)-4-nitrobenzamide (4e). A mixture of 3-chloro-4-nitrobenzoic acid (3 g; 15 mmol) and $SOCl_2$ (50 mL) reacted according to Method G. The acid chloride, which was obtained as dark yellowish oil after 12 hours of reaction, was added over a suspension of 3-chloro-4-nitroaniline (1.7 g; 10 mmol) and DIPEA (2.6 g; 3.5 mL; 20 mmol) in dry toluene (20 mL). The resulting mixture was stirred under argon atmosphere for 16 hours at room temperature. After workup 2, **4e** was obtained as brownish solid (2.7 g; 77%). ^{1}H NMR (300 MHz, DMSO-d_6) δ 11.09 (s, 1H), 8.32 (d, *J* = 1.8 Hz, 1H), 8.27 (d, *J* = 8.4 Hz, 1H), 8.20 (d, *J* = 2.4 Hz, 1H) 8.20 (d, *J* = 8.9 Hz, 1H), 8.12 (dd, *J* = 8.4, 1.8 Hz, 1H), 7.93 (dd, *J* = 8.9, 2.4 Hz, 1H). ^{13}C NMR (75 MHz, DMSO-d_6) δ 163.5, 149.4, 143.5, 142.2, 138.5, 130.9, 128.3, 127.5, 126.6, 125.9, 125.1, 121.7, 119.0. mp: 244.3 – 245.6 °C. HPLC (UV): > 95 %. LRMS (ESI$^-$): *m/z* 354.3 [M-H]$^-$.

2-Chloro-*N*-(2-chloro-4-nitrophenyl)-4-nitrobenzamide (4f). A mixture of 2-chloro-4-nitrobenzoic acid (1.5 g; 7.5 mmol) and $SOCl_2$ (30 mL) reacted according to Method G. The acid chloride, which was obtained as yellowish oil after 14 hours of reaction, was added over a suspension of 2-chloro-4-nitroaniline (863 mg; 5 mmol) and DIPEA (1.5 g; 11.25 mmol) in dry toluene (10 mL). The resulting mixture was stirred under argon atmosphere for 24 hours at room temperature. After workup 1, **4f** was obtained as brownish solid (1.5 g; 83%). ^{1}H NMR (300 MHz, DMSO-d_6) δ 10.89 (s, 1H), 8.43 (d, J = 2.2 Hz, 1H), 8.41 (d, J = 2.6 Hz, 1H), 8.32 (dd, J = 8.4, 2.2 Hz, 1H), 8.30 (dd, J = 9.0, 2.6 Hz, 1H), 8.22 (d, J = 9.0 Hz, 1H), 7.96 (d, J = 8.4 Hz, 1H). ^{13}C NMR (75 MHz, DMSO-d_6) δ 164.3, 148.6, 144.6, 141.4, 140.2, 131.3, 130.3, 126.8, 125.9, 125.1, 124.6, 123.1, 122.5. mp: 150.7 – 155.7 °C. HPLC (UV): > 95 %. LRMS (ESI$^-$): *m/z* 354.2 [M-H]$^-$.

2-Chloro-*N*-(3-chloro-4-nitrophenyl)-4-nitrobenzamide (4g). A mixture of 2-chloro-4-nitrobenzoic acid (1.7 g; 8.4 mmol) and $SOCl_2$ (20 mL) reacted according to Method G. The acid chloride, which was obtained as yellowish oil after 12 hours of reaction, was added over a suspension of 3-chloro-4-nitroaniline (970 mg; 5.6 mmol) and DIPEA (1.5 g; 2.0 mL; 11.3 mmol) in dry toluene (10 mL). The resulting mixture was stirred under argon atmosphere for 12 hours at room temperature. After workup 2, **4g** was obtained as yellowish solid (1.3 g; 65%). ^{1}H NMR (300 MHz, DMSO-d_6) δ 11.41 (s, 1H), 8.46 (d, J = 2.2 Hz, 1H), 8.34 (dd, J = 8.4, 2.2 Hz, 1H), 8.20 (d, J = 9.0 Hz, 1H), 8.13 (d, J = 2.2 Hz, 1H), 7.98 (d, J = 8.4 Hz, 1H), 7.79 (dd, J = 9.0, 2.2 Hz, 1H). ^{13}C NMR (75 MHz, DMSO-d_6) δ 164.3, 148.8, 143.1, 142.4, 141.2, 131.2, 130.3, 127.7, 126.9, 124.8, 122.7, 121.1, 118.5. mp: 170.7 – 173.2 °C. HPLC (UV): > 95 %. LRMS (ESI$^-$): *m/z* 354.3 [M-H]$^-$.

2-Isopropoxy-*N*-(3-isopropoxy-4-nitrophenyl)-4-nitrobenzamide (4h). A mixture of 2-isopropoxy-4-nitrobenzoic acid[2] (0.5 g, 2.2 mmol) and $SOCl_2$ (5 mL) reacted according to Method G for 12 hours. The acid chloride, which was obtained as yellowish solid, was added over a stirring suspension of 2-isopropoxi-4-nitroaniline (291 mg; 1.5 mmol) and DIPEA (383 mg; 0.5 mL; 3.0 mmol) in dry toluene (10 mL). The resulting reaction mixture was stirred at room temperature under argon atmosphere for 48 hours. After workup 2, **4h** was obtained as yellowish solid after recrystallization from MeOH (410 mg; 69%). ^{1}H NMR (300 MHz, DMSO-d_6) δ 10.07 (s, 1H), 8.65 (d, J = 8.9 Hz, 1H), 8.15

(d, *J* = 8.6 Hz, 1H), 8.00 (d, *J* = 2.2 Hz, 1H), 7.95 (d, *J* = 2.2 Hz, 1H), 7.93 – 7.89 (m, 2H), 5.06 (d, *J* = 6.0 Hz, 1H), 4.95 (d, *J* = 6.0 Hz, 1H), 1.44 (d, *J* = 6.0 Hz, 6H), 1.39 (d, *J* = 6.0 Hz, 6H). ^{13}C NMR (75 MHz, DMSO-d_6) δ 162.8, 156.0, 150.8, 147.0, 143.6, 134.5, 133.1, 128.9, 120.3, 117.2, 116.1, 110.4, 108.5, 74.3, 73.0, 22.0. mp: 179.4 − 180.9 °C. HPLC (UV): > 95 %. LRMS (ESI$^-$): *m/z* 402.6 [M-H]$^-$.

3-fluoro-*N*-(2-fluoro-4-nitrophenyl)-4-nitrobenzamide (4i). A mixture of 3-fluoro-4-nitrobenzoic acid (1.13 g, 6.1 mmol) and $SOCl_2$ (5 mL) reacted according to Method G for 17 h. The acid chloride (610 mg, 3 mmol), which was obtained as yellowish solid, was dissolved in anhydrous THF (2 mL) and added over a stirring suspension of 2-fluoro-4-nitroaniline (312 mg, 2 mmol) and DIPEA (0.7 mL, 4 mmol) in anhydrous THF (5 mL). The resulting reaction mixture was stirred at room temperature under argon atmosphere for 3 days. Workup yielded **4i** as off-white solid (620 mg, 96%). m.p. 204.3 ºC. ^{1}H NMR (300 MHz, DMSO-d_6) δ 11.06 (s, 1H), 8.43 – 8.12 (m, 6H), 8.00 (dd, *J* = 7.0, 1.3 Hz, 1H). ^{13}C NMR (75 MHz, DMSO-d_6) δ 162.1, 158.7 (d, *J* = 253.1 Hz), 152.4 (d, *J* = 250.9 Hz), 149.7 (d, *J* = 9.2 Hz), 143.8 (d, *J* = 8.4 Hz), 132.1 (d, *J* = 11.4 Hz), 131.5 (d, *J* = 3.4 Hz), 129.6 (d, *J* = 15.8 Hz), 123.5, 120.6 (d, *J* = 3.5 Hz), 119.7 (d, *J* = 3.8 Hz), 112.1 (d, *J* = 27.3 Hz), 111.9 (d, *J* = 24.7 Hz).

3-fluoro-*N*-(3-fluoro-4-nitrophenyl)-4-nitrobenzamide (4j). Method G. Compound **4j** was obtained as off-white solid (230 mg, 65%). ^{1}H NMR (300 MHz, DMSO-d_6) δ 11.18 (s, 1H), 8.35 (t, *J* = 8.0 Hz, 1H), 8.25 (t, *J* = 8.9 Hz, 1H), 8.19 – 8.08 (m, 1H), 8.09 – 7.92 (m, 2H), 7.80 – 7.67 (m, 1H).^{13}C NMR (75 MHz, DMSO-d_6) δ 163.6, 155.4 (d, *J* = 259.8 Hz), 154.2 (d, *J* = 262.3 Hz), 145.6 (d, *J* = 11.5 Hz), 140.5 (d, *J* = 7.5 Hz), 138.9 (d, *J* = 7.8 Hz), 131.9 (d, *J* = 7.0 Hz), 127.5, 126.7 (d, *J* = 2.7 Hz), 124.8 (d, *J* = 4.1 Hz), 118.1 (d, *J* = 22.7 Hz), 115.9 (d, *J* = 3.3 Hz), 108.3 (d, *J* = 26.1 Hz).

2-fluoro-*N*-(2-fluoro-4-nitrophenyl)-4-nitrobenzamide (4k). Method G. Compound **4k** was obtained as off-white solid (270 mg, 77%). ^{1}H NMR (300 MHz, DMSO-d_6) δ 11.07 (s, 1H), 8.45–8.12 (m, 5H), 8.00 (dd, *J* = 6.9, 8.5 Hz, 1H). ^{13}C NMR (75 MHz, DMSO-d_6) δ 162.1, 158.7 (d, *J* = 253.3 Hz), 152.3 (*J* = 250.8 Hz), 154.0, 150.7, 149.7 (d, *J* = 9.1 Hz), 143.8 (d, *J* = 8.4 Hz), 132.1 (d, *J* = 11.6 Hz), 131.5 (d, *J* = 3.3 Hz), 129.6 (d, *J* = 15.7 Hz), 123.5, 120.6 (d, *J* = 3.3 Hz), 119.7 (d, *J* = 3.8 Hz), 112.1 (d, *J* = 27.5 Hz), 111.9 (d, *J* = 24.7 Hz).

2-fluoro-*N*-(3-fluoro-4-nitrophenyl)-4-nitrobenzamide (4l). Method G. Compound **4l** was obtained as off-white solid (272 mg, 77%). ^{1}H NMR (300 MHz, DMSO-d_6) δ 11.46 (s, 1H), 8.33 (dd, *J* = 2.2, 9.7 Hz, 1H), 8.0 – 8.17 (m, 2H), 8.07 – 8.00 (m, 1H), 8.00 – 7.90 (m, 1H), 7.64 (dd, *J* = 2.3, 9.0 Hz, 1H). ^{13}C NMR (75 MHz, DMSO-d_6) δ 162.1, 158.7 (d, *J* = 216.2 Hz), 153.8, 149.7 (d, *J* = 8.9 Hz), 145.2 (d, *J* = 11.4 Hz), 132.0 (d, *J* = 6.5 Hz), 131.3, 129.6 (d, *J* = 15.2 Hz), 127.7, 119.8 (d, *J* = 3.8 Hz), 115.5, 112.3 (d, *J* = 27.2 Hz), 107.9 (d, *J* = 26.1 Hz).

2) Synthesis of the diamino precursors (5b–l)

Method H1. Reduction of 4-nitrobenzamides with Tin(II) chloride (5d−g). $SnCl_2.2H_2O$ (10 equiv.) was added to a suspension of nitrobenzamide **4d−g** (1 equiv., 1.0 – 5.5 g scale) in EtOAc (15 – 60 mL) and a catalytic amount of concentrated HCl (20 drops). The reaction mixture was stirred overnight at 50 °C. The reaction mixture was filtered through a pad of Celite and the filter cake was rinsed with CH_2Cl_2:MeOH (1:1) (250 mL). The filtrate was evaporated under vacuum and the crude solid was dissolved in EtOAc and extracted with $NaHCO_3$ (pH = 8). The compound was extracted with EtOAc several times. Complete extraction from the aqueous phase was checked by TLC revealed with ninhydrin. The organic phase was washed with brine, dried with $MgSO_4$, and the solvent was evaporated under vacuum. Recrystallization from EtOAc (**5d, 5e**, **5f**) or $CHCl_3$ (**5g**) gave the product as solid powder.

Method H2. Parr hydrogenation with Pd-C (5b, **5c**, **5h–l)**. A 200 mL Parr hydrogenation flask was charged with the dinitrobenzamide derivative (0.2 – 2.4 g scale) dissolved in EtOAc (15 – 50 mL). The flask was cooled in an ice-bath, and 5% Pd-C catalyst (10% w/w) was added. After air was purged under vacuum, H_2 was introduced into the flask (39 Psi). The hydrogenation process was monitored by TLC using ninhydrin as staining agent. The resulting reaction mixture was diluted with MeOH and filtered through a pad of Celite. The organic phase was evaporated under vacuum to give the crude diamino compound.

4-Amino-*N*-(5-aminopyridin-2-yl)benzamide (5b). Compound **4b** (200 mg, 0.69 mmol) was hydrogenated following Method H2. Recrystallization from EtOAc gave **5b** as brown powder (130 mg, 83%). ^{1}H NMR (300 MHz, DMSO-d_6) δ 9.72 (s, 1H), 7.78 (s,

1H), 7.76 – 7.70 (m, 3H), 6.99 (dd, *J* = 8.7, 2.9 Hz, 1H), 6.55 (d, *J* = 8.6 Hz, 2H), 5.70 (s, 2H), 5.06 (s, 2H). ^{13}C NMR (75 MHz, DMSO-*d*$_6$) δ 164.62, 152.00, 142.50, 141.38, 133.15, 129.30, 122.41, 120.76, 115.63, 112.55. mp: 190–191 ºC. HPLC (UV): 90%. LRMS (ESI$^+$) *m/z* 229.2 [M+H]$^+$. HRMS (ESI$^+$) *m/z* 229.1085 [M+H]$^+$ (Calc. for $C_{12}H_{13}N_4O$: 229.1084).

5-Amino-*N*-(5-aminopyridin-2-yl)picolinamide (5c). Compound **4c** (1.5 g; 5.3 mmol) was hydrogenated following Method H2 in presence of Pd-C 5% (300 mg; 20% w/w). **5c** was obtained by recrystallization from EtOAc as brownish powder (1.2 g; 99.8%). ^{1}H NMR (400 MHz, DMSO-*d*$_6$) δ 9.80 (s, 1H), 7.96 (d, *J* = 2.7 Hz, 1H), 7.94 (d, *J* = 8.7 Hz, 1H), 7.82 (d, *J* = 8.7 Hz, 1H), 7.70 (d, *J* = 2.7 Hz, 1H), 7.03 (dd, *J* = 8.7, 2.7 Hz, 2H), 6.12 (s, 2H), 5.13 (s, 2H). ^{13}C NMR (101 MHz, DMSO-*d*$_6$) δ 161.5, 148.0, 141.7, 141.3, 136.4, 134.4, 133.7, 123.1, 122.7, 119.4, 113.3. mp: 220.7 – 222.9 °C. HPLC (UV) > 95%. LRMS (ESI$^+$): *m/z* 229.1 [M+H]$^+$.

4-Amino-*N*-(4-amino-2-chlorophenyl)-3-chlorobenzamide (5d). Compound **4d** (5.5 g; 15.4 mmol) and $SnCl_2.2H_2O$ (35 g; 154 mmol) reacted following Method H1. **5d** was obtained by recrystallization from EtOAc as a brownish powder (4.1 g; 90%). ^{1}H NMR (300 MHz, DMSO-*d*$_6$) δ 9.40 (s, 1H), 7.86 (d, *J* = 2.0 Hz, 1H), 7.67 (dd, *J* = 8.5, 2.0 Hz, 1H), 7.04 (d, *J* = 8.5 Hz, 1H), 6.81 (d, *J* = 8.5 Hz, 1H), 6.68 (d, *J* = 2.5 Hz, 1H), 6.51 (dd, *J* = 8.5, 2.5 Hz, 1H), 5.95 (br s, 2H), 5.33 (br s, 2H). ^{13}C NMR (75 MHz, DMSO-*d*$_6$) δ 164.3, 148.3, 147.7, 130.7, 129.8, 128.8, 127.7, 123.0, 122.1, 116.1, 114.1, 113.4, 112.6. mp: 174.8 – 179.3 °C. HPLC (UV) > 95%. LRMS (ESI$^+$) *m/z* 296.1 [M+H]$^+$.

4-Amino-*N*-(4-amino-3-chlorophenyl)-3-chlorobenzamide (5e). Compound **4e** (2 g; 5.6 mmol) and $SnCl_2.2H_2O$ (12.7 g; 56.2 mmol) reacted following Method H1. **5e** was obtained by recrystallization from EtOAc as yellowish powder (400 mg; 24%). ^{1}H NMR (400 MHz, Methanol-*d*$_4$) δ 7.84 (d, *J* = 2.1 Hz, 1H), 7.64 (dd, *J* = 8.5, 2.1 Hz, 1H), 7.60 (d, *J* = 2.4 Hz, 1H), 7.27 (dd, *J* = 8.6, 2.4 Hz, 1H), 6.84 (d, *J* = 8.5 Hz, 1H), 6.83 (d, *J* = 8.6 Hz, 1H). ^{13}C NMR (101 MHz, Methanol-*d*$_4$) δ 167.4, 149.2, 142.0, 131.1, 130.1, 128.5, 124.3, 123.8, 122.7, 119.8, 118.7, 117.1, 115.5. mp: 190.0 – 192.5 °C. HPLC (UV) > 95%. LRMS (ESI$^+$) *m/z* 296.1 [M+H]$^+$.

4-Amino-*N*-(4-amino-2-chlorophenyl)-2-chlorobenzamide (5f). Compound **4f** (1.4 g; 4.0 mmol) and $SnCl_2.2H_2O$ (9 g; 40 mmol) reacted following Method H1. **5f** was obtained

by recrystallization from EtOAc as a brownish powder (693 mg; 59%). ^{1}H NMR (400 MHz, DMSO-d_6) δ 9.25 (s, 1H), 7.33 (d, *J* = 8.2 Hz, 1H), 7.16 (d, *J* = 8.4 Hz, 1H), 6.66 (d, *J* = 2.6 Hz, 1H), 6.62 (d, *J* = 2.2 Hz, 1H), 6.56 – 6.47 (m, 2H), 5.75 (s, 2H), 5.32 (s, 2H). ^{13}C NMR (126 MHz, DMSO-d_6) δ 165.3, 151.5, 148.0, 131.5, 130.8, 129.7, 128.8, 122.9, 122.2, 113.8, 113.4, 112.6, 111.6. mp: 180.7 – 183.2 °C. HPLC (UV) > 95%. LRMS (ESI$^+$) *m/z* 296.1 [M+H]$^+$.

4-Amino-*N*-(4-amino-3-chlorophenyl)-2-chlorobenzamide (5g). Compound **4g** (1 g; 2.5 mmol) and $SnCl_2.2H_2O$ (4.75 g; 25 mmol) reacted following Method H1. **5g** was obtained by recrystallization from $CHCl_3$ as yellowish powder (520 mg; 71%). ^{1}H NMR (300 MHz, DMSO-d_6) δ 9.83 (s, 1H), 7.68 (d, *J* = 2.4 Hz, 1H), 7.28 (dd, *J* = 8.8, 2.4 Hz, 1H), 7.23 (d, *J* = 8.4 Hz, 1H), 6.75 (d, *J* = 8.8 Hz, 1H), 6.63 (d, *J* = 2.1 Hz, 1H), 6.53 (dd, *J* = 8.4, 2.1 Hz, 1H), 5.74 (s, 2H), 5.12 (s, 2H). ^{13}C NMR (126 MHz, DMSO-d_6) δ 164.7, 151.3, 140.3, 140.3, 131.3, 130.4, 129.7, 123.1, 120.4, 119.8, 116.8, 115.5, 113.6, 111.6. mp: decomposition at 218.2 °C. HPLC (UV) > 95%. LRMS (ESI$^+$) *m/z* 296.1 [M+H]$^+$.

4-Amino-*N*-(4-amino-2-isopropoxyphenyl)-2-isopropoxybenzamide (5h). Compound **4h** (324 mg, 0.8 mmol) was hydrogenated following Method H2 in presence of Pd-C 5% (65 mg). **5h** was obtained as brownish solid (253 mg, 92%) by recrystallization from EtOAc/Hexane. ^{1}H NMR (300 MHz, DMSO-d_6) δ 9.51 (s, 1H), 7.87 (d, *J* = 8.6 Hz, 1H), 7.72 (d, *J* = 8.5 Hz, 1H), 6.32 (d, *J* = 2.3 Hz, 1H), 6.30 (d, *J* = 2.0 Hz, 1H), 6.23 (dd, *J* = 8.5, 2.0 Hz, 1H), 6.12 (dd, *J* = 8.6, 2.3 Hz, 1H), 5.72 (s, 2H), 4.86 (s, 2H), 4.65 (d, *J* = 6.0 Hz, 1H), 4.49 (d, *J* = 6.0 Hz, 1H), 1.39 (d, *J* = 6.0 Hz, 6H), 1.30 (d, *J* = 6.0 Hz, 6H). ^{13}C NMR (75 MHz, DMSO-d_6) δ 162.3, 157.1, 153.3, 148.0, 145.2, 132.8, 122.6, 118.4, 110.3, 106.8, 105.6, 100.1, 98.4, 71.6, 70.6, 22.2, 22.0. mp > 126.5 ºC. HPLC (UV) > 95 %. LRMS (ESI$^+$) *m/z* 343.3 [M+H]$^+$.

4-amino-*N*-(4-amino-2-fluorophenyl)-3-fluorobenzamide (5i). Compound **4i** (476 mg, 1.47 mmol) was hydrogenated according to Method H2 in presence of Pd-C 5% (86 mg). **5i** was obtained as greyish solid (230 mg, 59%) by recrystallization from EtOAc/MeOH (98/2). ^{1}H NMR (300 MHz, DMSO-d_6) δ 9.32 (s, 1H), 7.59 (m, 2H), 7.01 (t, *J* = 8.7 Hz, 1H), 6.77 (t, *J* = 8.7 Hz, 1H), 6.38 (m, 2H), 5.76 (s, 2H), 5.30 (s, 2H). ^{13}C NMR (75 MHz, DMSO-d_6) δ 164.3, 157.4 (d, *J* = 243.1 Hz), 149.4 (d, *J* = 236.9 Hz), 148.5 (d, *J* = 11.0

Hz), 139.9 (d, J = 13.0 Hz), 128.8 (d, J = 3.5 Hz), 124.7, 121.1 (d, J = 5,5 Hz), 114.7 (d, J = 4.9 Hz), 114.3 (d, J = 19.6 Hz), 113.4 (d, J = 13.5 Hz), 109.3, 100.4 (d, J = 23.0 Hz).

4-amino-*N*-(4-amino-3-fluorophenyl)-3-fluorobenzamide (5j). Compound **4j** (43.1 mg, 0.13 mmol) was hydrogenated according to Method H2 in presence of Pd-C 5%. **5j** was obtained as greyish solid (30.3 mg, 86%) by recrystallization from EtOAc. ^{1}H NMR (300 MHz, DMSO-d_6) δ 9.67 (s, 1H), 7.74 – 7.42 (m, 3H), 7.21 – 7.16 (m, 1H), 6.88 – 6.65 (m, 2H), 5.78 (s, 2H), 4.90 (s, 2H). ^{13}C NMR (75 MHz, DMSO-d_6) δ 163.7, 149.7 (d, J = 235.8 Hz), 148.9 (d, J = 236.1 Hz), 139.9 (d, J = 13.1 Hz), 132.1 (d, J = 13.0 Hz), 128.9 (d, J = 9.5 Hz), 124.7, 121.6 (d, J = 5.5 Hz), 116.8 (d, J = 3.1 Hz), 115.8 (d, J = 5.4 Hz), 114.7 (d, J = 5.0 Hz), 114.2 (d, J = 19.3 Hz), 108.2 (d, J = 23.0 Hz).

4-amino-*N*-(4-amino-2-fluorophenyl)-2-fluorobenzamide (5k). Compound **4k** (200 mg, 0.62 mmol) was hydrogenated according to Method H2 in presence of Pd-C 5% (60 mg) to yield **5k** as greyish solid after recrystallization from EtOAc. ^{1}H NMR (300 MHz, DMSO-d_6) δ 8.82 (d, J = 7.3 Hz, 1H), 7.49 (t, J = 8.8 Hz, 1H), 7.26 (t, J = 8.8 Hz, 1H), 6.46 – 6.27 (m, 4H), 6.00 (s, 2H), 5.28 (s, 2H). ^{13}C NMR (75 MHz, DMSO-d_6) δ 162.3, 161.5 (d, J = 245.5 Hz), 156.2 (d, J = 241.9 Hz), 153.7 (d, J = 12.7 Hz), 147.9 (d, J = 10.8 Hz), 131.9 (d, J = 4.8 Hz), 127.1, 113.9 (d, J = 12.7 Hz), 109.5, 109.2 (d, J = 2.1 Hz), 108.7 (d, J = 12.8 Hz), 100.3 (d, J = 22.8 Hz), 99.2 (d, J = 26.6 Hz).

4-amino-*N*-(4-amino-3-fluorophenyl)-2-fluorobenzamide (5l). Compound **4l** (155 mg, 0.48 mmol) was hydrogenated in presence of Pd-C 5% (50 mg) according to method H2 to yield **5l** as greyish solid (20 mg, 16%) after recrystallization from EtOAc. ^{1}H NMR (300 MHz, DMSO-d_6) δ 9.77 – 9.27 (m, 1H), 7.60 – 7.32 (m, 2H), 7.22 – 7.04 (m, 1H), 6.70 (t, J = 9.6 Hz, 1H), 6.48 – 6.24 (m, 2H), 5.95 (s, 2H), 4.89 (s, 2H). ^{13}C NMR (75 MHz, DMSO-d_6) δ 162.2, 161.0 (d, J = 246.6 Hz), 153.4 (d, J = 12.5 Hz), 149.7 (d, J = 235.7 Hz), 132.1 (d, J = 13.1 Hz), 131.4 (d, J = 4.9 Hz), 128.8 (d, J = 9.6 Hz), 116.4 (d, J = 2.9 Hz), 115.8 (d, J = 5.4 Hz), 110.2 (d, J = 12.6 Hz), 109.4, 107.8 (d, J = 23.2 Hz), 99.3 (d, J = 26.0 Hz).

3) Synthesis of isothiocyanates (7a, 7c–g, 10, 11, and 15)

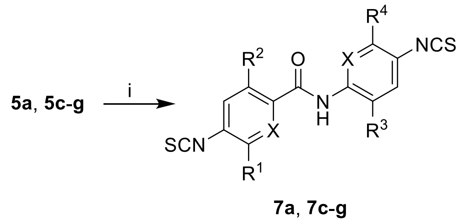

Cmpd	X	Y	R1	R2	R3	R4
**a**	CH	CH	H	H	H	H
**c**	N	N	H	H	H	H
**d**	CH	CH	Cl	H	Cl	H
**e**	CH	CH	Cl	H	H	Cl
**f**	CH	CH	H	Cl	Cl	H
**g**	CH	CH	H	Cl	H	Cl

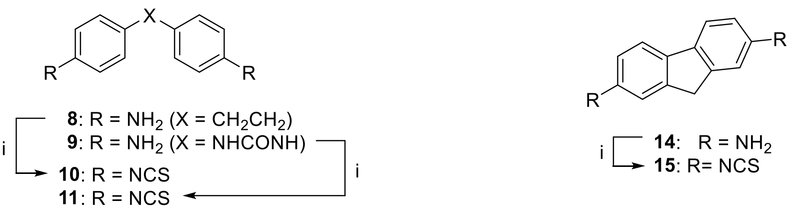

[a]Reagents and conditions. (i) $CSCl_2$, Et_2O:H_2O (3:1), rt (24–92%).

Method I. The reaction was performed in a KIMAX tube. A solution of diamine (**5a**, **5c**–**g**; 1 eq.) and thiophosgene (2.5 eq) in Et_2O:H_2O mixture (3:1) (2 mL) was stirred at room temperature overnight. After stirring a solid appeared at the bottom of the tube. The solid precipitate was filtered in a funnel and washed with water (100 mL). The product was dried under vacuum at 40 ºC to yield the product. **CAUTION!** work in well-ventilated fume-hood and wear adequate protecting clothes. Thiophosgene residues should be disposed off adequately.

4-isothiocyanato-*N*-(4-isothiocyanatophenyl)benzamide (7a). 4-Amino-*N*-(4-aminophenyl)benzamide (**5a**, 3.4 g, 15 mmol) reacted with thiophosgene (2.5 mL, 33 mmol) according to method I to yield **7a** as grey solid (92%). The spectroscopic data were consistent with the literature.[3] ^{1}H NMR (300 MHz, Chloroform–*d*) δ 7.88 (s 1H), 7.85 (d, *J* = 8.7, 2H), 7.63 (d, *J* = 8.8, 2H), 7.31 (d, *J* = 8.7, 2H), 7.22 (d, *J* = 8.8, 2H). Mp. 199–200 °C. HPLC (UV) > 95%. LRMS (ESI$^+$) *m/z* 312.3 (M+H).

5-isothiocyanato-*N*-(5-isothiocyanatopyridin-2-yl)picolinamide (7c). A Kimax tube was loaded with a suspension of **5c** (74.5 mg, 0.32 eq.) in a mixture Et_2O:H_2O 3:1 (2 mL) and thiophosgene (62 µL, 0.81 eq.). The reaction mixture was stirred at room temperature for 4 hours. The brownish precipitate was collected by filtration on a fritted plate and washed with water. Purification by column chromatography on silica was performed using Petroleum ether:EtOAc as elution mixture (100:0 → 50:50) to yield **7c** as brownish solid (52 mg, 52 %). mp: 117.7–122.8 ºC. ^{1}H NMR (300 MHz, DMSO–d_6) δ 10.43 (s,

1H), 8.81 (s, 1H), 8.63 – 8.43 (m, 1H), 8.37 – 8.12 (m, 3H), 8.00 (m, 1H). ^{13}C NMR (75 MHz, DMSO–*d*$_6$) δ 181.4, 172.1, 165.0, 162.2, 153.8, 146.3, 139.9, 136.2, 129.7, 126.3, 124.6, 123.6. HPLC (UV) > 95%. LRMS (ESI$^+$) *m/z* 314.3 (M+H).

3-chloro-*N*-(2-chloro-4-isothiocyanatophenyl)-4-isothiocyanatobenzamide (7d). Compound **5d** (100 mg, 0.34 mmol) reacted with thiophosgene (65 µL, 0.85 mmol) according to method I to yield **7d** as brownish solid (99.6 mg, 44%). mp: 177.4–182.8 ºC. ^{1}H NMR (300 MHz, Chloroform–*d*) δ 8.53 (d, *J* = 8.9 Hz, 1H), 8.31 (s, 1H), 7.98 (s, 1H), 7.74 (d, *J* = 8.3 Hz, 1H), 7.36 (dd, *J* = 8.3, 1.1 Hz, 1H), 7.21 (dd, *J* = 8.9, 1.3 Hz, 1H). HPLC (UV) > 95%. LRMS (ESI$^-$) *m/z* 377. 8 (M-H).

3-chloro-*N*-(3-chloro-4-isothiocyanatophenyl)-4-isothiocyanatobenzamide (7e). Compound **5e** (150 mg, 0.51 mmol) and thiophosgene (0.1 mL; 1.28 mmol) reacted according to method I to yield **7e** was obtained as a whitish solid (160 mg, 82.6%). mp: 186.3–187.6 ºC. ^{1}H NMR (300 MHz, DMSO–*d*$_6$) δ 10.63 (s, 1H), 8.15 (d, *J* = 2.0 Hz, 1H), 8.08 (d, *J* = 2.4 Hz, 1H), 7.94 (dd, *J* = 8.4, 2.0 Hz, 1H), 7.73 (dd, *J* = 8.8, 2.4 Hz, 1H), 7.67 (d, *J* = 8.4 Hz, 1H), 7.51 (d, *J* = 8.8 Hz, 1H). ^{13}C NMR (75 MHz, DMSO–*d*$_6$) δ 163.2, 139.1, 138.7, 136.7, 134.0, 131.4, 130.5, 130.4, 129.2, 128.0, 127.5, 127.3, 123.3, 120.7, 119.7. LRMS (ESI$^-$) *m/z* 377.7 [M-H].

2-chloro-*N*-(2-chloro-4-isothiocyanatophenyl)-4-isothiocyanatobenzamide (7f). Compound **5f** (100 mg, 0.34 mmol) reacted with thiophosgene (98 mg, 65 µL, 0.85 mmol) according to method I. Column chromatography on silica using Petroleum ether: EtOAc (100:0 → 25:75) was performed to yield **7f** as whitish solid (85 mg, 66%). mp: 226.8–228 ºC. ^{1}H NMR (300 MHz, DMSO–*d*$_6$) δ 10.42 (s, 1H), 7.79 (d, *J* = 8.5 Hz, 1H), 7.74 (d, *J* = 2.0 Hz, 1H), 7.73 (d, *J* = 2.4 Hz, 1H), 7.68 (d, *J* = 8.2 Hz, 1H), 7.53 (dd, *J* = 8.2, 2.0 Hz, 1H), 7.48 (dd, *J* = 8.5, 2.4 Hz, 1H). HPLC (UV) > 95%. LRMS (ESI$^-$) *m/z* 377.8 (M-H).

2-chloro-*N*-(3-chloro-4-isothiocyanatophenyl)-4-isothiocyanatobenzamide (7g). Compound **5g** (310 mg, 1.05 mmol) and thiophosgene (0.2 mL; 2.63 mmol) reacted according to method I. **7g** was obtained as a yellowish solid (94 mg, 24%). mp: 115.6–118 ºC. ^{1}H NMR (300 MHz, Chloroform–*d*) δ 8.15 (s, 1H), 7.86 (d, *J* = 2.3 Hz, 1H), 7.74 (d, *J* = 8.2 Hz, 1H), 7.45 (dd, *J* = 8.6, 2.3 Hz, 1H), 7.28 (d, *J* = 2.0 Hz, 1H), 7.22 (d, *J* = 8.6 Hz, 1 H), 7.20 (dd, *J* = 8.2, 2.0 Hz, 1 H). ^{13}C NMR (75 MHz, Chloroform–*d*) δ 163.3,

140.1, 139.1, 136.8, 135.4, 132.62, 132.59, 132.0, 131.8, 127.4, 127.1, 126.3, 124.8, 121.5, 119.1. HPLC (UV) > 95%. LRMS (ESI^-) *m/z* 377.8 (M-H).

1,2-bis(4-isothiocyanatophenyl)ethane (10). 4,4'-Diaminobibenzyl (1.28 g, 6 mmol) reacted with thiophosgene (1.73 g, 1.15 mL, 15 mmol) according to method I. Column chromatography on silica using *n*-hexane:EtOAc (100:0 → 80:20) was performed to yield **10** as whitish solid (1.4 g, 79%). The spectroscopic data are consistent with the literature.[3] ^{1}H NMR (400 MHz, Chloroform–*d*) δ 7.12 (d, *J* = 8.6 Hz, 4H), 7.07 (d, *J* = 8.6 Hz, 4H), 2.89 (s, 4H). ^{13}C NMR (101 MHz, Chloroform–*d*) δ 140.6, 135.1, 129.8, 129.3, 125.8, 37.3. HPLC (UV) > 95%. LRMS (ESI^+) *m/z* 297.3 (M+H).

1,3-bis(4-isothiocyanatophenyl)urea (11). A suspension of 1,3-bis(4-aminophenyl)urea[3] (201 mg, 0.83 mmol.) in Et_2O:H_2O (3:1) (2 mL) was stirred at room temperature, followed by the addition of thiophosgene (0.16 mL, 2.08 mmol). The resulting mixture was stirred overnight. The precipitate was filtered over a fritted plate and washed with water to yield **11** as whitish solid (174 mg: 64%). The spectroscopic data are consistent with the literature.[3] mp > 300 ºC. ^{1}H NMR (400 MHz, DMSO–d_6) δ 9.03 (s, 2H), 7.52 (d, *J* = 8.9 Hz, 4H), 7.37 (d, *J* = 8.9 Hz, 4H). ^{13}C NMR (101 MHz, DMSO–d_6) δ 152.1, 139.2, 132.2, 126.7, 123.2, 119.0. HPLC (UV) > 95%. LRMS (ESI^+) *m/z* 314.3 (M+H).

2,7-diisothiocyanato-9*H*-fluorene (15). 2,7-Diaminofluorene (137 mg, 0.7 mmol) reacted with thiophosgene (201 mg: 130 µL, 1.75 mmol) according to method I to yield **15** as brownish solid (169 mg; 86%). mp: decomposes at 213.6 ºC. ^{1}H NMR (400 MHz, DMSO–d_6) δ 8.01 (d, *J* = 8.1 Hz, 2H), 7.69 (d, *J* = 2.0 Hz, 2H), 7.49 (dd, *J* = 8.1, 2.0 Hz, 2H), 3.98 (s, 2H). ^{13}C NMR (101 MHz, DMSO–d_6) δ 145.1, 139.6, 133.4, 128.7, 125.1, 123.0, 121.7, 36.4. HPLC (UV) > 95%. LRMS (ESI^-) *m/z* 279.4 (M-H).

4) Synthesis of *N,N''*-(9*H*-fluorene-2,7-diyl)dipicolinimidamide (23) using method C2.

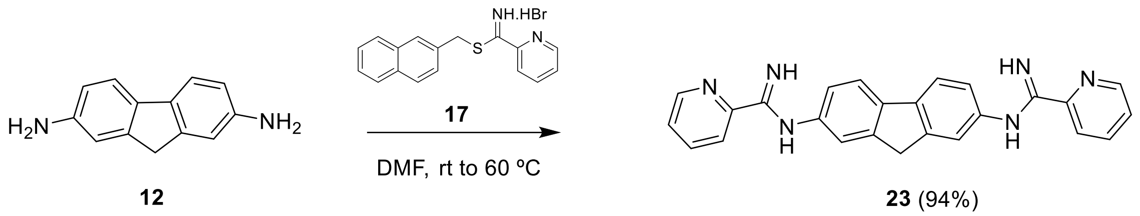

9H-fluorene-2,7-diamine (24.7 mg, 0.13 mmol) and **17** (180.9 mg, 0.50 mmol) were dissolved in 6 ml of anhydrous DMF in a KIMAX sealed tube. The reaction mixture was stirred under argon atmosphere over 4h at room temperature. Then, the reaction was heated at 60 ºC for one day. Water (10 mL) was added and the aqueous layer was extracted with EtOAc and CH_2Cl_2 to remove the by-products and starting material. The aqueous layer was concentrated *in vacuo* to yield a brownish oil. Hexane was added and the precipitate was filtered over a Buchner funnel. The precipitate was re-dissolved in water and a precipitate was formed by the addition of a saturated aqueous solution of $NaHCO_3$. The precipitate was collected and dried to give a yellow powder (48.0 mg; 94%). m.p: 210.2-211.4ºC. LRMS (ESI$^+$) *m/z* 405 [M+H]$^+$. ^{1}H NMR (400 MHz, DMSO-d_6) δ 8.65 (d, *J* = 4.8 Hz, 2H), 8.35 (d, *J* = 7.9 Hz, 2H), 7.97 (t, *J* = 7.7 Hz, 2H), 7.77 (d, *J* = 8.0 Hz, 2H), 7.56 (dd, *J* = 7.5, 4.8 Hz, 2H), 7.16 (s, 2H), 6.96 (d, *J* = 8.0 Hz, 2H), 6.76 (bs, 2H), 3.87 (s, 2H). ^{13}C NMR (101 MHz, DMSO-d_6) δ 152.0, 151.5, 148.1, 147.9, 143.9, 137.1, 136.2, 125.4, 121.3, 120.5, 119.9, 118.5, 36.6.

5) NMR spectra and HPLC-MS traces of target compounds

Compound **1c**

^{1}H NMR (400 MHz, DMSO-d_6)

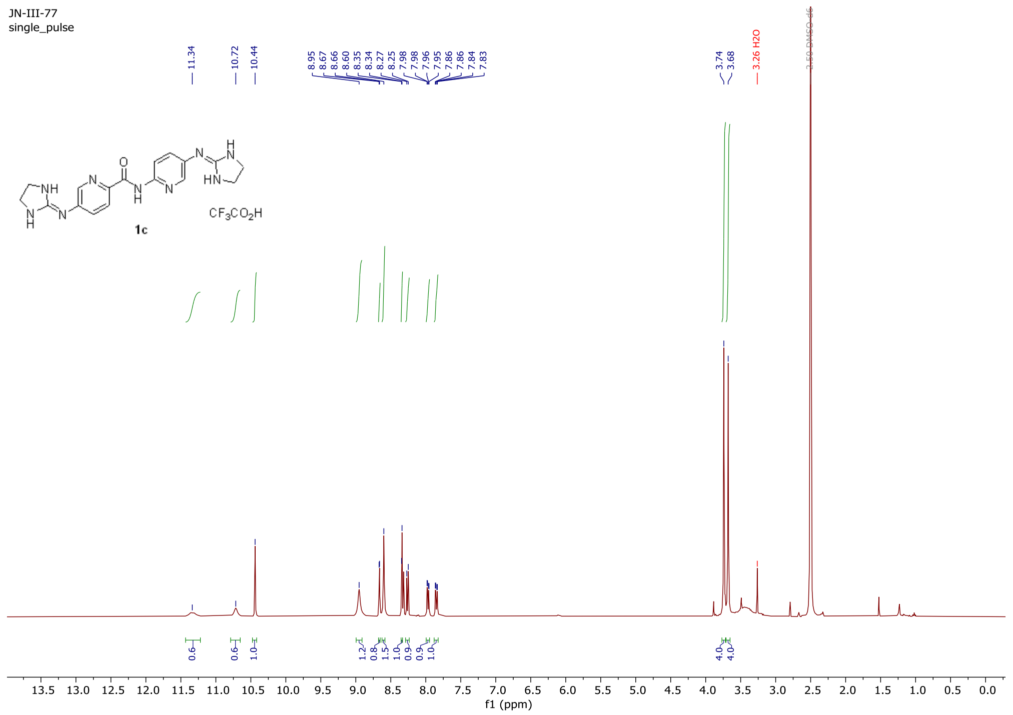

^{13}C NMR (101 MHz, DMSO-d_6)

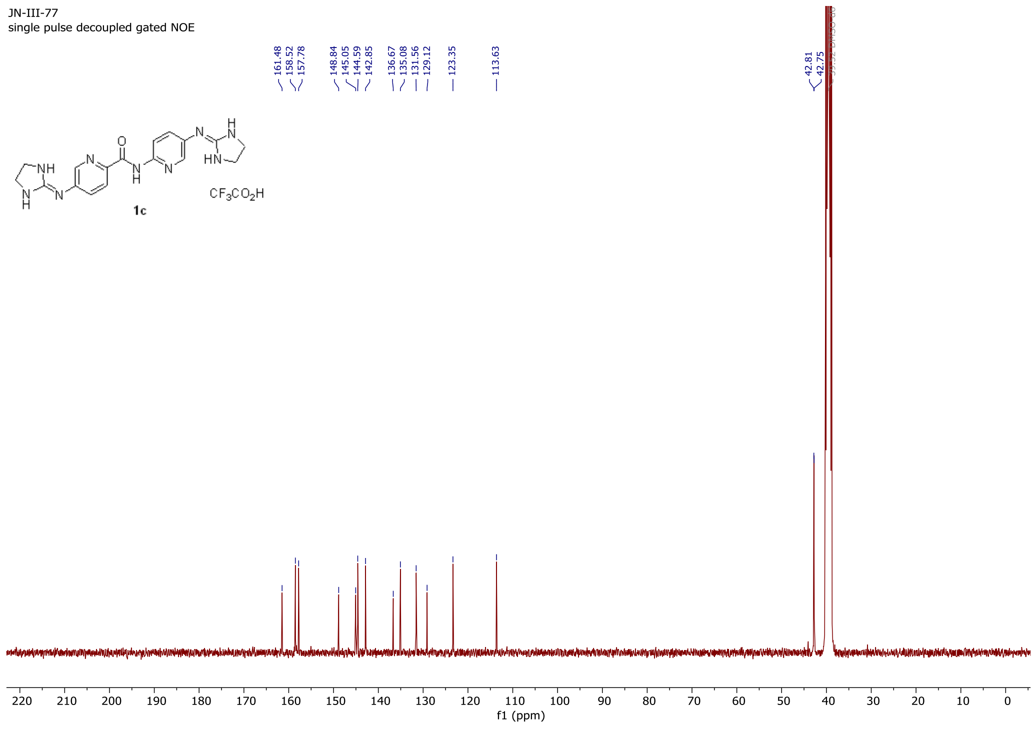

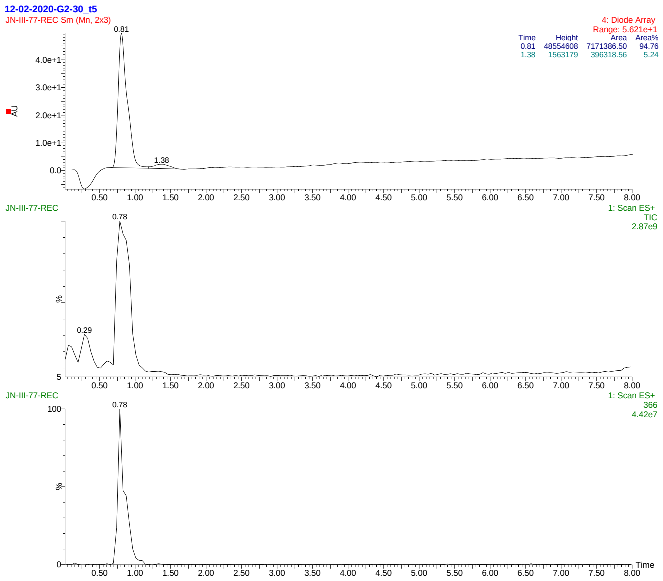

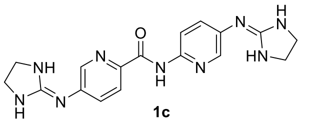

1c

Compound **1d**

^{1}H NMR (400 MHz, DMSO-d_6)

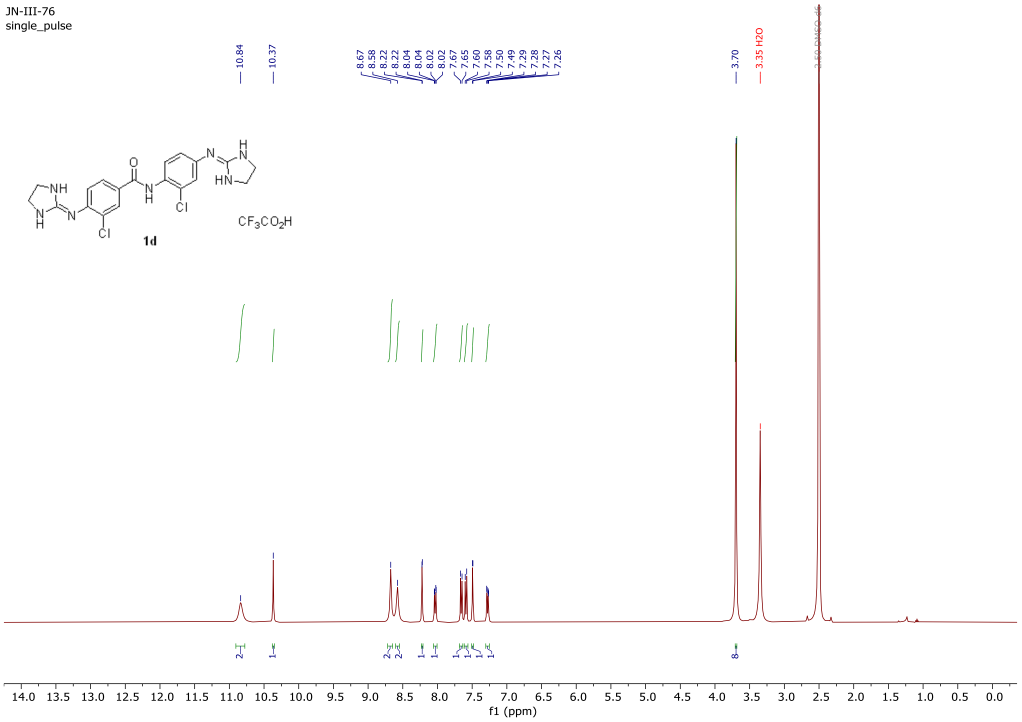

^{13}C NMR (101 MHz, DMSO-d_6)

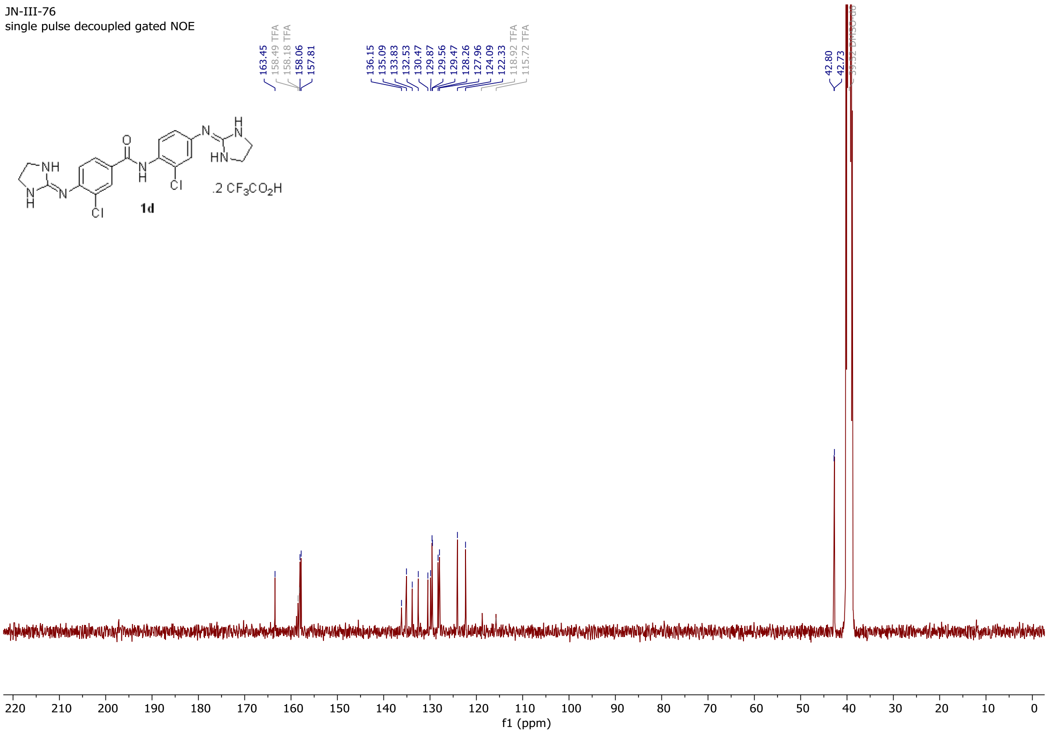

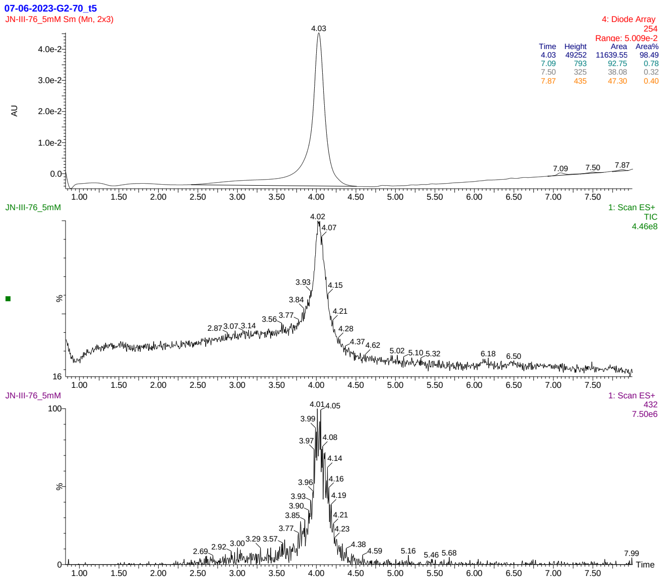

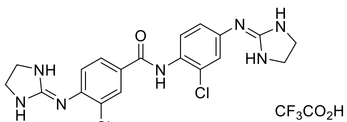

1d CF$_3$CO$_2$H

Compound **1e**

^{1}H NMR (500 MHz, DMSO-d_6)

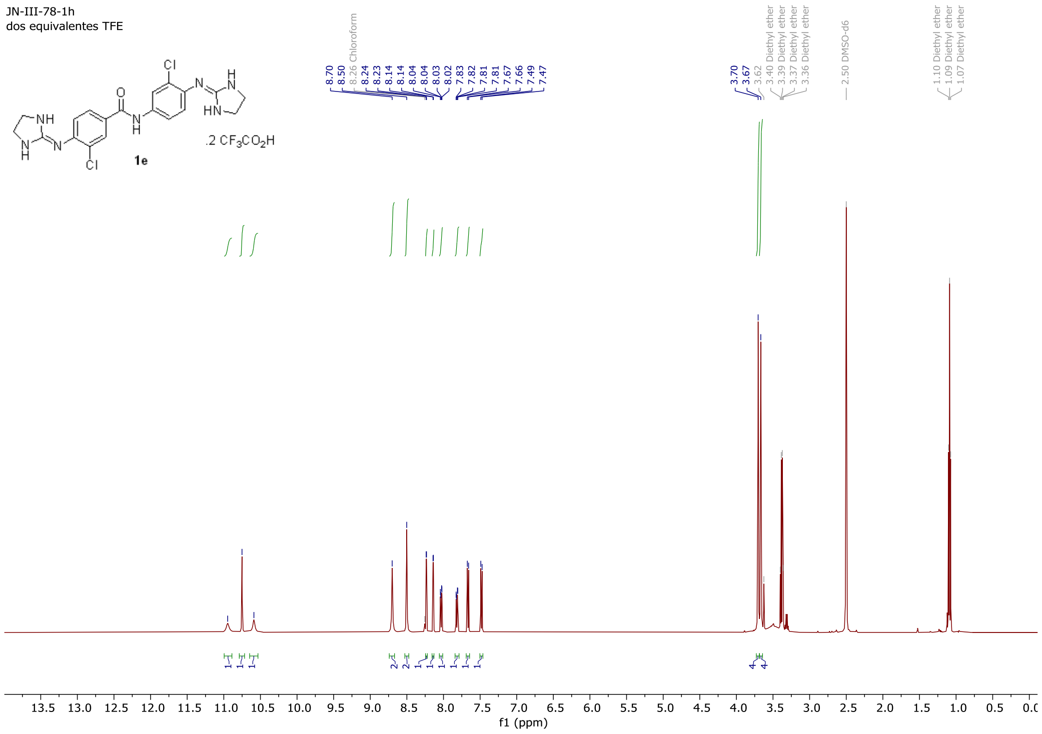

^{13}C NMR (126 MHz, DMSO-d_6)

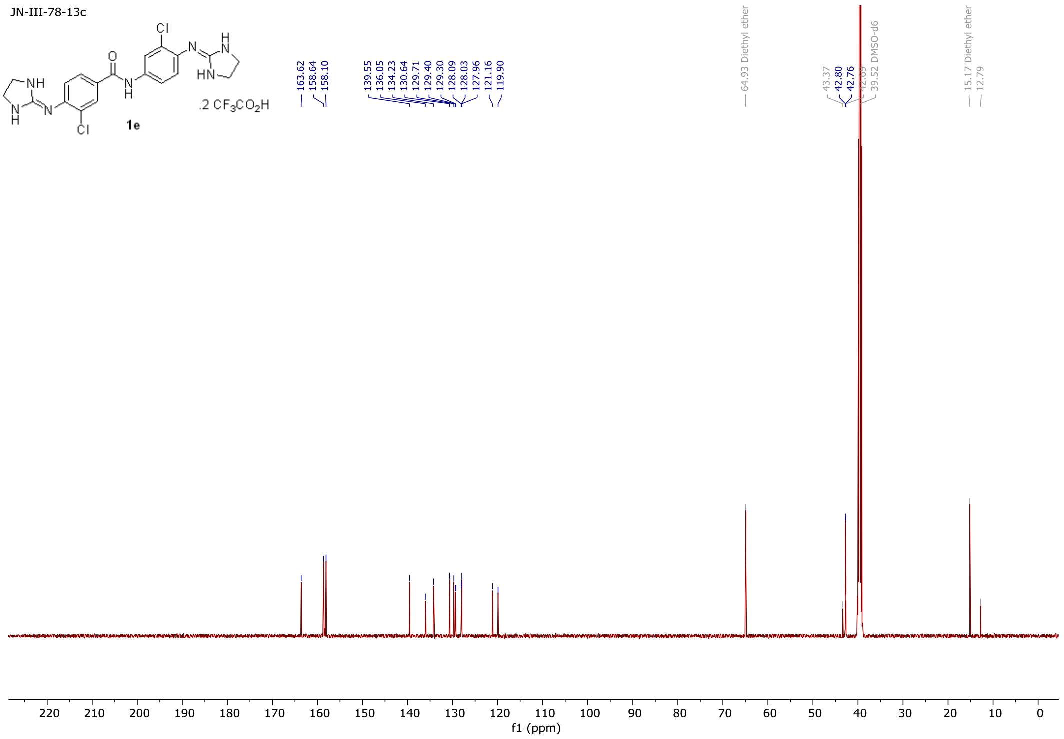

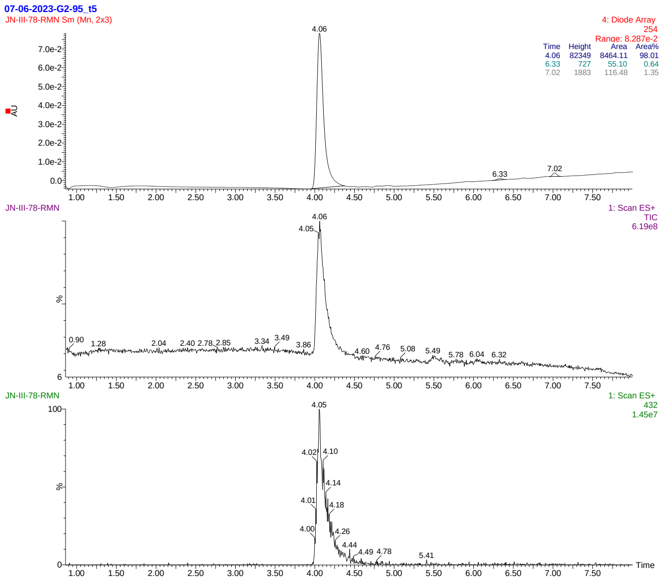

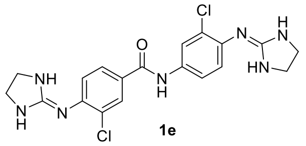

1e

Compound **1f**

^{1}H NMR (500 MHz, DMSO-d_6)

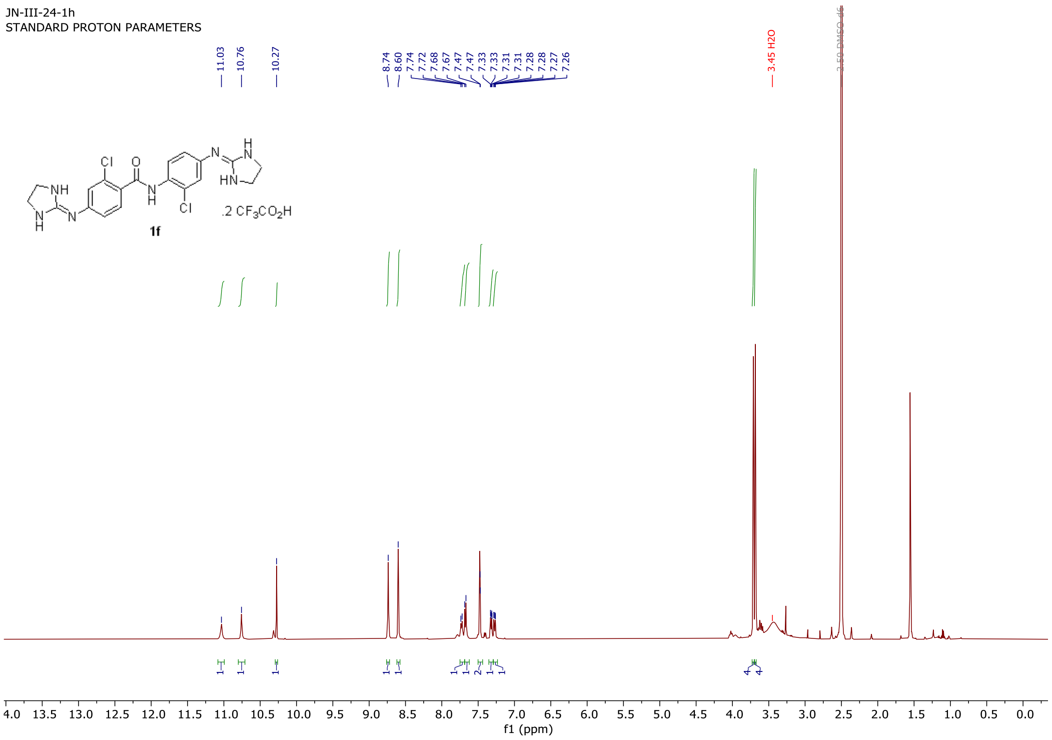

^{13}C NMR (126 MHz, DMSO-d_6)

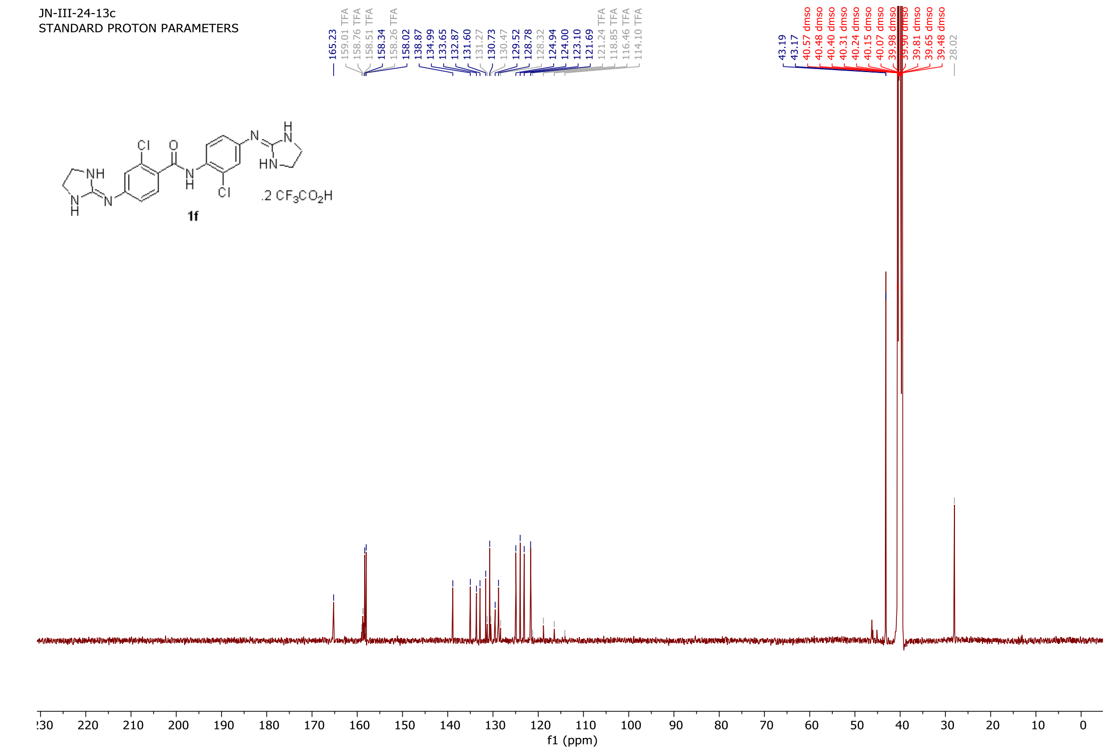

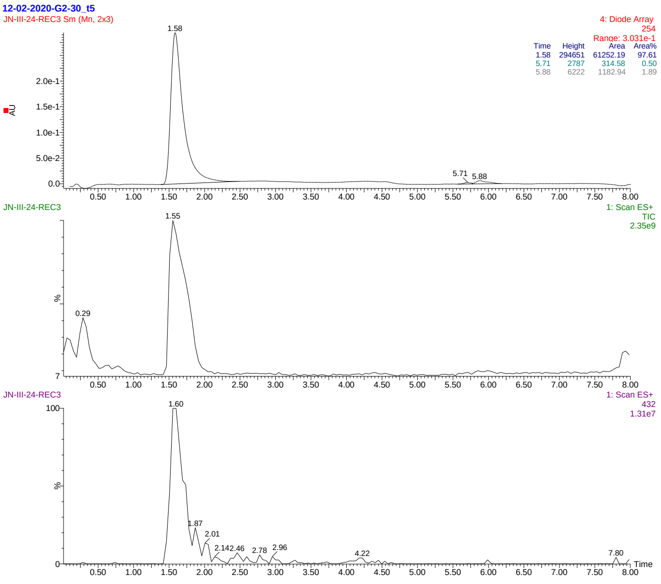

Time	Height	Area	Area%
1.58	294651	61252.19	97.61
5.71	2787	314.58	0.50
5.88	6222	1182.94	1.89

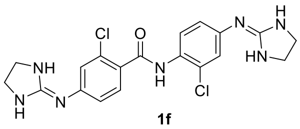

1f

Compound **1g**

^{1}H NMR (500 MHz, DMSO-d_6)

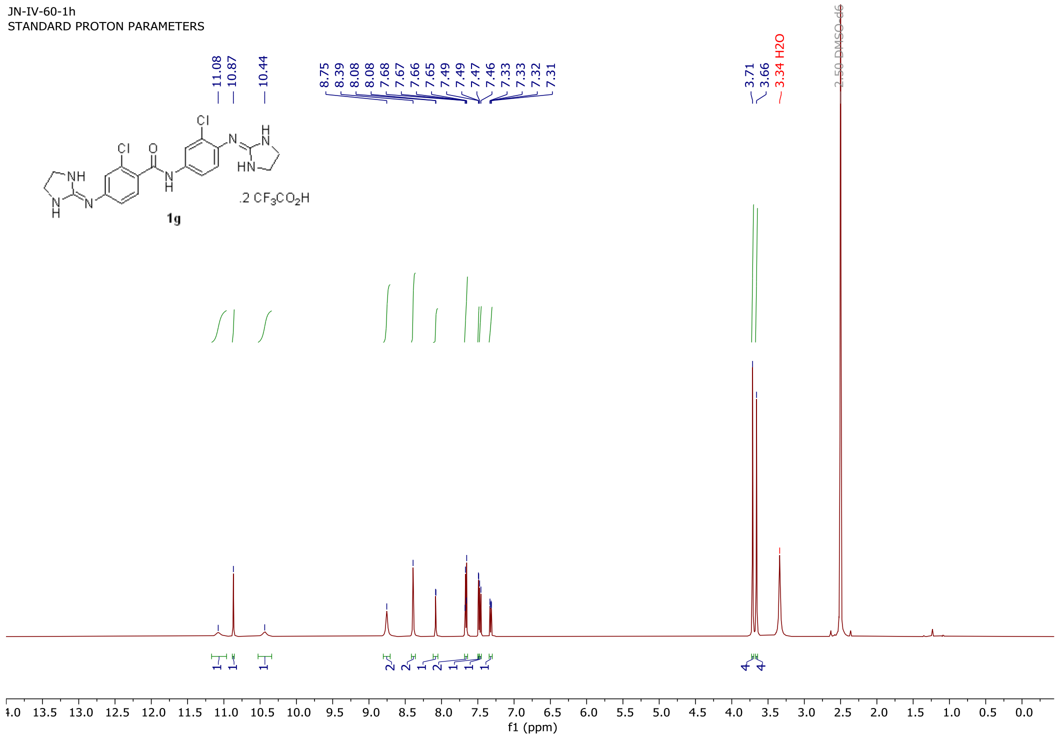

^{13}C NMR (126 MHz, DMSO-d_6)

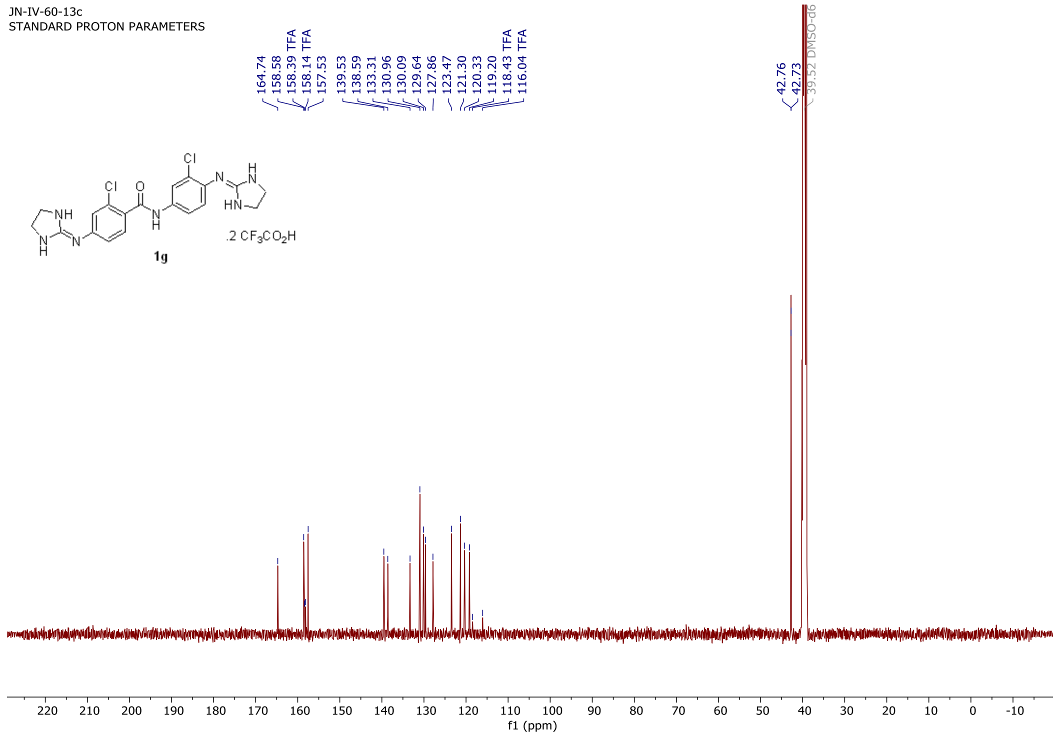

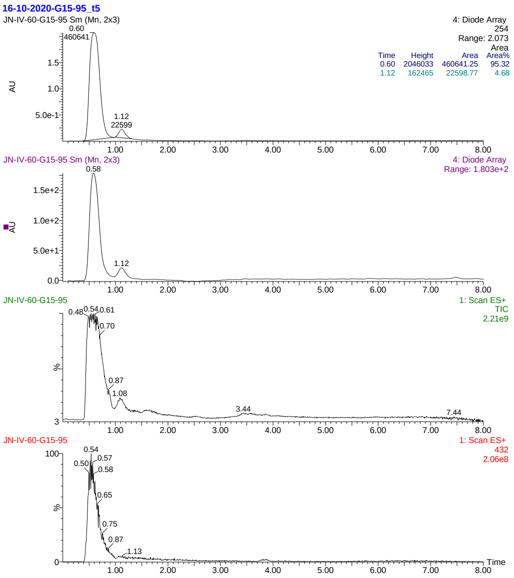

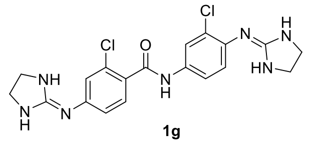

1g

Compound **1h**

^{1}H NMR (500 MHz, DMSO-d_6)

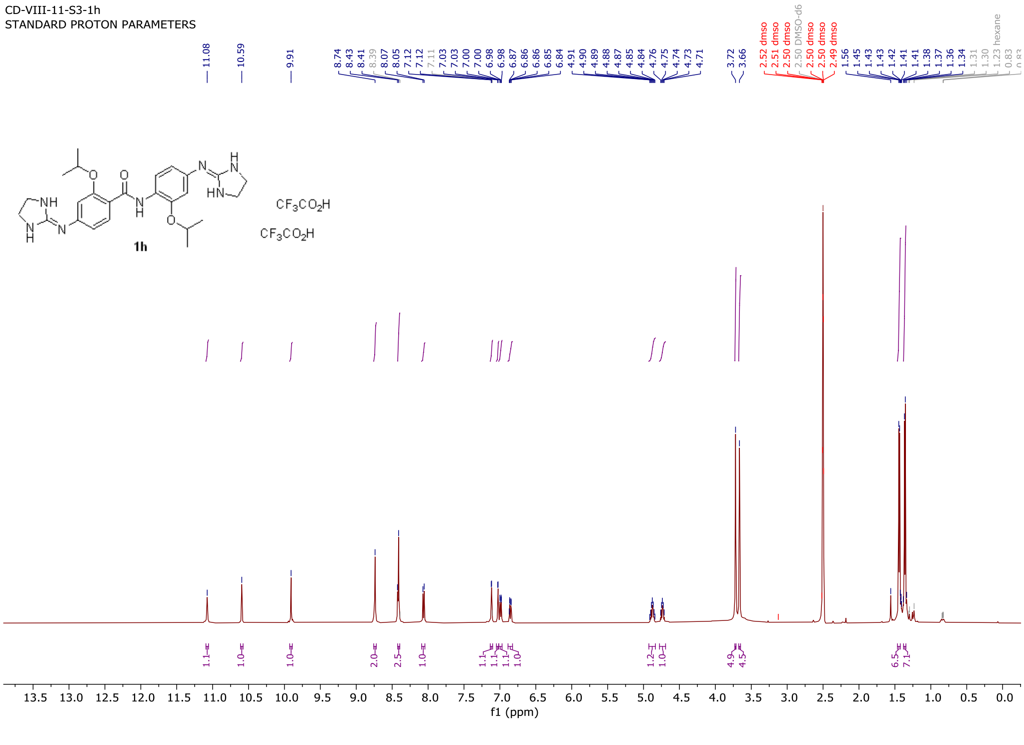

^{13}C NMR (126 MHz, DMSO-d_6)

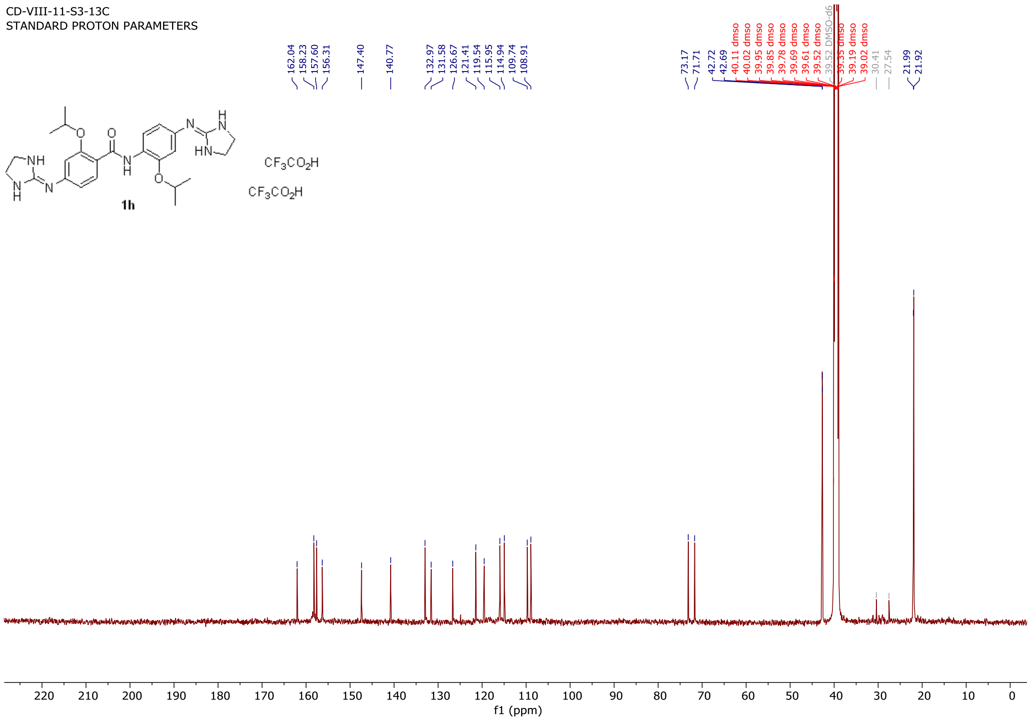

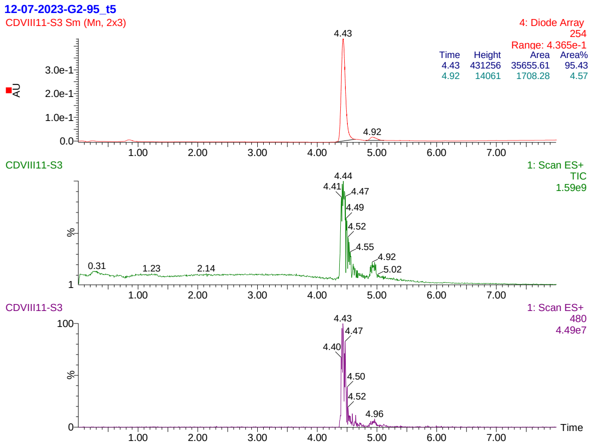

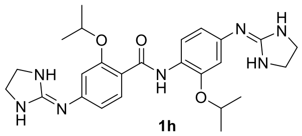

1h

Compound **1i**

^{1}H NMR (400 MHz, CD_3OD)

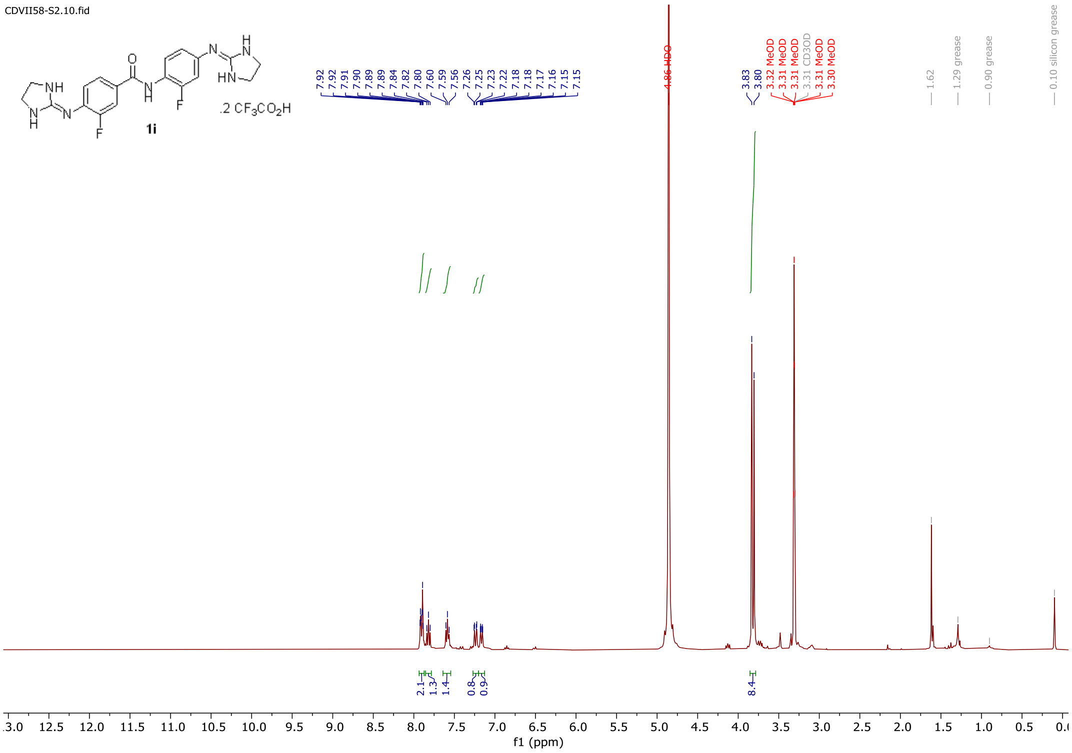

^{13}C NMR (101 MHz, CD_3OD)

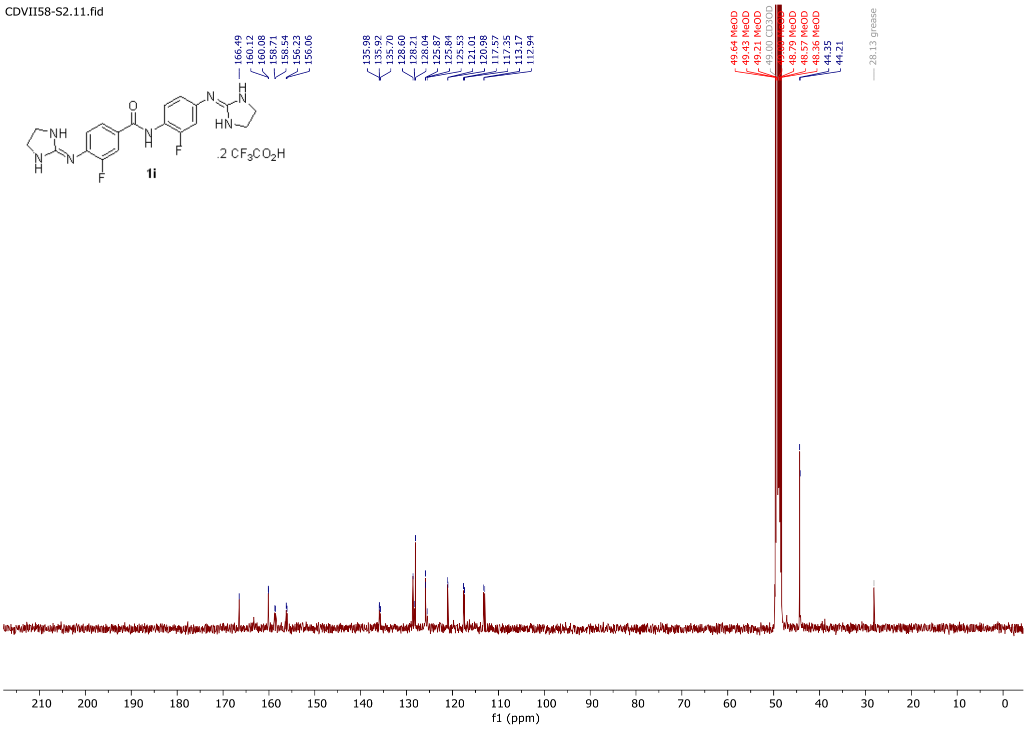

Compound **2a**

^{1}H NMR (500 MHz, DMSO-d_6)

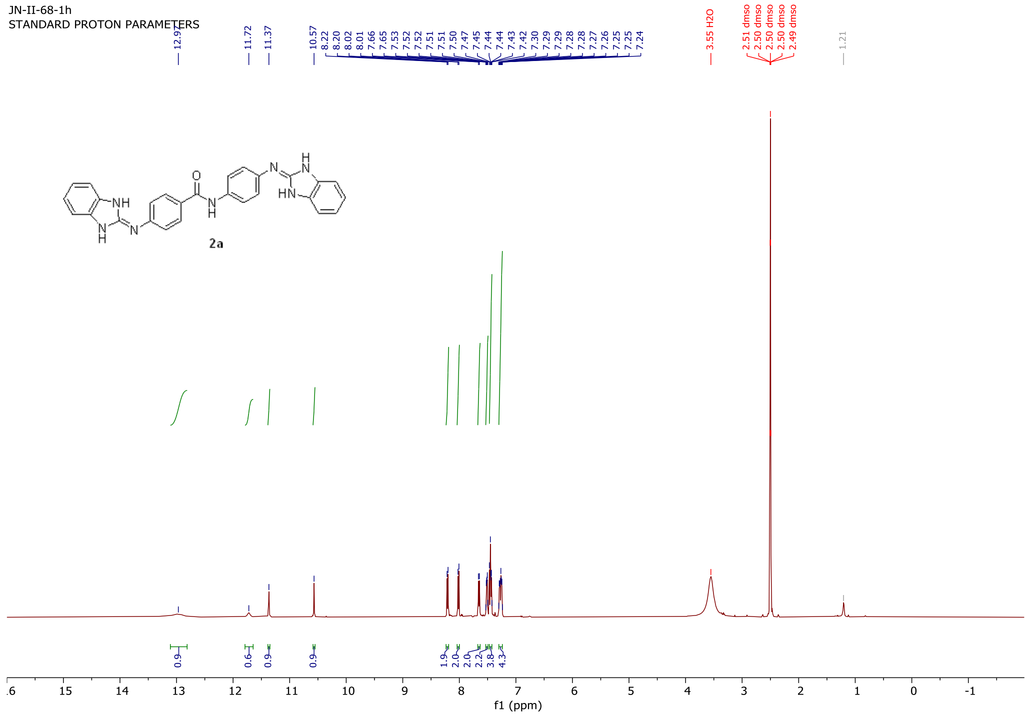

^{13}C NMR (126 MHz, DMSO-d_6)

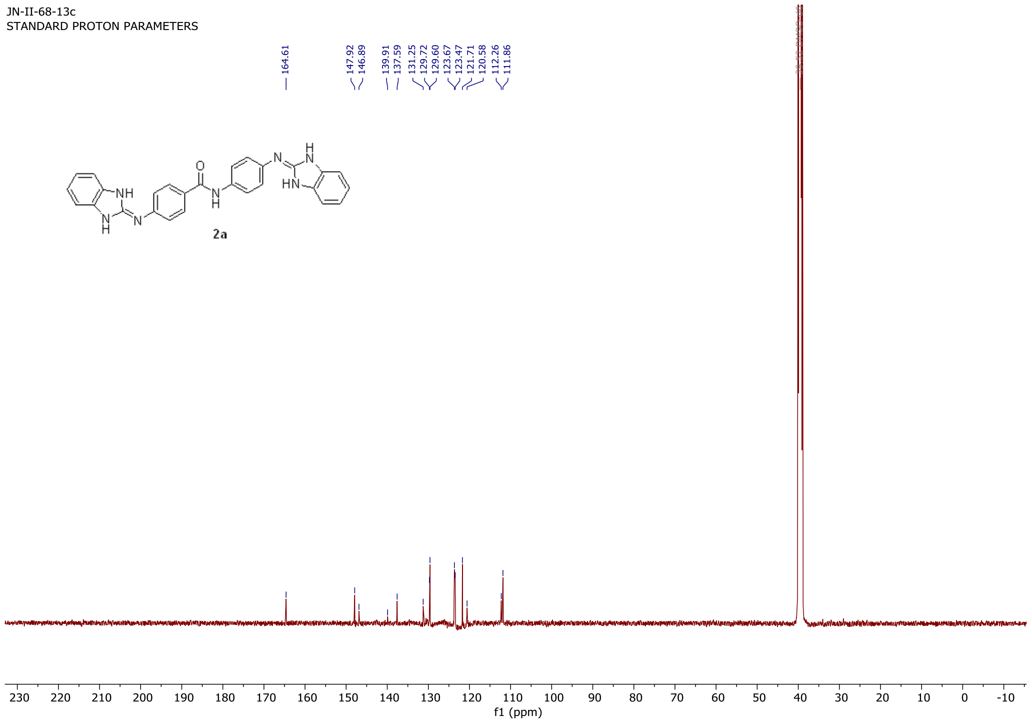

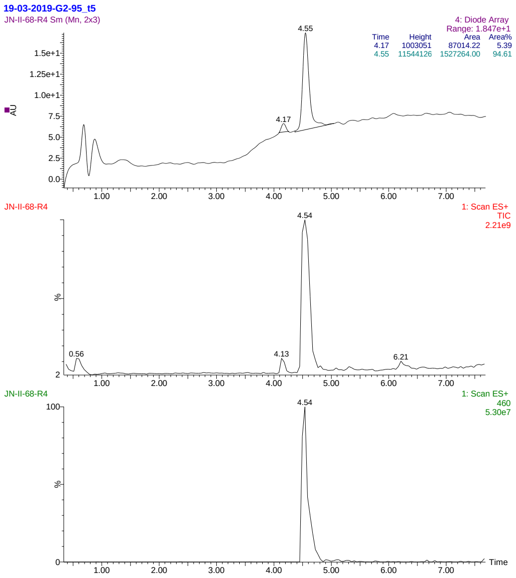

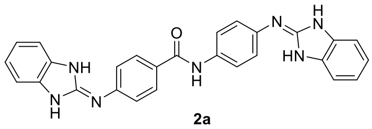

2a

Compound **2c**

^{1}H NMR (300 MHz, DMSO-d_6)

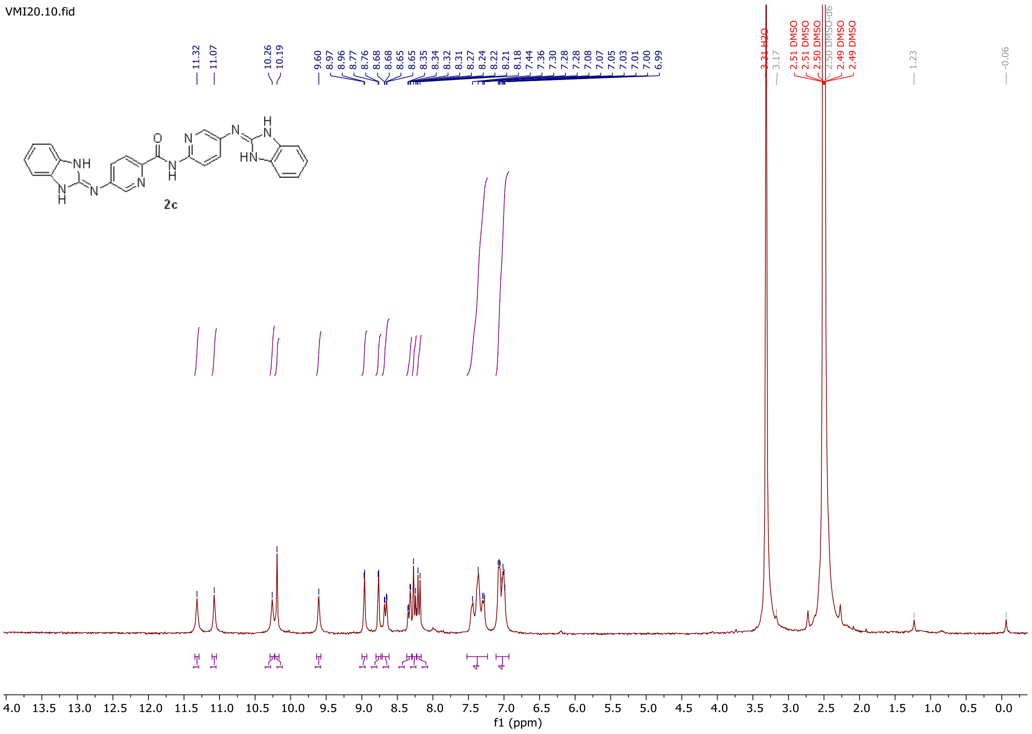

^{13}C NMR (75 MHz, DMSO-d_6)

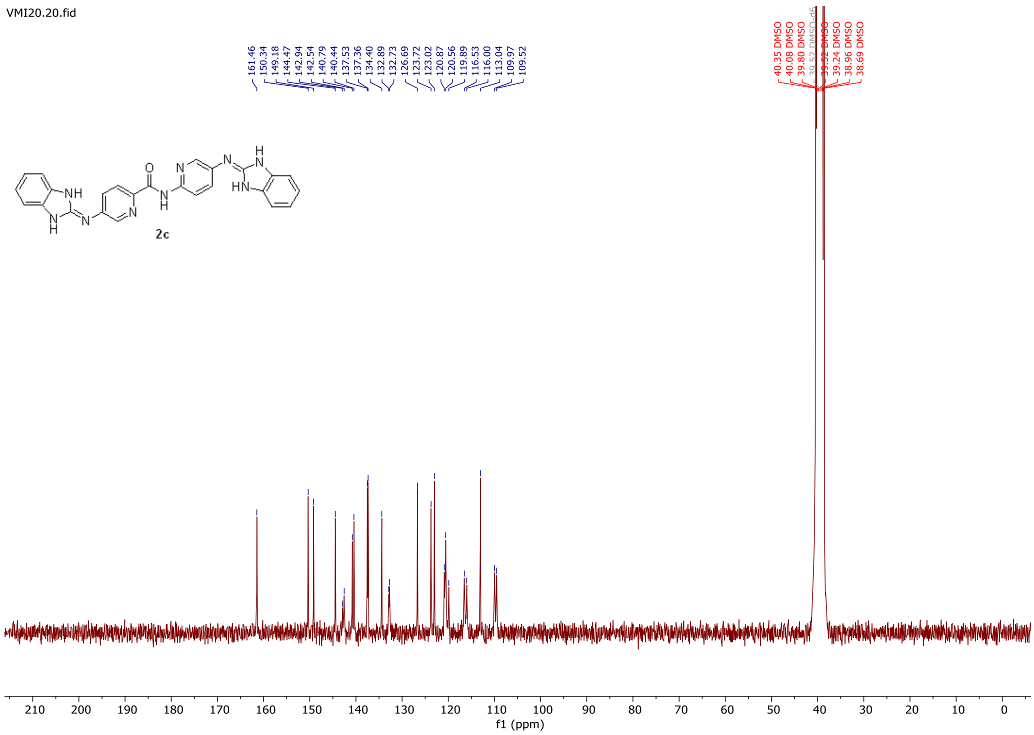

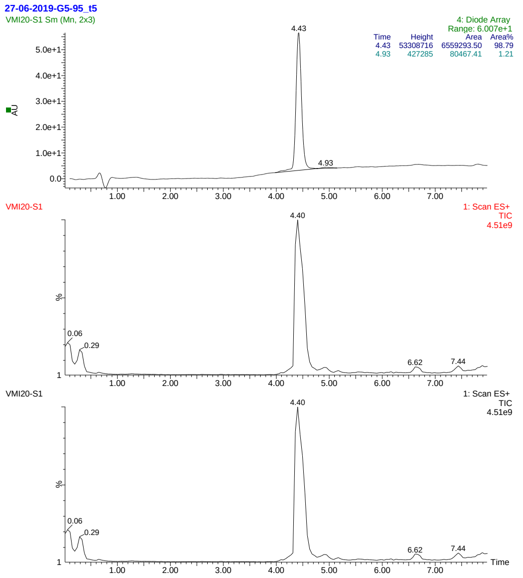

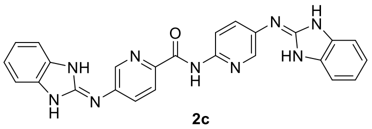

2c

Compound **2d**

^{1}H NMR (500 MHz, DMSO-d_6)

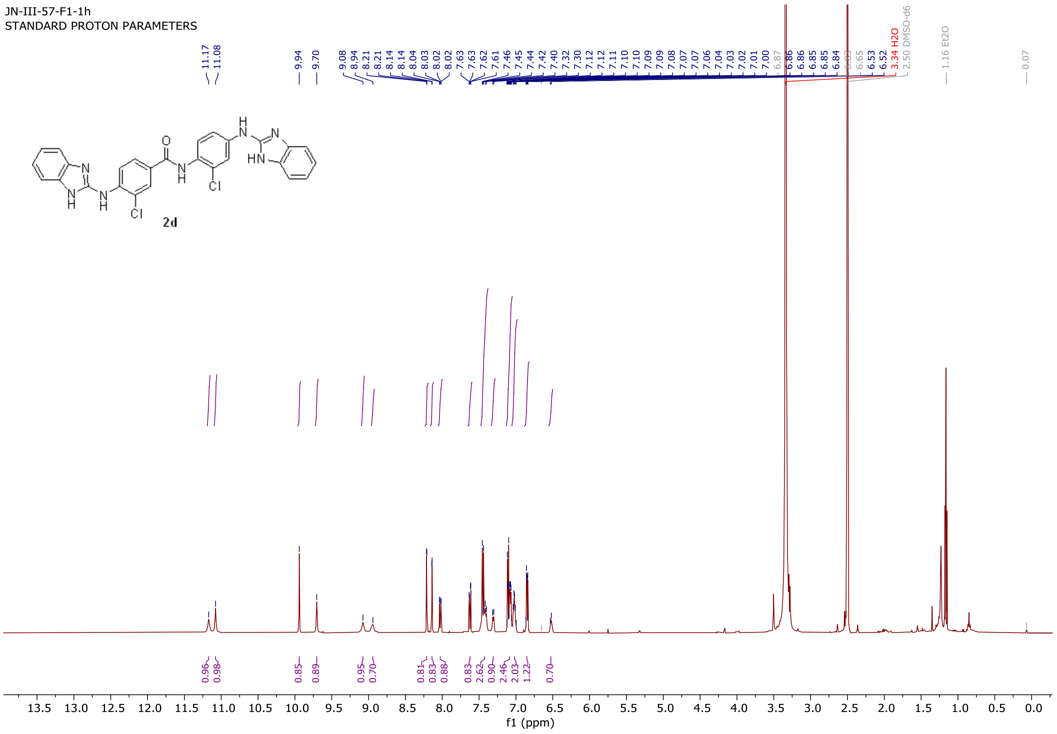

^{13}C NMR (126 MHz, DMSO-d_6)

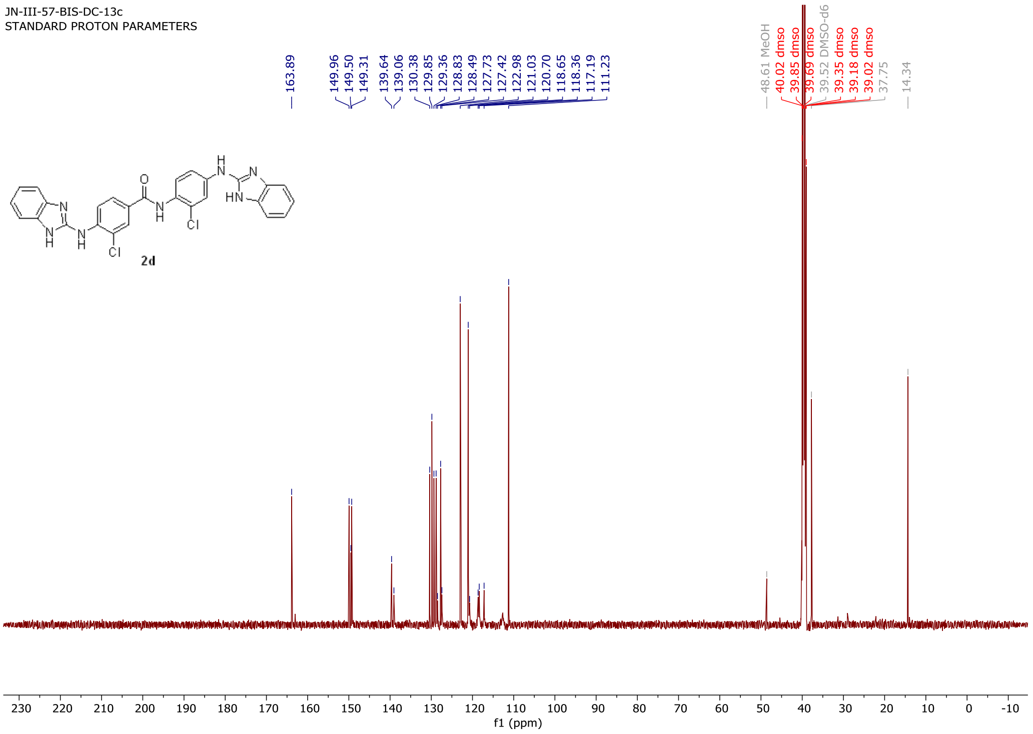

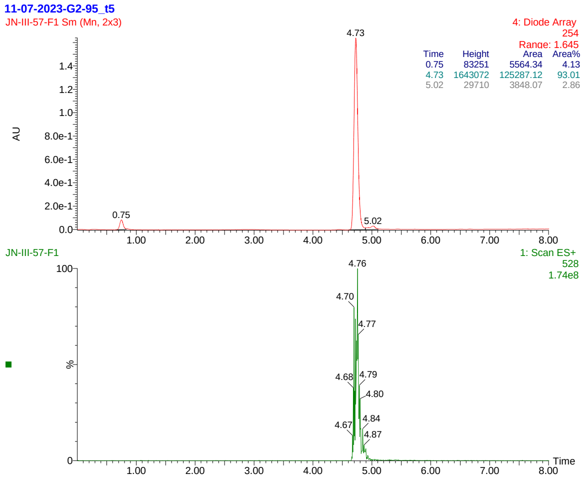

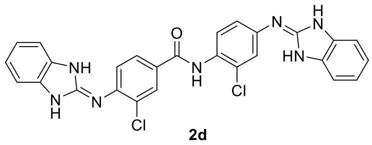

2d

Compound **2e**

^{1}H NMR (400 MHz, DMSO-d_6)

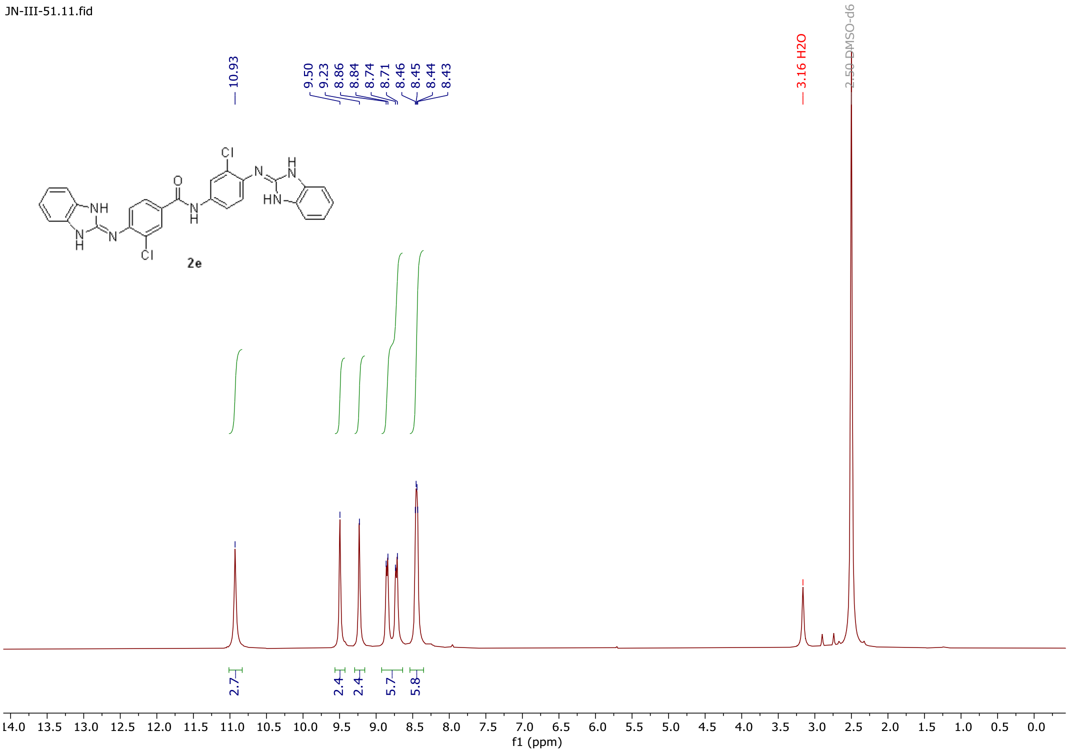

^{13}C NMR (100 MHz, DMSO-d_6)

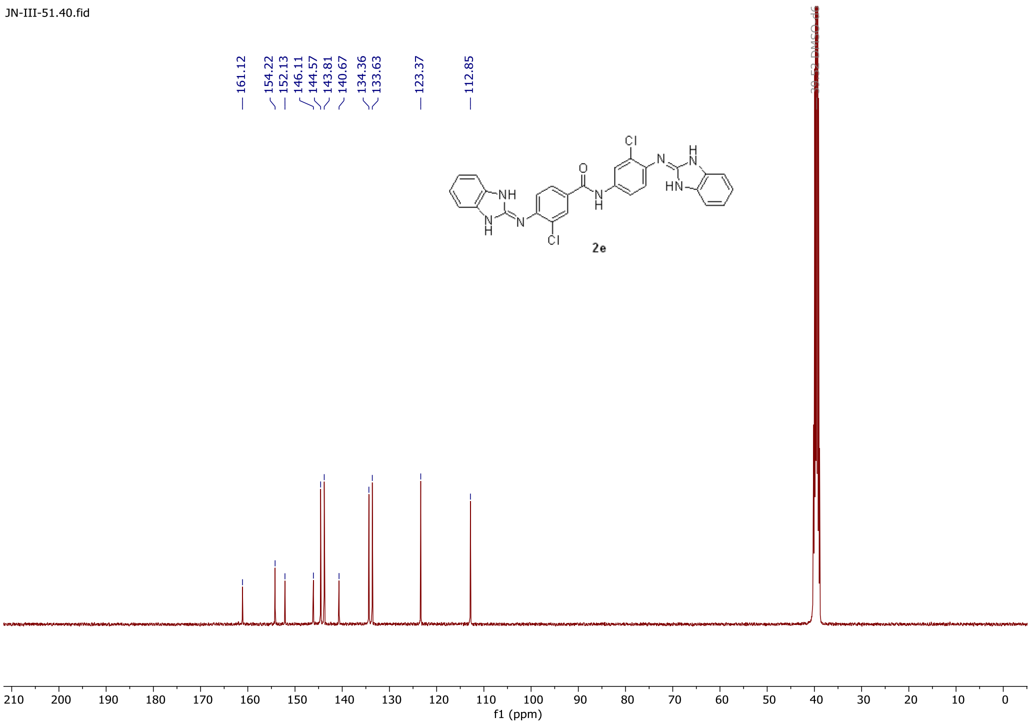

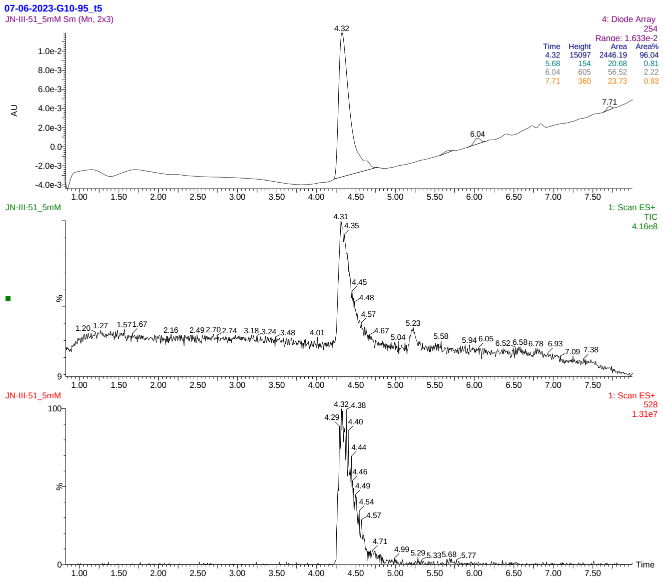

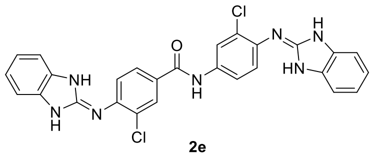

Compound **2f**

^{1}H NMR (400 MHz, DMSO-d_6)

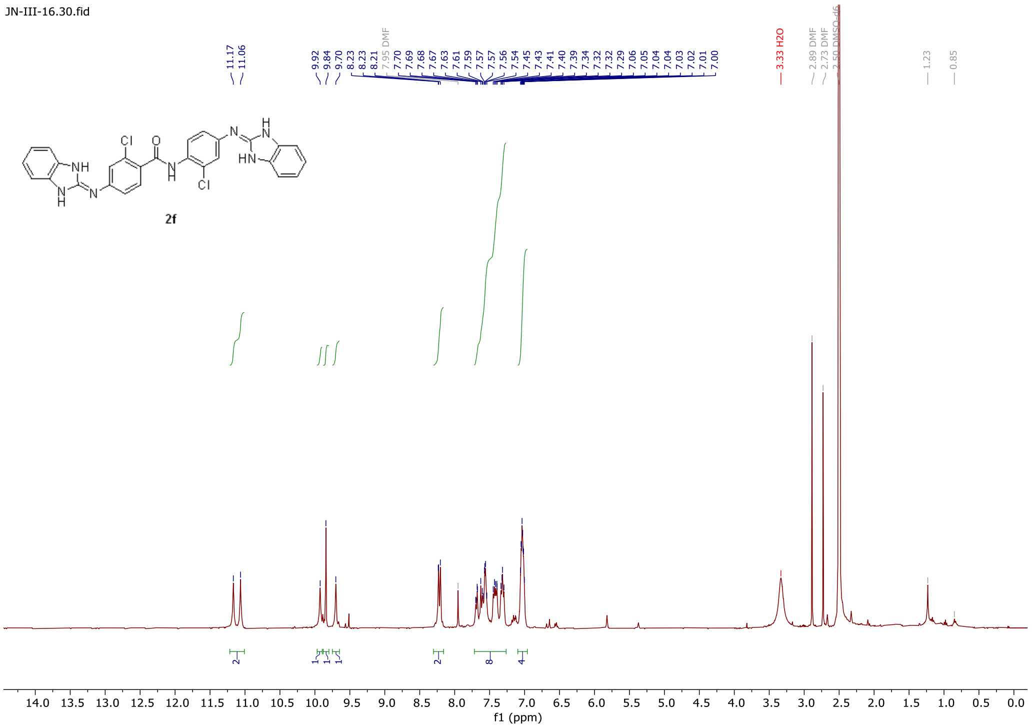

^{13}C NMR (100 MHz, DMSO-d_6)

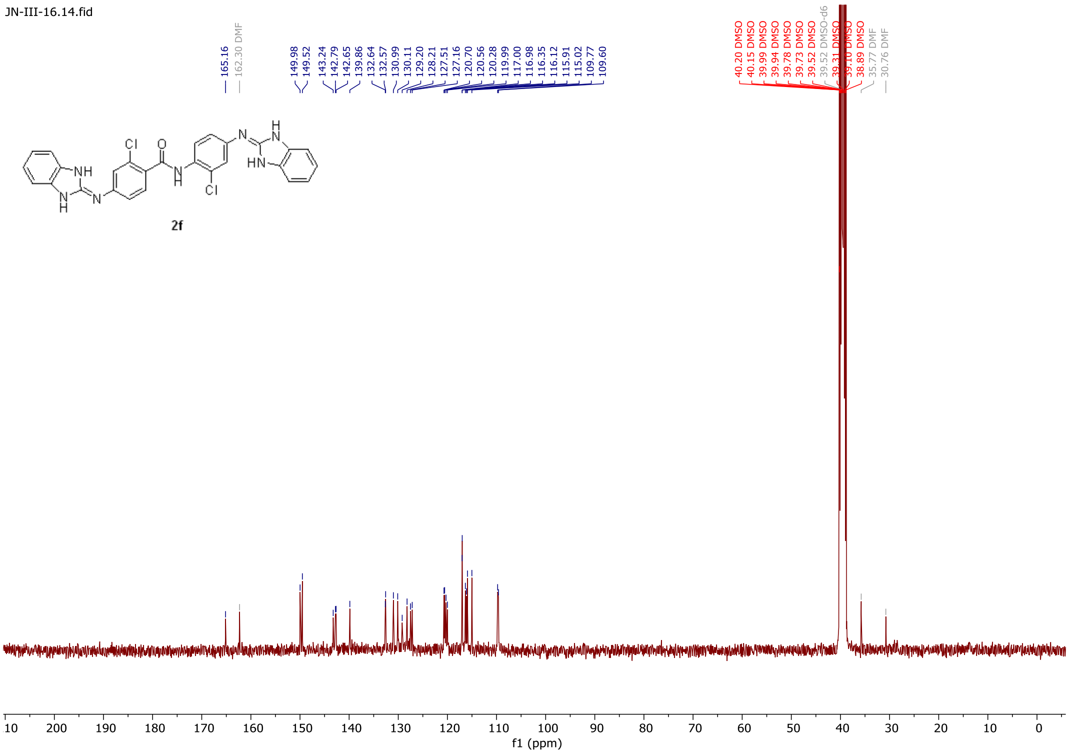

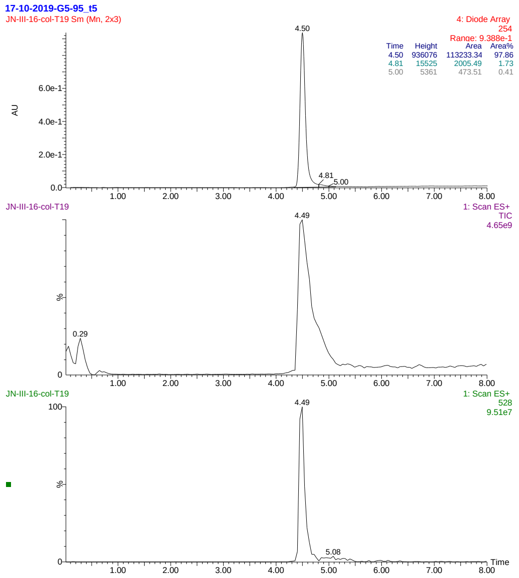

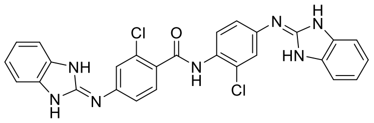

2f

Compound **2g**

^{1}H NMR (500 MHz, Methanol-d_4)

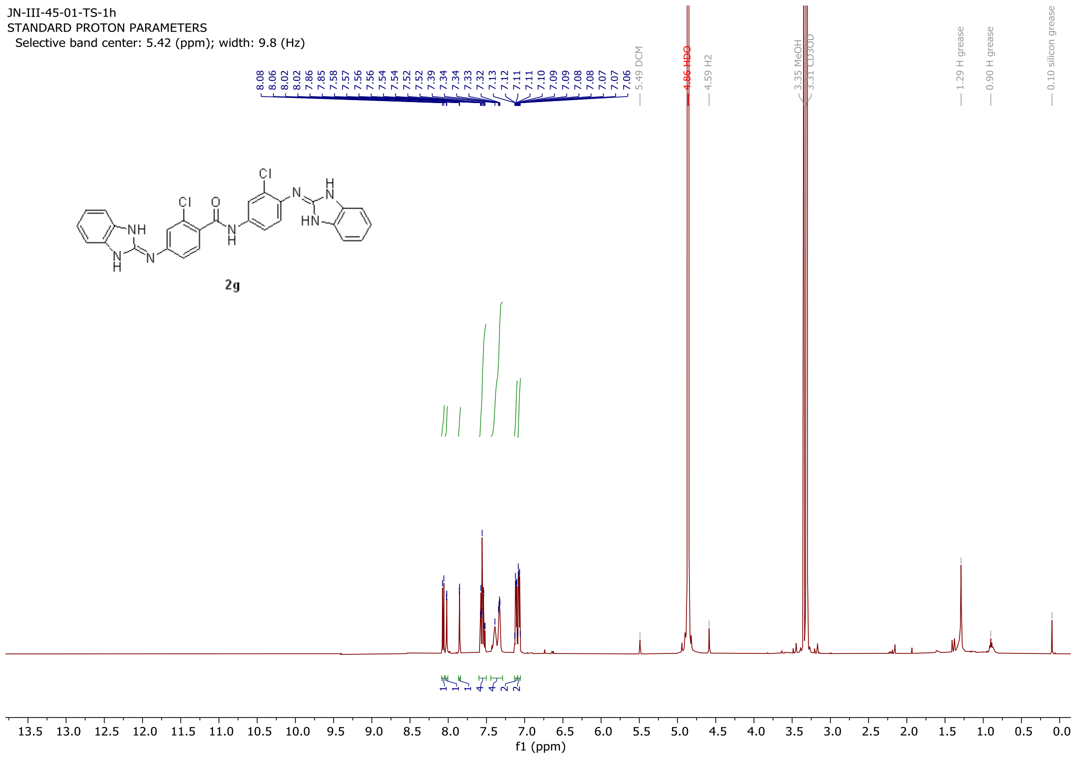

^{13}C NMR (126 MHz, Methanol-d_4)

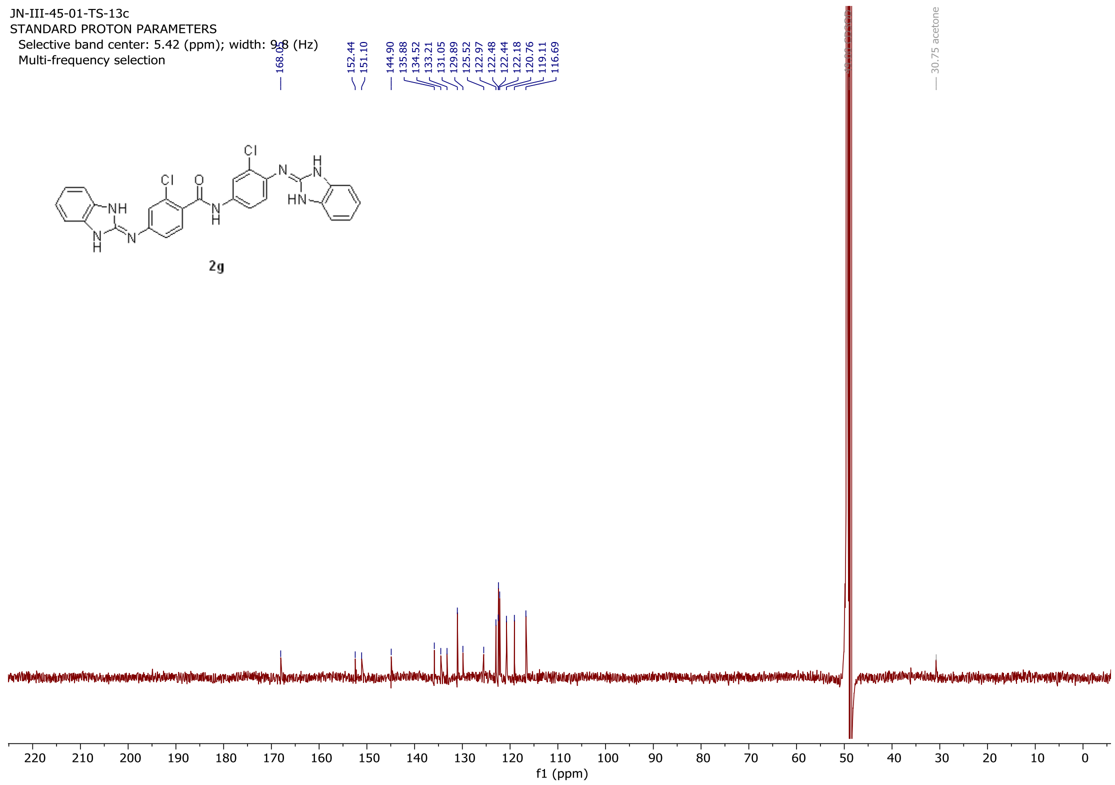

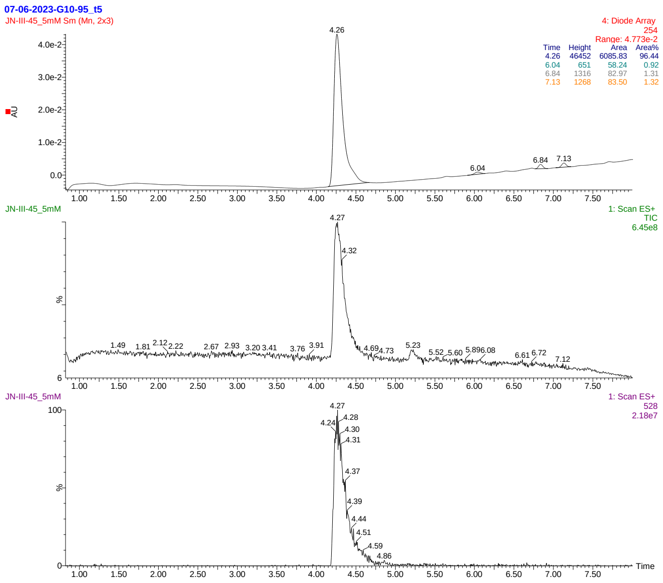

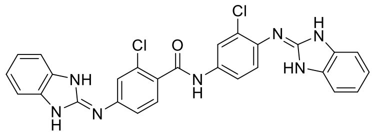

2g

Compound **12**

^{1}H NMR (400 MHz, DMSO-d_6)

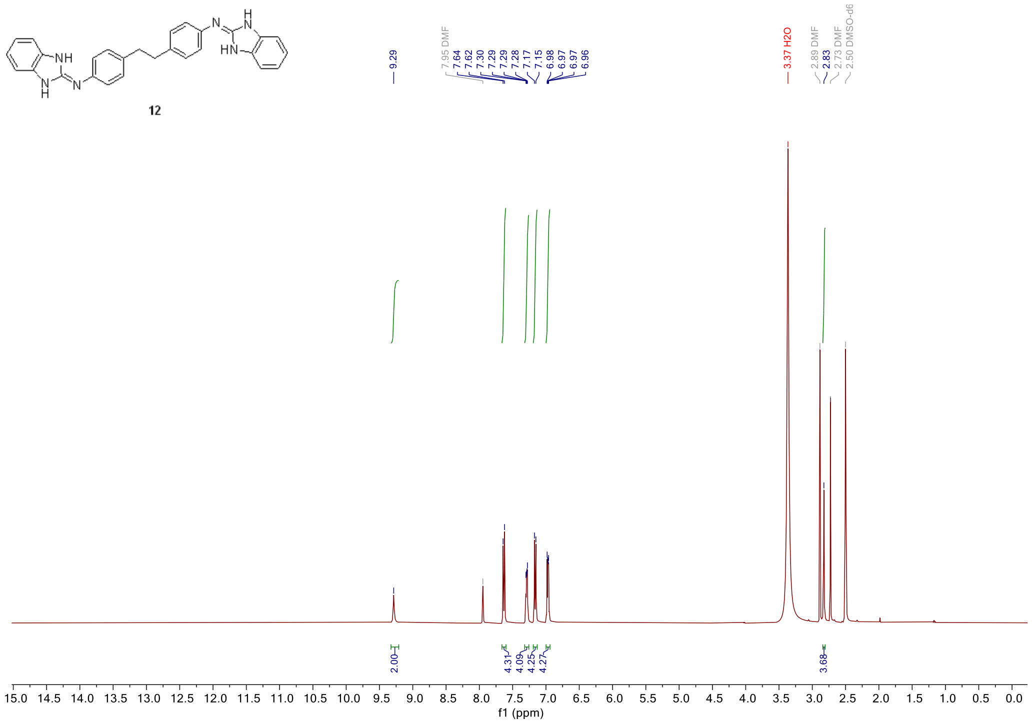

^{13}C NMR (101 MHz, DMSO-d_6)

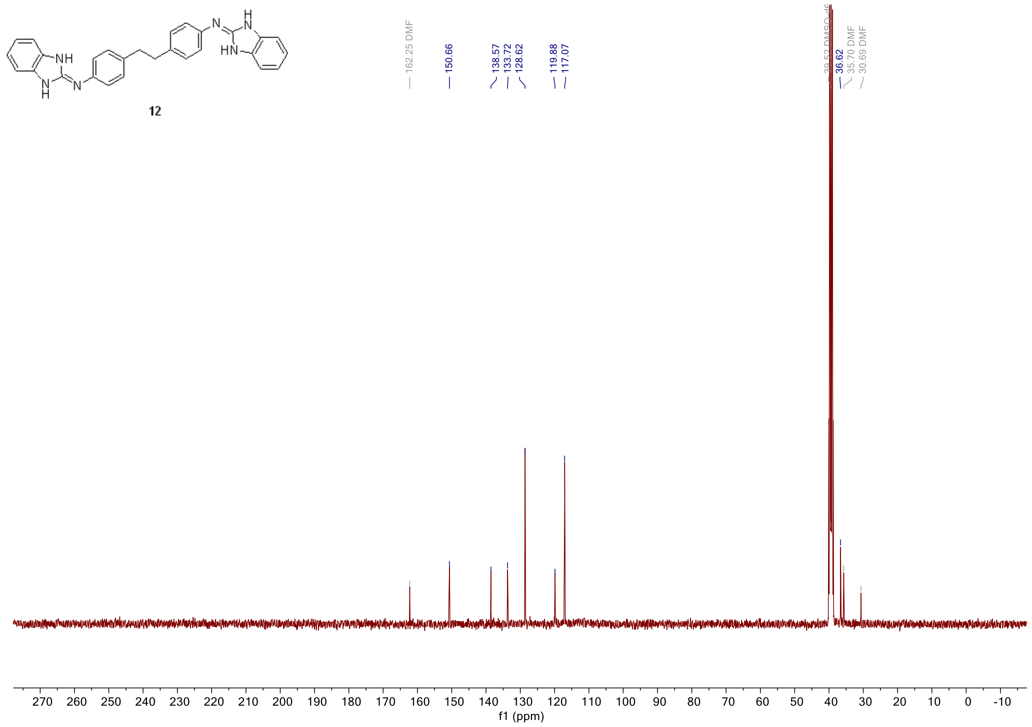

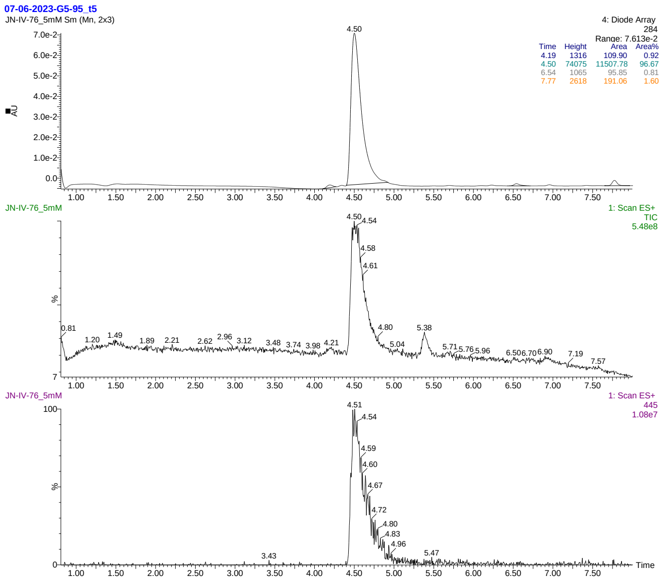

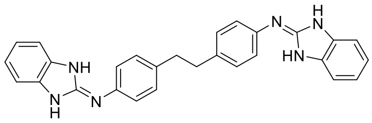

12

Compound **13**

^{1}H NMR (400 MHz, DMSO-d_6)

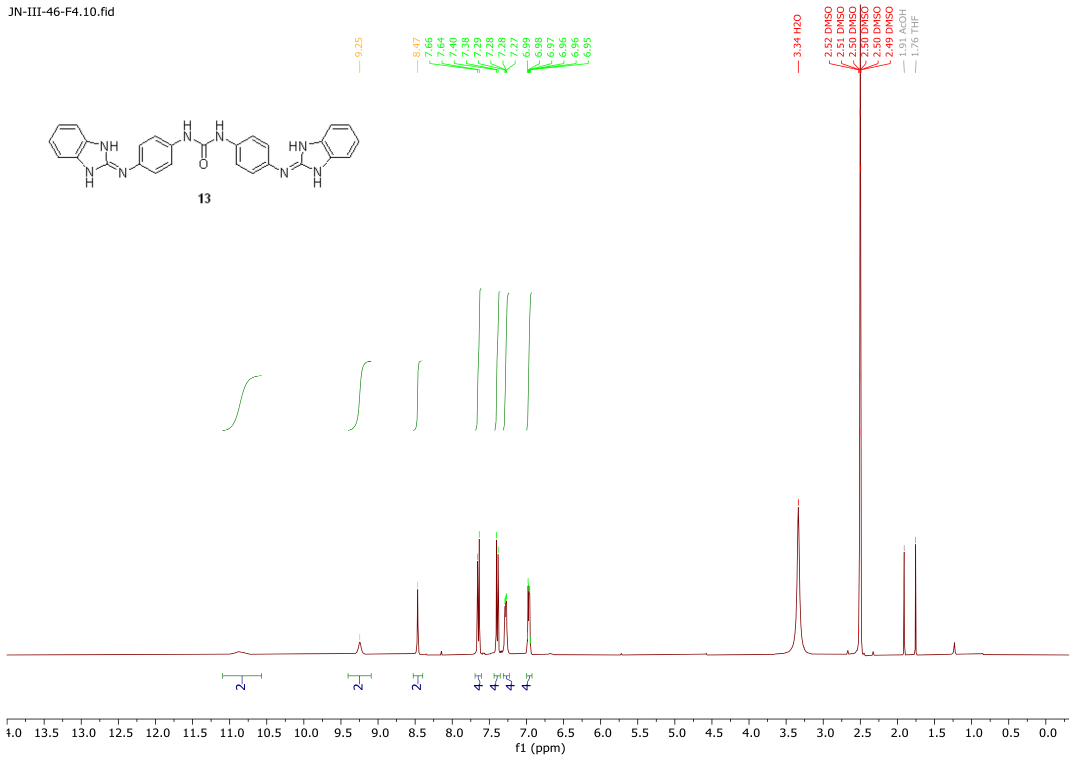

^{13}C NMR (100 MHz, DMSO-d_6)

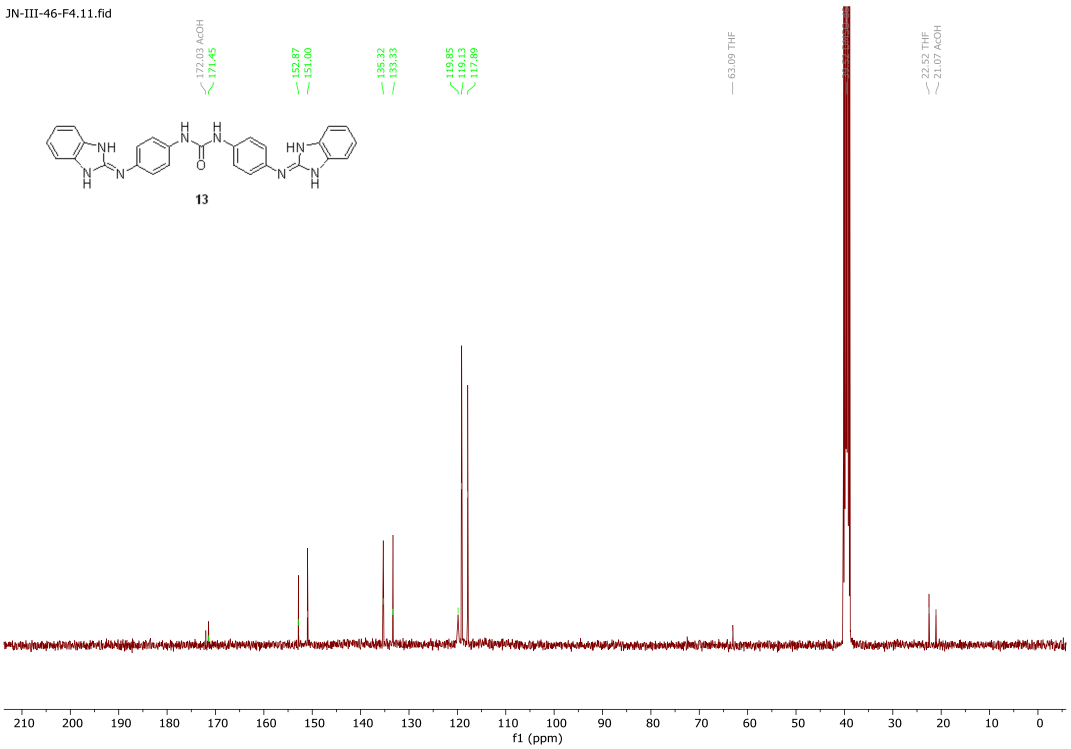

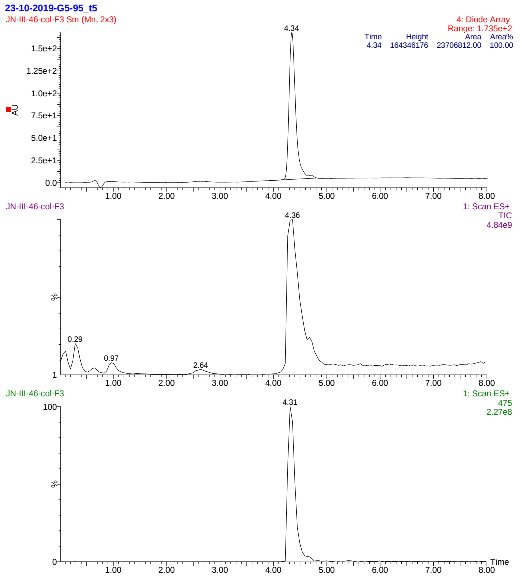

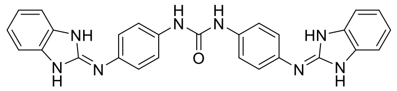

13

Compound **16**

^{1}H NMR (400 MHz, DMSO-d_6)

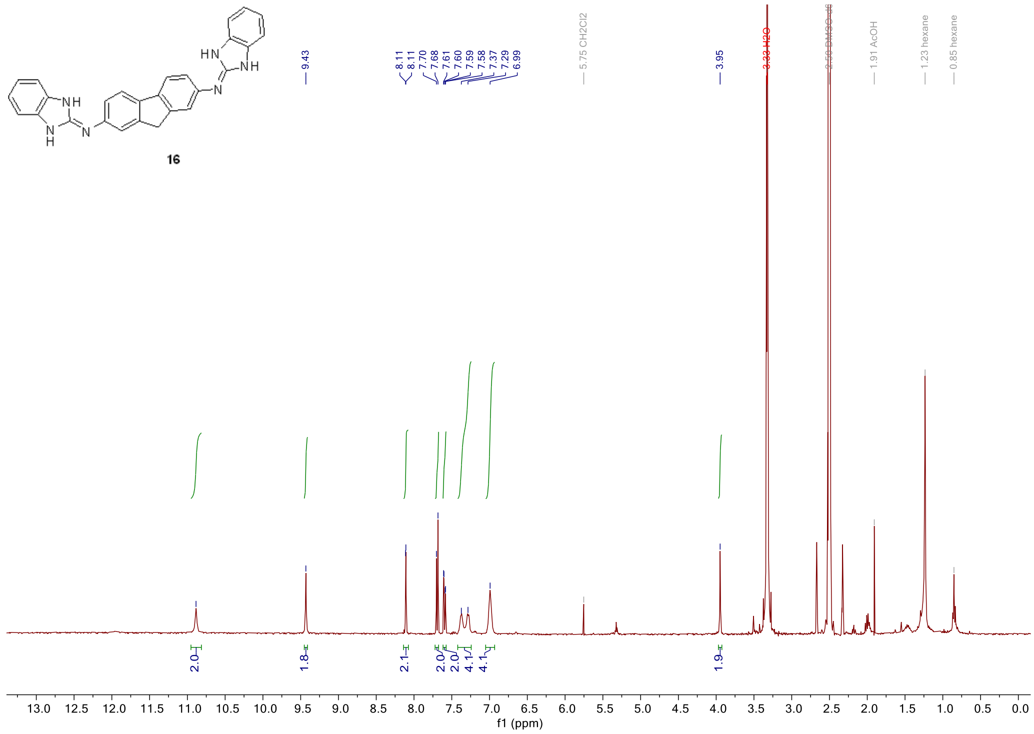

^{13}C NMR (126 MHz, DMSO-d_6)

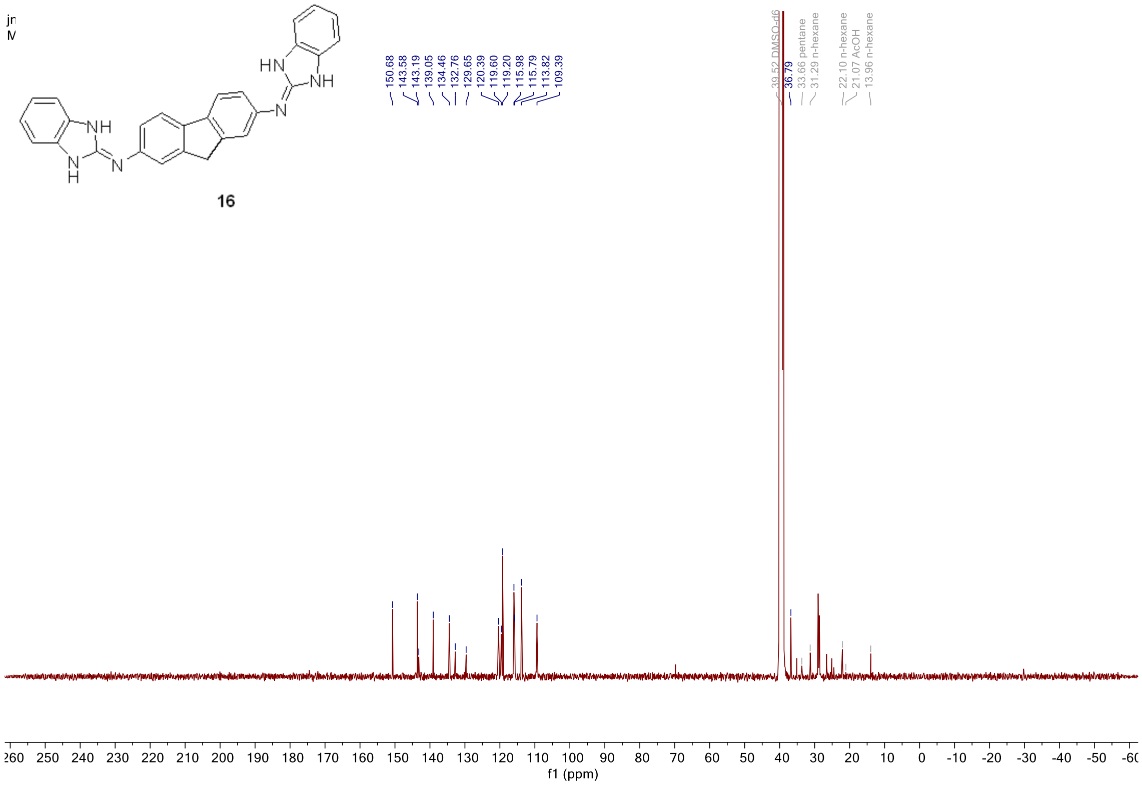

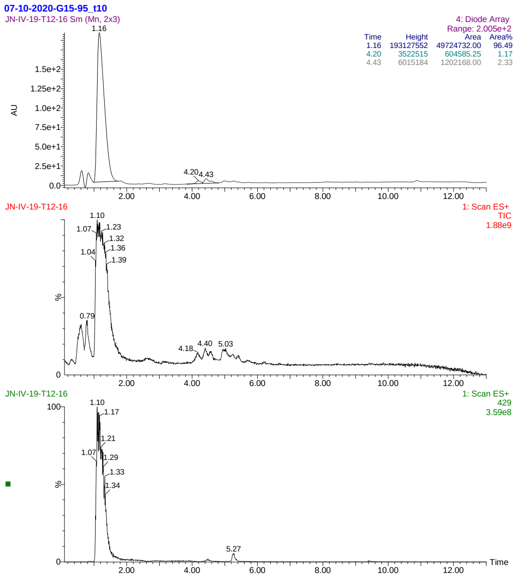
07-10-2020-G15-95_t10
JN-IV-19-T12-16 Sm (Mn, 2x3)
4: Diode Array
Range: 2.005e+2
Time Height Area Area%
1.16 193127552 49724732.00 96.49
4.20 3522515 604585.25 1.17
4.43 6015184 1202168.00 2.33
JN-IV-19-T12-16
1: Scan ES+
TIC
1.88e9
JN-IV-19-T12-16
1: Scan ES+
429
3.59e8
Time

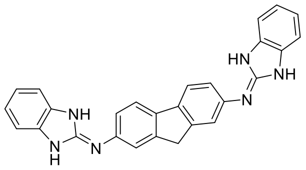

16

Compound **3a**

^{1}H NMR (400 MHz, DMSO-d_6)

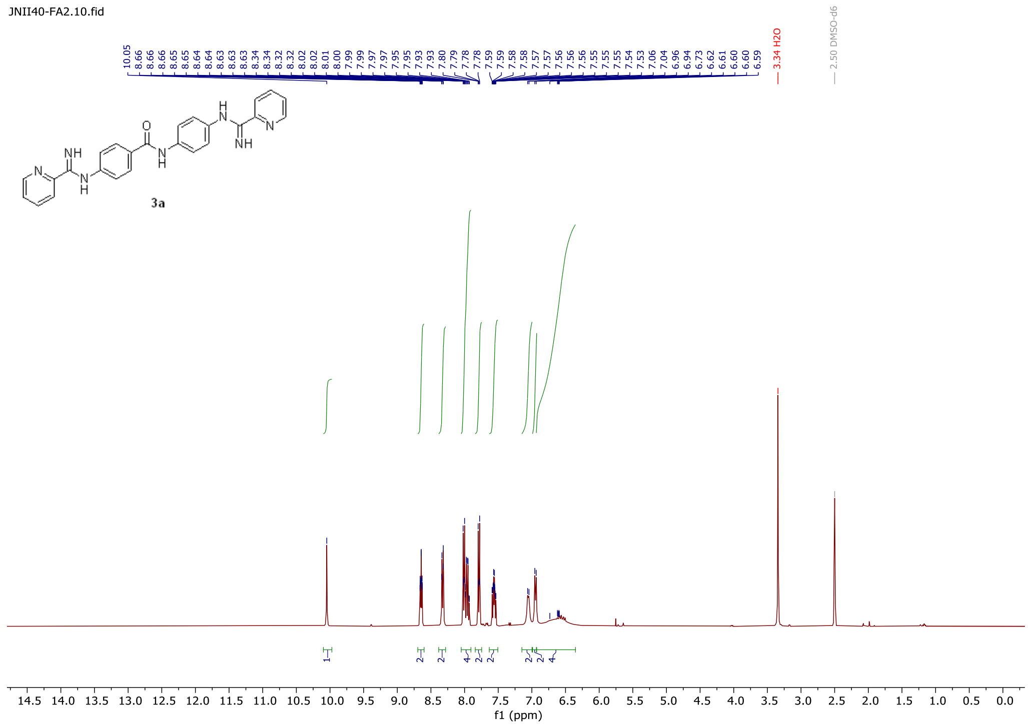

^{13}C NMR (101 MHz, DMSO-d_6)

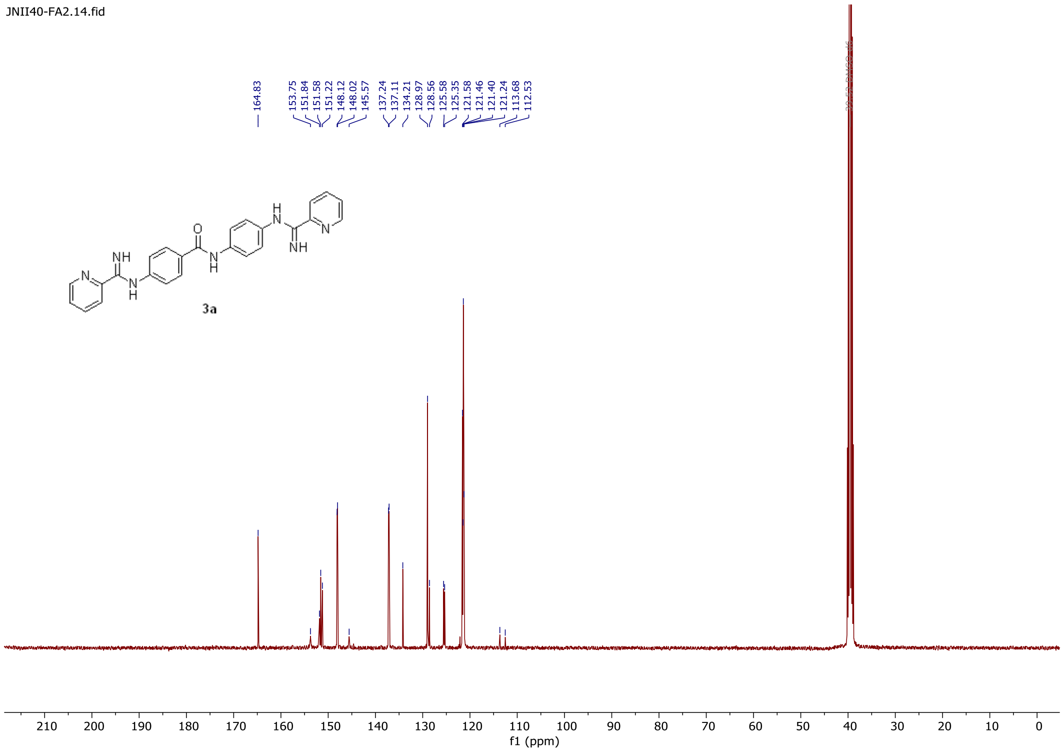

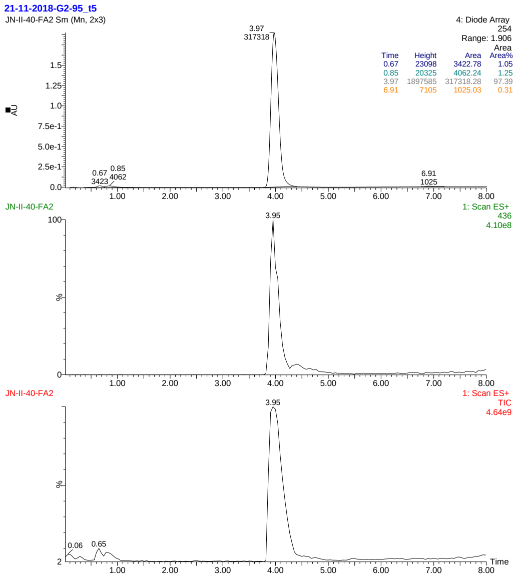

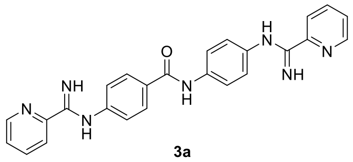

3a

Compound **3b**

^{1}H NMR (400 MHz, DMSO-d_6)

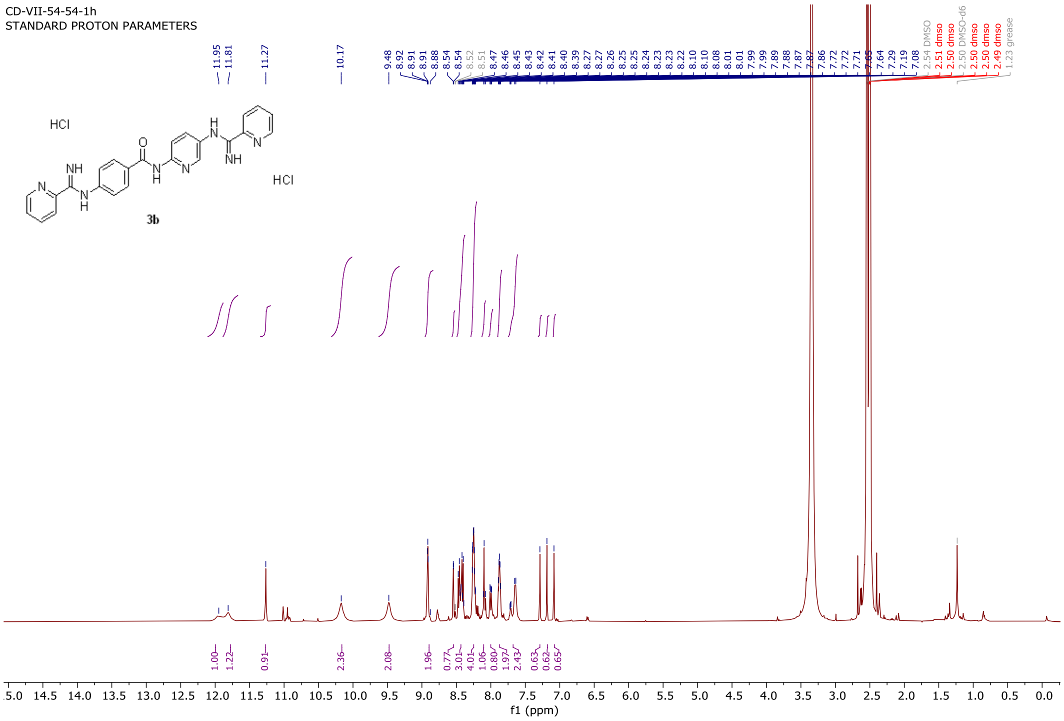

^{13}C NMR (126 MHz, DMSO-d_6)

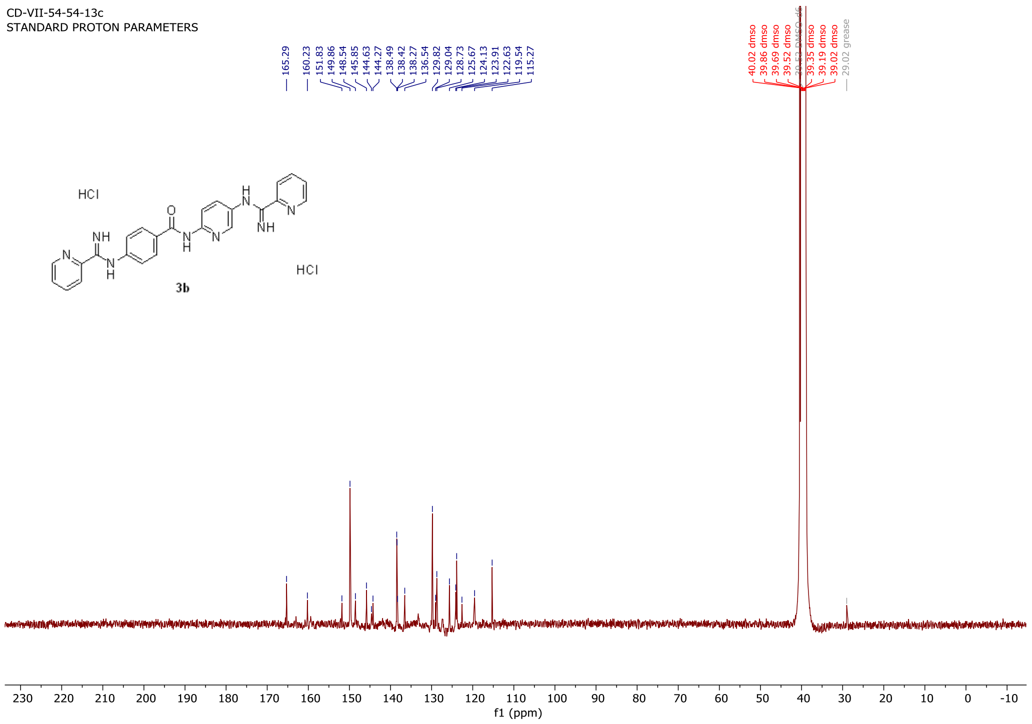

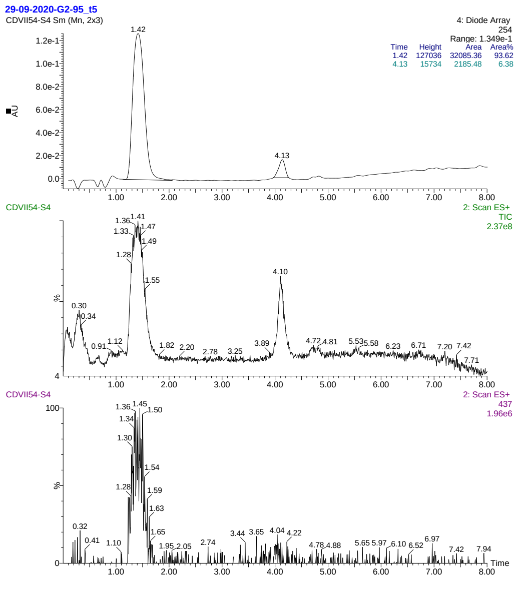

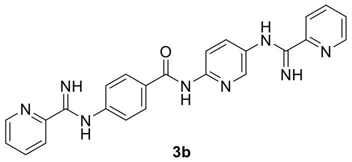

3b

Compound **3c** (free base and TFA salt)

^{1}H NMR (500 MHz, DMSO-d_6)

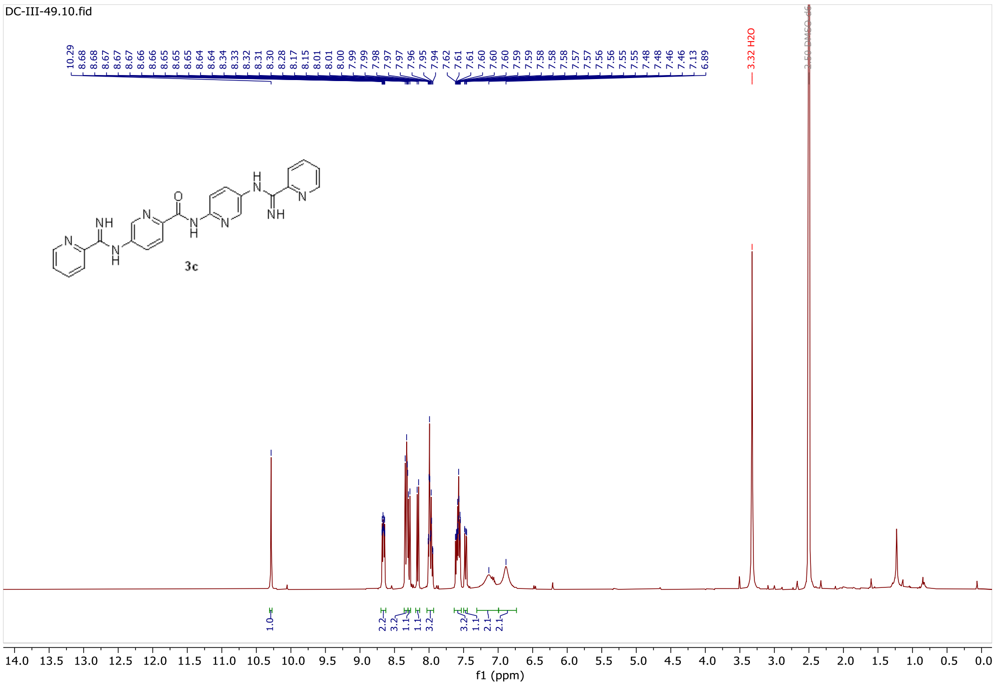

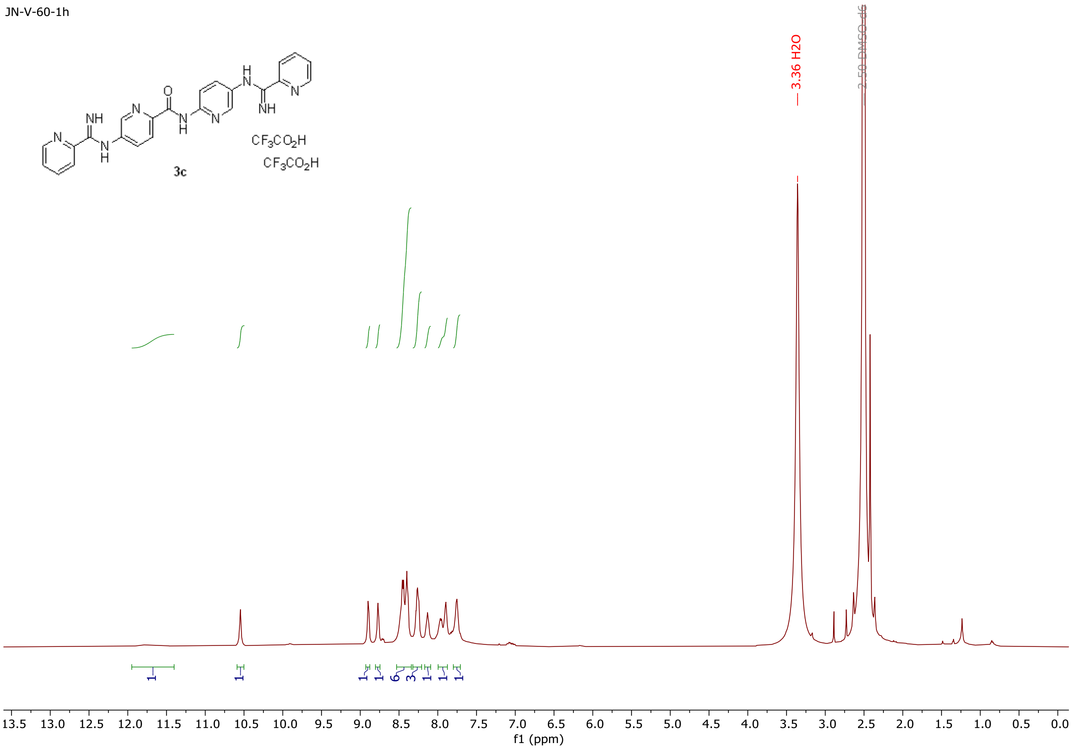

^{13}C NMR (126 MHz, DMSO-d_6)

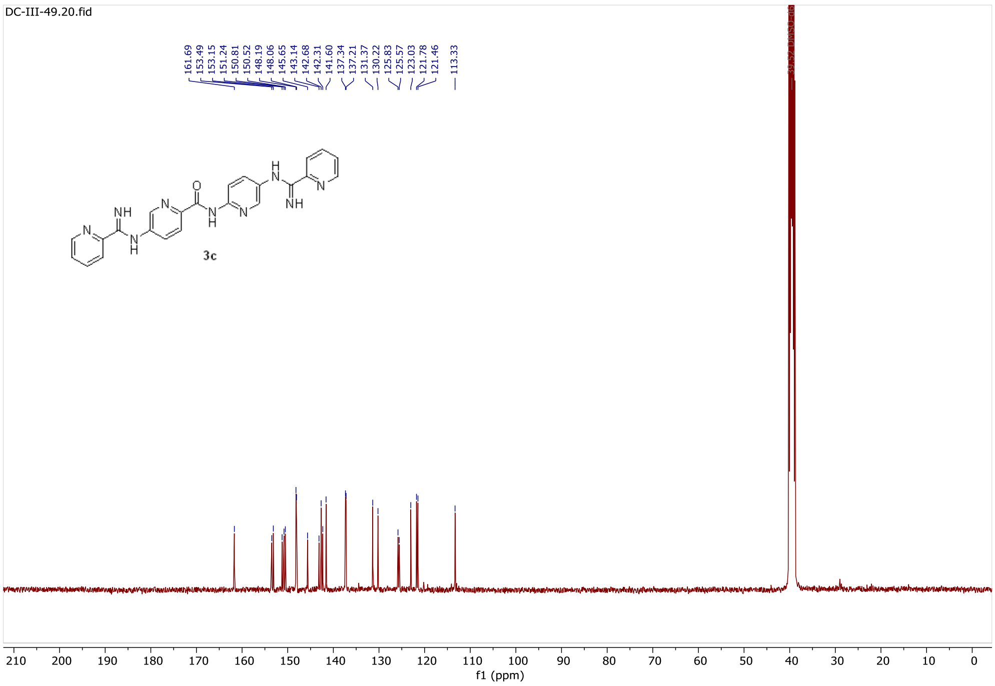

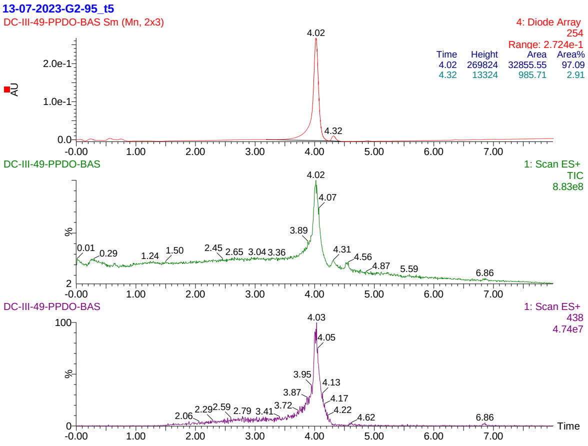

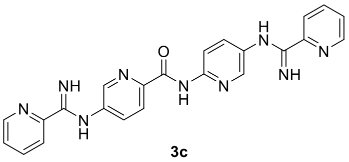

3c

Compound **3d**

^{1}H NMR (500 MHz, DMSO-d_6)

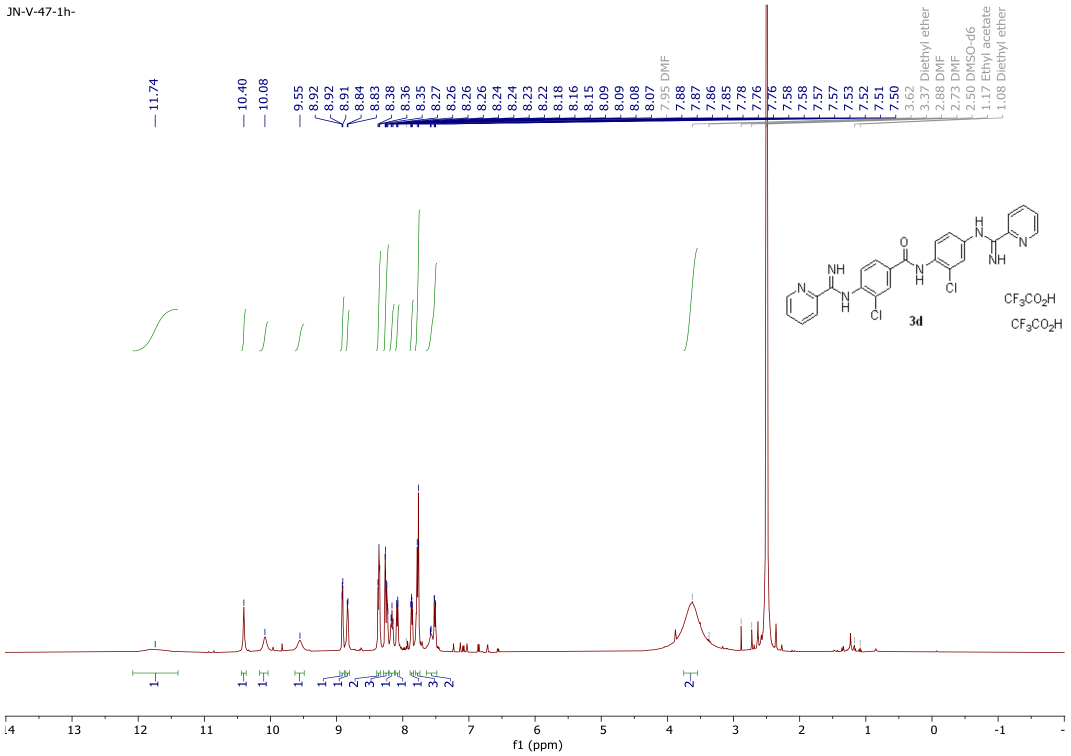

^{13}C NMR (126 MHz, DMSO-d_6)

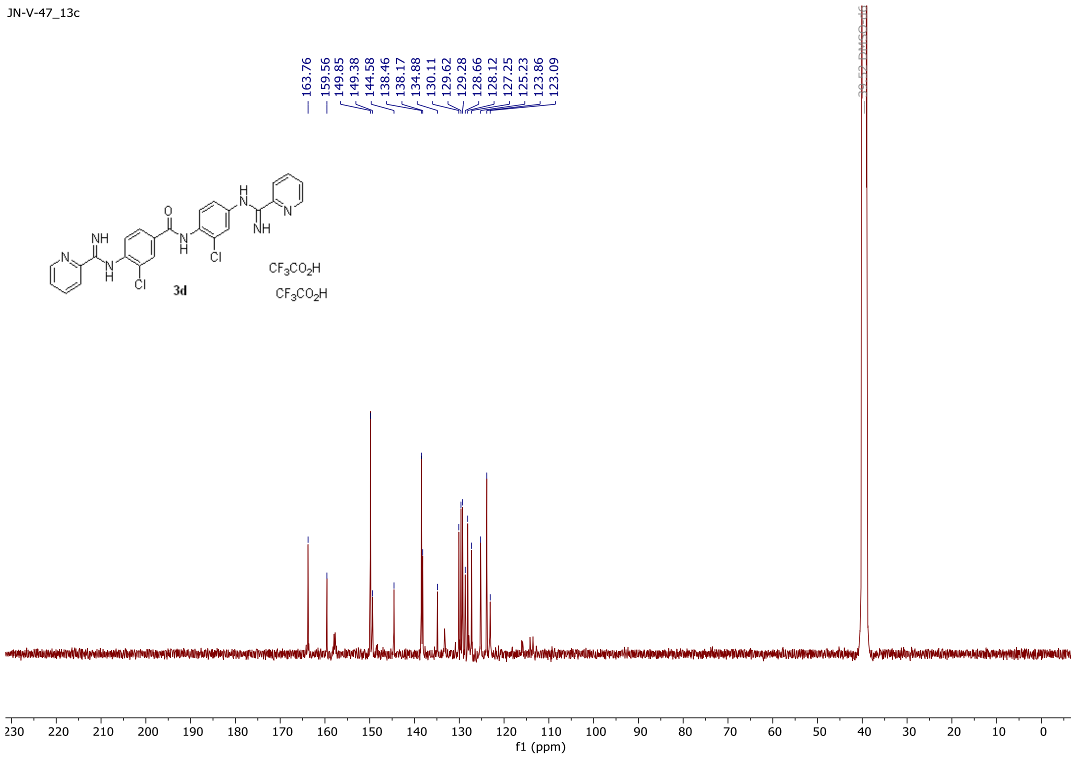

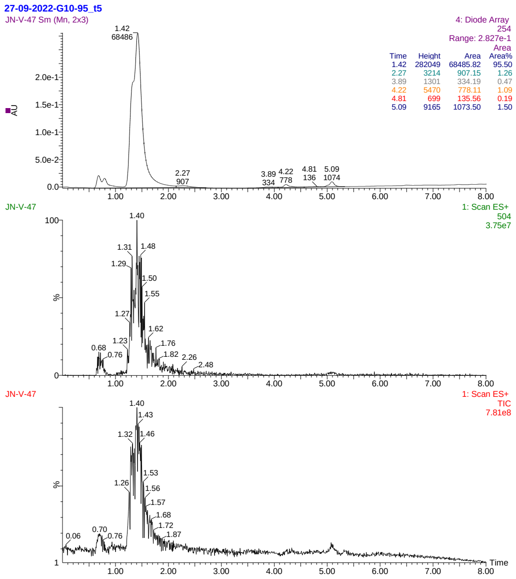

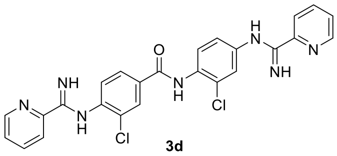

3d

Compound **3e**

^{1}H NMR (400 MHz, DMSO-d_6)

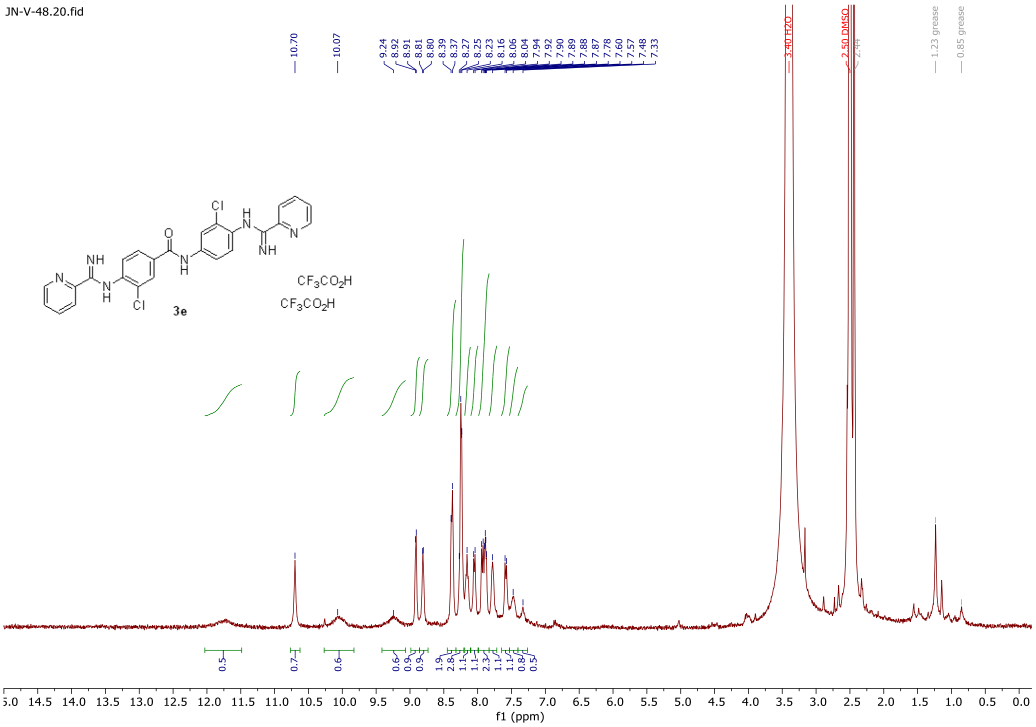

^{13}C NMR (101 MHz, DMSO-d_6)

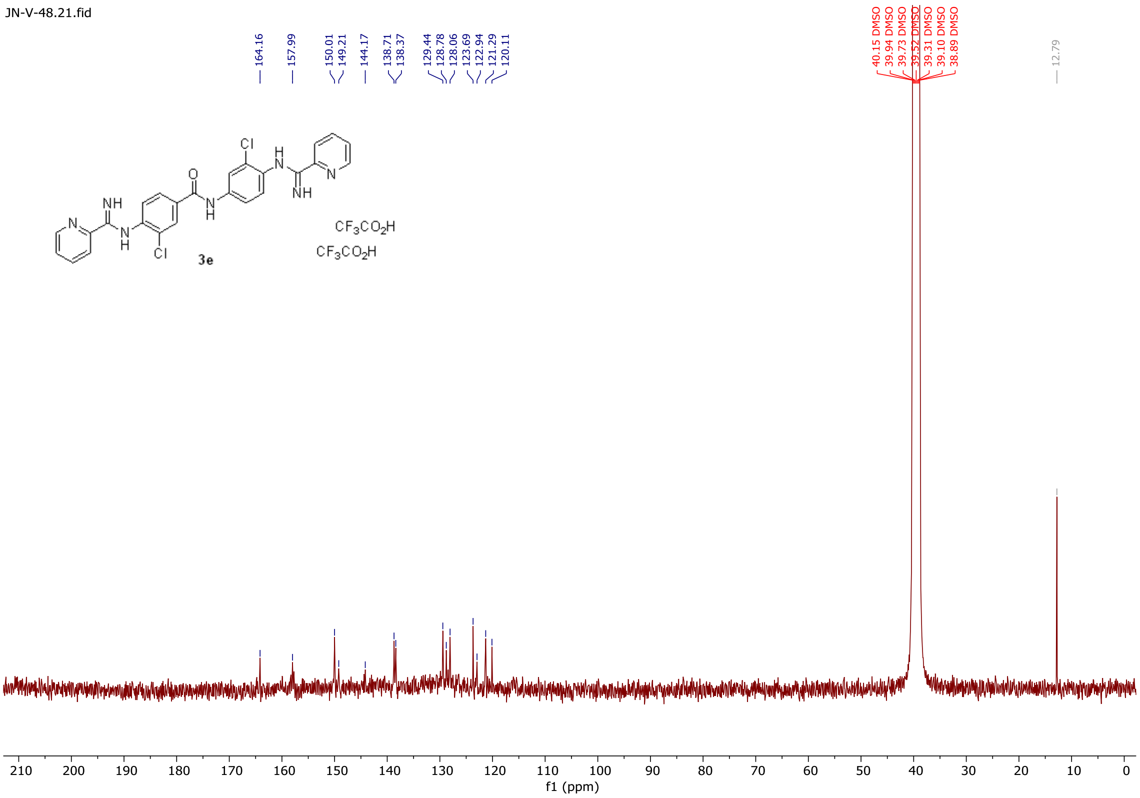

Compound **3f**

^{1}H NMR (500 MHz, DMSO-d_6)

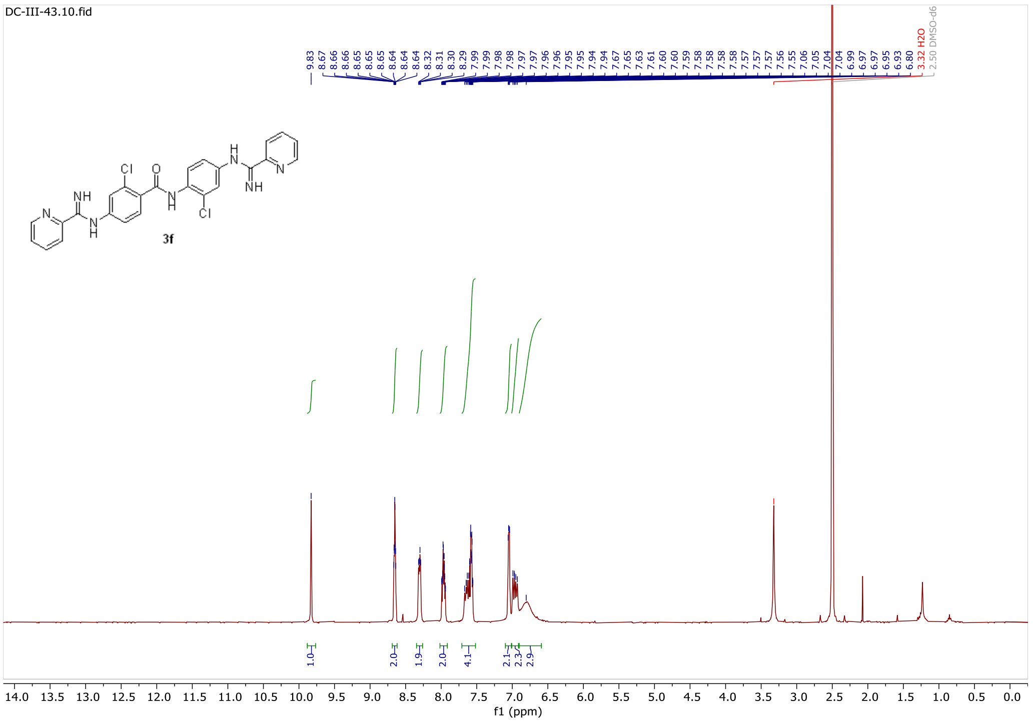

^{13}C NMR (101 MHz, DMSO-d_6)

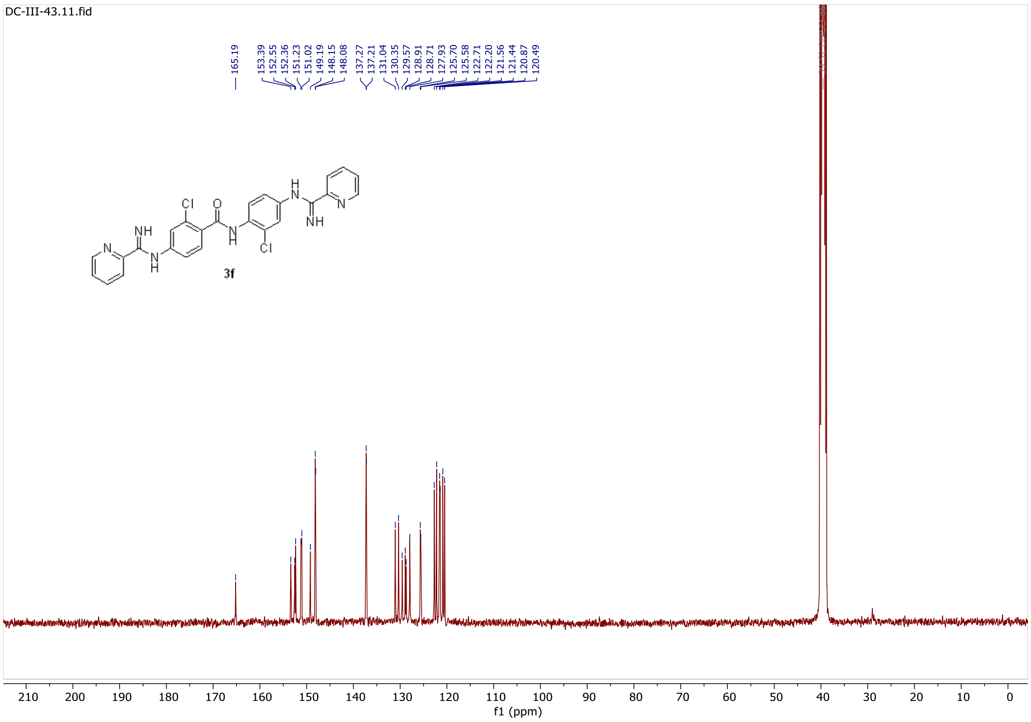

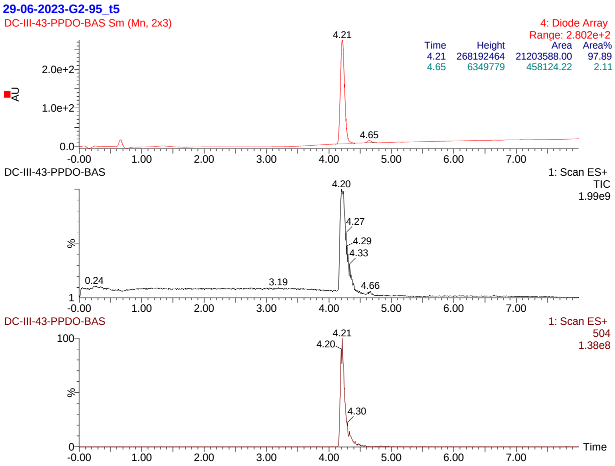

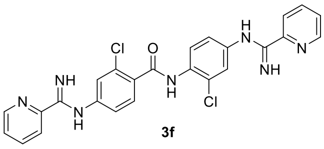

Compound **3g**

^{1}H NMR (400 MHz, DMSO-d_6)

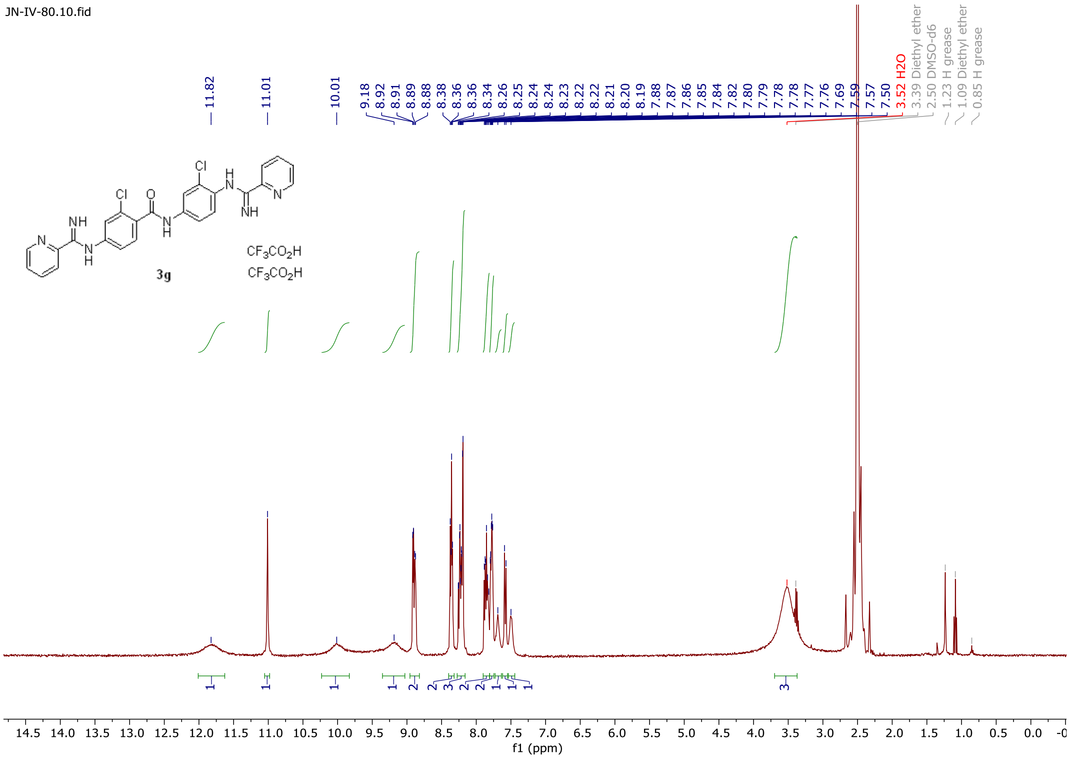

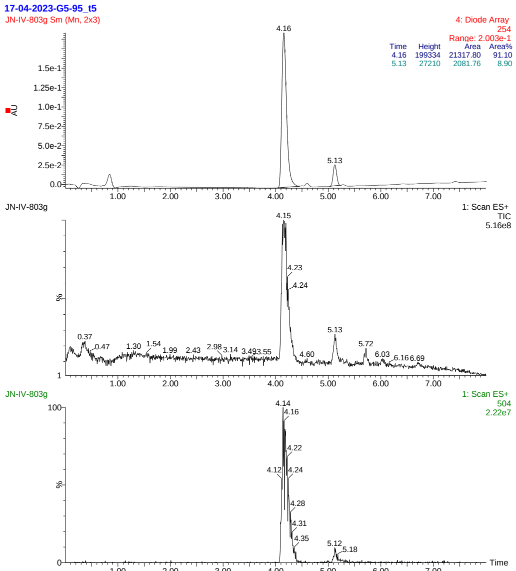

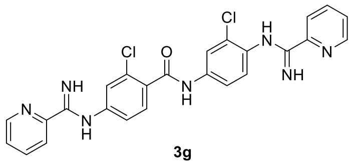

3g

Compound **3h**

^{1}H NMR (400 MHz, DMSO-d_6)

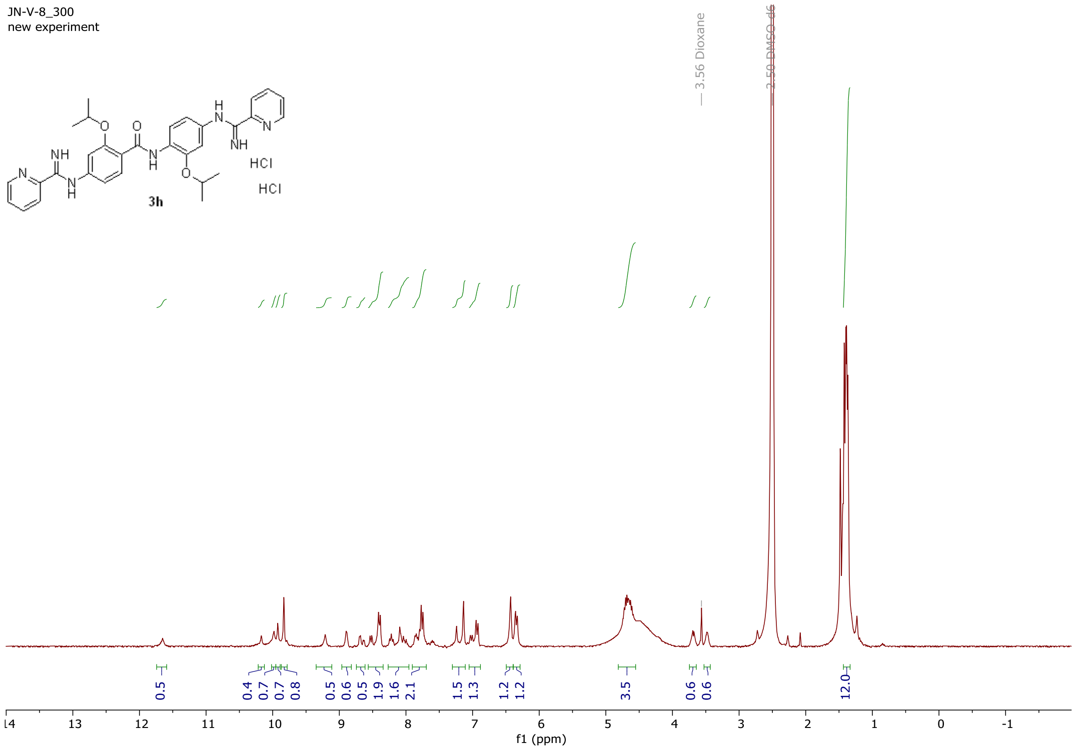

^{13}C NMR (101 MHz, DMSO-d_6)

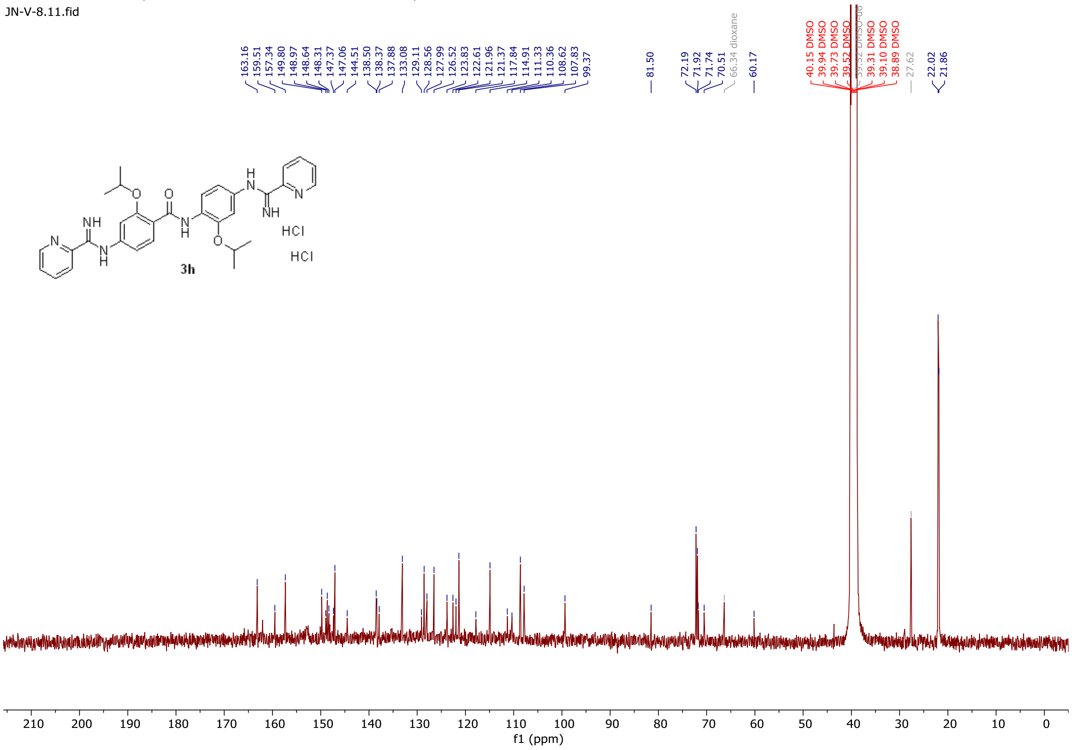

Compound **3i**

^{1}H NMR (300 MHz, DMSO-d_6)

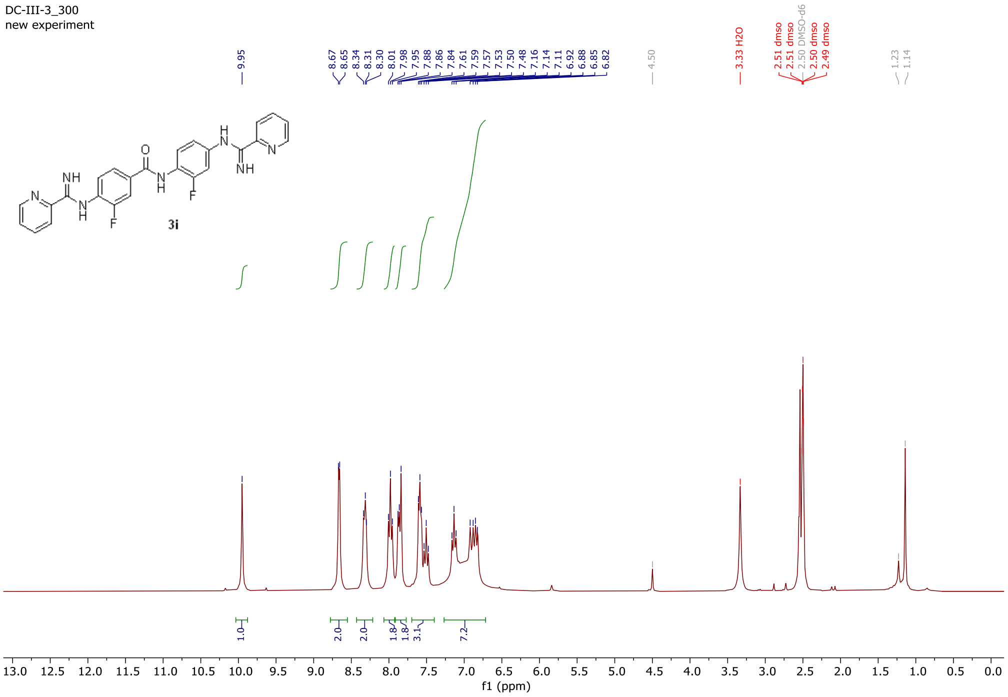

^{13}C NMR (101 MHz, DMSO-d_6)

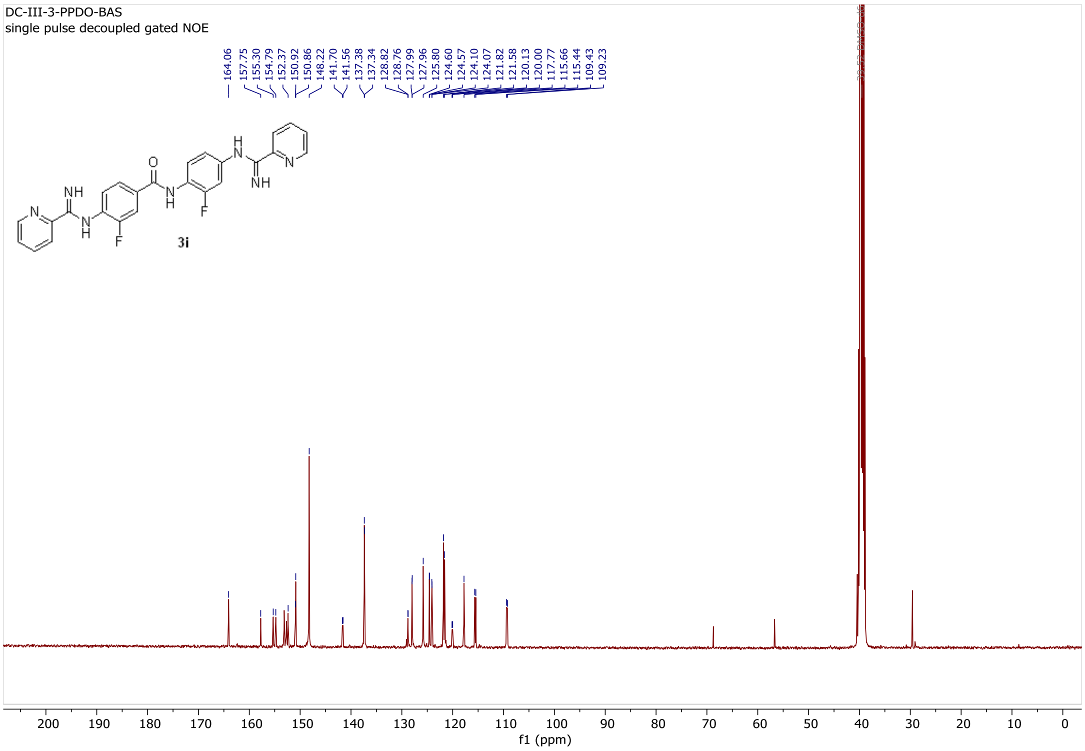

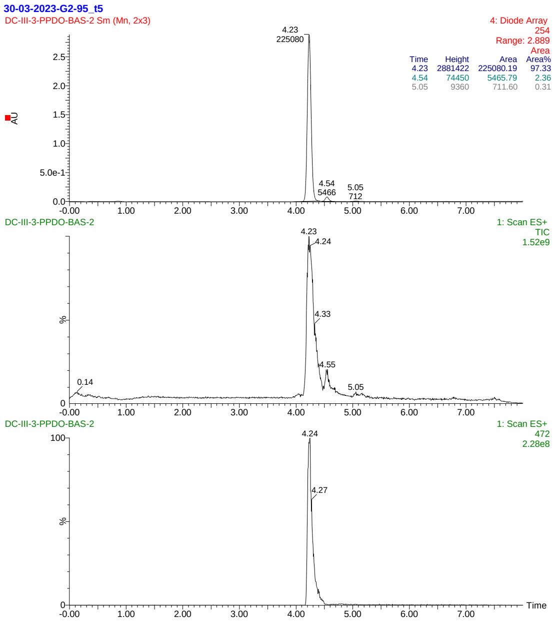

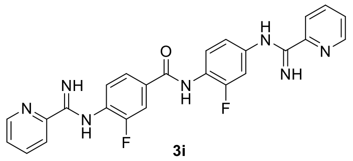

3i

Compound **3j**

^{1}H NMR (400 MHz, DMSO-d_6)

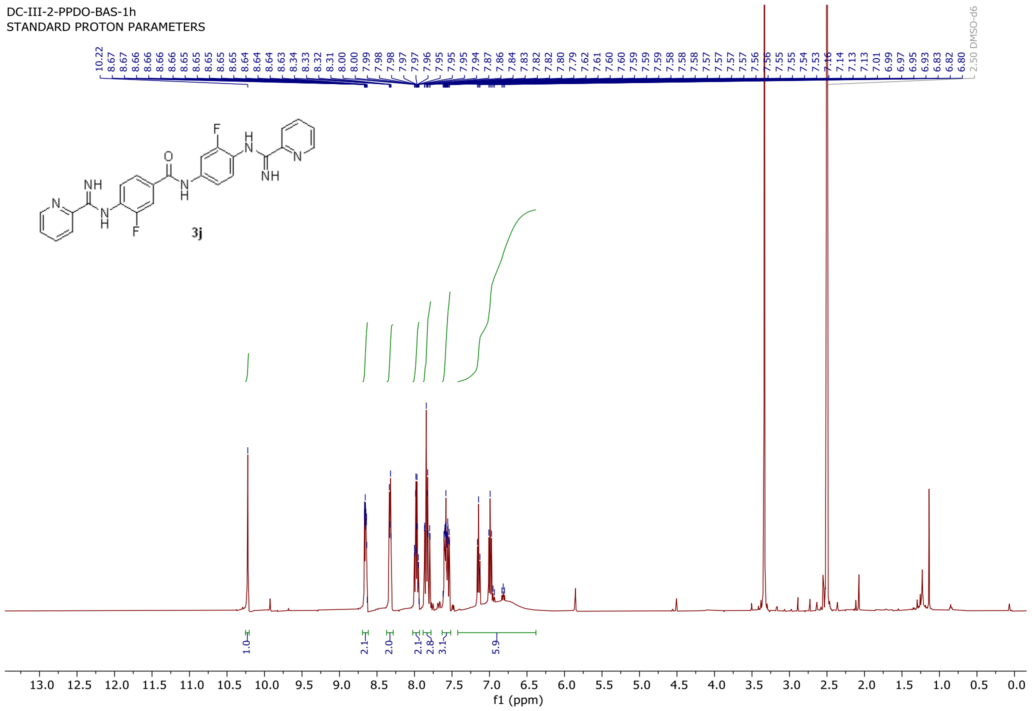

^{13}C NMR (101 MHz, DMSO-d_6)

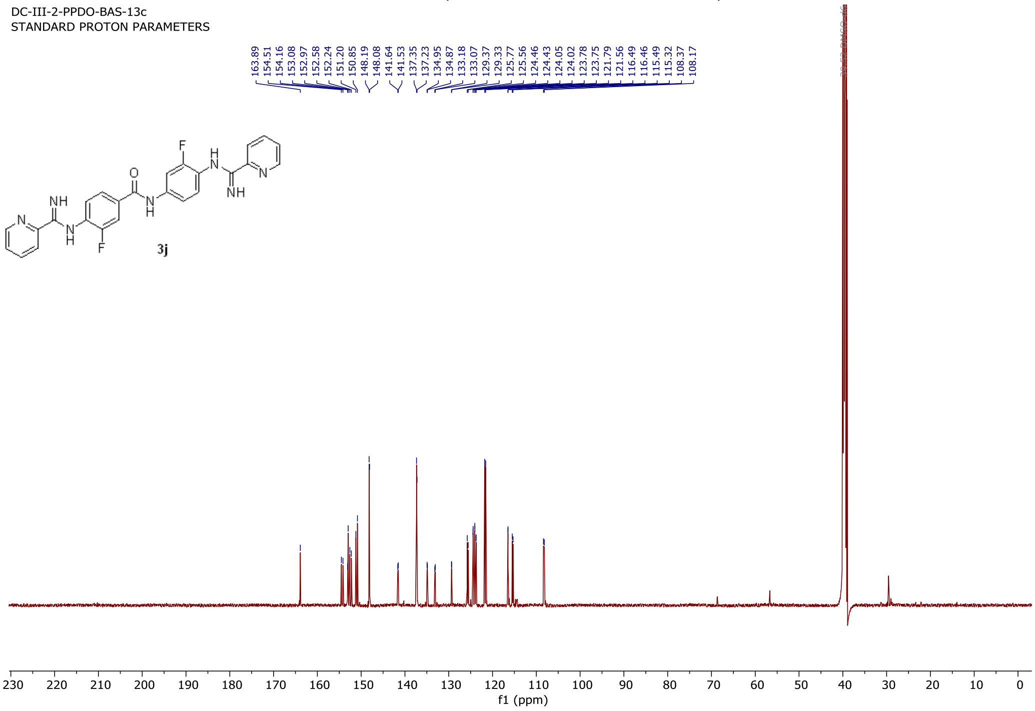

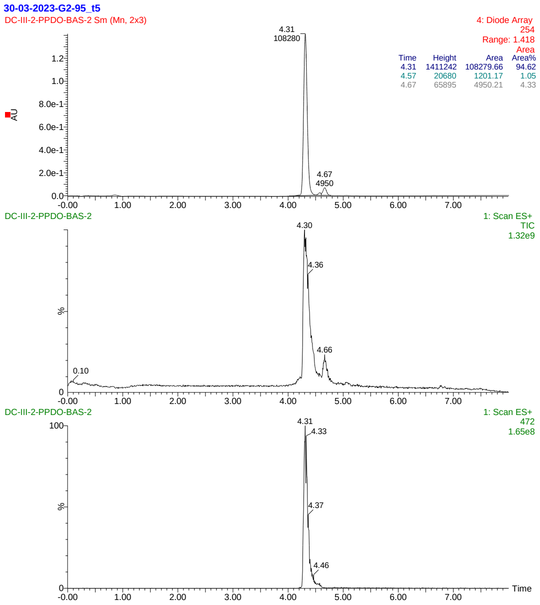

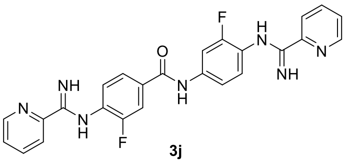

3j

Compound **3k**

^{1}H NMR (400 MHz, DMSO-d_6)

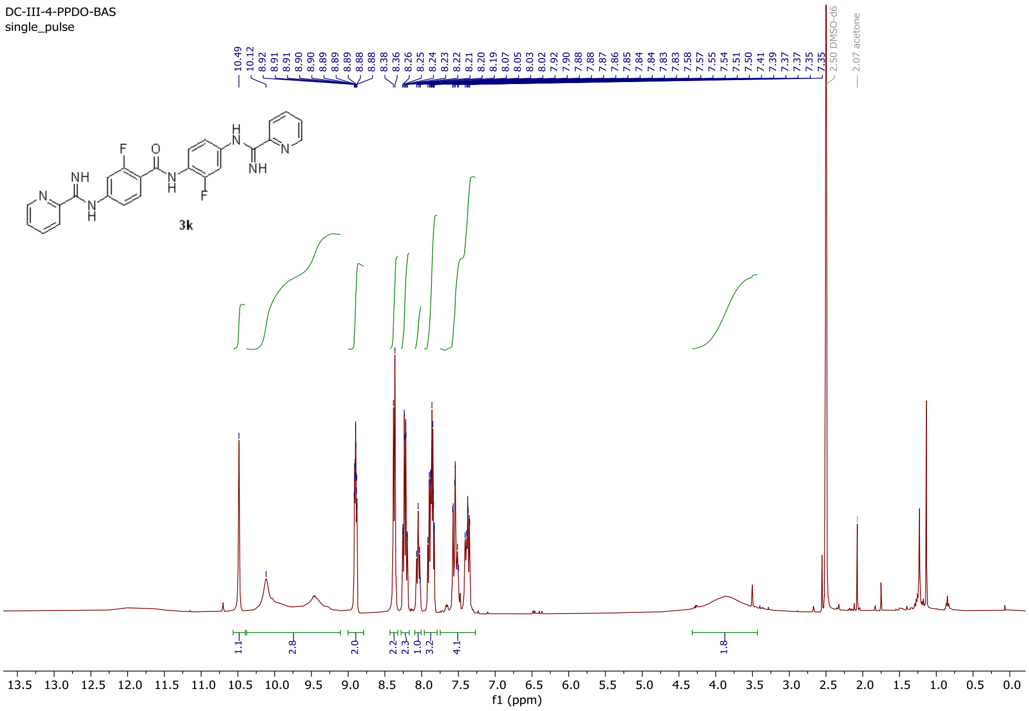

^{13}C NMR (101 MHz, DMSO-d_6)

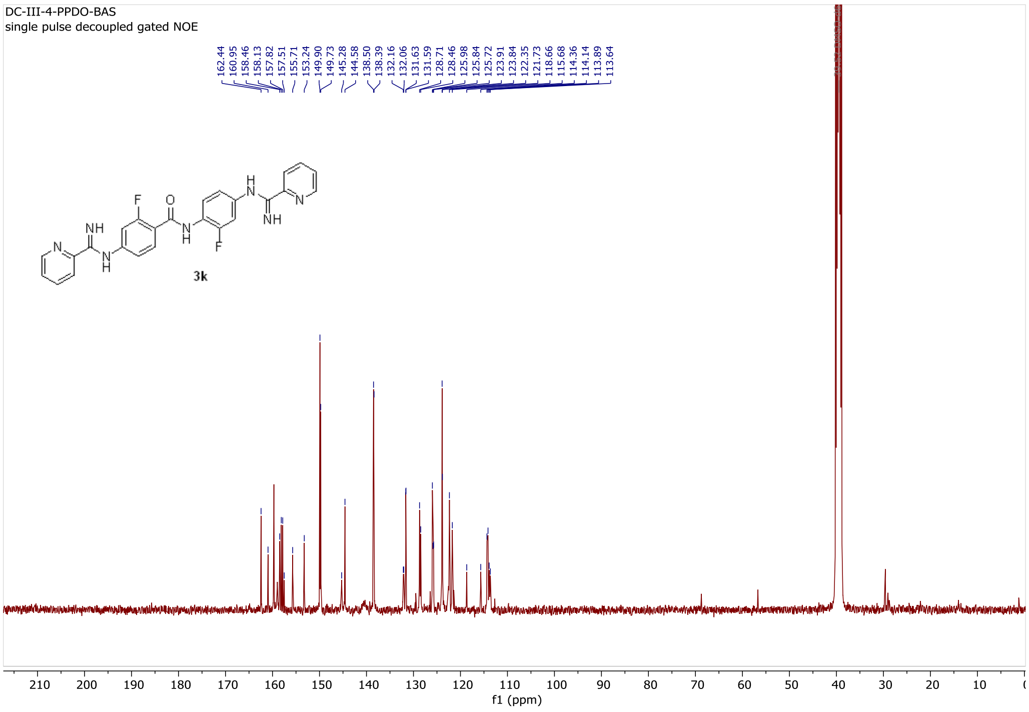

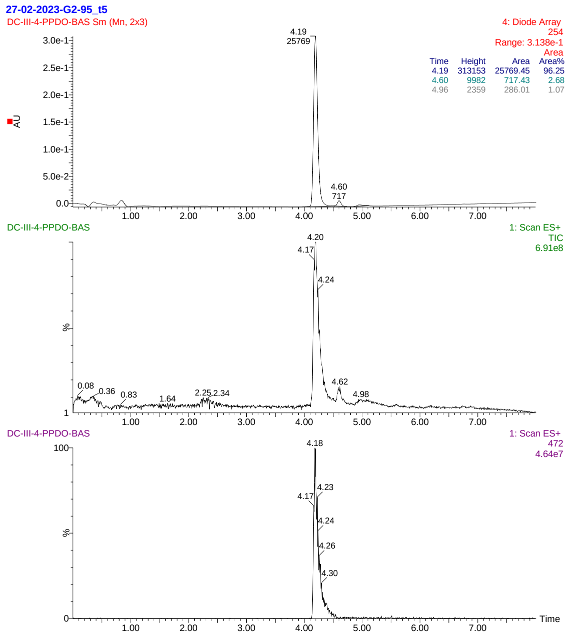

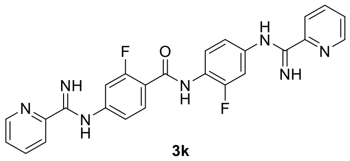

3k

Compound **3l**

^{1}H NMR (400 MHz, DMSO-d_6)

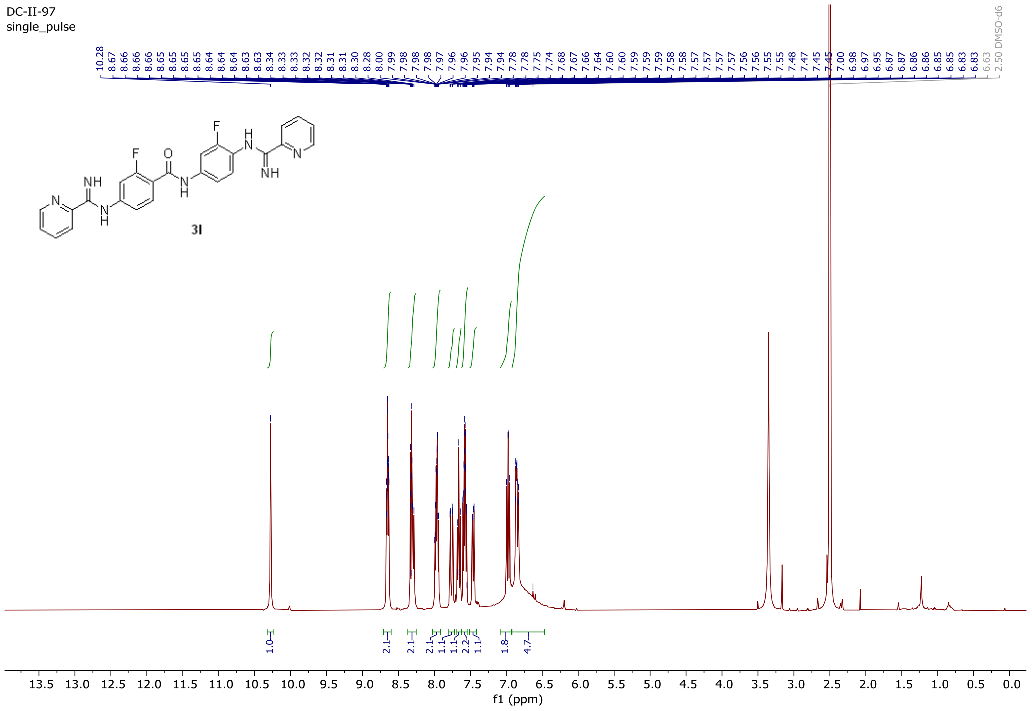

^{13}C NMR (101 MHz, DMSO-d_6)

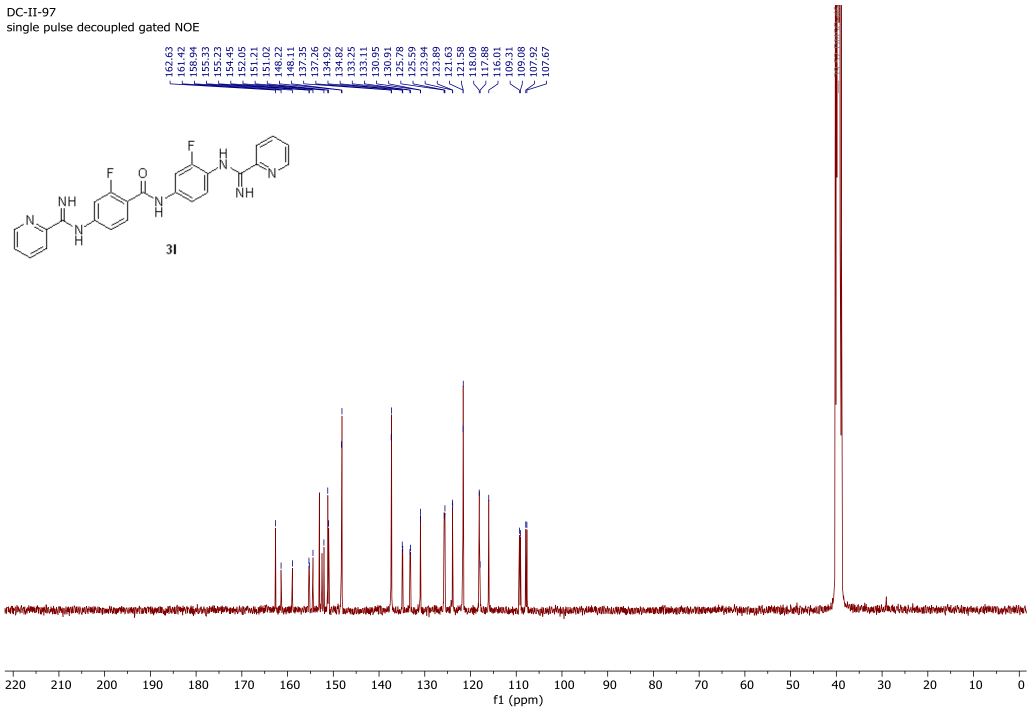

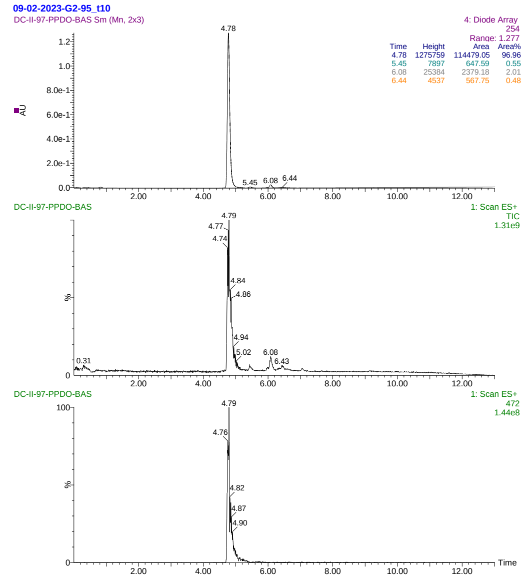

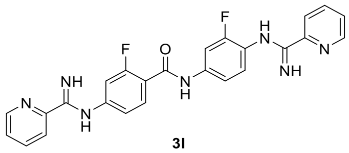

3l

Compound **18**

^{1}H NMR (500 MHz, DMSO-d_6)

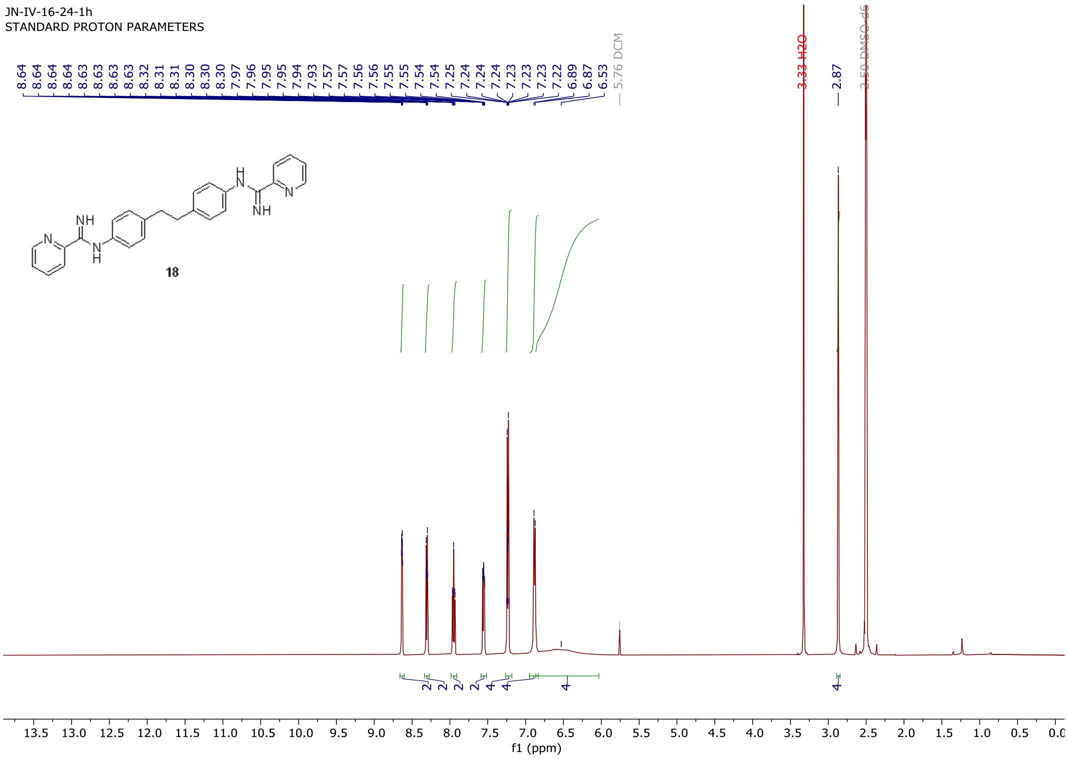

^{13}C NMR (126 MHz, DMSO-d_6

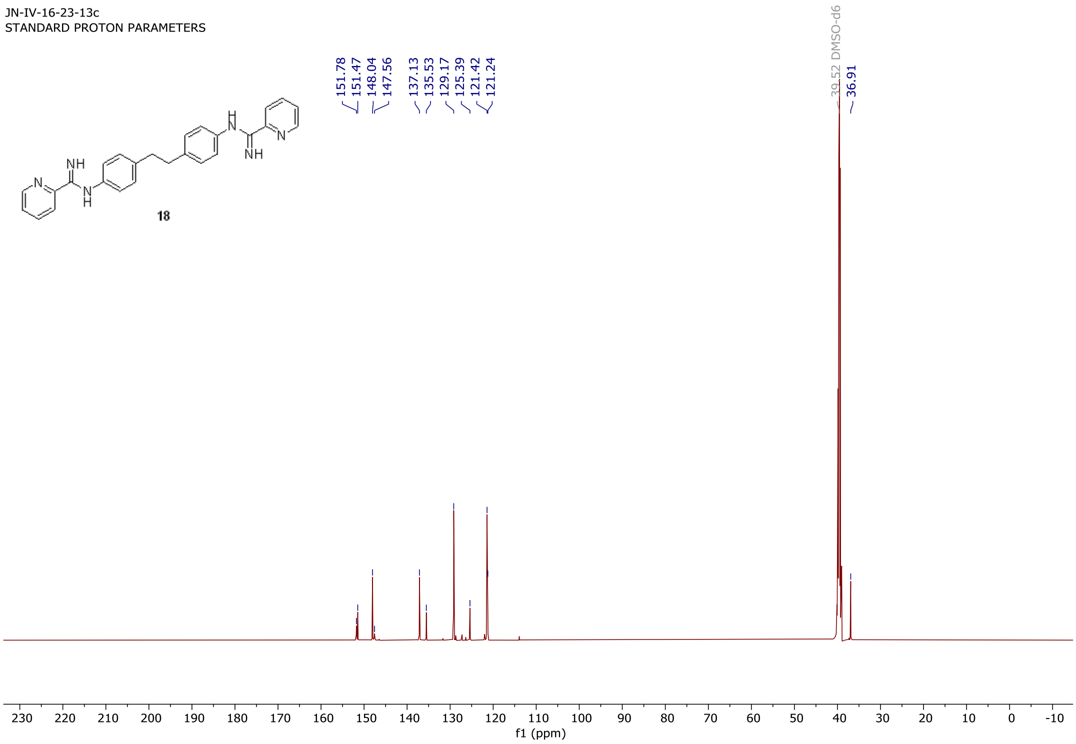

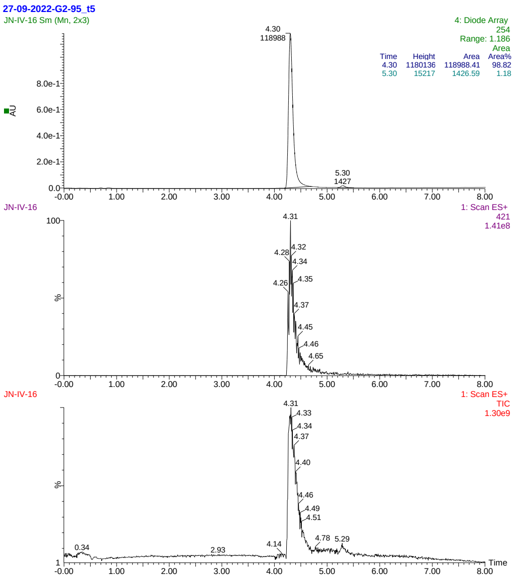

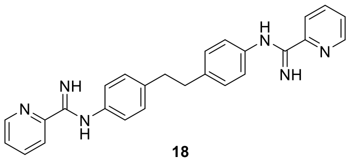

18

Compound **19**

^{1}H NMR (500 MHz, DMSO-d_6)

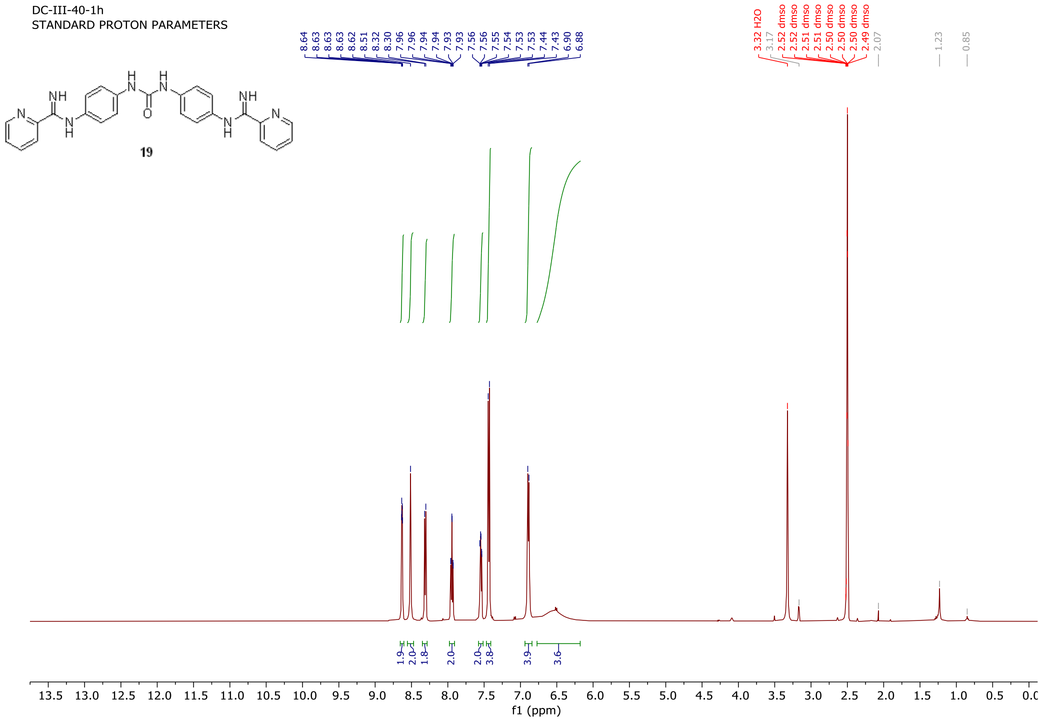

^{13}C NMR (126 MHz, DMSO-d_6)

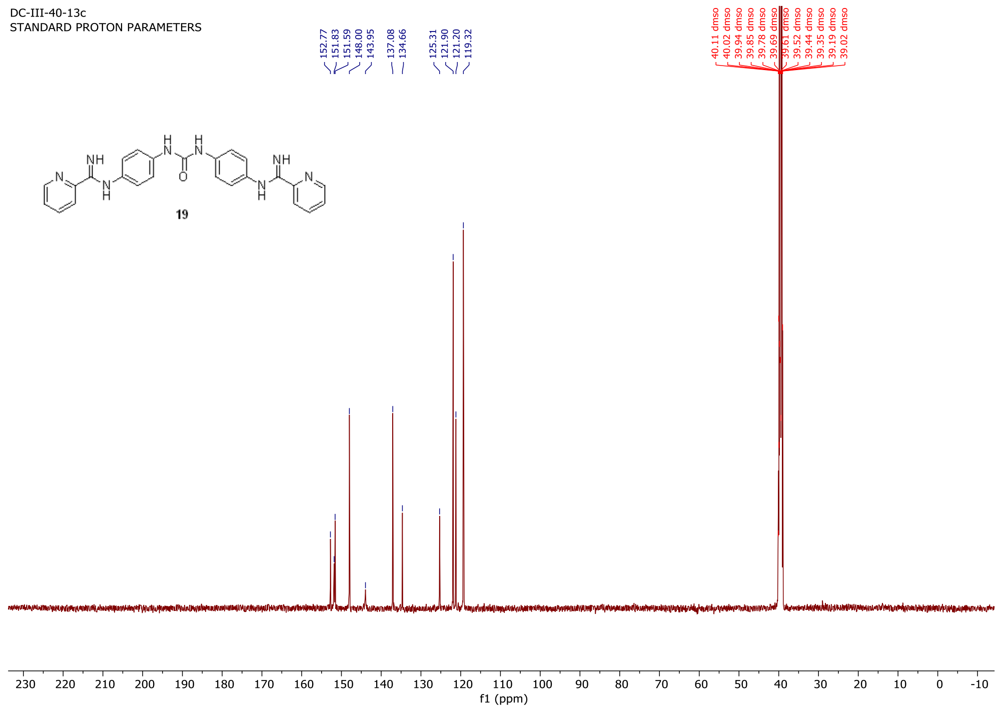

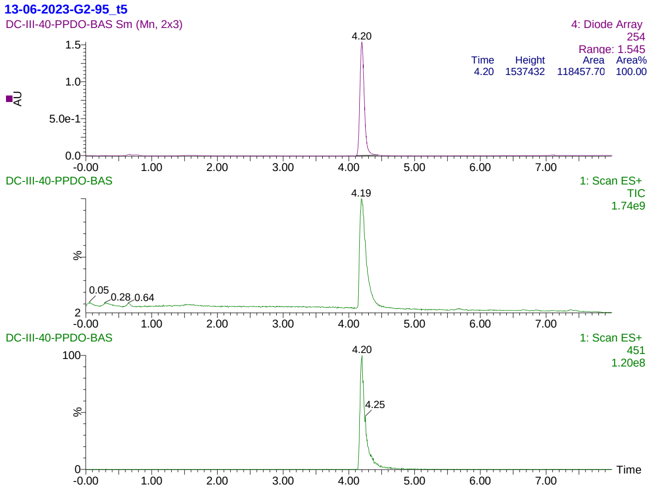

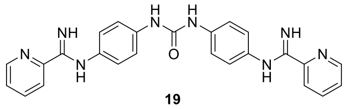

19

Compound **20**

^{1}H NMR (500 MHz, DMSO-d_6)

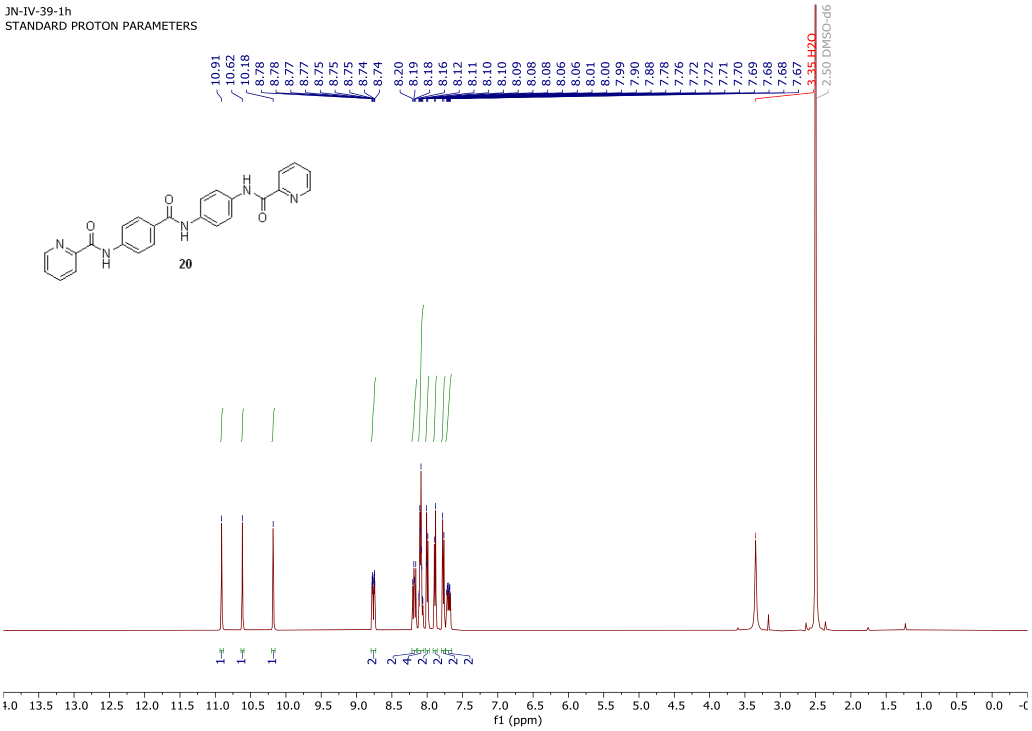

^{13}C NMR (126 MHz, DMSO-d_6)

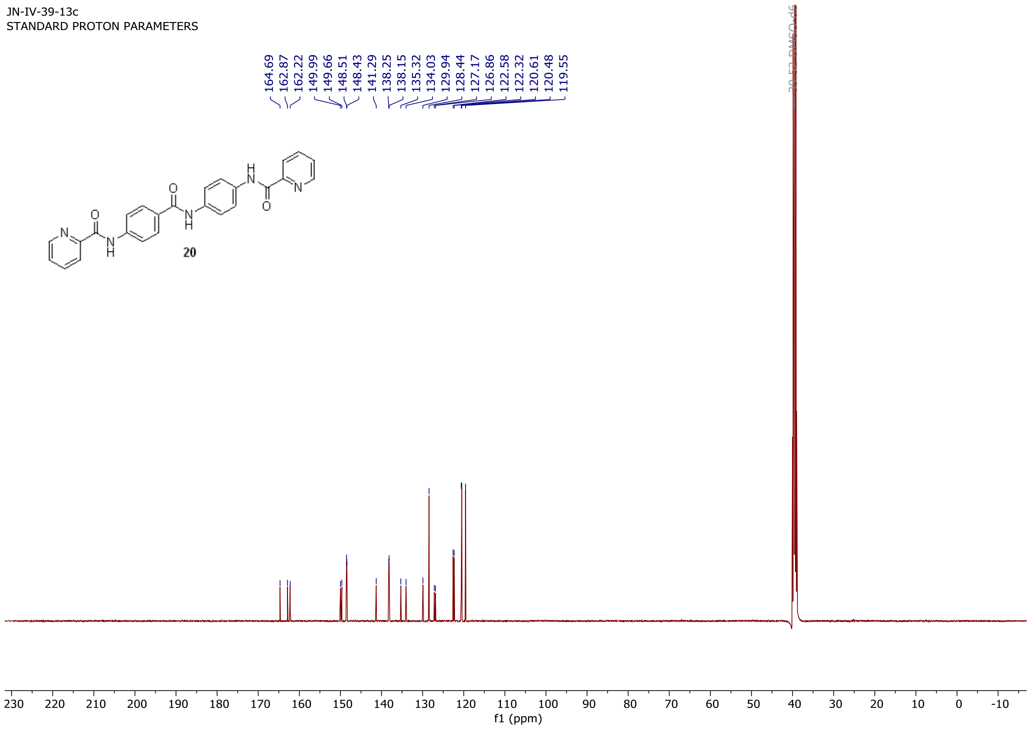

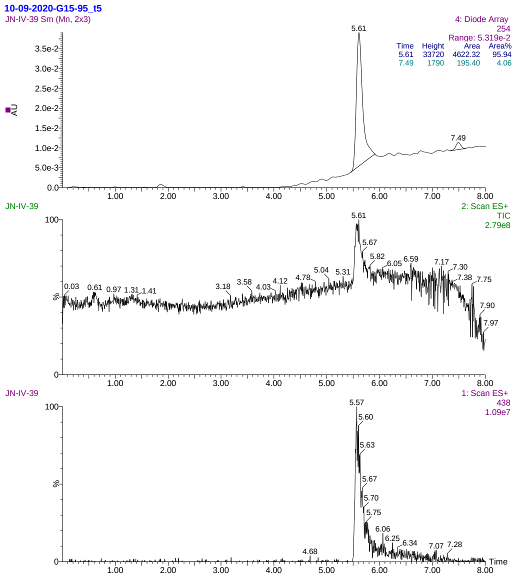

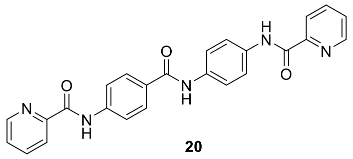

20

Compound **23**

^{1}H NMR (400 MHz, DMSO-d_6)

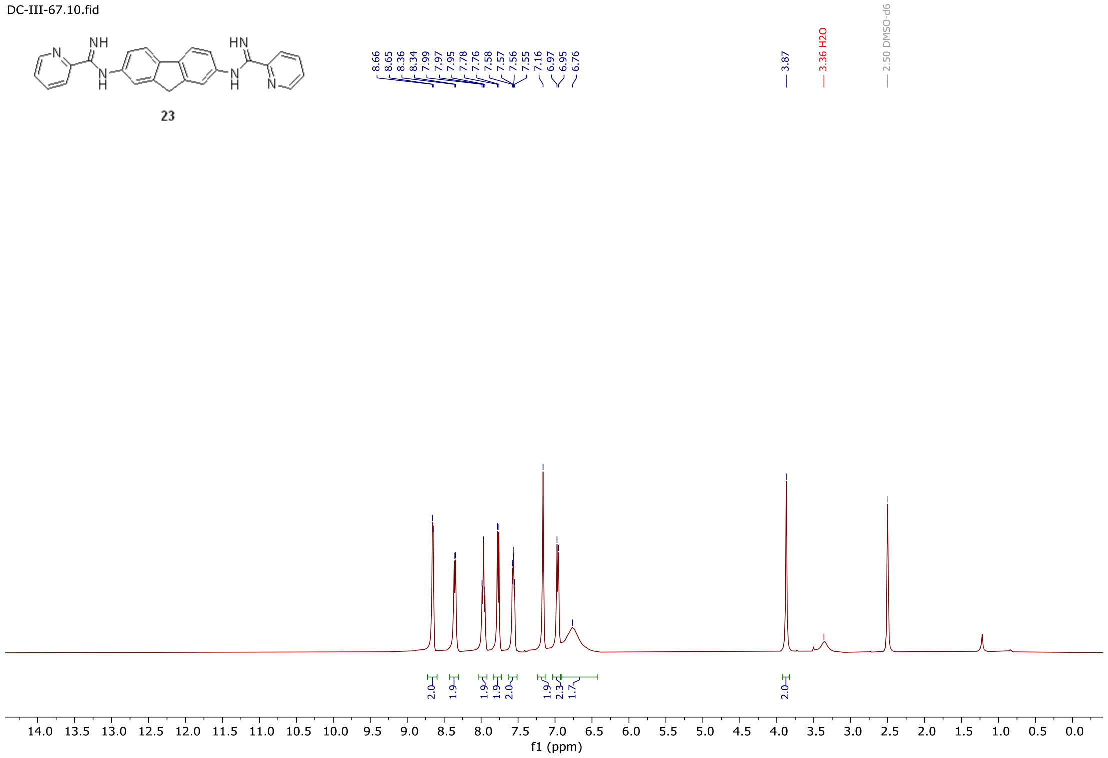

^{13}C NMR (101 MHz, DMSO-d_6)

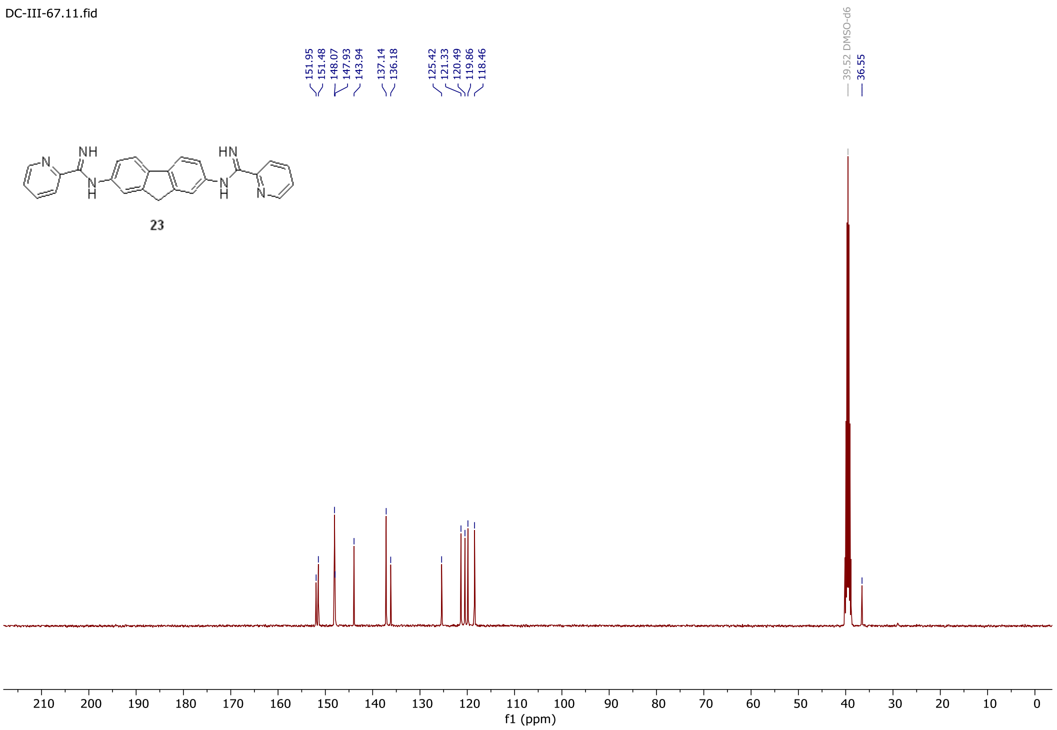

References

(1) Tse, W. C.; Boger, D. L. A Fluorescent Intercalator Displacement Assay for Establishing DNA Binding Selectivity and Affinity. *Curr. Protocols Nucleic Acid Chem.* **2005**, *20* (1), 8.5.1-8.5.11. DOI: https://doi.org/10.1002/0471142700.nc0805s20.

(2) Nué-Martinez, J. J.; Alkorta, I.; Dardonville, C. High yield synthesis of trans-azoxybenzene versus 2-isopropoxy-4-nitrobenzoic acid: influence of temperature and base concentration. *Arkivoc* **2021**, *viii*, 265-276. DOI: https://doi.org/10.24820/ark.5550190.p011.489.

(3) Ríos Martínez, C. H.; Miller, F.; Ganeshamoorthy, K.; Glacial, F.; Kaiser, M.; De Koning, H. P.; Eze, A. A.; Lagartera, L.; Herraiz, T.; Dardonville, C. A new nonpolar N-hydroxy imidazoline lead compound with improved activity in a murine model of late-stage *Trypanosoma brucei brucei* infection is not cross-resistant with diamidines. *Antimicrob. Agents Chemother.* **2015**, *59* (2), 890-904, Article. DOI: 10.1128/AAC.03958-14 Scopus.
